# Population size regulation is density-dependent in *Rhodnius prolixus* (Hemiptera: Reduviidae) through an irritability mechanism

**DOI:** 10.1590/0074-02760220211

**Published:** 2023-06-02

**Authors:** Enrique Hector Weir, Jorge Eduardo Rabinovich

**Affiliations:** 1 Ecosystems Advisors, College Station, Texas, United States; 2 Consejo Nacional de Investigaciones Científicas y Técnicas-Centro Científico Tecnológico La Plata, Universidad Nacional de La Plata, Centro de Estudios Parasitológicos y de Vectores, La Plata, Argentina

**Keywords:** population regulation, density-dependence, Triatominae, host irritability, net reproductive rate

## Abstract

**BACKGROUND:**

Physical factors can determine the level of triatomine abundance, but do not regulate their population densities, and neither do natural enemies.

**OBJECTIVES:**

To identify the processes associated with density-dependent triatomine population regulation.

**METHODS:**

We set-up a laboratory experiment with four interconnected boxes; the central box harbored *Rhodnius prolixus* bugs and one hamster. Stage 5 and adult densities of 10, 20, 30, 40, and 60 bugs per hamster, were replicated four times (except the density of 60 bugs). Hamster’s irritability and several triatomine responses were measured: feeding, development time and longevity, mortality, fecundity, dispersal, and the net reproductive value (*R*_*o*_ ).

**FINDINGS:**

Density had a statistically significant effect on irritability, but not on the percent of bugs feeding. Density was significant on blood meal size ingested in bugs that did not move between boxes, but not significant when the bugs moved. Density and irritability affected the proportion of stage 5 nymphs molting, and the proportion of adult bugs dying per day and over a three-week period. There was a highly significant effect of density and irritability on *R*_*o*_ .

**MAIN CONCLUSIONS:**

We showed that a density-dependent mechanism, acting through the irritability of the host, seems the most plausible process regulating populations in triatomines.

*Rhodnius prolixus* is one of the most important vectors of Chagas disease in several Latin American countries.^1^ However, this species has shown substantial changes in its geographical distribution over the last few decades, being completely eliminated from Central America, and from some parts of Venezuela and Colombia.

Despite thorough studies on the physiology, ethology, genetics, and ecology of this species (over 1300 research papers have been published), little is known about the possible mechanisms that regulate *R. prolixus*’ population numbers in the wild and in domiciliary environments. Environmental physical factors (mainly rainfall and temperature) are important not only in determining the ecological and geographical distribution of *R. prolixus*, but also the level of population abundance of this species; the latter results from several effects of temperature and/or humidity on physiological functions (mainly interfering or impairing with protein synthesis, cell division, differentiation, and reducing the midgut protease and the brain hormone); these physiological factors affect critical population dynamics traits such as survival,^2^ fecundity,^3^ and molting.^4^ However, it is an accepted general principle in population dynamics that environmental factors do not have the capacity to regulate population size, particularly in arthropods;^5^ effective population regulation mechanisms in triatomines should then be sought in terms of biological factors, such as natural enemies and/or its own numbers, a conclusion also arrived at for other hematophagous insects.

A review of the known natural enemies of triatomines (predators, parasites, parasitoids, and pathogens; see Supplementary data I) showed that their numerical responses do not seem to be able to cope with the population growth rate of most triatomine species, indicating that natural enemies are not capable of controlling the population size of *R. prolixus*. Thus, we should turn to density-dependent population regulation processes, and try to identify the possible main mechanisms involved.

There is an increase in nymph development time with increasing density in *R. prolixus*,^6^ which has a strong effect on the net reproductive rate (*R*_*o*_ ); however, in that experiment, *R*_*o*_ never dropped below one, despite some experimental densities being well above the equivalent of the usual domiciliary *R. prolixus* population densities.

Mechanisms of density-dependent population regulation that operate due to hosts becoming inaccessible after an irritability effect produced by high densities have been shown in some hematophagous insects such as mosquitoes and tse-tse flies; see Tatchell^7^ for a thorough review of the different mechanisms in blood-sucking host-parasite feeding interactions in arthropods. From the results of field studies with *Triatoma infestans*, the possibility of a density-dependent mechanism was suggested not to be related to host availability (which in rural houses is usually high) but rather to host accessibility.^8^ This hypothesis assumes that an increase in biting rate due to a high density of the triatomine population produces a state of nuisance and irritability in the hosts, with a series of consequences as depicted in Fig. 1 (modified from Weir^9^). The effect of dispersal as one of the possible density-dependent mechanisms was not presented in the original hypothesis by Schofield;^8^ however, in addition to the density effects upon mortality, fecundity, and developmental time for *T. infestans*, dispersal was also incorporated as an additional mechanism related to population regulation;^10^ so, we considered that its inclusion in Fig. 1 was justified. The only other experimental research on density as a factor in *T. infestans* feeding was provided by, also confirmed this effect using two densities (10 and 40 bugs/mouse) and showed that there was a decrease of the nutritional condition of bugs reared at the higher density;^11^ this author attributed (but did not measure) this effect to irritation and movement of the host impeding complete meals. The decrease in nutritional conditions of triatomine bugs was also found under domiciliary conditions: in Brazil, in a house that had a density of 134 bugs/person, on average, adult bugs bit twice as often as adults in another house that had a density of 256 bugs/person;^12^ this author summarized clearly this process, stating that “a sleeping man can probably support only a limited number of bug-bites per night before becoming restless and disturbing the bugs before they can engorge fully. Nymphs and adults may take 10 min or more to engorge fully; if feeding is interrupted before this time, only a fraction of the maximum possible blood meal is taken”. Measurements in human volunteers on the perception of the effects of an increasing number of second-stage nymphs of *R. prolixus*, showed that the mean perception score was higher the larger the number of kissing bugs biting the arm of six volunteers (densities of 0, 2, 5 and 10 bugs per volunteer were used).^13^ Interestingly, a triatomine mathematical model was proposed that explicitly incorporated a density-dependent population regulation based upon an irritability mechanism, and was tested with *T. infestans*.^14^

However, no research has specifically addressed an experimental measure of the irritability of hosts in triatomines, and an estimate of the consequences of an incomplete blood meal on mortality, fecundity, and developmental time, as factors in population regulation. Our study was designed to verify, under laboratory conditions, the host-irritability hypothesis, and for that purpose, a series of experiments were set up using *R. prolixus*, and designed to test the components identified in Fig. 1.

**Fig. 1:**
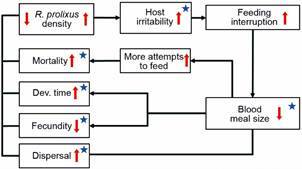
potential processes involved in the density-dependent population regulation in triatomines. Blue stars identify those processes that were measured in the laboratory. The red arrows indicate the expected direction of the change in the response of each process (increasing **↑** or decreasing **↓**) as a function of an increase in *Rhodnius prolixus* density, which was set-up by the experimenter in the laboratory design (see text for the densities used).

After reviewing the bibliography related to the environment vs density-dependence debate, we realized that most of it was relatively “old” (from 25-30 years ago), because the debate was considered settled; however, the research presented here shows relevant aspects of our knowledge regarding density-dependence that remain to be clarified.

## MATERIALS AND METHODS

*Materials and rearing conditions* - To test the hypothesis presented in Fig. 1, we used hamsters as hosts and stage 5 nymphs and adults of both sexes of *R. prolixus* as the biting insects (kissing bugs). The kissing bugs were reared in a climate-controlled room at 28 ± 1ºC and 60 ± 10% RH and fed with live chickens for one hour once a week. More details on the rearing method can be found in Rabinovich.^15^ The stage 5 nymphs used in the experiments were selected 8 - 10 days after molting from stage 4 nymphs, while the adults were selected 15 - 17 days after their emergence as adults, and were not fed since they entered either the fifth instar or the adult stage.

All experiments were carried out at the Department of Ecology, of the Venezuelan Institute of Scientific Research, Caracas, Venezuela (IVIC, for its acronym in Spanish). The hamsters were provided by the Animal Rearing House of IVIC; in order to have as much homogeneity as possible, only males within a weight range of 125 - 150 g were used. The hamsters remained in the same rearing room as the kissing bugs for one week before the start of the experiments, as an acclimation period. Being kissing bugs insects that feed at dusk and dawn, while the hamsters have a nocturnal activity, the latter were not the best selection as hosts for *R. prolixus*; however, this decision was forced by availability factors due to the need of a large and continuous delivery of host animals, and hamsters were the only species that could be steadily made available by the Animal Rearing House of IVIC. Each individual hamster was used only once in each trial.

**Fig. 2:**
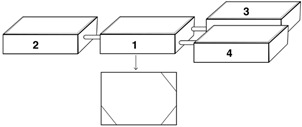
schematic drawing of the experimental set-up. The central box (box 1) is where the kissing bugs and the hamster were located to carry out the density-dependent experiments, and the three lateral boxes (boxes 2, 3 and 4) had one un-accessible hamster each, and were used to evaluate a dispersal response to bug density. The lower box is box 1 seen in a plan view to show the location of the vertical wooden partitions in three corners offered as a refuge to the kissing bugs. See text for a full description of the boxes, as well as the dimensions of the boxes and connecting tubes. Drawing by María Laura Morote of CEPAVE.

*Experimental set-up* - The experiment was set up in a system of four interconnected plastic boxes (40 x 20 x 10 cm): a central box and three lateral boxes (Fig. 2). In the central box, the kissing bugs and one hamster were put together at the start of the experiment; two sides of the central box were connected to the three lateral boxes, separated by vertical wooden partitions, with several holes that served as a refuge for the bugs from the hamster and also allowed the bugs to move out to feed on the hamster. Each of the lateral boxes was connected to the central box by plastic tubes (5 cm long, and 3 cm in diameter), that had a funnel on the side of the lateral boxes, allowing the dispersal of bugs only from the central box to the lateral boxes, but preventing their return to the central box. Additionally, one hamster was also placed in each lateral box, but isolated from the bugs by nylon and wire meshes, so that the hamsters in the lateral boxes would act only as an attractive stimulus for the bugs to move from the central to the lateral boxes, but not as an actual feeding source. All boxes had a wire mesh cover to allow for ventilation, and their bottoms were covered with sawdust to absorb the hamster’s moisture; the bottom of the corners with the vertical wooden partitions (the bugs’ refuge) was covered with filter paper.

*Experimental design* - The experimental design was based on five bug densities (10, 20, 30, 40, and 60 bugs per hamster) for the two types of bugs used (stage 5 nymphs, and adults, always used separately); four replicates per density were set-up, except for density 40 using the stage 5 nymphs, which had five replicates, and density 60, which was not replicated (there was only one trial of 60 individuals for both stages of bugs); the experiments with adults had a mix of females and males (in variable numbers). The difference in the number of replicates was due to the laboratory’s availability of bugs for the experiments. In short, we set up a total of 36 independent trials (each lasting for three successive days) in a period of nine months, that involved the use of over 1200 bugs and 150 hamsters. After the three-day trials, bugs were kept under observation for three weeks to measure the molting of stage-5 nymphs and the longevity and fecundity of adults.

*Response variables measured* - In addition to the irritability scores of the hamsters (see below), the following variables were measured in each of the three consecutive experimental days: blood intake, mortality (usually due to the hamster), and dispersal to the lateral boxes. The last two variables were obtained by direct count of dead and/or live individuals, checking them each day at the same time; the former by weighing the bugs (at the start of the day when setting-up the trial, and at the end of each of the three days) with a 0.1 mg readability Mettler precision balance. Additionally, fecundity and longevity were measured in the adults, and the molting time and the proportion of molting of stage 5 nymphs to adults. We also estimated the mortality rate of both stages; to this avail, after the trials ended their third experimental day, the stage 5 nymphs were kept under observation in separate vials (under the same laboratory conditions but with no further feeding) until they either died or molted to adults; similarly, adult females were also kept under observation in separate vials for a period of three weeks, with no further feeding, to check their survival, the number of eggs laid, and the egg hatching rate, during that period. Contrary to the irritability scores measured on the hamsters, a stress measure on the kissing bugs with increasing bug density was not carried out, because preliminary trials showed that potential stress due to the irritability of the hamster was not evident, and because triatomines are well known for their passivity, and even a tendency to aggregate, usually following conspecific feces. See Table for a summary of the variables measured.

**TABLE:** List of the variables, measured or calculated, that were used to identify the density-dependent processes in *Rhodnius prolixus*, grouped under six types of responses, and used as dependent variables with bug density as an independent variable for statistical analyses

Type of response	Variable
Feeding	Irritability
	Proportion of bugs feeding on days 1, 2, 3, and in the consecutive three days
	Proportion of bugs feeding once, twice and thrice in three days
	BMS ingested on days 1, 2, 3, and in three days
	BMS ingested on days 1, 2, 3, and in three days of bugs that did not disperse
	BMS ingested on days 1, 2, 3, and in three days of bugs that dispersed
Development time/longevity^*^	Total molting time and molting time since feeding stage 5 nymphs
	Proportion of stage 5 nymphs molting
	Longevity of adults
Mortality	Proportion dead on days 1, 2, 3 and in consecutive three days of stage 5 nymphs
	Proportion dead on days 1, 2, 3 and in consecutive three days of adults
	Proportion of females alive at the end of weeks 1, 2, and 3
Reproduction	Fecundity: eggs/female/week in weeks 1, 2, and 3
	Eggs hatched in weeks 1, 2, and 3
	Overall fecundity (eggs/female/life)
Dispersal between boxes	Proportion moved on days 1, 2, 3, and in three days
	Proportion moved on days 1, 2, 3, and in three days, that had fed
	Proportion moved on days 2,3, fed day 1 or 2, or days 1 and 2 before
Population indicator	*R’*_*o*_ (net reproductive rate)

BMS: stands for blood meal size (mg); *: development time/longevity are expressed in days.

**Fig. 3:**
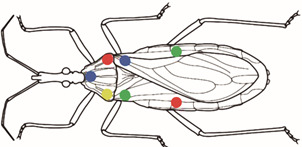
location of the seven paint marks on bugs. These marks were painted on each bug to allow for an individual follow-up of bugs during the experiments. Drawing by María Laura Morote of CEPAVE.

In order to keep track of the successive individual weights during the 3-day trials, as well as the movements between the central and lateral boxes, each bug received seven paint marks of up to four different colors. Preliminary tests showed that water soluble paints, although practical due to their quick drying time, were toxic for the kissing bugs; we tested, and finally used a non-toxic enamel paint, with good properties (spreading itself easily, and with good persistence time despite the exposure to a brush and humidity); however, the potential effects of enamel paint on the hamster’s irritability were not tested. The paint was applied as follows: one color at the base of each of the four rear legs, and three-color marks in the pronotum, producing 2,400 permutations, that allowed each insect to be individually identified (Fig. 3). Kissing bugs found dead or dispersed were replaced by the same type of insect (stage 5 nymph or adult) in order to keep density constant.

*The net reproductive rate* - The net reproductive rate (*R*_*o*_ ) is not a measurable variable but could be calculated based upon some of the variables measured and some bibliographic information. *R*_*o*_ is defined as *∑l*_*x*_ *m*_*x*_ , where *x* is age, *l*_*x*_ is the survivorship function (average proportion of individuals surviving to age *x*), and *m*_*x*_ is the maternity function (average number of eggs laid per individual at age *x*). We included *R*_*o*_ as a population indicator (for it is a demographic parameter) because one of its properties is that for *R*_*o*_ > 1, the population will show to be growing, for *R*_*o*_ < 1, the population will show to be decreasing, and for *R*_*o*_ = 1, the population remains stable. Thus, *R*_*o*_ is an adequate parameter as an indicator of population regulation. If our hypothesis is not rejected, we would expect the estimated value of *R*_*o*_ to be a function of density and/or irritability.

However, in our case, the *R*_*o*_ formula cannot be computed for each of the experimental bug densities, because we had not included nymphs from stages 1 to 4 in our experiments, nor observed the complete age-dependent egg-laying schedule of the females (they were observed for only three weeks). As a proxy for *R*_*o,*_ we used the average survival of nymphs in stages 1-4 obtained from the literature that represented a variety of environmental, density, and feeding conditions (survival values and bibliographic sources are given in Supplementary data II). So, the *l*_*x*_ values of females were computed as:

Where, ∏ is the symbol for the product operation, *S* is a general symbol for the proportion of individuals surviving, *S*_*pa*_ represents the total proportion of surviving individuals during all pre-adult stages (*pa)*, *e* stands for eggs, *n* stands for nymphs, *f* stands for females; *i* are the weeks 1 to 3, and *j* are days from 1 to 3. The final value of our estimate of the net reproductive rate is then given by:

Where, *m*_*i*_ is the number of eggs laid per female per experimental week 1, 2, and 3. The day index *j* (1, 2, 3) reflects the duration of our experimental trials (three days) that provided data on the survival of stage 5 nymphs; the week index *i* (1, 2, 3) reflects the 3-week duration of the fecundity trials that provided data on the average number of eggs laid per female per week, and also the survival during that three-week period.

There are some caveats to our definition of *R*_*o*_ (and that is why we call it a proxy and represent it by *R’*_*0*_ ): it is not the actual *R*_*0*_ of *R. prolixus*, but the most representative *R*_*0*_ value associated with our experimental set up. There are some factors that influence *R’*_*0*_ to be larger than the expected *R*_*o*_ value, while other factors influence *R’*_*0*_ to be smaller than the expected *R*_*0*_ value. This is because the *l*_*x*_ and *m*_*x*_ functions in our experiments were not evaluated for the whole life of individual females (but for a maximum of three weeks, and with no additional feeding during that period of observation), resulting in an expected *R’*_*0*_ smaller than the actual *R*_*0*_ ; on the other hand, the average survival of nymphs of stages 1 to 4 obtained from the literature does not include any effects of the density of bugs, so we expect *R’*_*0*_ to be larger than the actual value of *R*_*0*_ for *R. prolixus*. However, for the purposes of testing our density-dependent hypothesis, these *R’*_*0*_ estimates are considered acceptable because they were estimated in the same way for all bug densities.

*The irritability scores* - Essential to the hypothesis being tested was the measurement of some degree of irritability or nuisance in the hamsters. For that purpose, the hamsters were checked early in the morning on the following day of each of the three trial days, and held in the experimenter’s hand for about five minutes, with the following increasing scale of nuisance and/or aggressive behaviors, defined as associated with the following eight conditions of the hamster: (a) sleeping; (b) quiet walking and sleeping; (c) calmly walking but with some snuffling, and alternating with rest; (d) quick walking with permanent snuffling, and scratching of the bottom of the box; (e) screaming in the box, (f) screaming when being held but without biting and quickly calming down; (g) screaming when being held, biting gently or occasionally, and trying to get loose; and (h) screaming when being held, frightened, and biting strongly and permanently, and trying strongly to get loose. Although there were no controls for the irritability behavior of the hamsters, we assumed that having used a highly homogenous group of hamsters (only males between 125-150 g of weight), there was probably a very small variability in the basic behavior of the hamsters affecting our estimates of irritability. Possible degrees of grooming behavior could not be estimated by our experimental set-up (which would require permanent visual observations). Based on these eight scores, the following irritability index (*I*) was constructed:

*I =* a [0:1] + b [0:1] + c [0:2] + d [0:3] + e [0:1] + f [0:1] + g [0:2] + h [0:3] 

Where the lower-case letters represent the above-mentioned behavior scores, and the numbers in square brackets are the range of intensities observed for each behavior score (zero meaning the behavior was not present). The differences in range for each score were dependent upon the nature of the scores themselves (some types of behaviors had a wider range than others). However, some types of behavior were very specific and could not be combined with others, while other types of behavior were more general, and could be combined with other scores (and that is the reason for using a summation of scores in the index *I*); thus, although the maximum possible individual component of the score value is three, the *I* index could eventually reach values as large as seven.

The original data, as they were recorded from the experimental set-up, can be found in the file Supplementary data V.

*Statistical analysis* - The data was subjected to several statistical analyses carried out with the R language^16^ using RStudio version 1.4.1103. We used the packages dplyr (version 1.2.2), ggplot2 (version 3.4.0), ggeasy (version 0.1.3), lattice (version 0.20-45), tidyverse (version 1.2.1), tidyr (version 1.2.1), betareg (version 3.1-4), rcompanion (version 2.4.18), and AICcmodavg (version 2.3-1). For the contour plot, we used the interpolation package akima (version 0.6-3.4), and the graphical package plotly (version 4.10.1). We carried out first an exploratory analysis of the data, looking at the potential relationships between density or irritability and the variables in Table.

Some of the results showed non-linear relationships, so we resorted to several non-linear models (see below) to look at the irritability as a function of density, as well as to model other variables as a function of irritability. When the response value was a proportion or a percentage, we used the beta regression. As measures of the goodness of fit of the models, we used the sum of squares (SSQ) between observed and expected values (*∑(Obs-Exp)* ^*2*^ ), the residual standard error, or the coefficient of determination, depending upon the model being fitted. The results of the fitted model equations were presented together with the adjusted *R* ^*2*^ values (except in the case of the beta regression, where the pseudo *R* ^*2*^ values were given) as well as the 0.95 *p*-value. When more than one model was tested with the same data, we resorted to the Akaike Information Criterion, corrected for small samples to select the best model (AICc).

For the relation of irritability as a function of density, and after some exploration with several models, we decided to test two non-linear models: the natural growth (also called monomolecular) model,^17^ given by: y = a*(1-exp(-b*x)); and a second-degree polynomial model (y = a*x2 + b*x + c). For the other response variables as a function of irritability, we tried the linear relationship, but also resorted to fitting to logarithmic, exponential, and power functions. The fitting of the data to all non-linear models was carried out with the R function *nls*.

The complete computer code in R language for all analyses can be found in the file Supplementary data VI, and the code for beta regressions in the file Supplementary data VII.

*Ethics* - We followed the guide for animal care of the Instituto Venezolano de Investigaciones Científicas (IVIC), the provider of the hamsters.

## RESULTS

*The irritability scores* - We looked into the effect of bug density on the irritability score of the hamster on each day of the 3-day trials, mainly because visual laboratory observations suggested some reduction in the irritability score of the hamster to the bites during those three successive experimental days. In Supplementary data III we show these interactions, separately for stage 5 nymphs and adults. A linear regression with a “Density x Day” interaction, showed that only the effect of bug density was statistically significant (p *=* 0.021 and adjusted *R* ^*2*^ *=* 0.4642 for stage 5 nymphs and p *=* 9.2e-08 and adjusted *R* ^*2*^ = 0.4721 for adults). Linear regressions applied to each experimental day separately showed a significant effect of density on irritability for day 1, but no significant effects for days 2 or 3. In the adults the effects of the experimental day on the irritability score of the hamster is seen for all three experimental days, but showed to be significant only at densities 10 and 60. However, it is quite suggestive that, in both stage 5 nymphs and adults, density 60 shows a clear reduction of the irritability score, which is also seen in other variables; this decline suggests some degree of wearying or fatigue in the hamster in its response to the biting of the kissing bugs (or, maybe by an increased degree of weakness of the hamster due to blood drainage; see Discussion). The irritability scores were averaged for the three successive days of each trial.

**Fig. 4:**
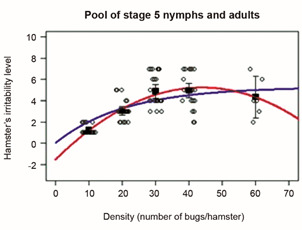
hamsters’ irritability scores as a function of bug density. This graph shows the experimental results for stage 5 nymphs and adults pooled. White circles are the original experimental values for all days and all replicates (they were slightly displaced horizontally to avoid overlap). Black squares are the experimental values averaged over all days and all replicates, and their vertical bars are the 95% confidence intervals (CI). Solid lines correspond to the fit to the natural growth model (blue; *Irr* = 5.09029*(1-exp(-0.04658*Dens)), and to the 2nd degree polynomial function (red; *Irr* = -0.0035279+0.3093827*Dens^2-1.5601374*Dens). For the second-degree polynomial fit p = 0.0197 for parameter *a*, p = 0.0136 for parameter *b*, and p = 0.1068 for parameter *c*; the *R* ^*2*^ value was 0.9812. For the natural growth model fit, p = 0.0196 for parameter *a*, and p = 0.1617 for parameter *b*, with a pseudo *R* ^*2*^ value of 0.9811 (using the McFadden approximation).

The average hamster’s irritability score as a function of bug density (for a pool of the three days of each trial) showed similar goodness-of-fit for both the 2nd degree polynomial and the natural growth models (Fig. 4). This relationship was almost identical for stage 5 nymphs and adults (sex not differentiated). We estimated the 95% confidence intervals of the experimental irritability values, for adults and stage 5 nymphs, separately; we observed that the 95% confidence intervals of both models and the experimental data overlapped completely (Supplementary data III), indicating no significant differences between models; however, the AICc was lower for the natural growth model, indicating it should be preferred over the 2nd degree model. We decided to pool the data from stage 5 nymphs and adults, and carry out a new analysis with the two non-linear models for the pooled data to obtain a single equation for each model to relate the hamsters’ irritability score (*Î*) and the bug density (Fig. 4). The resulting equations and their statistical significance are given in the legend of Fig. 4. The natural growth model stabilizes the hamster’s irritability score at high densities with an asymptotic irritability value of around 6 (model’s parameter *a*), while in the 2nd degree polynomial model the irritability decreases to zero at high bug density. As there was no experimental data of irritability scores for densities higher than 60 bugs/hamster, it is difficult to select among the two non-linear models (but the natural growth model has the attractive property that for x = 0, y = 0, as would be expected in the relationship of irritability as a function of density). Additionally, using Akaike’s index corrected for small samples (AICc), the natural growth model showed an AICc of -4.85 (k = 3), and the 2nd degree polynomial model showed an AICc of 7.74 (k = 4), where *k* is the number of parameters; thus, the natural growth model was preferred over the 2nd degree polynomial model.

Results by type of responses

*Feeding-related responses* - The bug’s feeding success is captured by the variable “proportion of bugs feeding”, which was analyzed for days 1, 2, and 3 as a function of density; those results were fitted, separately for stage 5 nymphs and adults, to a beta regression (Supplementary data IV). The effect of density on the proportion of bugs fed was statistically significant in stage 5 nymphs only for day 3 (p *=* 2.80E-05, with a pseudo *R* ^*2*^ value of 0.591), and in the case of the adults only for days 1 and 3 (p *=* 2.85E-09, with a pseudo *R* ^*2*^ value of 0.973, and p *=* 0.00568, with a pseudo *R* ^*2*^ value of 0.528, respectively). The effect of the experimental trial day was not statistically significant when all densities were pooled, neither for stage 5 nymphs (adjusted *R* ^*2*^ = 0.8394, and p *=* 0.1829) nor for adults (adjusted *R* ^*2*^ = 0.7586, and p = 0.2259). In a similar analysis pooling the trial days, the effect of density on the percent of bugs feeding was not significant for stage 5 nymphs (adjusted *R* ^*2*^ = -0.2418 with a p-value of 0.6703), but it proved to be significant for adults (adjusted *R* ^*2*^ = 0.7452 with a p *=* 0.0377).

The percent of kissing bugs feeding once, twice, or three times during the three-day experimental trials showed a similar pattern in stage 5 nymphs and adults: a decline with increasing bug density of the percent of bugs that feed only once; however, this percent increases slightly with density for the bugs that “need” a second and a third feeding on the hamster (Supplementary data IV). The beta regressions of the percent of adult bugs that fed once or twice as a function of density (for bugs that fed thrice, there were zero values, and the beta regression could not be applied) were both statistically significant (p *=* 1.69E-20, and 6.44E-45, and pseudo *R* ^*2*^ values of 0.9441, and 0.9685, respectively). For stage 5 nymphs, only bugs that fed once showed a statistically significant dependence on density (p *=* 0.0001, and pseudo *R* ^*2*^ = 0.7043). For details on these results, see Supplementary data IV.

**Fig. 5:**
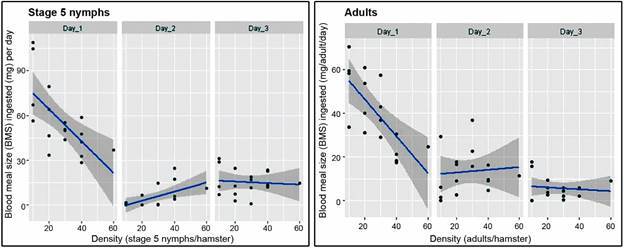
average blood meal size (BMS) ingested per individual (mg). The results correspond to each of the three experimental days (top row label), as a function of bug density (bugs/hamster), separately for stage 5 nymphs (left graph) and adults (right graph).

The results of the analysis of the average blood meal size (BMS) (mg) as a function of kissing bug density are given in Fig. 5, separately for each experimental day and for each stage. We found a decline of BMS with bug density on day 1, but a slight increase in BMS with bug density on days 2 and 3. The effects of density on BMS for any of the three-days trials was not statistically significant for stage 5 nymphs (p = 0.3083, adjusted *R* ^*2*^ = 0.0011) nor for adults *(*p *=* 0.1575, adjusted *R* ^*2*^ = 0.0208). However, after pooling all three days the effects of density on BMS was statistically significant for stage 5 nymphs (p = 0.02462, adjusted *R* ^*2*^ = 0.2326), and for adults *(*p = 0.00695, adjusted *R* ^*2*^ = 0.3539).

**Fig. 6:**
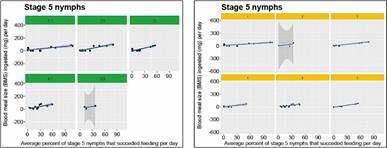
average blood meal size (BMS) ingested per individual (mg). The results correspond to stage 5 nymphs as a function of the average proportion of bugs that succeed to feed per day, and for each of the five experimental densities used (green top row label in the left-most graph), and for each average irritability score of the hamster (yellow top row label in the right-most graph). All linear regressions as a function of density were statistically significant except the one for density 60; the p-values and the adjusted *R* ^*2*^ were: p = 0.027 and *R* ^*2*^ = 0.5708, p *=* 7.248E-05 and *R* ^*2*^ = 0.7874, p *=* 3.789E-05 and *R* ^*2*^ = 0.8128, p = 0.0081 and *R* ^*2*^ = 0.4722, p = 0.5812 and *R* ^*2*^ = 0.2524, for densities 10, 20, 30, 40, and 60, respectively. The linear regressions as a function of irritability were also all statistically significant, except for irritability= 1 (p = 0.388 and *R* ^*2*^ = -0.013): p *=* 0.072 and *R* ^*2*^ = -0.6203, p = 0.072 and *R* ^*2*^ = -0.6203, p = 0.072 and *R* ^*2*^ = -0.6203, p = 0.072 and *R* ^*2*^ = -0.6203, p = 0.072 and *R* ^*2*^ = -0.6203, for irritabilities 2, 3, 4, 5 and 7, respectively.

The relationship between BMS and the average proportion of bugs that succeed to feed per day is shown for stage 5 nymphs in Fig. 6, both as a function of bug density and the irritability score; most of these models show a relatively flat slope at practically all densities; see legend of Fig. 6 for statistical significance.

**Fig. 7:**
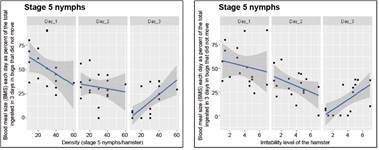
average blood meal size (BMS) ingested (mg) per day. The results correspond to stage 5 nymphs that did not move between boxes as a function of density (left-most graph), and of the irritability scores of the hamster (right-most graph).

**Fig. 8:**
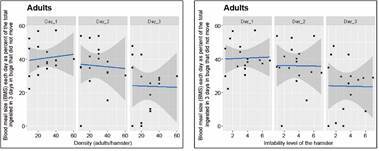
average blood meal size (BMS) ingested (mg) per day. The results correspond to adults that did not move between boxes as a function of density (left-most graph), and of the irritability score of the hamster (right-most graph).

Our density-dependent population regulation hypothesis was also associated to the possibility of moving to another box as bug density increases. The relationship between BMS as a function of both the bug density and the experimental trial day is given for stage 5 nymphs and adults (for bugs that did not move between boxes), in Figs 7-8, respectively. The right-most graph in Figs 7-8 expresses the same relationships but as a function of the irritability score of the hamster, instead of density. Similar results were obtained for bugs that moved between boxes, although the variability of the response was much larger (Supplementary data III). There was a significant effect of density (with the exception of the density of 60 bugs/hamster) on the BMS of bugs that did not move between boxes (p *=* 0.00272, 7.25 e-05, 3.79 e-05, 0.00811, and 0.581, for densities 10, 20, 30, 40, and 60, respectively), but no significance for the BMS of bugs that moved between boxes.

Independently of the bugs moving or not between boxes, we found a significant effect of density on the BMS ingested in the 3-day period for stage 5 nymphs (p *=* 0.0246, adjusted *R* ^*2*^ = 0.2326) and for adults (p *=* 0.0070, adjusted *R* ^*2*^ = 0.3539) when using the original replicates; as a function of the irritability score of the hamster, the linear model was statistically significant for stage 5 nymphs (p *=* 0.0324), but not for adults (p *=* 0.0749). However, when the replicates were averaged, there were no significant effects of density or irritability. The graphs of those linear models are shown in Supplementary data III, and for their statistical analysis based upon the beta regressions, see Supplementary data IV. Only stage 5 nymphs in day 3 showed to be significantly affected by density (p *=* 2.80E-05, pseudo *R* ^*2*^ = 0.590821), as well as adults in day 1 (p *=* 2.85E-09, pseudo *R* ^*2*^ = 0.873344), and in day 3 (p *=* 0.00568, pseudo *R* ^*2*^ = 0.5278).

**Fig. 9:**
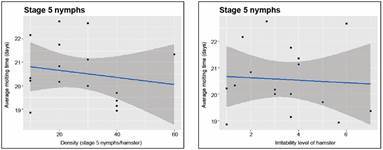
average stage 5 nymphs molting time (days). The results correspond to average stage 5 nymphs molting time into adults as a function of density (left-most graph), and of the irritability scores of the hamster (right-most graph).

**Fig. 10:**
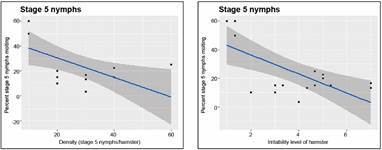
average molting success (%). The results correspond to the average molting success of stage 5 nymphs into adults as a function of density (left-most graph), and of the irritability score of the hamster (right-most graph).

*Developmental timing and longevity responses* - Figs 9-10 show the effect of density and irritability on the development time of stage 5 nymphs; the effects of density and irritability on the longevity of adults are shown in Supplementary data III. Neither the effects of density nor irritability on the average molting time of stage 5 nymphs (Fig. 9) are statistically significant (p *=* 0.514, adjusted *R* ^*2*^ = -0.03581, and p *=* 0.799, adjusted *R* ^*2*^ = -0.0619, for density and irritability, respectively). The beta regression shows that the proportion of stage 5 nymphs molting is significantly affected by both density (p *=* 0.00138, pseudo *R* ^*2*^ = 0.2261) and irritability (p *=* 0.00068, pseudo *R* ^*2*^ = 0.3212) during the three-week period of observations, and so we conclude that there is a significant reduction in the proportion of stage 5 nymphs that succeed in molting as bug density increased.

**Fig. 11:**
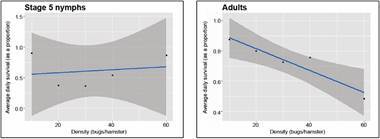
average proportion of individuals surviving. The results correspond to the average proportion of individuals surviving in a three-day period as a function of bug density, separately for stage 5 nymphs, and for adults. Each point is the average for all replicates of each bug density.

*Mortality responses* - In the mortality analysis, there were three cases of the stage 5 nymphs’ experiments in which deaths occurred during molting, and were excluded from the mortality statistics. We analyzed the effect of bug density on mortality as the average proportion of the initial density that died per day, and in the three-day period observed during the density-dependent experiments; the averages were taken among replicates. A beta regression of the effect of density on daily proportion surviving was statistically significant only for adults and not for stage 5 nymphs (Fig. 11). The p and pseudo *R* ^*2*^ values of the density effect on the proportion of adults dying were: 8.3E-09 and 0.8363; 0.0004 and 0.7263; 0.0009 and 0.6388, for days 1, 2, and 3, respectively, and 5.4E-11 and 0.8941, for the pool of the three-day trials (see Supplementary data IV for the complete results both in graph and tabular form). As stage 5 nymphs showed a non-linear relationship, we tested a second-degree polynomial, but none of the coefficients were significant (figure not shown).

**Fig. 12:**
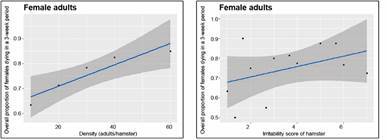
overall proportion of female adults dying. The results correspond to the proportion of female adults dying in a three-week period as a function of density (left graph), and as a function of the hamster’s irritability score.

The beta regression model of the effect of bug density on the proportion of female adults dying over a more extended period (the three-weeks of observation after the density trials were finished) (Fig. 12), was also statistically significant. The p and pseudo *R* ^*2*^ values of the density effect on the proportion of females dying were: 1.9E-08 and 0.8749, 2.69E-11 and 0.9024, 6.16E-19 and 0.9365, for weeks 1, 2, and 4, respectively, and 1.15E-25 and 0.9576, for the pool of the three-week trial; however, it was not significant as a function of irritability. Only the results for females are shown, because adult males were not followed after the three-day experimental density-dependent period.

**Fig. 13:**
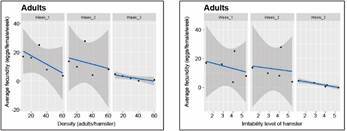
average weekly fecundity (eggs/female/week). The results correspond to the average fecundity in each week (top row labels) of the three-week observational period as a function of density (left-most graph), and of the irritability score of the hamster (right-most graph).

*Fecundity responses* - The effect of density on the reproductive performance of *R. prolixus* females (Fig. 13), expressed as the average number of eggs laid by females per week (within the three-week observational period after the three-day density experiment trials were over), was not statistically significant as a function of density (p *=* 0.3032, and adjusted *R* ^*2*^ = 0.1183), nor as a function of the irritability score (p *=* 0.5808, and adjusted *R* ^*2*^ = -0.1831).

**Fig. 14:**
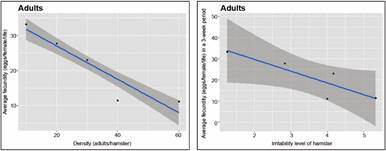
average total fecundity (eggs/female/life). The results correspond to the average total fecundity in a three-week observational period) as a function of density (left-most graph), and of the irritability score of the hamster (right-most graph).

Fecundity (*Fec*, expressed as all the eggs laid during a female’s life, *i.e.*, the cumulative three-weeks of our experiment), showed to be a statistically significant function of density (p *=* 0.0205, and adjusted *R* ^*2*^ = 0.8281), but not of irritability (p *=* 0.0534, and adjusted *R* ^*2*^ = 0.6826) (Fig. 14). The linear model of the effect of density on the fecundity per 3-weeks life was: *Fec* = 40.755 - 5.544 *Dens.

**Fig. 15:**
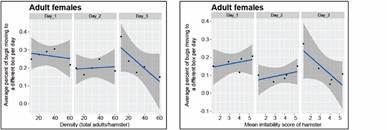
average daily proportion of female adults moving to another box. The results correspond to the average daily proportion of female adults moving to another box as a function of density (left-most graph), and of the irritability score of the hamster (right-most graph).

**Fig. 16:**
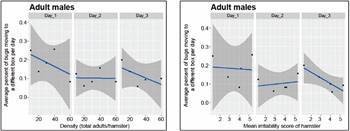
average daily proportion of male adults moving to another box. The results correspond to the average daily proportion of male adults moving to another box as a function of density (left-most graph), and of the irritability score of the hamster (right-most graph).

*Dispersal responses* - The effects of density and the irritability score of the hamster on the bug’s movements to other boxes are shown in Fig. 15 for adult females and in Fig. 16 for adult males.

Figures 15 and 16 show similar trends in the proportion of adult males and females dispersed as a function of density and irritability. Beta regression models pooling males and females showed to be statistically significant only for day 3 (p *=* 0.00259 and pseudo *R* ^*2*^ = 0.5866) and for the three days pooled (p *=* 0.0294 and pseudo *R* ^*2*^ = 0.5648), but not for days 1 and 2. As a function of irritability, the beta regression models pooling males and females showed to be statistically significant only for day 3 (p *=* 0.000479 and pseudo *R* ^*2*^ = 0.4641).

Without discriminating for having fed or not, both the proportion of adults and stage 5 nymphs moving to another box as a function of density were not statistically significant except for females for trial day 3 and for all three days (p *=* 0.00003, and pseudo *R* ^*2*^ = 0.7912, for day 3, and p *=* 0.02801, and pseudo *R* ^*2*^ = 0.5902, for the three-day period); the coefficients of the beta regressions and their goodness-of-fit statistics are given in the Supplementary data IV.

**Fig. 17:**
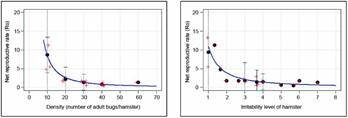
average of the net reproductive rate (*R’*_*o*_ ). The results correspond to the average of the proxy of the net reproductive rate as a function of density (left-most graph), and of the irritability score of the hamster (right-most graph). Red crosses are the original experimental values for all days and all replicates (they were slightly displaced horizontally to avoid overlap). Black circles are the experimental values averaged over all days and all replicates, and their vertical bars are the 95% confidence intervals (for Density =10, and Irritability = 1, due to the highly variable the *R’*_*o*_ values, the y-axis was truncated to facilitate visualization). Solid blue lines correspond to the fit to the power model (*R’*_*o*_ = 464.361*Dens ^-1.731^), and *R’*_*o*_ = 10.9319*Irrit ^-1.4834^). The p-values of the *b* coefficient of the power model were highly significant: p = 0.18483, and p = 0.00563, for *a* and *b* for *R*_*0*_ as a function of density, and p = 2.01e-05, and p = 0.000536 for *R*_*0*_ as a function of irritability.

*Population growth rate response* - Fig. 17 shows the results of the estimates of *R’*_*o*_ as a function of density and irritability, and their fit to a power model, that proved to be statistically significant with p *=* 0.0056 and 0.0005, with *R* ^*2*^ values of 0.7860 and 0.7291, for density and irritability, respectively.

**Fig. 18:**
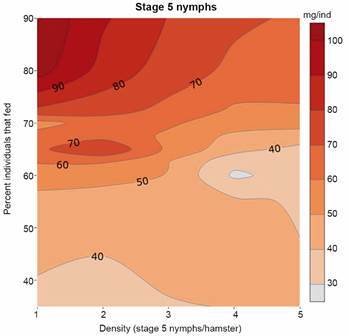
combined effect of the proportion of stage 5 nymphs that fed and density of stage 5 nymphs/hamster, on blood meal size (BMS). The numerical values of the contour lines (as well as the figure’s colour scale) correspond to BMS (average in mg/individual). The *x-*axis is an experimental density index (x= 1, 2, 3, 4, 5) that corresponds to Densities = 10, 20, 30, 40, and 60, respectively). All variables depict the results for trial day 1.

Despite the fact that it is known that for *R*_*0*_ > 1, the population grows; for *R*_*0*_ < 1, the population declines, and for *R*_*0*_ = 1, the population stays stable, as we could not estimate *R*_*0*_ directly but used *R’*_*0*_ as a proxy of *R*_*0*_ (a still acceptable one), we decided not to compare the density-dependent *R’*_*0*_ values in relation to the value of 1, as could have been done with the power model fit shown in Fig. 18.

*The interruption of feeding* - The feeding interruption process as depicted in Fig. 1 could not be measured directly, but to have a representation of that process, we can use as a proxy the combined effect of two measured variables: the percent of fed individuals and the blood meal size; their combination is an indicator of a subjacent interruption of the feeding process (fewer bugs feeding less blood). Fig. 18 is the graphical result of such a combination in a contour plot limited to stage 5 nymphs, and trial day 1; it shows that the interaction between the percent of fed individuals and the BMS is very strong for densities below 30 - 40 stage 5 nymphs/hamster (lines are more or less in a diagonal); however, those lines become almost horizontal above 30 - 40 stage 5 nymphs/hamster, indicating a dominant effect of the percent of fed individuals on the BMS.

*Hamster’s irritation results from behavior or blood drainage?* - Our hypothesis being that the irritability of the host is the fundamental mechanism triggering the population density-dependent processes, we analyzed the effect of density on the irritability in more detail, focusing on the declining trend of irritability along the three days of experimental trials, that becomes more conspicuous as density increases (Fig. 4 and Supplementary data III). To that avail, we assessed if the amount of blood drained from the hamster might explain this result, through a debilitating effect on the hamster.

**Fig. 19:**
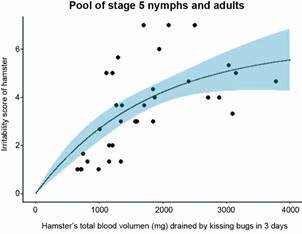
irritability score of the hamsters as a function of total blood volume drained (mg) from the hamsters. The results correspond to the average irritability score of the hamsters, using a pool of stage 5 nymphs and adults, in the 3-days experimental period, as a function of total blood volume drained from the hamsters. The black dots are the laboratory values for each density and each replicate. The black line is the fit to a natural growth (monomolecular) model of the form: *y* = a*(1-exp(-b*x)); the value of the parameter *a* (asymptotic irritability score for very high blood drainage) was 6.17 and was statistically significant (p = 0.000251); the value of the parameter *b* (rate of increase of the irritability score with blood drainage) was 0.00058 and was also statistically significant (p = 0.0308). The light blue area is the 90% confidence interval calculated from the natural growth model.

The irritability (*Irr*) score of the hamster as a function of total blood volume drained (mg) from the hamster (*BD*) by a pool of stage 5 nymphs and adults, in the 3-day experimental period, is shown in Fig. 19, together with the fit to the natural growth (monomolecular) model. The fitted equation is *Irr* = 6.173*(1-exp(-0.00058*BD)); both the parameter *a* (asymptotic irritability score for very high blood drainage) and the parameter *b* (rate of increase of the irritability score with blood drainage) were statistically significant (p *=* 0.000251 and p *=* 0.0308, respectively; pseudo *R* ^*2*^ = 1.96e-11, with McFadden approximation). The pool of stage 5 nymphs and adults was done for fitting purposes, but the experimental values of each stage were independent. The irritability score of the hamster tends to flatten out around a level of 6.2 (the theoretical maximum if *I* is 7), independent of how much blood was drained from the hamster.

The amount of blood drained from the hamster seemed to reach a high value (3000-4000 mg of blood) for high kissing bug densities (particularly for stage 5 nymphs). Hamsters have an average of 78 mL of blood/kg of live weight; as the average weight of the hamsters in IVIC’s experiments was 125-150 g (median 0.138 kg), the hamster’s blood volume is 78*0.138 = 10.8 mL; as the density of whole blood is 1.0565 (at 37ºC), then the hamster’s blood mass is 11,410 mg. Our results provided the BMS ingested by stage 5 nymphs and adults at each density accumulated in the first three days of the experimental trials for each replicate (see Fig. 19 and Supplementary data III). From the pool of stage 5 nymphs and adults, the minimum total BMS drained from the hamster was 706 mg, and the maximum total BMS drained from the hamster was 2,891 mg; these values represent 6.2% and 25.3% of the total blood mass of the hamster, respectively. However, it has been recommended that, to maintain a laboratory hamster healthy, if multiple small samples are extracted daily from it, those extractions should not be above 0.01 mL daily,^18^ that is, 0.01* 1.0565 = 0.010565 g daily, or 31.7 mg in three days. This means that, even at the lowest kissing bug density used in our experiments, the exposed hamsters were blood-drained well above the NC3Rs recommendations.^18^

## DISCUSSION

The main points emerging from our results revolve around a series of complex processes: host irritability, promoted by increasing bug density, leads to a reduction in blood intake by the bugs, leading in turn to a series of consequences such as changes in development time, female fecundity, and adult dispersal (with host irritability apparently associated with host blood-loss). Due to the complex interactions among these processes, we discuss the results below under several headings.

*General comparison with previous density-dependence data in triatomines* - The feeding status in triatomine populations can be considered the average feeding level reached by the individuals of such a population, as a response to the availability and accessibility of food resources;^12^ this feeding status may affect some of the life-history traits and population demographic parameters, thus resulting in a density-dependent population regulation.^8^ ^,^ ^9^ A density-dependent mechanism of population regulation for triatomines, by which an increase in bug densities will reduce the accessibility to food resources as a consequence of an increase in host irritability, which in turn may reduce the blood ingestion process by the triatomines was shown by.^8^ This author predicted that when food is inaccessible (in the sense of unapproachable), the resulting reduction in nutritional status of triatomines will decrease fecundity, and increase development time and mortality, leading to a reduction in the population size, and thus constitute a density-dependent population regulation process. Our experimental work confirmed most of those proposed mechanisms,^13^ and also added the potential influence of dispersal as another mechanism of density-dependent population regulation in *R. prolixus*, though it proved to be only marginally significant (with the exception of stage 5 nymphs).

Contrary to our results, a statistically significant effect of density of *R. prolixus* on the developmental rate of 2nd, 3rd, and 4th nymph instars was found, but not on the nymph survivorship;^6^ those authors also found no density effect on the net reproductive rate (*R*_*o*_ ), while we did obtain a strong significant effect with our proxy (*R’*_*o*_ ). The differences between our results and those of^6^ may derive from differences in the experimental conditions; the latter used: (i) chickens as a food source (a much larger host), (ii) a smaller container (a glass jar ≈ 4 L, half of the volume of our boxes, which had a volume of ≈ 8 L), and (iii) the highest *R. prolixus* density (128 bugs) was about twice our highest density (60 bugs). However, even accepting that these differences could influence the numerical values of the results, it is remarkable that, with few exceptions, no density effects were obtained.^6^ Looking at the experimental procedures of these two experiments, we arrived at the conclusion that a possible explanation to the differences in their results is related to the conditions of the hosts during the experiments: In their experiments^6^ the chicken was placed in a wooden box that had a hole in the bottom where the jar with the bugs was placed to allow feeding; but the chicken was restrained with a light towel, so that it was extremely limited in its movements, reducing the potential irritability response.

*The irritability response* - In our experimental design, the minimum density was set to 10 bugs/hamster; this density was selected because we believed that the hamster’s irritability response would begin to show above that density. However, from the results, it became apparent that 10 bugs/hamster already produced a certain degree of irritability in some variables. In adult *R. prolixus,* the effects of the experimental day on the irritability score of the hamster are seen only at two densities (10 and 60 bugs/hamster). However, it is quite suggestive that, in both stage 5 nymphs and adults, density 60 shows a reduction of the irritability score, which is also apparent in some response variables, suggesting some degree of “saturation” in the hamster’s reaction capacity to the biting of the kissing bugs (or, maybe simply, an increased degree of “weakness” of the hamster due to blood drainage). Unfortunately, as in our experimental design, the 60 bugs/hamster density level had only one replicate, so we cannot put too much confidence in this apparent reduction in irritability; so, we cannot discriminate if high bug density levels could stabilize irritability as suggested by the natural growth model or decrease towards zero as suggested by the second-degree polynomial model.

The question if the amount of blood drained from the hamster might explain our results through a debilitating effect on the hamster cannot be clearly answered. Despite the fact that the bug densities used resulted in hamsters being blood-drained well above the NC3Rs^18^ recommendations, the statistically significant fit to the natural growth model (Fig. 19) suggests that, at high bug densities, the irritability score tends to stabilize around a value of 6, while from a debilitating effect of high bug densities on the hamster, we would expect a further decrease of the irritability score. Our results would suggest that, if irritability is the expression of some degree of “behavioral adaptation” due to a weakening, wearing out, or “giving-up” of the hamster to the bites, then it is not due to a weakening of the hamster caused by blood drainage but rather a behavioral reaction to the kissing bugs biting. This interpretation is consistent with what it was found in human volunteers:^13^ the mean perception score was higher the larger the number of second-stage nymphs of *R. prolixus* biting the arm of six volunteers when densities of 0, 2, 5, and 10 bugs per volunteer were used.

*The feeding response and its consequences for population dynamics* - Our experimental results showed that, through a reduction in the feeding status, an increase in the density of *R. prolixus* may have several consequences for triatomine population dynamics. Below, we discuss some of those results and concomitant issues.

Our result of a significant reduction in BMS ingested per female with increasing bug density, resulted in a decrease in female fecundity. This relationship is consistent with the findings in *R. prolixus* and *T. infestans* indicating that fecundity decreases with a decrease in the blood intake;^10^ ^,^ ^19^ furthermore, it was showed that in *T. infestans* females there is a threshold of about 35 - 40 mg of blood ingested in order to be able to lay eggs;^20^ a similar and slightly higher threshold (55 mg of blood ingested) was found for *R. prolixus*.^19^ If we partition our data above and below that threshold of 55 mg of blood ingested found by^19^ for *R. prolixus*, we see that below the 55 mg threshold, the mean BMS is 32.4 mg (sd = 17.1; coef var = 52.8%), and the mean eggs/female in a three-week period of observation is 10.5 (sd = 7.1; coef var = 67.6%), while above the 55 mg threshold, the mean BMS is 88.3 mg (sd = 21.5; coef var = 24.4%), and the mean eggs/female in a three-week period is 31.1 (sd = 15.7; coef var = 50.5%). That is, the mean eggs/female in a three-week period of observation is about one third smaller below the 55 mg threshold (10.5 eggs/female) than above it (31.1 eggs/female). The bug density effect on fecundity can be considered equivalent to a reduction in the feeding frequency, which has been shown to decrease some reproductive parameters such as fecundity and the number of reproductive weeks, as occurs in *T. patagonica* and *T. infestans*.

We also have to consider the autogeny factor as influencing our results. *R. prolixus* shows autogeny (adult females may lay a few eggs even without feeding as adults, using the remnants of blood ingested as stage 5 nymphs); additionally, this autogeny is very sensitive to the blood source: the 55 mg threshold in *R. prolixus* was obtained using rabbit blood,^19^ and our own experiments used hamster blood, so we cannot establish the source of the difference in relation to the BMS threshold and its effect on egg-laying. From our analysis, the response of the number of eggs per female in a three-week period of observation as a function of density, as a function of blood meal size, and as a function of irritability were all statistically significant; this is strongly revealing, because those three factors (density, blood meal size, and irritability) are at the heart of the density-dependent population regulation hypothesis.

No effects on egg hatching were found in our bug density experiments. The reduction we observed in the amount of blood ingested as a consequence of an increasing bug density, suggests that even the few eggs laid with the ingestion of very small amounts of blood are able to complete their normal development.

Contrary to prediction,^8^ we found that the effects of density and irritability were not statistically significant on the average molting time of stage 5 nymphs. However, the proportion of stage 5 nymphs molting into adults was significantly reduced, as a function of both density and irritability, during our three-week period of observation. The lack of response in the stage 5 nymphs development time but a reduction in the proportion of stage 5 nymphs molting, makes sense due to the nature of the molting process: an “all or none” mechanism, where the blood ingested produces a longitudinal stretch of the dorsal intersegmental muscles of the abdomen, triggering the molting process. It was shown^19^ that the minimum blood meal that promoted molting in stage 5 nymphs of *R. prolixus* varied between 43.7 and 75.8 mg; resorting to the linear equation of our results for stage 5 nymphs (BMS = 99.0165- 0.8132* Density), those minimum blood meals promoting molting^19^ would imply a density of 68 and 29 stage 5 nymphs/hamster, respectively. In a laboratory experiment^21^ using mice as hosts and the bug *Panstrongylus megistus*, it was found a significant decrease in the percent of stage 5 nymphs molting with increasing densities of 12, 18, and 36 bugs/mouse (% molt = 3.8405*Density + 82.032; *r* = 0.945).

It has been suggested^10^ that in *T. infestans*, the reduction in BMS represents an adaptive advantage by keeping a high population density at a given time, by a separation of the feeding opportunities between individuals in the population; however, although this may be so, in the end the population is stabilized because of the reduction in fecundity and survival. It was also shown^10^ that, under domiciliary conditions, the difference between the reduced BMS (related to a “threshold hunger”, as called by^10^) and the “normal” blood meal size avoided dispersal and death (unless the food resource disappeared completely). Our experiments only partially confirm this, for there was an effect of density on adult mortality in the first three days of our experiments and over the longer period of three weeks, but not in stage 5 nymphs. However, it was considered that mortality was of very little relevance in density-dependent interactions for *T. infestans*.^8^ On the other hand, in a laboratory experiment using mice as hosts and the bug *P. megistus*, a significant increase in mortality was found^21^ with increasing densities of 12, 18, and 36 bugs/mouse (% mort = 1.1904*Density - 8.5885; *r* = 0.974).

The results of the dispersal of bugs between boxes do not follow previous predictions:^10^ there was no significant effect of density on dispersal (and even a slight effect with a negative sign: higher dispersal at lower densities). It was found that the proportion of adult *T. infestans* initiating flight depended on the nutritional status of the insects, and that flight activity was greatest about 12-18 days after feeding.^22^ However, no studies of that type were carried out with *R. prolixus*. Additionally, our results didn’t show any significant density effect in the relationship between BMS and moving to another box. However, these comparisons have to be taken with strong reservations for, in addition to the difference between triatomine species: (a) in our experiments, we observed movements only for three days (a much shorter period than the one determined for *T. infestans*),^22^ and (b) there is an important difference between dispersing by flight,^22^ and by walking (our experiment with the hamsters).

*The irritability mechanism* - We cannot end this discussion without referring to another component related to irritability: the effect of the blood intake by biting bugs on the hosts in terms of an inflammatory process. During their biting, certain kissing bug species produce in their hosts an allergic effect. However, triatomines also have in their saliva a substance that can decrease the irritation on the host. Additionally, a direct action on the nervous activity of the host has been described in *T. infestans*. Triatomines are also extremely efficient in finding blood vessels, thus reducing the number of probes on the host, which, together with the decrease of the inflammatory reaction caused by the saliva released shortly after the bite, strongly decreases the irritation of the host. However, such an evolutionary-adaptive strategy by triatomines to minimize the irritability of the host, would be at odds with the crucial role of irritability in our hypothesis of density-dependent population regulation. We will try to reconcile this apparent conflict in the conclusion section. Finally, our experimental set-up was not able to measure other concomitant variables, such as those of cannibalism among triatomines (or more correctly, “kleptohemodeipnonism”), or the potential effects of predation and parasitism by *Trypanosoma cruzi* on kissing bug behavior. The absence of these and other factors (see below) in our experimental set-up should convey a logical restrain to any temptation to extrapolate these results directly to natural habitats and/or other triatomine species.

Finally, our results, in terms of dispersal and density, show a relation quite different to other hematophagous insects, which tend to disperse to other food resources or die when access to the food source diminishes, like in mosquitoes^23^ or tse-tse flies.^24^

**Fig. 20:**
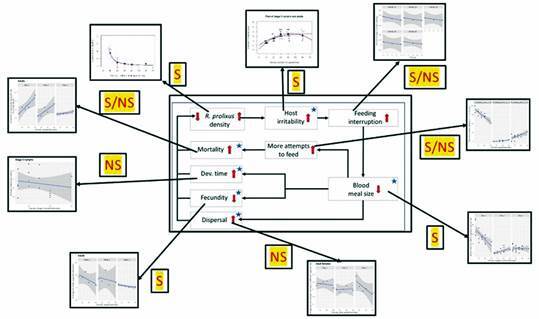
identification of the density-dependent mechanisms of the population regulation process in *Rhodnius Prolixus*. The results correspond, using as a template our hypothesis as given in Fig. 1, identifying those mechanisms that proved to have some degree of effectiveness. S: statistically significant; NS: not statistically significant; S/NS: statistical significance observed only in a given stage (or day or week). The small graphs inserted in Fig. 20 have as *x*-axis the kissing bug densities per hamster, and their *y*-axis is represented by the density-dependent variable names given in the small internal boxes (transferred from the template of our hypothesis as given in Fig. 1).

As an overall summary, Fig. 20 shows, using as a template our hypothesis as represented in Fig. 1, which of the density-dependent mechanisms of the population regulation process estimated in our laboratory experiments proved to be most effective.

We would like to end this discussion by tackling an important aspect related to a density-dependent population regulation in triatomines; it refers to the caveats associated with the overly simplified experimental design we used, which strongly limits the potential extrapolation to triatomine population regulation in terms of the time trajectories of population size, particularly in domiciliary and peri-domiciliary habitats. The first and most obvious of these reservations is that our experimental set-up is based on a small box as compared to a house or a corral. Additionally, we used only a single triatomine stage in each of the experiments (stage 5 nymphs or adults), while under domiciliary conditions usually all triatomine stages are present. There is also a remarkable difference between our experimental set-up and domiciliary conditions in terms of the number and type of hosts available: in the laboratory, only one small host with a weight of ≈135 g was available for the bugs, while houses and corrals are inhabited by several hosts (people, dogs, cats, and chickens) with a much larger biomass. Finally, we used in the laboratory a constant environment in terms of temperature and humidity, with no predators or parasites. Furthermore, indoor-resting chickens and dogs greatly modify human-bug contact rates.^25^ We were aware of these differences at the time we set up our experiment, and we selected the kissing bug densities, trying to reduce the consequences of these differences as much as possible. We considered that a density of 10 kissing bugs (stage 5 nymphs or adults) would not trigger an irritation response in a hamster, and would provide a baseline for our results. However, our results suggest that we miscalculated, and that, although weak, an irritation response might have been present at our minimum density of 10 kissing bugs/hamster.

*In conclusion* - Our results with *R. prolixus* and a hamster, couldn’t reject, in general terms, the hypothesis that there is a decrease in the average feeding status in the kissing bugs as a consequence of an irritability effect on the host at high triatomine densities; this decrease in the average feeding status leads to a decrease in life-time fecundity, but not of the stage 5 development times, or the dispersal of the kissing bugs. So, we conclude that most of the mechanisms previously postulated,^8^ ^,^ ^12^ acting through the irritability of the host, seem to be confirmed as effective in the population regulation process, at least as they emerge from our limited experimental conditions with *R. prolixus*. This conclusion may seem surprising, given the triatomine’s evolutionary-adaptive strategy of minimizing the irritability of the host (see the Discussion section). So, we here postulate, as a future research line that needs to be investigated, that the source of the host irritability triggering the density-dependent population regulation must be related to a behavioral and not a physiological response. Several indicators point to this suggestion: the perception of triatomine bug bites at increasing densities in human volunteers;^13^ the lack of almost any density-dependent response from chickens,^6^ where the host was restrained but the physiological response was not impaired; and, last but not least, the many anecdotal stories of people moving to a new rural house when the kissing bugs became excessively abundant, due to the extreme nuisance the triatomine bugs represent.

We are aware of the difficulty to generalizing this density-dependent population regulation, not only in extrapolating to natural environmental conditions but also in assuming that they may be valid in other triatomine species. There are multiple sources of variation in many traits of the triatomines that make any generalization problematic, particularly those related to body size and type and number of hosts; in *T. infestans*, the triatomines’ body-size was modified by habitat type, bug stage, and recent feeding (*e.g.*, bugs from chicken coops had a significantly larger body size than pig-corral and kitchen bugs);^26^ furthermore, these authors showed that there are significant links between body size, microsite temperatures, and various population fitness components. Despite these confounding effects, as well as the caveats of using *R’*_*o*_ as a proxy of the actual population net reproductive rate *R*_*o*_ , the strongly significant decrease in *R’*_*o*_ as a function of adult density leads us to believe that we have experimentally demonstrated, for the first time, that a density-dependent population regulation is acting through an irritability of the host, and becomes the most plausible mechanism in population regulation in *R. prolixus*, and possibly in other triatomine species.
