# Supplementary material for: Population size regulation is density-dependent in Rhodnius prolixus (Hemiptera: Reduviidae) through an irritability mechanism
Source: Mem Inst Oswaldo Cruz. 2023 Jun 2;118:e220211. doi: 10.1590/0074-02760220211 (PMC10243471; doi:10.1590/0074-02760220211)
Supplement: Supplementary file 1 [file 1678-8060-mioc-118-e220211-s.pdf]

SUPPLEMENTARY DATA I

Review of the bibliography on natural enemies of Triatominae as potential population controlling agents

From a general point of view the natural enemies of triatomines as potential population controlling agents were analyzed by Puime and Rosa[(1)] and Marti.[(2)] Many other studies have evaluated specific enemies such as predators,[(3,4)] parasites,[(5-9)] parasitoids,[(10-21)] and pathogens.[(22-27)]

Although several species have been identified as potential natural enemies, in general their numerical response[(28)] does not seem able to cope with the population growth rate of most triatomine species, particularly the relatively high population growth rate of *Rhodnius prolixus* under domiciliary conditions.

REFERENCES

1. Puime A, Rosa R. Predadores, parasitos y parasitoides de *Triatoma rubrovaria* (Blanchard, 1843) en Uruguay. Bol Soc Zool Uruguay. 1995; 9(segunda época): 48-55.

2. Marti GA. Enemigos naturales de triatominos de la Argentina. Expectativas para un control integrado. In: Storino R, editor. Chagas en el siglo XXI. La enfermedad olvidada. La Plata, Argentina: Fundación INCALP; 2006. p. 65-71.

3. Barreto M, Barreto P, d'Alessandro A. Predation on *Rhodnius prolixus* (Hemiptera: Reduviidae) by the spider *Theridion rufipes* (Araneida: Theridiidae). J Med Entomol. 1987; 24(1): 115-16.

4. Gómez-Núñez JC. *Tapinoma melanocephalum* as an inhibitor of *Rhodnius prolixus* populations. J Med Entomol. 1971; 8(6): 735-7.

5. Anderson RG. The biology of the conenose bug parasite, *Pimeliaphilus plumifer* Newell & Ryckman (Agarina: Pterygosomidae) (Hemiptera: Reduviidae). J Med Entomol. 1968; 5(4): 473-7.

6. Newell IM, Ryckman RE. *Pimeliaphilus zeledoni* n. sp. (Acari, Pterygosomidae), a parasite of *Triatoma dimidiata* (Latr.) (Hemiptera, Reduviidae). Bull So Calif Acad Sci. 1969; 68(3): 138-44.

7. Ghilini JM, Mauri RA. Aspectos poblacionales de *Pimeliaphilus triatomae* Cunliffe, 1952 (Acarina-Pterygosomidae), ectoparásito de *Triatoma infestans* Klug, 1834 (Hemiptera-Reduviidae). Rev Soc Entomol Arg. 1985; 44(3-4): 239-42.

8. Marti GA, Balsalobre A, Pazos RS, Ceccarelli S, Martínez PA. Distribución geográfica del género *Pimeliaphilus trägärdh* (Acari: Prostigmata) asociados a triatominos (Hemiptera: Reduviidae). Rev Soc Entomol Arg. 2017; 76(1-2): 41-5.

9. Eliceche DP, Achinelly MF, Silvestre C, Micieli MV, Marti GA. Entomopathogenic nematodes (Heterorhabditidae and Steinernematidae), to control *Triatoma infestans* populations (Hemiptera: Reduviidae), Chagas disease vector. Biol Control. 2022; 165: 104814. doi: 10.1016/j.biocontrol.2021.104814.

10. Lumbreras H, Arrarte J, Guevara B. Control biológico de los vectores de la enfermedad de Chagas. La presencia del *Telenomus fariai* infestando huevos de *Panstrongylus herreri* en el departamento de San Martin. Rev Med Per. 1955; 26(315): 63-73.

11. Zeledón R, Zúñiga A, Swartzwelder JC. Hallazgo de *Telenomus fariai* en San Rafael de Alajuela. Algunas observaciones sobre la ecología de este Microhimenóptero. 1st. Central American Microbiology Congress, San José, Costa Rica, December, 1965.

12. Bertram DS. Hymenopteran egg-parasite of triatomine bugs. Trans R Soc Trop Med Hyg. 1973; 67(1): 32.

13. De Santis L, Sarmiento JA, Rabinovich JE, de Piñero DF. Himenópteros parasitoides de *Rhodnius prolixus* (Hem.) en Venezuela. Rev Soc Entomol Argentina. 1975; 35(1-4): 135-42.

14. De Santis L, Coscarón MC, Loiacono MS. Contributions to the knowledge of the entomophagous insects that destroy the kissing-bugs. Rev Soc Entomol Arg. 1987; 44(2): 169-77.

15. Feliciangeli D, Rabinovich JE. Efecto de la densidad en *Ooencyrtus trinidadensis* (Chalcidoidea, Encyrtidae), un parásito endófago de los huevos de *Rhodnius prolixus*, vector de la enfermedad de Chagas en Venezuela. Rev Inst Med Trop São Paulo. 1977; 19(1): 21-34.

16. Feliciangeli MD. Experimental parasitism of Triatominae eggs (Hemiptera: Reduviidae) by the Microhymenoptera *Ooencyrtus trinidadensis venatorius*

CC BY

(Chalcidoidea: Encyrtidae) and *Telenomus costalimai* (Proctotrupoidea: Scelionidae). J Med Entomol. 1978; 15(1): 25-30.

17. Conde JE, Rabinovich JE. Larval competition between *Telenomus costalimai* (Hymenoptera: Scelionidae) and *Ooencyrtus trinidadensis venatorius* (Hymenoptera: Encyrtidae) after simultaneous oviposition in *Rhodnius prolixus* eggs (Hemiptera: Reduviidae). J Med Entomol. 1979; 16(5): 428-31.

18. Brewer MI, Arguello N, Gorla D, Rosacher C. Presencia de parasitoides oófagos de *Triatoma infestans* en el Departamento Cruz del Eje, Córdoba. Medicina (Buenos Aires). 1980; 40: 213-6.

19. De Santis L, de Regalia JA, de Silva MS, del Carmen M, de Larramendi C. Identificación de los enemigos naturales de la vinchuca. Medicina (Buenos Aires). 1980; 40(1): 197-206.

20. Fernandes AJ, Silva JC, Diotaiuti L. Parasitismo natural de ovos de triatomineos por *Telenomus fariai* Lima, 1927 no laboratório. Rev Soc Bras Med Trop. 1990; 23: 149-51.

21. Gorla DE. *Telenomus fariai* (Hymenoptera: Scelionidae) is not a good choice for the control of domestic populations of *Triatoma infestans* (Hemiptera: Reduviidae). Rev Soc Entomol Arg. 2020; 79(1): 31-3.

22. Luz C, Fargues J. Factors affecting conidial production of *Beauveria bassiana* from fungus-killed cadavers of *Rhodnius prolixus*. J Invert Pathol. 1998; 72(2): 97-103.

23. Beard CB, Cordon-Rosales C, Durvasula RV. Bacterial symbionts of the triatominae and their potential use in control of Chagas disease transmission. Ann Rev Entomol. 2002; 47(1): 123-41.

24. Vasquez P, Saldarriaga CAY, Chaverra DR. Susceptibility of fifth instar nymphs of *Rhodnius prolixus* (Hemipetra: Reduviidae) to the action of the fungus *Beauveria bassiana*. Rev Colomb Entomol. 2005; 31(1): 15-9.

25. Cazorla D, Moreno PM. Compatibilidad de 13 aislamientos de *Beauveria bassiana* patógenos para *Rhodnius prolixus* (Triatominae) con insecticidas químicos. Bol Mal Salud Amb. 2010; 50(2): 261-70.

26. Forlani L, Pedrini N, Girotti JR, Mijailovsky SJ, Cardozo RM, Gentile AG, et al. Biological control of the Chagas disease vector *Triatoma infestans* with the entomopathogenic fungus *Beauveria bassiana* combined with an aggregation cue: field, laboratory and mathematical modeling assessment. PLoS Negl Trop Dis. 2015; 9(5): e0003778.

27. Baldiviezo LV, Pedrini N, Santana M, Mannino MC, Nieva LB, Gentile A, et al. Isolation of *Beauveria bassiana* from the Chagas disease vector *Triatoma infestans* in the Gran Chaco region of Argentina: assessment of gene expression during host-pathogen interaction. J Fungi (Basel). 2020; 6(4): 219. doi: 10.3390/jof6040219.

28. Crawley MJ. The numerical responses of insect predators to changes in prey density. J Anim Ecology. 1975: 44(3): 877-92.

SUPPLEMENTARY DATA II

TABLE

Average proportional mortality values of nymphs of stages 1 to 4 of *Rhodnius prolixus*, obtained from the literature. These values were obtained at a variety of density, environmental, and feeding conditions, but always under a constant temperature regime

Temperature (ºC)	Relative humidity (%)	Food source	Freq. feed (days)	N1 Mort (prop)	N2 Mort (prop)	N3 Mort (prop)	N4 Mort (prop)	N5 Mort (prop)	Reference
28	65	Chicken	14	0.0632	0.034	0.12791	0.30667	0.1923	(1)
21	60	HMB	-	0.19	0.111	0.0972	0.0308	0.0159	(2)
27	80	Chicken	7	0	0	0	0	0	(3)
25	72.5	Mouse/rat	1	0.0667	0.143	0	0.08333	0.0909	(4)
25	72.5	Mouse/rat	18.5	0.0157	0	0	0.004	0	(4)
28	-	Other birds	10	0.01	0.011	0.05	0	0	(5)
28	-	Rabbit	10	0.01	0.016	0.017	0	0	(5)
28	-	Chicken	10	0.015	0.016	0.017	0.012	0.028	(5)
28	-	Other birds	10	0.025	0.078	0.184	0.357	0.476	(5)
28	-	Other birds	10	0.015	0.05	0.102	0.148	0.193	(5)
25	75	Mouse/rat	18	0	0	0	0	0.05	(6)
28	77.5	Chicken	7	0	0	-	-	0.044	(7)

HMB: heparinized mammal blood; N: stands for "Nymph"; the sign "-" indicates no data available.

REFERENCES

1. Gómez-Núñez J. Mass rearing of *Rhodnius prolixus*. Bull World Health Org. 1964; 31: 565-7.

2. Gómez I. Nuevas observaciones acerca de la acción patógena del *Trypanosoma rangeli* Tejera, 1920 sobre *Rhodnius prolixus* Stal. Rev Inst Med Trop São Paulo. 1967; 9(1): 5-10.

3. Rodríguez D, Rabinovich JE. The effect of density on some population parameters of *Rhodnius prolixus* (Hemiptera: Reduviidae) under laboratory conditions. J Med Entomol. 1980; 17(2): 165-71.

4. Lent H, Valderrama A. Observações em laboratório, sobre o ciclo evolutivo de *Rhodnius prolixus* Stal, 1859, *R. pictipes* Stal, 1872 e *R. neivai* Lent, 1953. Rev Bras Biol. 1977; 37(2): 325-44.

5. Gomes JEPL, Azambuja P, Garcia ES. Comparative studies on the growth and reproductive performances of *Rhodnius prolixus* reared on different blood sources. Mem Inst Oswaldo Cruz. 1990; 85(3): 299-304.

6. Añez N. Studies on *Trypanosoma rangeli* Tejera, 1920. VII - Its effect on the survival of infected Triatomine bugs. Mem Inst Oswaldo Cruz. 1984; 79(2): 249-55.

7. Arévalo A, Carranza JC, Guhl F, Clavijo JA, Vallejo GA. Comparación del ciclo de vida de *Rhodnius colombiensis* Moreno, Jurberg & Galvão, 1999 y *Rhodnius prolixus* Stal, 1872 (Hemiptera, Reduviidae, Triatominae) en condiciones de laboratorio. Biomédica. 2007; 27(Supl. 1): 119-29.

SUPPLEMENTARY DATA III

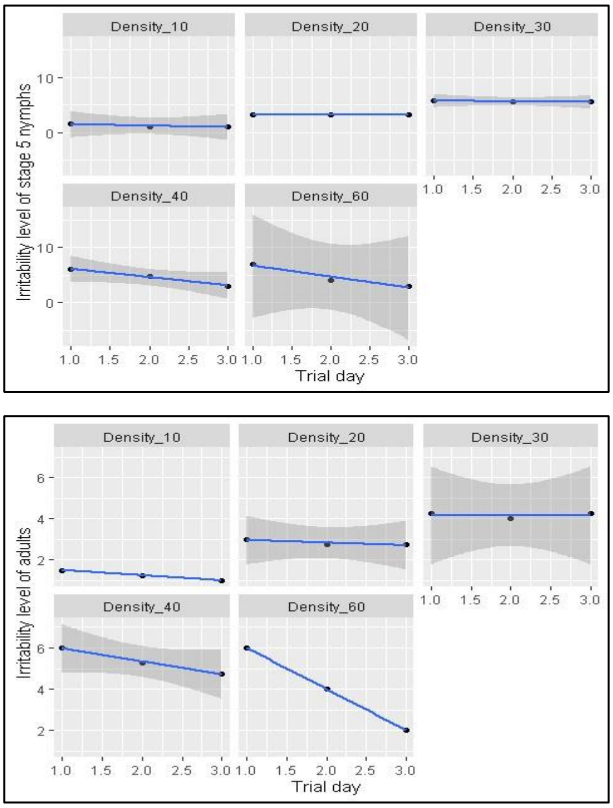

Fig. 1: results of linear regressions of the irritability score as dependent variable and each day of the three-days trials as independent variable. The regressions are given for each *Rhodnius prolixus* density, and separately for stage 5 nymphs and adults. Linear regressions applied to each experimental day separately showed a significant effect of density on irritability for day 1, but no significant effects for days 2 or 3. In the adults the effects of the experimental day on the irritability level of the hamster is seen for all three experimental days, but showed to be significant only at densities 10 and 60.

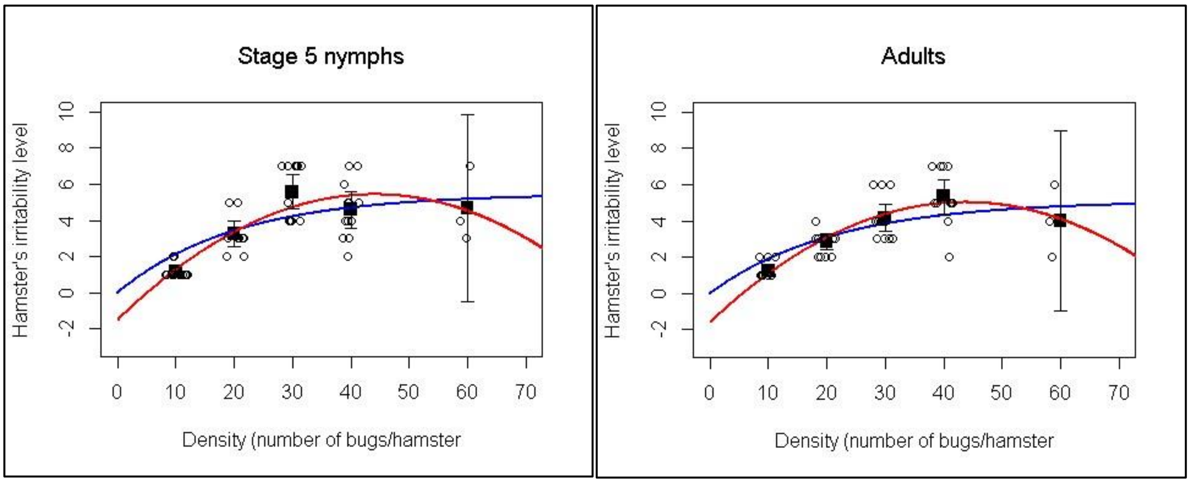

Fig. 2: fit of the irritability scores of *Rhodnius prolixus* as a function of stage 5 nymph and adult density/hamster to two non-linear models: natural growth model (solid blue line) and second-degree polynomial model (solid red line). White circles are the original experimental values for all days and replicates (slightly displaced horizontally to avoid overlap). Black squares are the experimental values averaged over all days and replicates, and their vertical bars are the 95% confidence intervals. For the natural growth model: *Irr* = 5.0903 * (1-exp(-0.0466 **Dens*); for the second-degree polynomial function: *Irr* = -0.0035279 + 0.3093827 **$Dens^2$* - 1.5601374 **Dens*. For the second-degree polynomial fit *p*= 0.0197 for parameter *a*, *p*= 0.0136 for parameter *b*, and p = 0.1068 for parameter *c*, with AICc = 7.74; for the natural growth model fit p = 0.0196 for parameter *a*, p = 0.1617 for parameter *b*, with AICc = -4.85; the AICc values suggest that the natural growth should be preferred.

CC BY

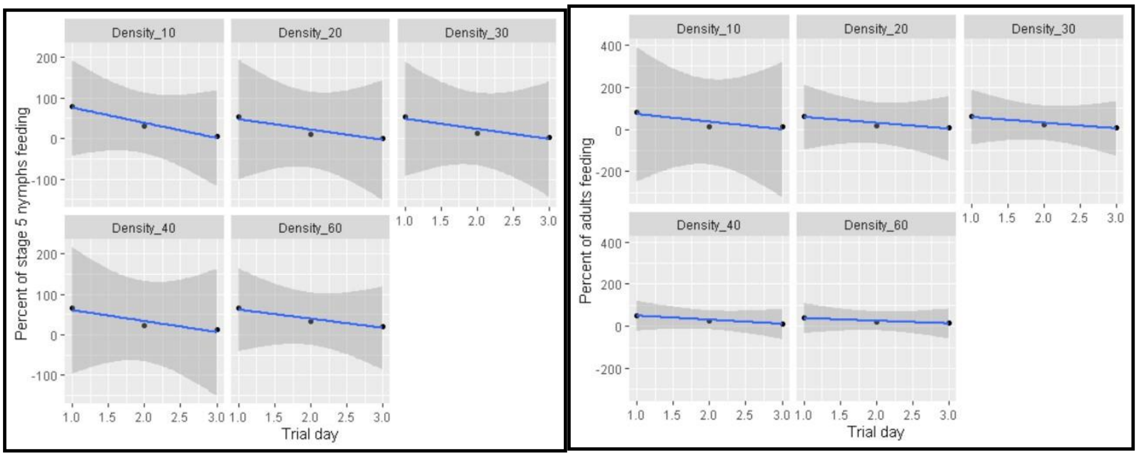

Fig. 3: percent of *Rhodnius prolixus* bugs feeding on day one, two, and three in the three-days experimental trials (x-axes), for stage 5 nymphs (left) and adults (right) as a function of bug density (bugs/hamster, identified in the top row). The effect of the experimental trial day (p = 0.00098) and the intercept p = 0.000139 were statistically significant, but not the effect of density, nor the interaction between density and the experimental day. The significance of the effect of the experimental trial day became even stronger when all densities were pooled (p = 4.35e-06).

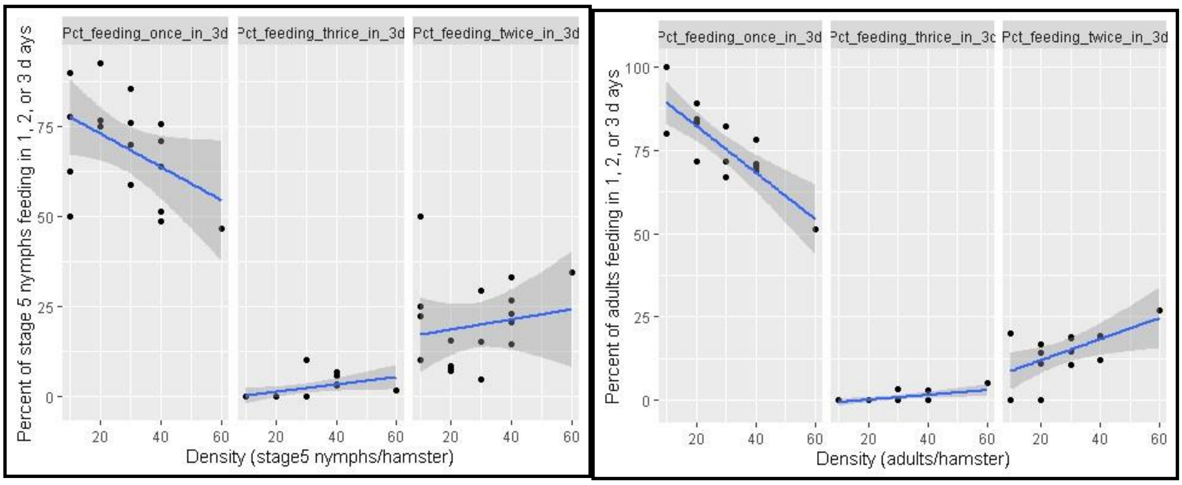

Fig. 4: percent of bugs feeding once, twice or thrice (top label) during the three-days experimental trials as a function of bug density. The slopes increase slightly with density for the bugs that “need” a second and a third meal. The overall regression of the effect of density on percent of bugs that fed once, twice or thrice for stage 5 nymphs (left panel), was not statistically significant (p = 0. 940) nor for adults (p = 0.855, right panel). However, the separate linear regressions of the percent of adult bugs that fed once, twice and thrice as a function of density were statistically significant (p = 0.000136, 0.01787, and 0.007987, for bugs that fed once, twice and thrice, respectively). For the stage 5 nymphs, only bugs that fed thrice showed a statistically significant dependence on density (p = 0.0554, 0.5256, and 0.03961, for stage 5 nymphs fed once, twice and thrice, respectively).

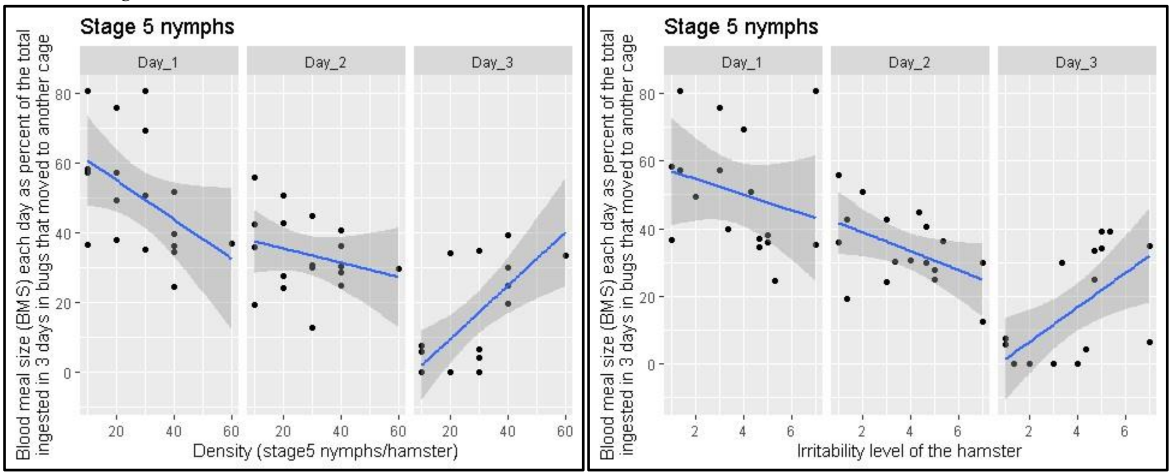

Fig. 5: average blood meal size (mg) per day as a function of bug density (left panel) and of irritability (right panel) of stage 5 nymphs that moved between boxes.

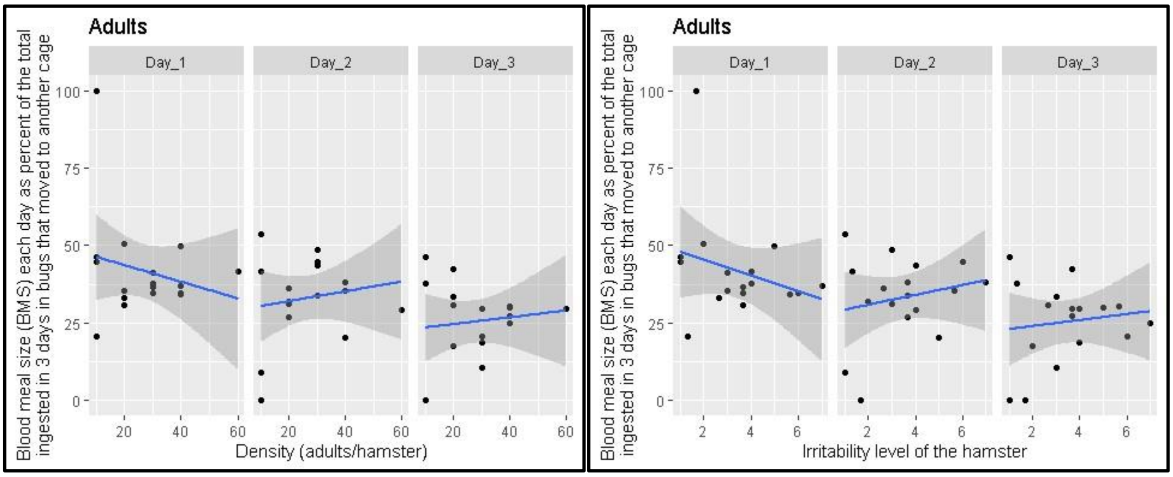

Fig. 6: same as Fig. 5 but for adult bugs that moved between boxes.

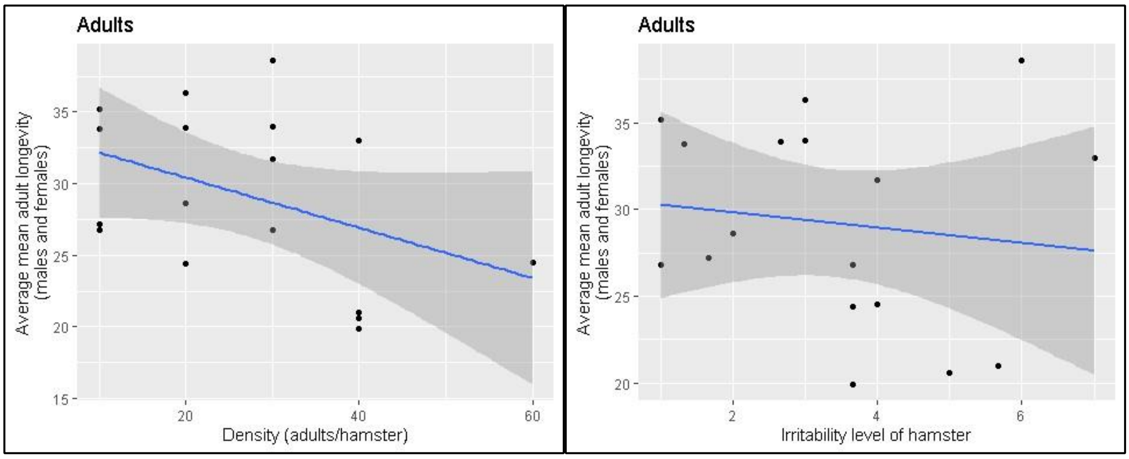

Fig. 7: average longevity of adults as a function of density and irritability. The average longevity (days) of adults as a function of density (left panel), and of irritability level of the hamster (right panel) are shown for the three-days period of the experimental trials. Neither the effects of density nor of irritability on adult survival was significant (p = 0.09392, and p = 0.612, respectively).

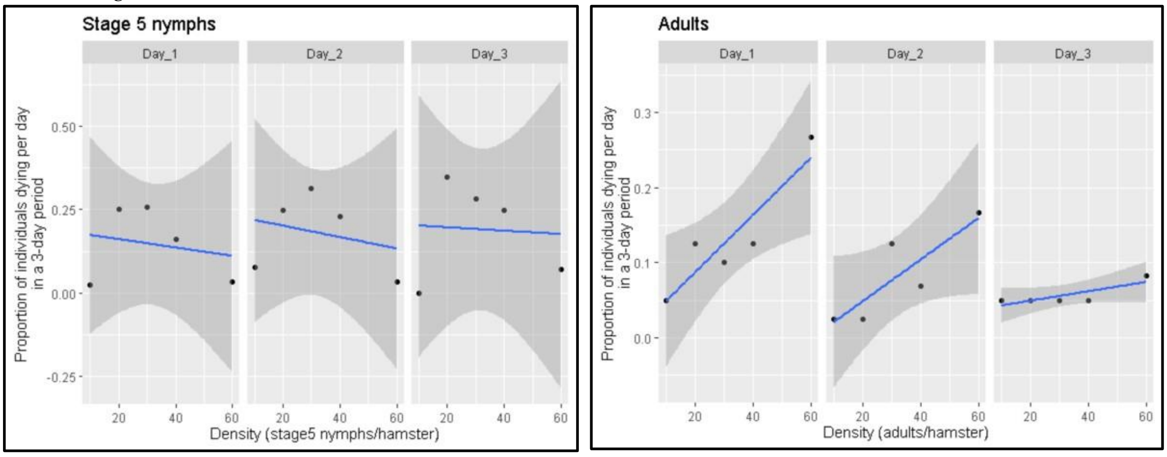

Fig. 8: average daily mortality of stage 5 nymphs and adults in a three-days period as a function of density. Only the regression for adults at day 2 was significant (p = 0.0214).

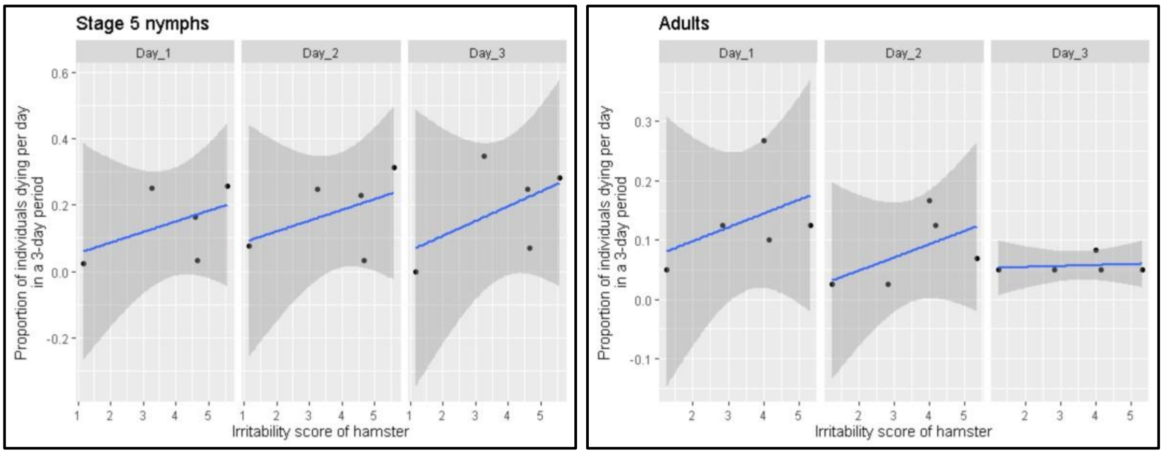

Fig. 9: average daily mortality of stage 5 nymphs and adults in a three-days period as a function of the irritability score of the hamster. Only the linear regression for stage 5 nymphs at day 1 was statistically significant (p = 0.0476).

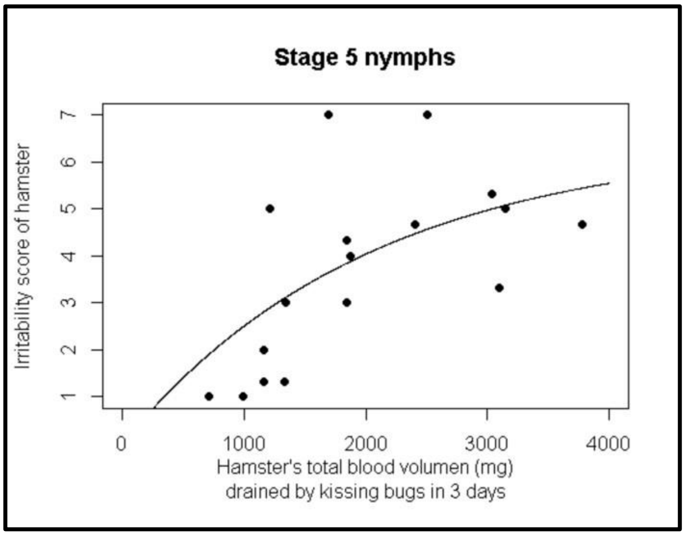

Fig. 10: average irritability score of hamsters as a function of total blood volume drained (mg) from the hamster by stage 5 nymphs, in the three-days experimental period. The black dots are the laboratory values for each density and each replicate. The black line is the fit to a monomolecular (also called natural growth) model of the form: $y = a*(1-\exp(-b*x))$; the value of the parameter a (asymptotic irritability score for very high blood drainage) was 6.45 and was statistically significant (p = 0.0219); the value of the parameter b (rate of increase of the irritability score with blood drainage) was 0.00049 and was not statistically significant (p = 0.1770).

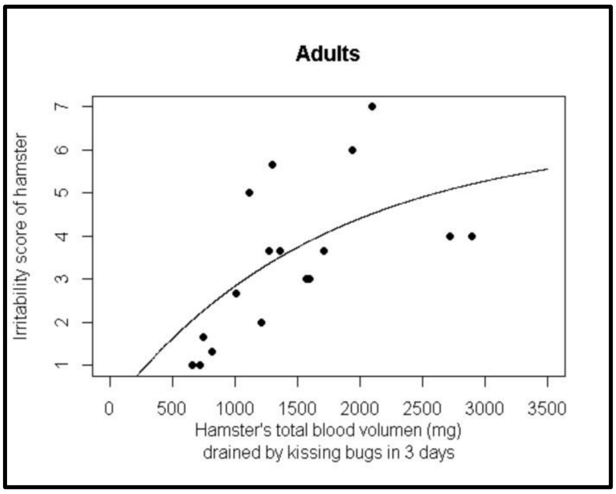

Fig. 11: irritability score of hamsters as a function of total blood volume drained (mg) from the hamster by adult *Rhodnius prolixus*, in the three-days experimental period. The black dots are the laboratory values for each density and each replicate. The black line is the fit to a monomolecular (also called natural growth) model of the form: $y = a*(1\text{-}\exp(-b*x))$; the value of the parameter *a* (asymptotic irritability score for very high blood drainage) was 6.352 and was statistically significant ($p = 0.0287$); the value of the parameter *b* (rate of increase of the irritability score with blood drainage) was 0.00059 and was not statistically significant ($p = 0.1748$).

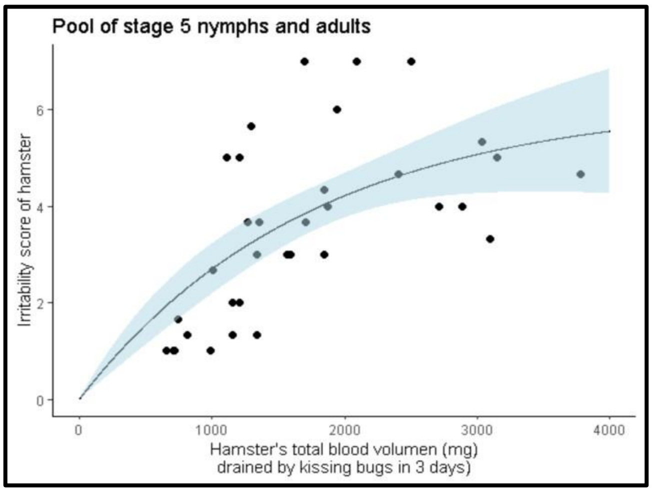

Fig. 12: irritability score of hamsters as a function of total blood volume drained (mg) from the hamster by a pooling the results of stage 5 nymphs and adults, in the three-days experimental period. The black dots are the laboratory values for each density and each replicate. The black line is the fit to a monomolecular (also called natural growth) model: $y = a*(1\text{-}\exp(-b*x))$; the value of the parameter *a* (asymptotic irritability score for very high blood drainage) was 6.173 and was statistically significant ($p = 0.000251$); the value of the parameter *b* (rate of increase of the irritability score with blood drainage) was 0.00058 and was also statistically significant ($p = 0.0308$). The light blue area is the 90% confidence interval of the values predicted with the natural growth (monomolecular) model.

TABLE

Probability values (p) of the linear regressions for each stage, each day, and each independent variable.

The p values highlighted in red are statistically significant ($p \leq 0.05$)

Independent variable	Day	Stage	
		Stage 5 nymphs	Adults
Density	1	0.5672	0.0856
	2	0.5558	0.0214
	3	0.3706	0.7464
Irritability	1	0.0476	0.1356
	2	0.0729	0.0685
	3	0.3353	0.7498

SUPPLEMENTARY DATA IV

Results of beta regressions for various response variables expressed as proportions

(1) Proportion of stage 5 nymphs that molted into adults

TABLE I

Results of the beta regressions for the proportion of stage 5 nymphs that molted into adults, during the three-weeks period after the three-days trials

Case	Intcpt	Density	Phi	LL	Ps_R2	p-value
N5 Molt	-0.5254	-0.01724	8.8995	3.143382	0.065156	0.57752

N5: stage 5 nymphs; Intcpt: coefficient of the intercept; Density: coefficient of the kissing bug density effect; Phi: precision; LL: log-likelihood; Ps_R2: pseudo R2; p-value: 95% probability value of the density effect. The number of degrees of freedom was 3.

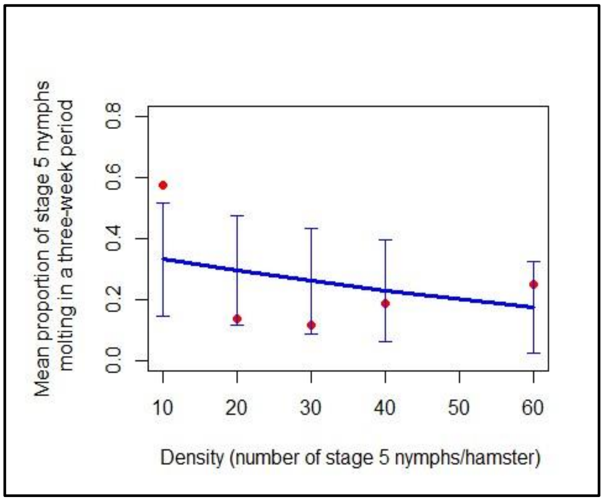

Fig. 1: response of stage 5 nymphs that molted into adults during a three-weeks period after the three-days trials. Red dots: mean observed values; blue line: beta model. The 95% confidence intervals correspond to the beta regression model (not to the observed values).

(2) Proportion of adults that moved to another box

TABLE II

Results of the beta regressions of the proportion of adults that moved to another box, during each of the three-days trials

Case	Intcpt	Density	Phi	LL	Ps_R^2	p-value-Dens
Fem/d1	-1.4558	-0.0046	149.1437	10.3988	0.1432	0.4217
Fem/d2	-2.2878	0.0025	114.3021	10.9228	0.0238	0.7498
Fem/d3	-0.8254	-0.0387	100.4235	10.2873	0.7912	0.00003
Fem/3d	-5.0364	-0.0317	1421.2052	26.8201	0.5902	0.02801
Male/d1	-1.0317	-0.0154	46.8147	7.5263	0.3824	0.1256
Male/d2	-2.1633	-0.0003	83.2526	10.1076	0.0003	0.9709
Male/d3	-1.4798	-0.0178	72.2489	9.4880	0.2909	0.0715
Male/3d	-5.2425	-0.0274	1365.6143	26.8586	0.4207	0.0660

Fem: adult females; Male: adult males; Intcpt: coefficient of the intercept; Density: coefficient of the kissing bug density effect; Phi: precision; LL: loglikelihood; Ps_R^2: pseudo R^2; p-vale: 95% probability value of the density effect. The number of degrees of freedom was 3. The cases for which the effect of the kissing bug density effect was statistically significant, are shown in red font.

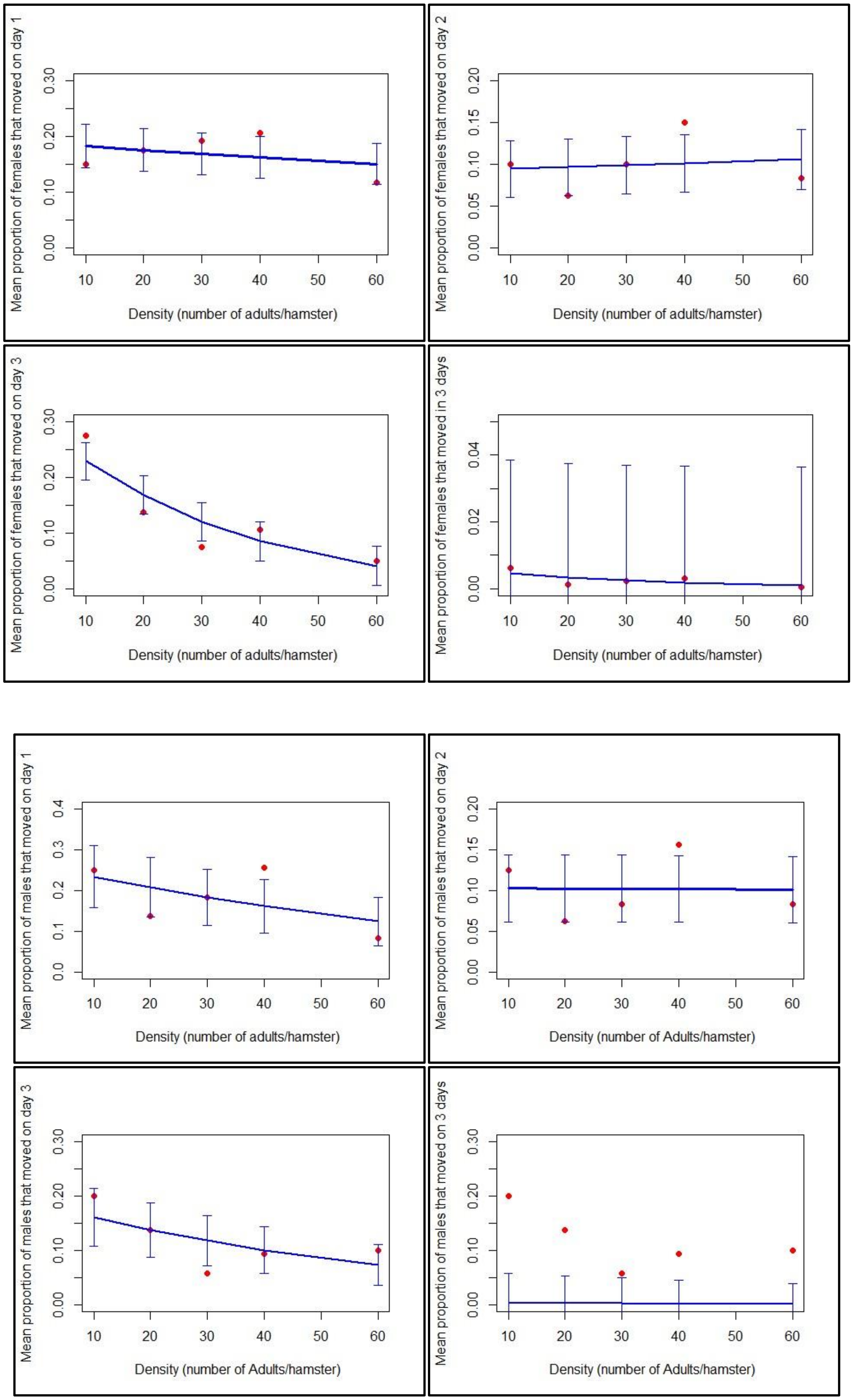

Fig. 2: response of females and males that moved from one box to another box in a three-days period during the three-days trials. Red dots: mean observed values; blue line: beta model. The 95% confidence intervals correspond to the beta regression model (not to the observed values).

(3) Proportion of stage 5 nymphs and adults that fed

TABLE III

Results of the beta regressions of the proportion of stage 5 nymphs (N5) and adults (AD) that fed during each of the three-days trials

Case	Intcpt	Density	Phi	LL	Ps_R^2	p-value
N5/Day1	0.727567	-0.0061	22.86516	4.516556	0.065156	0.57752
N5/Day2	-1.67956	0.011973	27.06235	5.828562	0.120707	0.300829
N5/Day3	-4.24014	0.048512	67.34106	10.9269	0.590821	2.80E-05
AD/Day1	1.504198	-0.03374	96.90088	8.17509	0.873344	2.85E-09
AD/Day2	-1.72772	0.009274	145.1985	10.06324	0.418011	0.082811
AD/Day3	-2.61012	0.014249	252.9395	12.73143	0.527748	0.005678

Intcpt: coefficient of the intercept; Density: coefficient of the kissing bug density effect; Phi: precision; LL: log-likelihood; Ps_R^2: pseudo R^2; p-value: 95% probability value of the density effect. The number of degrees of freedom was 3. The cases for which the effect of the kissing bug density effect was statistically significant, are shown in red font.

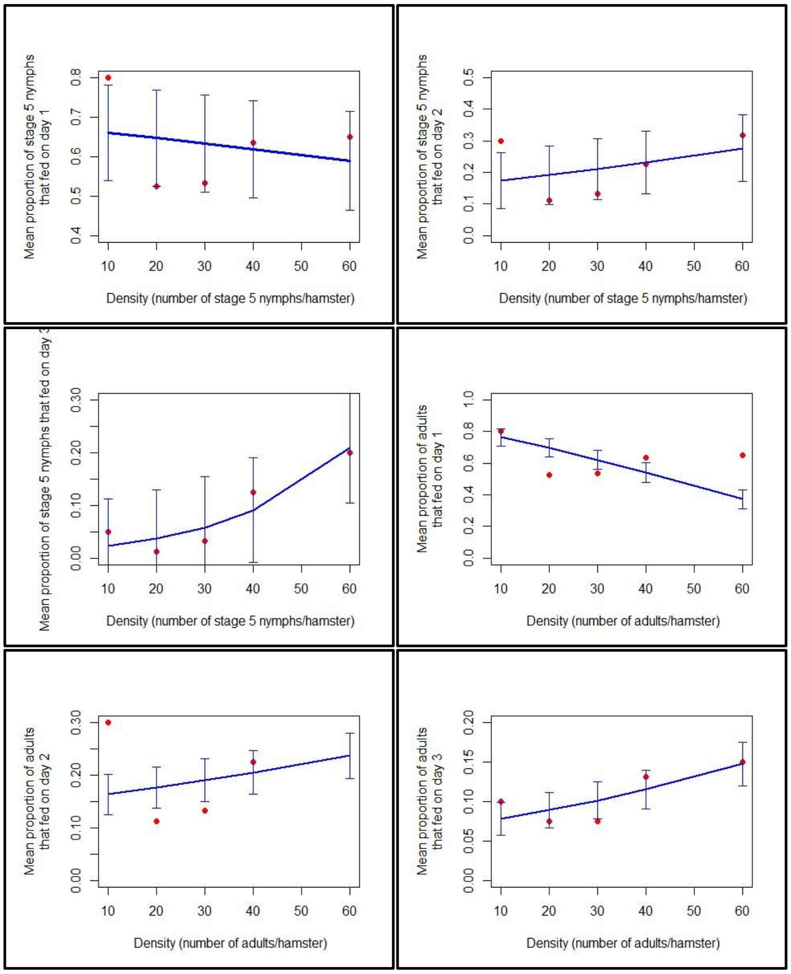

Fig. 3: response of stage 5 nymphs and adults that fed on each day in the three-days trials. Red dots: mean observed values; blue line: beta model. The 95% confidence intervals correspond to the beta regression model (not to the observed values).

(4) Proportion of stage 5 nymphs and adults that died

TABLE IV

Results of the beta regressions of the proportion of stage 5 nymphs (N5) and adults (AD) that died during each of the three-days trials

Case	Intcpt	Density	Phi	LL	Ps_R^2	p-value
N5/Day1	-1.6781	-0.0045	9.2629	5.0986	0.0213	0.8299
N5/Day2	-1.1441	-0.0133	11.5446	4.7689	0.1871	0.4730
N5/Day3	-2.2172	0.0187	4.0613	4.7221	0.1569	0.4577
N5/3Days	-0.3055	-0.0056	3.9155	0.9004	0.0169	0.8108
AD/Day1	-3.0304	0.0326	181.0328	11.6563	0.8363	8.3E-09
AD/Day2	-3.7327	0.0361	81.7739	11.1901	0.7263	0.0004
AD/Day3	-3.1810	0.0110	1083.8782	17.7546	0.6388	0.0009
AD/3Days	-2.2477	0.0325	154.0760	10.0153	0.8941	5.4E-11

Intcpt: coefficient of the intercept; Density: coefficient of the kissing bug density effect; Phi: precision; LL: log-likelihood; Ps_R^2: pseudo R^2; p-value: 95% probability value of the Density effect. The number of degrees of freedom was 3. The cases for which the effect of the kissing bug density effect was statistically significant, are shown in red font.

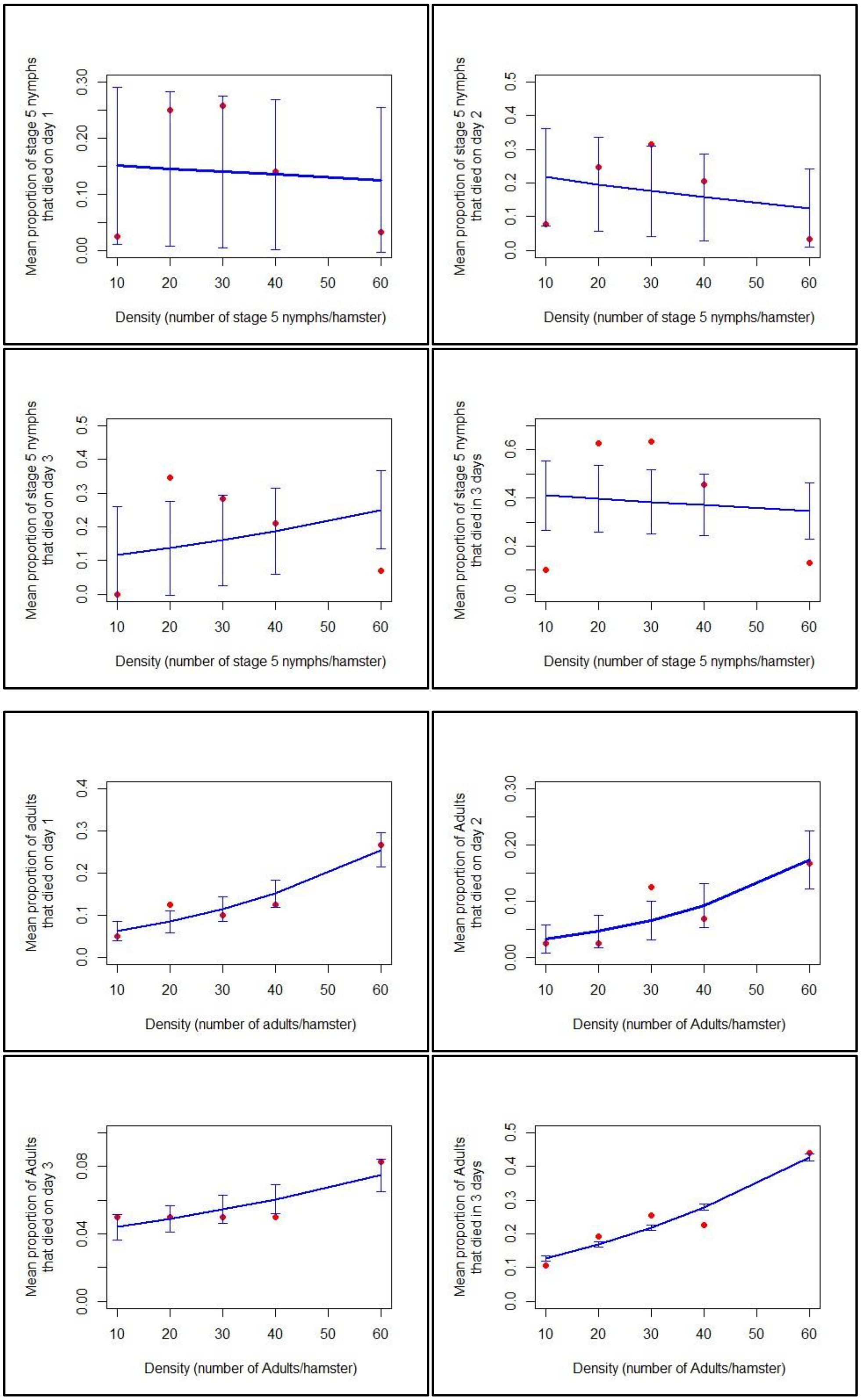

Fig. 4: response of stage 5 nymphs and adults that died on each day in the three-days trials. Red dots: mean observed values; blue line: beta model. The 95% confidence intervals correspond to the beta regression model (not to the observed values).

(5) Proportion of adult females that died

TABLE V

Results of the beta regressions of the proportion of adult females that died during each of the three-weeks after the three-days trials

Case	Intcpt	Density	Phi	LL	Ps_R^2	p-value
Fem/Week1	-0.03747	-0.01897	285.1804	10.82835	0.874902	1.90E-08
Fem/Week 2	-0.16613	-0.02174	340.282	11.4686	0.902402	2.64E-11
Fem/Week 3	-0.53522	-0.0221	715.127	13.76091	0.936519	6.16E-19
Fem/3Weeks	-2.00334	-0.05373	1495.203	20.49089	0.957594	1.15E-24

Intcpt: coefficient of the intercept; Density: coefficient of the kissing bug density effect; Phi: precision; LL: log-likelihood; Ps_R^2: pseudo R^2; p-value: 95% probability value of the Density effect. The number of degrees of freedom was 3. The cases for which the effect of the kissing bug density effect was statistically significant, are shown in red font.

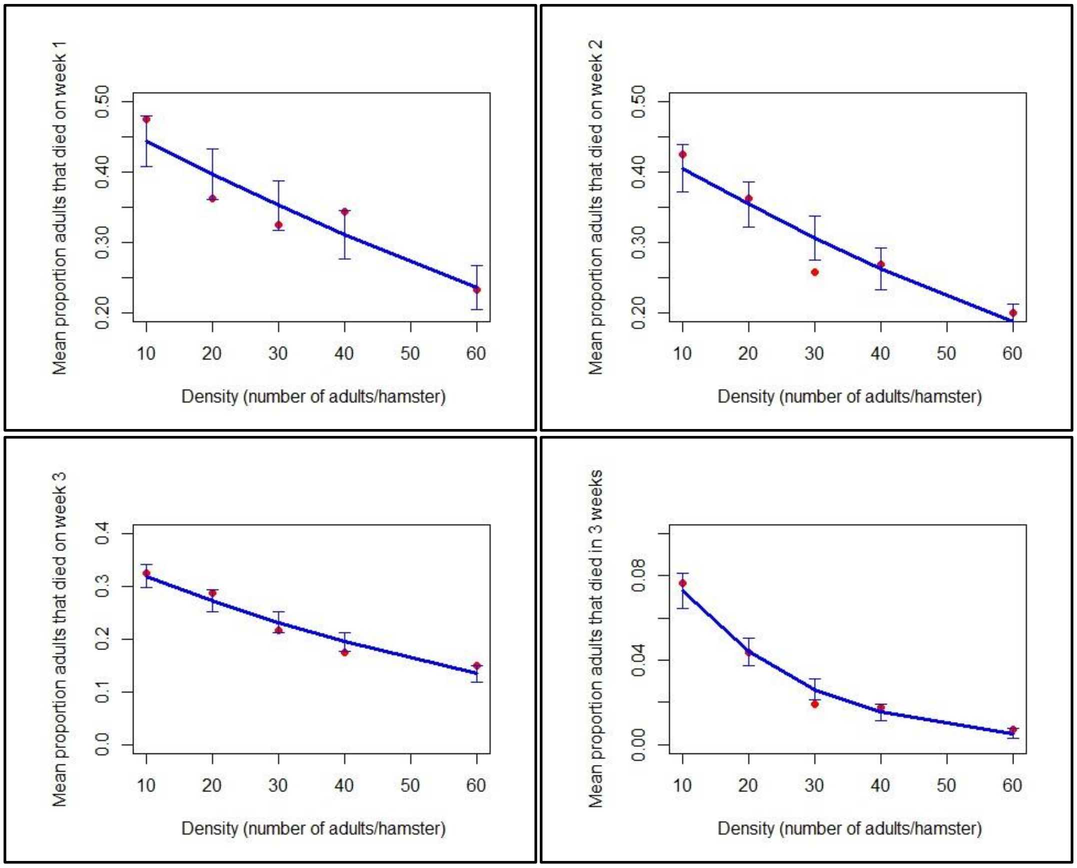

Fig. 5: response of the adults that died in each week of the three-week period after the three-days trials. Red dots: mean observed values; blue line: beta model. The 95% confidence intervals correspond to the beta regression model (not to the observed values).

(6) Proportion of adults and stage 5 nymphs that fed once or twice

TABLE VI

Results of the beta regressions of the proportion of adults and stage 5 nymphs that fed once or twice during each of the three-days trials

Case	Intcpt	Density	Phi	LL	Ps_R^2	p-value
N5/once	1.5618	-0.0264	67.2785	7.3510	0.7043	0.0001
N5/twice	-1.9421	0.0192	28.3570	5.9984	0.2551	0.0930
Ad/once	2.3352	-0.0383	221.5932	10.8802	0.9441	1.69E-20
Ad/twice	-2.5194	0.0253	1522.2469	16.1714	0.9685	6.44E-45

N5: stage 5 nymphs; Ad: adults; Intcpt: coefficient of the intercept; Density: coefficient of the kissing bug density effect; Phi: precision; LL: loglikelihood; Ps_R^2: pseudo R^2; p-value: 95% probability value of the Density effect. Degrees of freedom = 3. Cases with statistically significant density effect are shown in red font. NOTE: The proportion of adult females that fed thrice during each of the three-days trials was not fitted to a beta regression because there were some zero values.

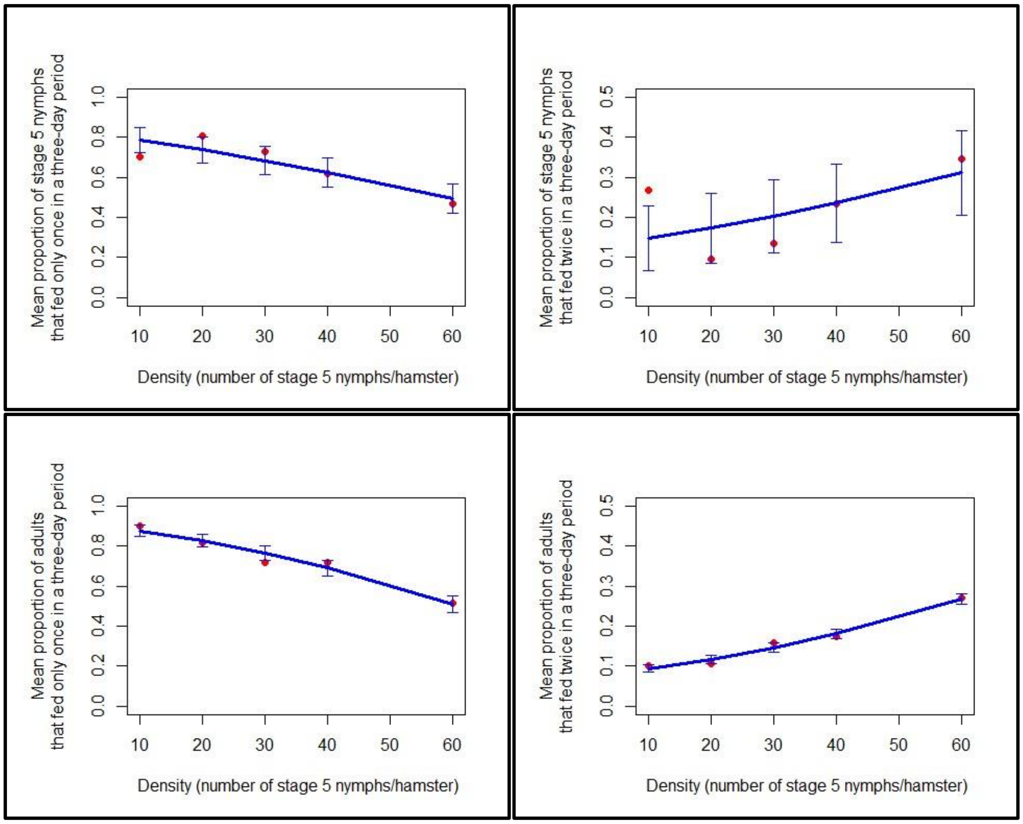

Fig. 6: response of the stage 5 nymphs that fed once or twice in the three-days trials. Red dots: mean observed values; blue line: beta model. The 95% confidence intervals correspond to the beta regression model (not to the observed values).

(7) Proportion of stage 5 nymphs and adults that moved (fed or not fed)

TABLE VII

Results of the beta regressions of the proportion of stage 5 nymphs and adults that moved (fed or not fed) during the three-days trials

Case	Intcpt	Density	Phi	LL	Ps_R^2	p-value-Dens
Ad/d1	-3.2375	0.0189	108.0801	11.8604	0.5808	0.0424
Ad/d2	-4.1288	0.0289	173.3166	14.4333	0.7641	0.0018
Ad/d3	-3.6555	0.0151	638.5478	17.1846	0.5842	0.0024
Ad/3d	-2.4926	0.0232	81.6648	9.3164	0.7296	0.0026
N5/d1	-3.1750	0.0194	170.4104	12.7205	0.5937	0.0074
N5/d2	-3.8662	0.0332	1732.4507	18.8566	0.9685	0.0000
N5/d3	-3.5225	0.0118	548.5781	16.8388	0.5454	0.0278
N/3d	-2.3801	0.0257	336.8494	12.4335	0.8966	0.0000

N5: stage 5 nymphs; 3d: during the (cumulative) three days of the trial; Intcpt: coefficient of the intercept; Density: coefficient of the kissing bug density effect; Phi: precision; LL: log-likelihood; Ps_R^2: pseudo R^2; p-value: 95% probability value of the density effect. Number of degrees of freedom = 3. The cases for which the effect of the kissing bug density effect was statistically significant, are shown in red font.

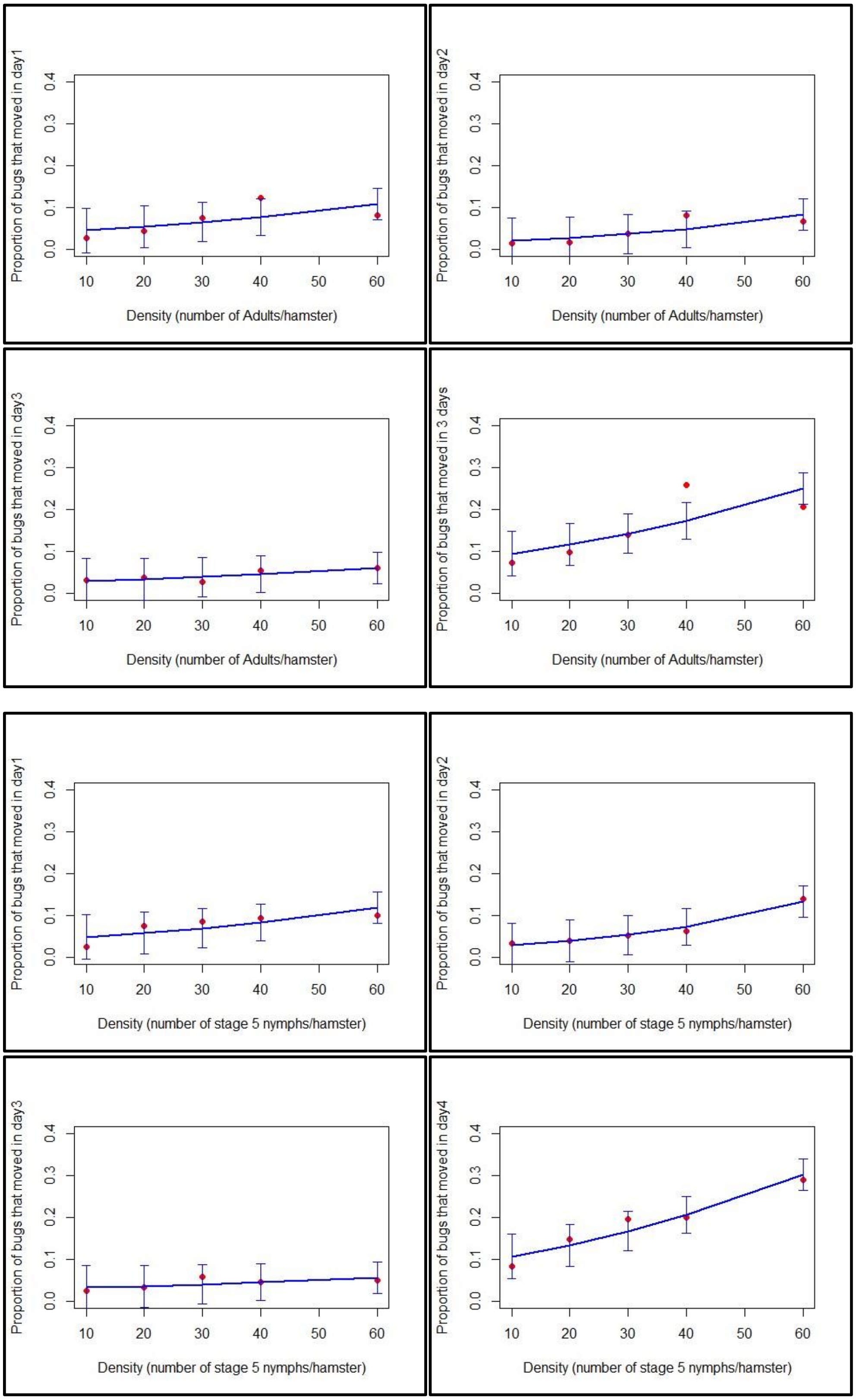

Fig. 7: response of the proportion stage 5 nymphs (upper four graphs) and adults (lower four graphs) that moved (fed or not) into another box during the three-days trials. Red dots: mean observed values; blue line: beta model. The 95% confidence intervals correspond to the beta regression model (not to the observed values).

SUPPLEMENTARY DATA V

The following table provides the original data as recorded from the experimental set-up, and some calculated response variables. The original table of the recorded data was divided into 25 parts in order to allow fitting in the width of one page; each part carries the same first four columns to identify each experimental combination.

TABLE

Data set with the original experimental results

(Part 1)

Dens	Replic	Stage	Sex	Irritability	N_dead_D1	N_dead_D2	N_dead_D3	N_dead_3Days
10	1	5	NA	1	1	1	0	2
10	2	5	NA	1	0	0	0	0
10	3	5	NA	1.3	0	0	0	0
10	4	5	NA	1.3	0	2	0	2
20	1	5	NA	2	5	3	4	12
20	2	5	NA	3	4	6	4	14
20	3	5	NA	3	7	3	5	15
20	4	5	NA	5	4	3	2	9
30	1	5	NA	7	8	8	2	18
30	2	5	NA	4	7	10	6	23
30	3	5	NA	7	6	5	5	16
30	4	5	NA	4.3	10	5	4	19
40	1	5	NA	4.7	5	12	7	24
40	2	5	NA	5	6	8	5	19
40	3	5	NA	3.3	5	6	11	22
40	4	5	NA	5.3	10	5	3	18
40	5	5	NA	NA	2	4	2	8
60	1	5	NA	4.7	2	2	4	8
10	1	6	NA	1	0	0	0	0
10	2	6	NA	1	0	0	0	0
10	3	6	NA	1.3	0	0	0	0
10	4	6	NA	1.7	2	1	2	5
20	1	6	NA	3	2	0	3	5
20	2	6	NA	2	6	0	0	6
20	3	6	NA	2.7	1	0	0	1
20	4	6	NA	3.7	1	2	1	4

CC BY

30	1	6	NA	3	2	2	1	5
30	2	6	NA	3.7	1	5	3	9
30	3	6	NA	6	8	2	1	11
30	4	6	NA	4	1	6	1	8
40	1	6	NA	5	6	3	3	12
40	2	6	NA	3.7	3	2	1	6
40	3	6	NA	5.7	5	2	3	10
40	4	6	NA	7	6	4	1	11
60	1	6	NA	4	16	10	5	31
10	1	6	1	NA	NA	NA	NA	NA
10	2	6	1	NA	NA	NA	NA	NA
10	3	6	1	NA	NA	NA	NA	NA
10	4	6	1	NA	NA	NA	NA	NA
20	1	6	1	NA	NA	NA	NA	NA
20	2	6	1	NA	NA	NA	NA	NA
20	3	6	1	NA	NA	NA	NA	NA
20	4	6	1	NA	NA	NA	NA	NA
30	1	6	1	NA	NA	NA	NA	NA
30	2	6	1	NA	NA	NA	NA	NA
30	3	6	1	NA	NA	NA	NA	NA
30	4	6	1	NA	NA	NA	NA	NA
40	1	6	1	NA	NA	NA	NA	NA
40	2	6	1	NA	NA	NA	NA	NA
40	3	6	1	NA	NA	NA	NA	NA
40	4	6	1	NA	NA	NA	NA	NA
60	1	6	1	NA	NA	NA	NA	NA
10	1	6	2	NA	NA	NA	NA	NA
10	2	6	2	NA	NA	NA	NA	NA
10	3	6	2	NA	NA	NA	NA	NA
10	4	6	2	NA	NA	NA	NA	NA
20	1	6	2	NA	NA	NA	NA	NA
20	2	6	2	NA	NA	NA	NA	NA
20	3	6	2	NA	NA	NA	NA	NA
20	4	6	2	NA	NA	NA	NA	NA
30	1	6	2	NA	NA	NA	NA	NA

30	2	6	2	NA	NA	NA	NA	NA
30	3	6	2	NA	NA	NA	NA	NA
30	4	6	2	NA	NA	NA	NA	NA
40	1	6	2	NA	NA	NA	NA	NA
40	2	6	2	NA	NA	NA	NA	NA
40	3	6	2	NA	NA	NA	NA	NA
40	4	6	2	NA	NA	NA	NA	NA
60	1	6	2	NA	NA	NA	NA	NA
10	1	6	NA	1	NA	NA	NA	NA
10	2	6	NA	1	NA	NA	NA	NA
10	3	6	NA	1.3	NA	NA	NA	NA
10	4	6	NA	1.7	NA	NA	NA	NA
20	1	6	NA	3	NA	NA	NA	NA
20	2	6	NA	2	NA	NA	NA	NA
20	3	6	NA	2.7	NA	NA	NA	NA
20	4	6	NA	3.7	NA	NA	NA	NA
30	1	6	NA	3	NA	NA	NA	NA
30	2	6	NA	3.7	NA	NA	NA	NA
30	3	6	NA	6	NA	NA	NA	NA
30	4	6	NA	4	NA	NA	NA	NA
40	1	6	NA	5	NA	NA	NA	NA
40	2	6	NA	3.7	NA	NA	NA	NA
40	3	6	NA	5.7	NA	NA	NA	NA
40	4	6	NA	7	NA	NA	NA	NA
60	1	6	NA	4	NA	NA	NA	NA
10	1	5	NA	1	NA	NA	NA	NA
10	1	5	NA	NA	NA	NA	NA	NA
10	2	5	NA	1	NA	NA	NA	NA
10	3	5	NA	1.3	NA	NA	NA	NA
10	4	5	NA	1.3	NA	NA	NA	NA
20	1	5	NA	2	NA	NA	NA	NA
20	2	5	NA	3	NA	NA	NA	NA
20	3	5	NA	3	NA	NA	NA	NA
20	4	5	NA	5	NA	NA	NA	NA
30	1	5	NA	7	NA	NA	NA	NA

30	2	5	NA	4	NA	NA	NA	NA
30	3	5	NA	7	NA	NA	NA	NA
30	4	5	NA	4.3	NA	NA	NA	NA
40	1	5	NA	4.7	NA	NA	NA	NA
40	2	5	NA	5	NA	NA	NA	NA
40	3	5	NA	3.3	NA	NA	NA	NA
40	4	5	NA	5.3	NA	NA	NA	NA
60	1	5	NA	4.7	NA	NA	NA	NA
10	1	6	NA	1	NA	NA	NA	NA
10	2	6	NA	1	NA	NA	NA	NA
10	3	6	NA	1.3	NA	NA	NA	NA
10	4	6	NA	1.7	NA	NA	NA	NA
20	1	6	NA	3	NA	NA	NA	NA
20	2	6	NA	2	NA	NA	NA	NA
20	3	6	NA	2.7	NA	NA	NA	NA
20	4	6	NA	3.7	NA	NA	NA	NA
30	1	6	NA	3	NA	NA	NA	NA
30	2	6	NA	3.7	NA	NA	NA	NA
30	3	6	NA	6	NA	NA	NA	NA
30	4	6	NA	4	NA	NA	NA	NA
40	1	6	NA	5	NA	NA	NA	NA
40	2	6	NA	3.7	NA	NA	NA	NA
40	3	6	NA	5.7	NA	NA	NA	NA
40	4	6	NA	7	NA	NA	NA	NA
60	1	6	NA	4	NA	NA	NA	NA
10	1	5	NA	1	NA	NA	NA	NA
10	2	5	NA	1	NA	NA	NA	NA
10	3	5	NA	1.3	NA	NA	NA	NA
10	4	5	NA	1.3	NA	NA	NA	NA
20	1	5	NA	2	NA	NA	NA	NA
20	2	5	NA	3	NA	NA	NA	NA
20	3	5	NA	3	NA	NA	NA	NA
20	4	5	NA	5	NA	NA	NA	NA
30	1	5	NA	7	NA	NA	NA	NA
30	2	5	NA	4	NA	NA	NA	NA

30	3	5	NA	7	NA	NA	NA	NA
30	4	5	NA	4.3	NA	NA	NA	NA
40	1	5	NA	4.7	NA	NA	NA	NA
40	2	5	NA	5	NA	NA	NA	NA
40	3	5	NA	3.3	NA	NA	NA	NA
40	4	5	NA	5.3	NA	NA	NA	NA
40	5	5	NA	NA	NA	NA	NA	NA
60	1	5	NA	4.7	NA	NA	NA	NA
10	1	6	1	1	NA	NA	NA	NA
10	2	6	1	1	NA	NA	NA	NA
10	3	6	1	1.3	NA	NA	NA	NA
10	4	6	1	1.7	NA	NA	NA	NA
20	1	6	1	3	NA	NA	NA	NA
20	2	6	1	2	NA	NA	NA	NA
20	3	6	1	2.7	NA	NA	NA	NA
20	4	6	1	3.7	NA	NA	NA	NA
30	1	6	1	3	NA	NA	NA	NA
30	2	6	1	3.7	NA	NA	NA	NA
30	3	6	1	6	NA	NA	NA	NA
30	4	6	1	4	NA	NA	NA	NA
40	1	6	1	5	NA	NA	NA	NA
40	2	6	1	3.7	NA	NA	NA	NA
40	3	6	1	5.7	NA	NA	NA	NA
40	4	6	1	7	NA	NA	NA	NA
60	1	6	1	4	NA	NA	NA	NA
10	1	5	NA	1	NA	NA	NA	NA
10	2	5	NA	1	NA	NA	NA	NA
10	3	5	NA	1.3	NA	NA	NA	NA
10	4	5	NA	1.3	NA	NA	NA	NA
20	1	5	NA	2	NA	NA	NA	NA
20	2	5	NA	3	NA	NA	NA	NA
20	3	5	NA	3	NA	NA	NA	NA
20	4	5	NA	5	NA	NA	NA	NA
30	1	5	NA	7	NA	NA	NA	NA
30	2	5	NA	4	NA	NA	NA	NA

30	3	5	NA	7	NA	NA	NA	NA
30	4	5	NA	4.3	NA	NA	NA	NA
40	1	5	NA	4.7	NA	NA	NA	NA
40	2	5	NA	5	NA	NA	NA	NA
40	3	5	NA	3.3	NA	NA	NA	NA
40	4	5	NA	5.3	NA	NA	NA	NA
40	5	5	NA	NA	NA	NA	NA	NA
60	1	5	NA	4.7	NA	NA	NA	NA
10	1	5	NA	1	NA	NA	NA	NA
10	2	5	NA	1	NA	NA	NA	NA
10	3	5	NA	1.3	NA	NA	NA	NA
10	4	5	NA	1.7	NA	NA	NA	NA
20	1	5	NA	3	NA	NA	NA	NA
20	2	5	NA	2	NA	NA	NA	NA
20	3	5	NA	2.7	NA	NA	NA	NA
20	4	5	NA	3.7	NA	NA	NA	NA
30	1	5	NA	3	NA	NA	NA	NA
30	2	5	NA	3.7	NA	NA	NA	NA
30	3	5	NA	6	NA	NA	NA	NA
30	4	5	NA	4	NA	NA	NA	NA
40	1	5	NA	5	NA	NA	NA	NA
40	2	5	NA	3.7	NA	NA	NA	NA
40	3	5	NA	5.7	NA	NA	NA	NA
40	4	5	NA	7	NA	NA	NA	NA
40	5	5	NA	NA	NA	NA	NA	NA
60	1	5	NA	4	NA	NA	NA	NA

Dens: density; Replic: replicate; Stage [Nymph 5 (= 5) or Adult (= 6)]; Sex (1 = females, 2 = males); “NA“ indicates data not applicable or not available for the given combination.

(Part 2)

Dens	Replic	Stage	Sex	Prop dead D1	Prop dead D2	Prop dead D3	Mean prop dead 3 says	Sd prop dead 3 days
10	1	5	NA	0.10	0.11	0	0.07	0.06
10	2	5	NA	0.00	0.00	0	0	0
10	3	5	NA	0.00	0.00	0	0	0
10	4	5	NA	0.00	0.20	0	0.07	0.12
20	1	5	NA	0.25	0.20	0.33	0.26	0.07
20	2	5	NA	0.20	0.38	0.40	0.33	0.11
20	3	5	NA	0.35	0.23	0.50	0.36	0.14
20	4	5	NA	0.20	0.19	0.15	0.18	0.02
30	1	5	NA	0.27	0.36	0.14	0.26	0.11
30	2	5	NA	0.23	0.43	0.46	0.38	0.13
30	3	5	NA	0.20	0.21	0.26	0.22	0.03
30	4	5	NA	0.33	0.25	0.27	0.28	0.04
40	1	5	NA	0.13	0.34	0.30	0.26	0.12
40	2	5	NA	0.15	0.24	0.19	0.19	0.04
40	3	5	NA	0.13	0.17	0.38	0.23	0.14
40	4	5	NA	0.25	0.17	0.12	0.18	0.07
40	5	5	NA	0.05	0.11	0.06	0.07	0.03
60	1	5	NA	0.03	0.03	0.07	0.05	0.02
10	1	6	NA	0	0	0	0	0
10	2	6	NA	0	0	0	0	0
10	3	6	NA	0	0	0	0	0
10	4	6	NA	0.20	0.10	0.20	0.17	0.06
20	1	6	NA	0.10	0.00	0.15	0.08	0.08
20	2	6	NA	0.30	0.00	0	0.10	0.17
20	3	6	NA	0.05	0.00	0	0.02	0.03
20	4	6	NA	0.05	0.10	0.05	0.07	0.03
30	1	6	NA	0.07	0.07	0.03	0.06	0.02
30	2	6	NA	0.03	0.17	0.10	0.10	0.07
30	3	6	NA	0.27	0.07	0.03	0.12	0.13
30	4	6	NA	0.03	0.20	0.03	0.09	0.10
40	1	6	NA	0.15	0.08	0.08	0.10	0.04
40	2	6	NA	0.08	0.05	0.03	0.05	0.03

40	3	6	NA	0.13	0.05	0.08	0.08	0.04
40	4	6	NA	0.15	0.10	0.03	0.09	0.06
60	1	6	NA	0.27	0.17	0.08	0.17	0.09
10	1	6	1	NA	NA	NA	NA	NA
10	2	6	1	NA	NA	NA	NA	NA
10	3	6	1	NA	NA	NA	NA	NA
10	4	6	1	NA	NA	NA	NA	NA
20	1	6	1	NA	NA	NA	NA	NA
20	2	6	1	NA	NA	NA	NA	NA
20	3	6	1	NA	NA	NA	NA	NA
20	4	6	1	NA	NA	NA	NA	NA
30	1	6	1	NA	NA	NA	NA	NA
30	2	6	1	NA	NA	NA	NA	NA
30	3	6	1	NA	NA	NA	NA	NA
30	4	6	1	NA	NA	NA	NA	NA
40	1	6	1	NA	NA	NA	NA	NA
40	2	6	1	NA	NA	NA	NA	NA
40	3	6	1	NA	NA	NA	NA	NA
40	4	6	1	NA	NA	NA	NA	NA
60	1	6	1	NA	NA	NA	NA	NA
10	1	6	2	NA	NA	NA	NA	NA
10	2	6	2	NA	NA	NA	NA	NA
10	3	6	2	NA	NA	NA	NA	NA
10	4	6	2	NA	NA	NA	NA	NA
20	1	6	2	NA	NA	NA	NA	NA
20	2	6	2	NA	NA	NA	NA	NA
20	3	6	2	NA	NA	NA	NA	NA
20	4	6	2	NA	NA	NA	NA	NA
30	1	6	2	NA	NA	NA	NA	NA
30	2	6	2	NA	NA	NA	NA	NA
30	3	6	2	NA	NA	NA	NA	NA
30	4	6	2	NA	NA	NA	NA	NA
40	1	6	2	NA	NA	NA	NA	NA
40	2	6	2	NA	NA	NA	NA	NA
40	3	6	2	NA	NA	NA	NA	NA

40	4	6	2	NA	NA	NA	NA	NA
60	1	6	2	NA	NA	NA	NA	NA
10	1	6	NA	NA	NA	NA	NA	NA
10	2	6	NA	NA	NA	NA	NA	NA
10	3	6	NA	NA	NA	NA	NA	NA
10	4	6	NA	NA	NA	NA	NA	NA
20	1	6	NA	NA	NA	NA	NA	NA
20	2	6	NA	NA	NA	NA	NA	NA
20	3	6	NA	NA	NA	NA	NA	NA
20	4	6	NA	NA	NA	NA	NA	NA
30	1	6	NA	NA	NA	NA	NA	NA
30	2	6	NA	NA	NA	NA	NA	NA
30	3	6	NA	NA	NA	NA	NA	NA
30	4	6	NA	NA	NA	NA	NA	NA
40	1	6	NA	NA	NA	NA	NA	NA
40	2	6	NA	NA	NA	NA	NA	NA
40	3	6	NA	NA	NA	NA	NA	NA
40	4	6	NA	NA	NA	NA	NA	NA
60	1	6	NA	NA	NA	NA	NA	NA
10	1	5	NA	NA	NA	NA	NA	NA
10	1	5	NA	NA	NA	NA	NA	NA
10	2	5	NA	NA	NA	NA	NA	NA
10	3	5	NA	NA	NA	NA	NA	NA
10	4	5	NA	NA	NA	NA	NA	NA
20	1	5	NA	NA	NA	NA	NA	NA
20	2	5	NA	NA	NA	NA	NA	NA
20	3	5	NA	NA	NA	NA	NA	NA
20	4	5	NA	NA	NA	NA	NA	NA
30	1	5	NA	NA	NA	NA	NA	NA
30	2	5	NA	NA	NA	NA	NA	NA
30	3	5	NA	NA	NA	NA	NA	NA
30	4	5	NA	NA	NA	NA	NA	NA
40	1	5	NA	NA	NA	NA	NA	NA
40	2	5	NA	NA	NA	NA	NA	NA
40	3	5	NA	NA	NA	NA	NA	NA

40	4	5	NA	NA	NA	NA	NA	NA
60	1	5	NA	NA	NA	NA	NA	NA
10	1	6	NA	NA	NA	NA	NA	NA
10	2	6	NA	NA	NA	NA	NA	NA
10	3	6	NA	NA	NA	NA	NA	NA
10	4	6	NA	NA	NA	NA	NA	NA
20	1	6	NA	NA	NA	NA	NA	NA
20	2	6	NA	NA	NA	NA	NA	NA
20	3	6	NA	NA	NA	NA	NA	NA
20	4	6	NA	NA	NA	NA	NA	NA
30	1	6	NA	NA	NA	NA	NA	NA
30	2	6	NA	NA	NA	NA	NA	NA
30	3	6	NA	NA	NA	NA	NA	NA
30	4	6	NA	NA	NA	NA	NA	NA
40	1	6	NA	NA	NA	NA	NA	NA
40	2	6	NA	NA	NA	NA	NA	NA
40	3	6	NA	NA	NA	NA	NA	NA
40	4	6	NA	NA	NA	NA	NA	NA
60	1	6	NA	NA	NA	NA	NA	NA
10	1	5	NA	NA	NA	NA	NA	NA
10	2	5	NA	NA	NA	NA	NA	NA
10	3	5	NA	NA	NA	NA	NA	NA
10	4	5	NA	NA	NA	NA	NA	NA
20	1	5	NA	NA	NA	NA	NA	NA
20	2	5	NA	NA	NA	NA	NA	NA
20	3	5	NA	NA	NA	NA	NA	NA
20	4	5	NA	NA	NA	NA	NA	NA
30	1	5	NA	NA	NA	NA	NA	NA
30	2	5	NA	NA	NA	NA	NA	NA
30	3	5	NA	NA	NA	NA	NA	NA
30	4	5	NA	NA	NA	NA	NA	NA
40	1	5	NA	NA	NA	NA	NA	NA
40	2	5	NA	NA	NA	NA	NA	NA
40	3	5	NA	NA	NA	NA	NA	NA
40	4	5	NA	NA	NA	NA	NA	NA

40	5	5	NA	NA	NA	NA	NA	NA
60	1	5	NA	NA	NA	NA	NA	NA
10	1	6	1	NA	NA	NA	NA	NA
10	2	6	1	NA	NA	NA	NA	NA
10	3	6	1	NA	NA	NA	NA	NA
10	4	6	1	NA	NA	NA	NA	NA
20	1	6	1	NA	NA	NA	NA	NA
20	2	6	1	NA	NA	NA	NA	NA
20	3	6	1	NA	NA	NA	NA	NA
20	4	6	1	NA	NA	NA	NA	NA
30	1	6	1	NA	NA	NA	NA	NA
30	2	6	1	NA	NA	NA	NA	NA
30	3	6	1	NA	NA	NA	NA	NA
30	4	6	1	NA	NA	NA	NA	NA
40	1	6	1	NA	NA	NA	NA	NA
40	2	6	1	NA	NA	NA	NA	NA
40	3	6	1	NA	NA	NA	NA	NA
40	4	6	1	NA	NA	NA	NA	NA
60	1	6	1	NA	NA	NA	NA	NA
10	1	5	NA	NA	NA	NA	NA	NA
10	2	5	NA	NA	NA	NA	NA	NA
10	3	5	NA	NA	NA	NA	NA	NA
10	4	5	NA	NA	NA	NA	NA	NA
20	1	5	NA	NA	NA	NA	NA	NA
20	2	5	NA	NA	NA	NA	NA	NA
20	3	5	NA	NA	NA	NA	NA	NA
20	4	5	NA	NA	NA	NA	NA	NA
30	1	5	NA	NA	NA	NA	NA	NA
30	2	5	NA	NA	NA	NA	NA	NA
30	3	5	NA	NA	NA	NA	NA	NA
30	4	5	NA	NA	NA	NA	NA	NA
40	1	5	NA	NA	NA	NA	NA	NA
40	2	5	NA	NA	NA	NA	NA	NA
40	3	5	NA	NA	NA	NA	NA	NA
40	4	5	NA	NA	NA	NA	NA	NA

40	5	5	NA	NA	NA	NA	NA	NA
60	1	5	NA	NA	NA	NA	NA	NA
10	1	5	NA	NA	NA	NA	NA	NA
10	2	5	NA	NA	NA	NA	NA	NA
10	3	5	NA	NA	NA	NA	NA	NA
10	4	5	NA	NA	NA	NA	NA	NA
20	1	5	NA	NA	NA	NA	NA	NA
20	2	5	NA	NA	NA	NA	NA	NA
20	3	5	NA	NA	NA	NA	NA	NA
20	4	5	NA	NA	NA	NA	NA	NA
30	1	5	NA	NA	NA	NA	NA	NA
30	2	5	NA	NA	NA	NA	NA	NA
30	3	5	NA	NA	NA	NA	NA	NA
30	4	5	NA	NA	NA	NA	NA	NA
40	1	5	NA	NA	NA	NA	NA	NA
40	2	5	NA	NA	NA	NA	NA	NA
40	3	5	NA	NA	NA	NA	NA	NA
40	4	5	NA	NA	NA	NA	NA	NA
40	5	5	NA	NA	NA	NA	NA	NA
60	1	5	NA	NA	NA	NA	NA	NA

Dens: density; Replic: replicate; Stage [Nymph 5 (= 5) or Adult (= 6)]; Sex (1 = females, 2 = males); “NA“ indicates data not applicable or not available for the given combination.

(Part 3)

Dens	Replic	Stage	Sex	N disp. Dead D1	N disp. Dead D2	N disp. Dead D3	N disp. Dead 3 Days	Prop disp. Dead D1
10	1	5	NA	0	0	0	0	0
10	2	5	NA	0	0	0	0	0
10	3	5	NA	0	0	0	0	0
10	4	5	NA	0	0	0	0	0
20	1	5	NA	1	0	0	1	0.05
20	2	5	NA	0	1	0	1	0
20	3	5	NA	1	0	0	1	0.05
20	4	5	NA	0	1	1	2	0
30	1	5	NA	1	0	0	1	0.03
30	2	5	NA	0	0	0	0	0
30	3	5	NA	0	0	0	0	0
30	4	5	NA	0	1	0	1	0
40	1	5	NA	1	0	3	4	0.03
40	2	5	NA	1	1	0	2	0.03
40	3	5	NA	1	1	2	4	0.03
40	4	5	NA	1	1	0	2	0.03
40	5	5	NA	1	0	0	1	0.03
60	1	5	NA	0	0	0	0	0
10	1	6	NA	0	0	0	0	0
10	2	6	NA	0	0	0	0	0
10	3	6	NA	0	0	0	0	0
10	4	6	NA	0	0	0	0	0
20	1	6	NA	0	0	0	0	0
20	2	6	NA	0	0	0	0	0
20	3	6	NA	0	0	0	0	0
20	4	6	NA	0	0	0	0	0
30	1	6	NA	0	0	1	1	0
30	2	6	NA	0	1	0	1	0
30	3	6	NA	0	0	0	0	0
30	4	6	NA	0	1	0	1	0
40	1	6	NA	0	0	1	1	0
40	2	6	NA	2	1	0	3	0.05
40	3	6	NA	0	0	0	0	0

40	4	6	NA	3	1	8	12	0.08
60	1	6	NA	1	2	1	4	0.02
10	1	6	1	NA	NA	NA	NA	NA
10	2	6	1	NA	NA	NA	NA	NA
10	3	6	1	NA	NA	NA	NA	NA
10	4	6	1	NA	NA	NA	NA	NA
20	1	6	1	NA	NA	NA	NA	NA
20	2	6	1	NA	NA	NA	NA	NA
20	3	6	1	NA	NA	NA	NA	NA
20	4	6	1	NA	NA	NA	NA	NA
30	1	6	1	NA	NA	NA	NA	NA
30	2	6	1	NA	NA	NA	NA	NA
30	3	6	1	NA	NA	NA	NA	NA
30	4	6	1	NA	NA	NA	NA	NA
40	1	6	1	NA	NA	NA	NA	NA
40	2	6	1	NA	NA	NA	NA	NA
40	3	6	1	NA	NA	NA	NA	NA
40	4	6	1	NA	NA	NA	NA	NA
60	1	6	1	NA	NA	NA	NA	NA
10	1	6	2	NA	NA	NA	NA	NA
10	2	6	2	NA	NA	NA	NA	NA
10	3	6	2	NA	NA	NA	NA	NA
10	4	6	2	NA	NA	NA	NA	NA
20	1	6	2	NA	NA	NA	NA	NA
20	2	6	2	NA	NA	NA	NA	NA
20	3	6	2	NA	NA	NA	NA	NA
20	4	6	2	NA	NA	NA	NA	NA
30	1	6	2	NA	NA	NA	NA	NA
30	2	6	2	NA	NA	NA	NA	NA
30	3	6	2	NA	NA	NA	NA	NA
30	4	6	2	NA	NA	NA	NA	NA
40	1	6	2	NA	NA	NA	NA	NA
40	2	6	2	NA	NA	NA	NA	NA
40	3	6	2	NA	NA	NA	NA	NA
40	4	6	2	NA	NA	NA	NA	NA
60	1	6	2	NA	NA	NA	NA	NA

10	1	6	NA	NA	NA	NA	NA	NA
10	2	6	NA	NA	NA	NA	NA	NA
10	3	6	NA	NA	NA	NA	NA	NA
10	4	6	NA	NA	NA	NA	NA	NA
20	1	6	NA	NA	NA	NA	NA	NA
20	2	6	NA	NA	NA	NA	NA	NA
20	3	6	NA	NA	NA	NA	NA	NA
20	4	6	NA	NA	NA	NA	NA	NA
30	1	6	NA	NA	NA	NA	NA	NA
30	2	6	NA	NA	NA	NA	NA	NA
30	3	6	NA	NA	NA	NA	NA	NA
30	4	6	NA	NA	NA	NA	NA	NA
40	1	6	NA	NA	NA	NA	NA	NA
40	2	6	NA	NA	NA	NA	NA	NA
40	3	6	NA	NA	NA	NA	NA	NA
40	4	6	NA	NA	NA	NA	NA	NA
60	1	6	NA	NA	NA	NA	NA	NA
10	1	5	NA	NA	NA	NA	NA	NA
10	1	5	NA	NA	NA	NA	NA	NA
10	2	5	NA	NA	NA	NA	NA	NA
10	3	5	NA	NA	NA	NA	NA	NA
10	4	5	NA	NA	NA	NA	NA	NA
20	1	5	NA	NA	NA	NA	NA	NA
20	2	5	NA	NA	NA	NA	NA	NA
20	3	5	NA	NA	NA	NA	NA	NA
20	4	5	NA	NA	NA	NA	NA	NA
30	1	5	NA	NA	NA	NA	NA	NA
30	2	5	NA	NA	NA	NA	NA	NA
30	3	5	NA	NA	NA	NA	NA	NA
30	4	5	NA	NA	NA	NA	NA	NA
40	1	5	NA	NA	NA	NA	NA	NA
40	2	5	NA	NA	NA	NA	NA	NA
40	3	5	NA	NA	NA	NA	NA	NA
40	4	5	NA	NA	NA	NA	NA	NA
60	1	5	NA	NA	NA	NA	NA	NA
10	1	6	NA	NA	NA	NA	NA	NA

10	2	6	NA	NA	NA	NA	NA	NA
10	3	6	NA	NA	NA	NA	NA	NA
10	4	6	NA	NA	NA	NA	NA	NA
20	1	6	NA	NA	NA	NA	NA	NA
20	2	6	NA	NA	NA	NA	NA	NA
20	3	6	NA	NA	NA	NA	NA	NA
20	4	6	NA	NA	NA	NA	NA	NA
30	1	6	NA	NA	NA	NA	NA	NA
30	2	6	NA	NA	NA	NA	NA	NA
30	3	6	NA	NA	NA	NA	NA	NA
30	4	6	NA	NA	NA	NA	NA	NA
40	1	6	NA	NA	NA	NA	NA	NA
40	2	6	NA	NA	NA	NA	NA	NA
40	3	6	NA	NA	NA	NA	NA	NA
40	4	6	NA	NA	NA	NA	NA	NA
60	1	6	NA	NA	NA	NA	NA	NA
10	1	5	NA	NA	NA	NA	NA	NA
10	2	5	NA	NA	NA	NA	NA	NA
10	3	5	NA	NA	NA	NA	NA	NA
10	4	5	NA	NA	NA	NA	NA	NA
20	1	5	NA	NA	NA	NA	NA	NA
20	2	5	NA	NA	NA	NA	NA	NA
20	3	5	NA	NA	NA	NA	NA	NA
20	4	5	NA	NA	NA	NA	NA	NA
30	1	5	NA	NA	NA	NA	NA	NA
30	2	5	NA	NA	NA	NA	NA	NA
30	3	5	NA	NA	NA	NA	NA	NA
30	4	5	NA	NA	NA	NA	NA	NA
40	1	5	NA	NA	NA	NA	NA	NA
40	2	5	NA	NA	NA	NA	NA	NA
40	3	5	NA	NA	NA	NA	NA	NA
40	4	5	NA	NA	NA	NA	NA	NA
40	5	5	NA	NA	NA	NA	NA	NA
60	1	5	NA	NA	NA	NA	NA	NA
10	1	6	1	NA	NA	NA	NA	NA
10	2	6	1	NA	NA	NA	NA	NA

10	3	6	1	NA	NA	NA	NA	NA
10	4	6	1	NA	NA	NA	NA	NA
20	1	6	1	NA	NA	NA	NA	NA
20	2	6	1	NA	NA	NA	NA	NA
20	3	6	1	NA	NA	NA	NA	NA
20	4	6	1	NA	NA	NA	NA	NA
30	1	6	1	NA	NA	NA	NA	NA
30	2	6	1	NA	NA	NA	NA	NA
30	3	6	1	NA	NA	NA	NA	NA
30	4	6	1	NA	NA	NA	NA	NA
40	1	6	1	NA	NA	NA	NA	NA
40	2	6	1	NA	NA	NA	NA	NA
40	3	6	1	NA	NA	NA	NA	NA
40	4	6	1	NA	NA	NA	NA	NA
60	1	6	1	NA	NA	NA	NA	NA
10	1	5	NA	NA	NA	NA	NA	NA
10	2	5	NA	NA	NA	NA	NA	NA
10	3	5	NA	NA	NA	NA	NA	NA
10	4	5	NA	NA	NA	NA	NA	NA
20	1	5	NA	NA	NA	NA	NA	NA
20	2	5	NA	NA	NA	NA	NA	NA
20	3	5	NA	NA	NA	NA	NA	NA
20	4	5	NA	NA	NA	NA	NA	NA
30	1	5	NA	NA	NA	NA	NA	NA
30	2	5	NA	NA	NA	NA	NA	NA
30	3	5	NA	NA	NA	NA	NA	NA
30	4	5	NA	NA	NA	NA	NA	NA
40	1	5	NA	NA	NA	NA	NA	NA
40	2	5	NA	NA	NA	NA	NA	NA
40	3	5	NA	NA	NA	NA	NA	NA
40	4	5	NA	NA	NA	NA	NA	NA
40	5	5	NA	NA	NA	NA	NA	NA
60	1	5	NA	NA	NA	NA	NA	NA
10	1	5	NA	NA	NA	NA	NA	NA
10	2	5	NA	NA	NA	NA	NA	NA
10	3	5	NA	NA	NA	NA	NA	NA

10	4	5	NA	NA	NA	NA	NA	NA
20	1	5	NA	NA	NA	NA	NA	NA
20	2	5	NA	NA	NA	NA	NA	NA
20	3	5	NA	NA	NA	NA	NA	NA
20	4	5	NA	NA	NA	NA	NA	NA
30	1	5	NA	NA	NA	NA	NA	NA
30	2	5	NA	NA	NA	NA	NA	NA
30	3	5	NA	NA	NA	NA	NA	NA
30	4	5	NA	NA	NA	NA	NA	NA
40	1	5	NA	NA	NA	NA	NA	NA
40	2	5	NA	NA	NA	NA	NA	NA
40	3	5	NA	NA	NA	NA	NA	NA
40	4	5	NA	NA	NA	NA	NA	NA
40	5	5	NA	NA	NA	NA	NA	NA
60	1	5	NA	NA	NA	NA	NA	NA

Dens: density; Replic: replicate; Stage [Nymph 5 (= 5) or Adult (= 6)]; Sex (1 = females, 2 = males); “NA“ indicates data not applicable or not available for the given combination.

(Part 4)

Dens	Replic	Stage	Sex	Prop disp. Dead D2	Prop disp. Dead D3	Mean prop disp dead 3 Days	Sd prop disp dead 3 Days	N dead fed D1
10	1	5	NA	0	0	0	0	0
10	2	5	NA	0	0	0	0	0
10	3	5	NA	0	0	0	0	0
10	4	5	NA	0	0	0	0	0
20	1	5	NA	0	0	0.02	0.03	0
20	2	5	NA	0.05	0	0.02	0.03	0
20	3	5	NA	0	0	0.02	0.03	2
20	4	5	NA	0.05	0.05	0.03	0.03	0
30	1	5	NA	0	0	0.01	0.02	3
30	2	5	NA	0	0	0	0	4
30	3	5	NA	0	0	0	0	0
30	4	5	NA	0.03	0	0.01	0.02	0
40	1	5	NA	0	0.08	0.03	0.04	0
40	2	5	NA	0.03	0	0.02	0.02	2
40	3	5	NA	0.03	0.05	0.03	0.02	1
40	4	5	NA	0.03	0	0.02	0.02	3
40	5	5	NA	0	0	0.01	0.01	0
60	1	5	NA	0	0	0	0	0
10	1	6	NA	0	0	0	0	0
10	2	6	NA	0	0	0	0	0
10	3	6	NA	0	0	0	0	0
10	4	6	NA	0	0	0	0	0
20	1	6	NA	0	0	0	0	0
20	2	6	NA	0	0	0	0	0
20	3	6	NA	0	0	0	0	0
20	4	6	NA	0	0	0	0	0
30	1	6	NA	0	0.03	0.01	0.02	0
30	2	6	NA	0.03	0	0.01	0.02	1
30	3	6	NA	0	0	0	0	1
30	4	6	NA	0.03	0	0.01	0.02	0
40	1	6	NA	0	0.03	0.01	0.01	9
40	2	6	NA	0.03	0	0.03	0.03	0
40	3	6	NA	0	0	0	0	0

40	4	6	NA	0.03	0.20	0.10	0.09	0
60	1	6	NA	0.03	0.02	0.02	0.01	1
10	1	6	1	NA	NA	NA	NA	NA
10	2	6	1	NA	NA	NA	NA	NA
10	3	6	1	NA	NA	NA	NA	NA
10	4	6	1	NA	NA	NA	NA	NA
20	1	6	1	NA	NA	NA	NA	NA
20	2	6	1	NA	NA	NA	NA	NA
20	3	6	1	NA	NA	NA	NA	NA
20	4	6	1	NA	NA	NA	NA	NA
30	1	6	1	NA	NA	NA	NA	NA
30	2	6	1	NA	NA	NA	NA	NA
30	3	6	1	NA	NA	NA	NA	NA
30	4	6	1	NA	NA	NA	NA	NA
40	1	6	1	NA	NA	NA	NA	NA
40	2	6	1	NA	NA	NA	NA	NA
40	3	6	1	NA	NA	NA	NA	NA
40	4	6	1	NA	NA	NA	NA	NA
60	1	6	1	NA	NA	NA	NA	NA
10	1	6	2	NA	NA	NA	NA	NA
10	2	6	2	NA	NA	NA	NA	NA
10	3	6	2	NA	NA	NA	NA	NA
10	4	6	2	NA	NA	NA	NA	NA
20	1	6	2	NA	NA	NA	NA	NA
20	2	6	2	NA	NA	NA	NA	NA
20	3	6	2	NA	NA	NA	NA	NA
20	4	6	2	NA	NA	NA	NA	NA
30	1	6	2	NA	NA	NA	NA	NA
30	2	6	2	NA	NA	NA	NA	NA
30	3	6	2	NA	NA	NA	NA	NA
30	4	6	2	NA	NA	NA	NA	NA
40	1	6	2	NA	NA	NA	NA	NA
40	2	6	2	NA	NA	NA	NA	NA
40	3	6	2	NA	NA	NA	NA	NA
40	4	6	2	NA	NA	NA	NA	NA
60	1	6	2	NA	NA	NA	NA	NA
10	1	6	NA	NA	NA	NA	NA	NA

10	2	6	NA	NA	NA	NA	NA	NA
10	3	6	NA	NA	NA	NA	NA	NA
10	4	6	NA	NA	NA	NA	NA	NA
20	1	6	NA	NA	NA	NA	NA	NA
20	2	6	NA	NA	NA	NA	NA	NA
20	3	6	NA	NA	NA	NA	NA	NA
20	4	6	NA	NA	NA	NA	NA	NA
30	1	6	NA	NA	NA	NA	NA	NA
30	2	6	NA	NA	NA	NA	NA	NA
30	3	6	NA	NA	NA	NA	NA	NA
30	4	6	NA	NA	NA	NA	NA	NA
40	1	6	NA	NA	NA	NA	NA	NA
40	2	6	NA	NA	NA	NA	NA	NA
40	3	6	NA	NA	NA	NA	NA	NA
40	4	6	NA	NA	NA	NA	NA	NA
60	1	6	NA	NA	NA	NA	NA	NA
10	1	5	NA	NA	NA	NA	NA	NA
10	1	5	NA	NA	NA	NA	NA	NA
10	2	5	NA	NA	NA	NA	NA	NA
10	3	5	NA	NA	NA	NA	NA	NA
10	4	5	NA	NA	NA	NA	NA	NA
20	1	5	NA	NA	NA	NA	NA	NA
20	2	5	NA	NA	NA	NA	NA	NA
20	3	5	NA	NA	NA	NA	NA	NA
20	4	5	NA	NA	NA	NA	NA	NA
30	1	5	NA	NA	NA	NA	NA	NA
30	2	5	NA	NA	NA	NA	NA	NA
30	3	5	NA	NA	NA	NA	NA	NA
30	4	5	NA	NA	NA	NA	NA	NA
40	1	5	NA	NA	NA	NA	NA	NA
40	2	5	NA	NA	NA	NA	NA	NA
40	3	5	NA	NA	NA	NA	NA	NA
40	4	5	NA	NA	NA	NA	NA	NA
60	1	5	NA	NA	NA	NA	NA	NA
10	1	6	NA	NA	NA	NA	NA	NA
10	2	6	NA	NA	NA	NA	NA	NA
10	3	6	NA	NA	NA	NA	NA	NA

10	4	6	NA	NA	NA	NA	NA	NA
20	1	6	NA	NA	NA	NA	NA	NA
20	2	6	NA	NA	NA	NA	NA	NA
20	3	6	NA	NA	NA	NA	NA	NA
20	4	6	NA	NA	NA	NA	NA	NA
30	1	6	NA	NA	NA	NA	NA	NA
30	2	6	NA	NA	NA	NA	NA	NA
30	3	6	NA	NA	NA	NA	NA	NA
30	4	6	NA	NA	NA	NA	NA	NA
40	1	6	NA	NA	NA	NA	NA	NA
40	2	6	NA	NA	NA	NA	NA	NA
40	3	6	NA	NA	NA	NA	NA	NA
40	4	6	NA	NA	NA	NA	NA	NA
60	1	6	NA	NA	NA	NA	NA	NA
10	1	5	NA	NA	NA	NA	NA	NA
10	2	5	NA	NA	NA	NA	NA	NA
10	3	5	NA	NA	NA	NA	NA	NA
10	4	5	NA	NA	NA	NA	NA	NA
20	1	5	NA	NA	NA	NA	NA	NA
20	2	5	NA	NA	NA	NA	NA	NA
20	3	5	NA	NA	NA	NA	NA	NA
20	4	5	NA	NA	NA	NA	NA	NA
30	1	5	NA	NA	NA	NA	NA	NA
30	2	5	NA	NA	NA	NA	NA	NA
30	3	5	NA	NA	NA	NA	NA	NA
30	4	5	NA	NA	NA	NA	NA	NA
40	1	5	NA	NA	NA	NA	NA	NA
40	2	5	NA	NA	NA	NA	NA	NA
40	3	5	NA	NA	NA	NA	NA	NA
40	4	5	NA	NA	NA	NA	NA	NA
40	5	5	NA	NA	NA	NA	NA	NA
60	1	5	NA	NA	NA	NA	NA	NA
10	1	6	1	NA	NA	NA	NA	NA
10	2	6	1	NA	NA	NA	NA	NA
10	3	6	1	NA	NA	NA	NA	NA
10	4	6	1	NA	NA	NA	NA	NA
20	1	6	1	NA	NA	NA	NA	NA

20	2	6	1	NA	NA	NA	NA	NA
20	3	6	1	NA	NA	NA	NA	NA
20	4	6	1	NA	NA	NA	NA	NA
30	1	6	1	NA	NA	NA	NA	NA
30	2	6	1	NA	NA	NA	NA	NA
30	3	6	1	NA	NA	NA	NA	NA
30	4	6	1	NA	NA	NA	NA	NA
40	1	6	1	NA	NA	NA	NA	NA
40	2	6	1	NA	NA	NA	NA	NA
40	3	6	1	NA	NA	NA	NA	NA
40	4	6	1	NA	NA	NA	NA	NA
60	1	6	1	NA	NA	NA	NA	NA
10	1	5	NA	NA	NA	NA	NA	NA
10	2	5	NA	NA	NA	NA	NA	NA
10	3	5	NA	NA	NA	NA	NA	NA
10	4	5	NA	NA	NA	NA	NA	NA
20	1	5	NA	NA	NA	NA	NA	NA
20	2	5	NA	NA	NA	NA	NA	NA
20	3	5	NA	NA	NA	NA	NA	NA
20	4	5	NA	NA	NA	NA	NA	NA
30	1	5	NA	NA	NA	NA	NA	NA
30	2	5	NA	NA	NA	NA	NA	NA
30	3	5	NA	NA	NA	NA	NA	NA
30	4	5	NA	NA	NA	NA	NA	NA
40	1	5	NA	NA	NA	NA	NA	NA
40	2	5	NA	NA	NA	NA	NA	NA
40	3	5	NA	NA	NA	NA	NA	NA
40	4	5	NA	NA	NA	NA	NA	NA
40	5	5	NA	NA	NA	NA	NA	NA
60	1	5	NA	NA	NA	NA	NA	NA
10	1	5	NA	NA	NA	NA	NA	NA
10	2	5	NA	NA	NA	NA	NA	NA
10	3	5	NA	NA	NA	NA	NA	NA
10	4	5	NA	NA	NA	NA	NA	NA
20	1	5	NA	NA	NA	NA	NA	NA
20	2	5	NA	NA	NA	NA	NA	NA
20	3	5	NA	NA	NA	NA	NA	NA

20	4	5	NA	NA	NA	NA	NA	NA
30	1	5	NA	NA	NA	NA	NA	NA
30	2	5	NA	NA	NA	NA	NA	NA
30	3	5	NA	NA	NA	NA	NA	NA
30	4	5	NA	NA	NA	NA	NA	NA
40	1	5	NA	NA	NA	NA	NA	NA
40	2	5	NA	NA	NA	NA	NA	NA
40	3	5	NA	NA	NA	NA	NA	NA
40	4	5	NA	NA	NA	NA	NA	NA
40	5	5	NA	NA	NA	NA	NA	NA
60	1	5	NA	NA	NA	NA	NA	NA

Dens: density; Replic: replicate; Stage [Nymph 5 (= 5) or Adult (= 6)]; Sex (1 = females, 2 = males); “NA“ indicates data not applicable or not available for the given combination.

(Part 5)

Dens	Replic	Stage	Sex	N dead fed D2	N dead fed D3	N dead fed 3 Days	Prop dead fed D1	Prop dead fed D2
10	1	5	NA	0	0	0	0	0
10	2	5	NA	0	0	0	0	0
10	3	5	NA	0	0	0	0	0
10	4	5	NA	1	0	1	0	0.10
20	1	5	NA	2	2	4	0	0.10
20	2	5	NA	4	3	7	0	0.20
20	3	5	NA	2	5	9	0.10	0.11
20	4	5	NA	1	3	4	0.00	0.05
30	1	5	NA	4	1	8	0.10	0.15
30	2	5	NA	7	2	13	0.13	0.27
30	3	5	NA	4	2	6	0.00	0.13
30	4	5	NA	2	4	6	0.00	0.07
40	1	5	NA	6	7	13	0.00	0.15
40	2	5	NA	8	4	14	0.05	0.21
40	3	5	NA	4	9	14	0.03	0.10
40	4	5	NA	2	3	8	0.08	0.05
40	5	5	NA	2	1	3	0	0.05
60	1	5	NA	2	4	6	0	0.03
10	1	6	NA	0	0	0	0	0
10	2	6	NA	0	0	0	0	0
10	3	6	NA	0	0	0	0	0
10	4	6	NA	0	0	0	0	0
20	1	6	NA	0	3	3	0	0
20	2	6	NA	0	0	0	0	0
20	3	6	NA	0	0	0	0	0
20	4	6	NA	2	0	2	0	0.10
30	1	6	NA	1	1	2	0	0.03
30	2	6	NA	4	2	7	0.03	0.13
30	3	6	NA	1	0	2	0.03	0.03
30	4	6	NA	5	1	6	0	0.17
40	1	6	NA	1	2	12	0.23	0.03
40	2	6	NA	0	1	1	0	0
40	3	6	NA	2	1	3	0	0.05

40	4	6	NA	3	0	3	0	0.08
60	1	6	NA	4	3	8	0.02	0.07
10	1	6	1	NA	NA	NA	NA	NA
10	2	6	1	NA	NA	NA	NA	NA
10	3	6	1	NA	NA	NA	NA	NA
10	4	6	1	NA	NA	NA	NA	NA
20	1	6	1	NA	NA	NA	NA	NA
20	2	6	1	NA	NA	NA	NA	NA
20	3	6	1	NA	NA	NA	NA	NA
20	4	6	1	NA	NA	NA	NA	NA
30	1	6	1	NA	NA	NA	NA	NA
30	2	6	1	NA	NA	NA	NA	NA
30	3	6	1	NA	NA	NA	NA	NA
30	4	6	1	NA	NA	NA	NA	NA
40	1	6	1	NA	NA	NA	NA	NA
40	2	6	1	NA	NA	NA	NA	NA
40	3	6	1	NA	NA	NA	NA	NA
40	4	6	1	NA	NA	NA	NA	NA
60	1	6	1	NA	NA	NA	NA	NA
10	1	6	2	NA	NA	NA	NA	NA
10	2	6	2	NA	NA	NA	NA	NA
10	3	6	2	NA	NA	NA	NA	NA
10	4	6	2	NA	NA	NA	NA	NA
20	1	6	2	NA	NA	NA	NA	NA
20	2	6	2	NA	NA	NA	NA	NA
20	3	6	2	NA	NA	NA	NA	NA
20	4	6	2	NA	NA	NA	NA	NA
30	1	6	2	NA	NA	NA	NA	NA
30	2	6	2	NA	NA	NA	NA	NA
30	3	6	2	NA	NA	NA	NA	NA
30	4	6	2	NA	NA	NA	NA	NA
40	1	6	2	NA	NA	NA	NA	NA
40	2	6	2	NA	NA	NA	NA	NA
40	3	6	2	NA	NA	NA	NA	NA
40	4	6	2	NA	NA	NA	NA	NA
60	1	6	2	NA	NA	NA	NA	NA

10	1	6	NA	NA	NA	NA	NA	NA
10	2	6	NA	NA	NA	NA	NA	NA
10	3	6	NA	NA	NA	NA	NA	NA
10	4	6	NA	NA	NA	NA	NA	NA
20	1	6	NA	NA	NA	NA	NA	NA
20	2	6	NA	NA	NA	NA	NA	NA
20	3	6	NA	NA	NA	NA	NA	NA
20	4	6	NA	NA	NA	NA	NA	NA
30	1	6	NA	NA	NA	NA	NA	NA
30	2	6	NA	NA	NA	NA	NA	NA
30	3	6	NA	NA	NA	NA	NA	NA
30	4	6	NA	NA	NA	NA	NA	NA
40	1	6	NA	NA	NA	NA	NA	NA
40	2	6	NA	NA	NA	NA	NA	NA
40	3	6	NA	NA	NA	NA	NA	NA
40	4	6	NA	NA	NA	NA	NA	NA
60	1	6	NA	NA	NA	NA	NA	NA
10	1	5	NA	NA	NA	NA	NA	NA
10	1	5	NA	NA	NA	NA	NA	NA
10	2	5	NA	NA	NA	NA	NA	NA
10	3	5	NA	NA	NA	NA	NA	NA
10	4	5	NA	NA	NA	NA	NA	NA
20	1	5	NA	NA	NA	NA	NA	NA
20	2	5	NA	NA	NA	NA	NA	NA
20	3	5	NA	NA	NA	NA	NA	NA
20	4	5	NA	NA	NA	NA	NA	NA
30	1	5	NA	NA	NA	NA	NA	NA
30	2	5	NA	NA	NA	NA	NA	NA
30	3	5	NA	NA	NA	NA	NA	NA
30	4	5	NA	NA	NA	NA	NA	NA
40	1	5	NA	NA	NA	NA	NA	NA
40	2	5	NA	NA	NA	NA	NA	NA
40	3	5	NA	NA	NA	NA	NA	NA
40	4	5	NA	NA	NA	NA	NA	NA
60	1	5	NA	NA	NA	NA	NA	NA
10	1	6	NA	NA	NA	NA	NA	NA

10	2	6	NA	NA	NA	NA	NA	NA
10	3	6	NA	NA	NA	NA	NA	NA
10	4	6	NA	NA	NA	NA	NA	NA
20	1	6	NA	NA	NA	NA	NA	NA
20	2	6	NA	NA	NA	NA	NA	NA
20	3	6	NA	NA	NA	NA	NA	NA
20	4	6	NA	NA	NA	NA	NA	NA
30	1	6	NA	NA	NA	NA	NA	NA
30	2	6	NA	NA	NA	NA	NA	NA
30	3	6	NA	NA	NA	NA	NA	NA
30	4	6	NA	NA	NA	NA	NA	NA
40	1	6	NA	NA	NA	NA	NA	NA
40	2	6	NA	NA	NA	NA	NA	NA
40	3	6	NA	NA	NA	NA	NA	NA
40	4	6	NA	NA	NA	NA	NA	NA
60	1	6	NA	NA	NA	NA	NA	NA
10	1	5	NA	NA	NA	NA	NA	NA
10	2	5	NA	NA	NA	NA	NA	NA
10	3	5	NA	NA	NA	NA	NA	NA
10	4	5	NA	NA	NA	NA	NA	NA
20	1	5	NA	NA	NA	NA	NA	NA
20	2	5	NA	NA	NA	NA	NA	NA
20	3	5	NA	NA	NA	NA	NA	NA
20	4	5	NA	NA	NA	NA	NA	NA
30	1	5	NA	NA	NA	NA	NA	NA
30	2	5	NA	NA	NA	NA	NA	NA
30	3	5	NA	NA	NA	NA	NA	NA
30	4	5	NA	NA	NA	NA	NA	NA
40	1	5	NA	NA	NA	NA	NA	NA
40	2	5	NA	NA	NA	NA	NA	NA
40	3	5	NA	NA	NA	NA	NA	NA
40	4	5	NA	NA	NA	NA	NA	NA
40	5	5	NA	NA	NA	NA	NA	NA
60	1	5	NA	NA	NA	NA	NA	NA
10	1	6	1	NA	NA	NA	NA	NA
10	2	6	1	NA	NA	NA	NA	NA

10	3	6	1	NA	NA	NA	NA	NA
10	4	6	1	NA	NA	NA	NA	NA
20	1	6	1	NA	NA	NA	NA	NA
20	2	6	1	NA	NA	NA	NA	NA
20	3	6	1	NA	NA	NA	NA	NA
20	4	6	1	NA	NA	NA	NA	NA
30	1	6	1	NA	NA	NA	NA	NA
30	2	6	1	NA	NA	NA	NA	NA
30	3	6	1	NA	NA	NA	NA	NA
30	4	6	1	NA	NA	NA	NA	NA
40	1	6	1	NA	NA	NA	NA	NA
40	2	6	1	NA	NA	NA	NA	NA
40	3	6	1	NA	NA	NA	NA	NA
40	4	6	1	NA	NA	NA	NA	NA
60	1	6	1	NA	NA	NA	NA	NA
10	1	5	NA	NA	NA	NA	NA	NA
10	2	5	NA	NA	NA	NA	NA	NA
10	3	5	NA	NA	NA	NA	NA	NA
10	4	5	NA	NA	NA	NA	NA	NA
20	1	5	NA	NA	NA	NA	NA	NA
20	2	5	NA	NA	NA	NA	NA	NA
20	3	5	NA	NA	NA	NA	NA	NA
20	4	5	NA	NA	NA	NA	NA	NA
30	1	5	NA	NA	NA	NA	NA	NA
30	2	5	NA	NA	NA	NA	NA	NA
30	3	5	NA	NA	NA	NA	NA	NA
30	4	5	NA	NA	NA	NA	NA	NA
40	1	5	NA	NA	NA	NA	NA	NA
40	2	5	NA	NA	NA	NA	NA	NA
40	3	5	NA	NA	NA	NA	NA	NA
40	4	5	NA	NA	NA	NA	NA	NA
40	5	5	NA	NA	NA	NA	NA	NA
60	1	5	NA	NA	NA	NA	NA	NA
10	1	5	NA	NA	NA	NA	NA	NA
10	2	5	NA	NA	NA	NA	NA	NA
10	3	5	NA	NA	NA	NA	NA	NA

10	4	5	NA	NA	NA	NA	NA	NA
20	1	5	NA	NA	NA	NA	NA	NA
20	2	5	NA	NA	NA	NA	NA	NA
20	3	5	NA	NA	NA	NA	NA	NA
20	4	5	NA	NA	NA	NA	NA	NA
30	1	5	NA	NA	NA	NA	NA	NA
30	2	5	NA	NA	NA	NA	NA	NA
30	3	5	NA	NA	NA	NA	NA	NA
30	4	5	NA	NA	NA	NA	NA	NA
40	1	5	NA	NA	NA	NA	NA	NA
40	2	5	NA	NA	NA	NA	NA	NA
40	3	5	NA	NA	NA	NA	NA	NA
40	4	5	NA	NA	NA	NA	NA	NA
40	5	5	NA	NA	NA	NA	NA	NA
60	1	5	NA	NA	NA	NA	NA	NA

Dens: density; Replic: replicate; Stage [Nymph 5 (= 5) or Adult (= 6)]; Sex (1 = females, 2 = males); “NA“ indicates data not applicable or not available for the given combination.

(Part 6)

Dens	Replic	Stage	Sex	N dead fed D3	N dead fed 3 Days	Prop dead fed D1	Prop dead fed D2	Prop dead fed D3
10	1	5	NA	0	0	0	0	0
10	2	5	NA	0	0	0	0	0
10	3	5	NA	0	0	0	0	0
10	4	5	NA	0	1	0	0.1	0
20	1	5	NA	2	4	0	0.1	0.11
20	2	5	NA	3	7	0	0.2	0.19
20	3	5	NA	5	9	0.1	0.11	0.31
20	4	5	NA	3	4	0	0.05	0.16
30	1	5	NA	1	8	0.1	0.15	0.04
30	2	5	NA	2	13	0.13	0.27	0.11
30	3	5	NA	2	6	0	0.13	0.08
30	4	5	NA	4	6	0	0.07	0.14
40	1	5	NA	7	13	0	0.15	0.21
40	2	5	NA	4	14	0.05	0.21	0.13
40	3	5	NA	9	14	0.03	0.1	0.26
40	4	5	NA	3	8	0.08	0.05	0.09
40	5	5	NA	1	3	0	0.05	0.03
60	1	5	NA	4	6	0	0.03	0.07
10	1	6	NA	0	0	0	0	0
10	2	6	NA	0	0	0	0	0
10	3	6	NA	0	0	0	0	0
10	4	6	NA	0	0	0	0	0
20	1	6	NA	3	3	0	0	0.15
20	2	6	NA	0	0	0	0	0
20	3	6	NA	0	0	0	0	0
20	4	6	NA	0	2	0	0.1	0
30	1	6	NA	1	2	0	0.03	0.03
30	2	6	NA	2	7	0.03	0.13	0.07
30	3	6	NA	0	2	0.03	0.03	0
30	4	6	NA	1	6	0	0.17	0.03
40	1	6	NA	2	12	0.23	0.03	0.05
40	2	6	NA	1	1	0	0	0.03
40	3	6	NA	1	3	0	0.05	0.03
40	4	6	NA	0	3	0	0.08	0

60	1	6	NA	3	8	0.02	0.07	0.05
10	1	6	1	NA	NA	NA	NA	NA
10	2	6	1	NA	NA	NA	NA	NA
10	3	6	1	NA	NA	NA	NA	NA
10	4	6	1	NA	NA	NA	NA	NA
20	1	6	1	NA	NA	NA	NA	NA
20	2	6	1	NA	NA	NA	NA	NA
20	3	6	1	NA	NA	NA	NA	NA
20	4	6	1	NA	NA	NA	NA	NA
30	1	6	1	NA	NA	NA	NA	NA
30	2	6	1	NA	NA	NA	NA	NA
30	3	6	1	NA	NA	NA	NA	NA
30	4	6	1	NA	NA	NA	NA	NA
40	1	6	1	NA	NA	NA	NA	NA
40	2	6	1	NA	NA	NA	NA	NA
40	3	6	1	NA	NA	NA	NA	NA
40	4	6	1	NA	NA	NA	NA	NA
60	1	6	1	NA	NA	NA	NA	NA
10	1	6	2	NA	NA	NA	NA	NA
10	2	6	2	NA	NA	NA	NA	NA
10	3	6	2	NA	NA	NA	NA	NA
10	4	6	2	NA	NA	NA	NA	NA
20	1	6	2	NA	NA	NA	NA	NA
20	2	6	2	NA	NA	NA	NA	NA
20	3	6	2	NA	NA	NA	NA	NA
20	4	6	2	NA	NA	NA	NA	NA
30	1	6	2	NA	NA	NA	NA	NA
30	2	6	2	NA	NA	NA	NA	NA
30	3	6	2	NA	NA	NA	NA	NA
30	4	6	2	NA	NA	NA	NA	NA
40	1	6	2	NA	NA	NA	NA	NA
40	2	6	2	NA	NA	NA	NA	NA
40	3	6	2	NA	NA	NA	NA	NA
40	4	6	2	NA	NA	NA	NA	NA
60	1	6	2	NA	NA	NA	NA	NA
10	1	6	NA	NA	NA	NA	NA	NA
10	2	6	NA	NA	NA	NA	NA	NA

10	3	6	NA	NA	NA	NA	NA	NA
10	4	6	NA	NA	NA	NA	NA	NA
20	1	6	NA	NA	NA	NA	NA	NA
20	2	6	NA	NA	NA	NA	NA	NA
20	3	6	NA	NA	NA	NA	NA	NA
20	4	6	NA	NA	NA	NA	NA	NA
30	1	6	NA	NA	NA	NA	NA	NA
30	2	6	NA	NA	NA	NA	NA	NA
30	3	6	NA	NA	NA	NA	NA	NA
30	4	6	NA	NA	NA	NA	NA	NA
40	1	6	NA	NA	NA	NA	NA	NA
40	2	6	NA	NA	NA	NA	NA	NA
40	3	6	NA	NA	NA	NA	NA	NA
40	4	6	NA	NA	NA	NA	NA	NA
60	1	6	NA	NA	NA	NA	NA	NA
10	1	5	NA	NA	NA	NA	NA	NA
10	1	5	NA	NA	NA	NA	NA	NA
10	2	5	NA	NA	NA	NA	NA	NA
10	3	5	NA	NA	NA	NA	NA	NA
10	4	5	NA	NA	NA	NA	NA	NA
20	1	5	NA	NA	NA	NA	NA	NA
20	2	5	NA	NA	NA	NA	NA	NA
20	3	5	NA	NA	NA	NA	NA	NA
20	4	5	NA	NA	NA	NA	NA	NA
30	1	5	NA	NA	NA	NA	NA	NA
30	2	5	NA	NA	NA	NA	NA	NA
30	3	5	NA	NA	NA	NA	NA	NA
30	4	5	NA	NA	NA	NA	NA	NA
40	1	5	NA	NA	NA	NA	NA	NA
40	2	5	NA	NA	NA	NA	NA	NA
40	3	5	NA	NA	NA	NA	NA	NA
40	4	5	NA	NA	NA	NA	NA	NA
60	1	5	NA	NA	NA	NA	NA	NA
10	1	6	NA	NA	NA	NA	NA	NA
10	2	6	NA	NA	NA	NA	NA	NA
10	3	6	NA	NA	NA	NA	NA	NA
10	4	6	NA	NA	NA	NA	NA	NA

20	1	6	NA	NA	NA	NA	NA	NA
20	2	6	NA	NA	NA	NA	NA	NA
20	3	6	NA	NA	NA	NA	NA	NA
20	4	6	NA	NA	NA	NA	NA	NA
30	1	6	NA	NA	NA	NA	NA	NA
30	2	6	NA	NA	NA	NA	NA	NA
30	3	6	NA	NA	NA	NA	NA	NA
30	4	6	NA	NA	NA	NA	NA	NA
40	1	6	NA	NA	NA	NA	NA	NA
40	2	6	NA	NA	NA	NA	NA	NA
40	3	6	NA	NA	NA	NA	NA	NA
40	4	6	NA	NA	NA	NA	NA	NA
60	1	6	NA	NA	NA	NA	NA	NA
10	1	5	NA	NA	NA	NA	NA	NA
10	2	5	NA	NA	NA	NA	NA	NA
10	3	5	NA	NA	NA	NA	NA	NA
10	4	5	NA	NA	NA	NA	NA	NA
20	1	5	NA	NA	NA	NA	NA	NA
20	2	5	NA	NA	NA	NA	NA	NA
20	3	5	NA	NA	NA	NA	NA	NA
20	4	5	NA	NA	NA	NA	NA	NA
30	1	5	NA	NA	NA	NA	NA	NA
30	2	5	NA	NA	NA	NA	NA	NA
30	3	5	NA	NA	NA	NA	NA	NA
30	4	5	NA	NA	NA	NA	NA	NA
40	1	5	NA	NA	NA	NA	NA	NA
40	2	5	NA	NA	NA	NA	NA	NA
40	3	5	NA	NA	NA	NA	NA	NA
40	4	5	NA	NA	NA	NA	NA	NA
40	5	5	NA	NA	NA	NA	NA	NA
60	1	5	NA	NA	NA	NA	NA	NA
10	1	6	1	NA	NA	NA	NA	NA
10	2	6	1	NA	NA	NA	NA	NA
10	3	6	1	NA	NA	NA	NA	NA
10	4	6	1	NA	NA	NA	NA	NA
20	1	6	1	NA	NA	NA	NA	NA
20	2	6	1	NA	NA	NA	NA	NA

20	3	6	1	NA	NA	NA	NA	NA
20	4	6	1	NA	NA	NA	NA	NA
30	1	6	1	NA	NA	NA	NA	NA
30	2	6	1	NA	NA	NA	NA	NA
30	3	6	1	NA	NA	NA	NA	NA
30	4	6	1	NA	NA	NA	NA	NA
40	1	6	1	NA	NA	NA	NA	NA
40	2	6	1	NA	NA	NA	NA	NA
40	3	6	1	NA	NA	NA	NA	NA
40	4	6	1	NA	NA	NA	NA	NA
60	1	6	1	NA	NA	NA	NA	NA
10	1	5	NA	NA	NA	NA	NA	NA
10	2	5	NA	NA	NA	NA	NA	NA
10	3	5	NA	NA	NA	NA	NA	NA
10	4	5	NA	NA	NA	NA	NA	NA
20	1	5	NA	NA	NA	NA	NA	NA
20	2	5	NA	NA	NA	NA	NA	NA
20	3	5	NA	NA	NA	NA	NA	NA
20	4	5	NA	NA	NA	NA	NA	NA
30	1	5	NA	NA	NA	NA	NA	NA
30	2	5	NA	NA	NA	NA	NA	NA
30	3	5	NA	NA	NA	NA	NA	NA
30	4	5	NA	NA	NA	NA	NA	NA
40	1	5	NA	NA	NA	NA	NA	NA
40	2	5	NA	NA	NA	NA	NA	NA
40	3	5	NA	NA	NA	NA	NA	NA
40	4	5	NA	NA	NA	NA	NA	NA
40	5	5	NA	NA	NA	NA	NA	NA
60	1	5	NA	NA	NA	NA	NA	NA
10	1	5	NA	NA	NA	NA	NA	NA
10	2	5	NA	NA	NA	NA	NA	NA
10	3	5	NA	NA	NA	NA	NA	NA
10	4	5	NA	NA	NA	NA	NA	NA
20	1	5	NA	NA	NA	NA	NA	NA
20	2	5	NA	NA	NA	NA	NA	NA
20	3	5	NA	NA	NA	NA	NA	NA
20	4	5	NA	NA	NA	NA	NA	NA

30	1	5	NA	NA	NA	NA	NA	NA
30	2	5	NA	NA	NA	NA	NA	NA
30	3	5	NA	NA	NA	NA	NA	NA
30	4	5	NA	NA	NA	NA	NA	NA
40	1	5	NA	NA	NA	NA	NA	NA
40	2	5	NA	NA	NA	NA	NA	NA
40	3	5	NA	NA	NA	NA	NA	NA
40	4	5	NA	NA	NA	NA	NA	NA
40	5	5	NA	NA	NA	NA	NA	NA
60	1	5	NA	NA	NA	NA	NA	NA

Dens: density; Replic: replicate; Stage [Nymph 5 (= 5) or Adult (= 6)]; Sex (1 = females, 2 = males); “NA“ indicates data not applicable or not available for the given combination.

(Part 7)

Dens	Replic	Stage	Sex	Mean prop dead fed 3 Days	Sd prop dead fed 3 Days	Mean Ad Longevity	Sd Ad longevity	N moved Day1
10	1	5	NA	0	0	NA	NA	NA
10	2	5	NA	0	0	NA	NA	NA
10	3	5	NA	0	0	NA	NA	NA
10	4	5	NA	0.03	0.058	NA	NA	NA
20	1	5	NA	0.07	0.061	NA	NA	NA
20	2	5	NA	0.13	0.112	NA	NA	NA
20	3	5	NA	0.18	0.12	NA	NA	NA
20	4	5	NA	0.07	0.081	NA	NA	NA
30	1	5	NA	0.1	0.052	NA	NA	NA
30	2	5	NA	0.17	0.088	NA	NA	NA
30	3	5	NA	0.07	0.067	NA	NA	NA
30	4	5	NA	0.07	0.071	NA	NA	NA
40	1	5	NA	0.12	0.106	NA	NA	NA
40	2	5	NA	0.13	0.08	NA	NA	NA
40	3	5	NA	0.13	0.118	NA	NA	NA
40	4	5	NA	0.07	0.016	NA	NA	NA
40	5	5	NA	0.03	0.025	NA	NA	NA
60	1	5	NA	0.03	0.034	NA	NA	NA
10	1	6	NA	0	0	26.8	15.3	NA
10	2	6	NA	0	0	35.2	10.4	NA
10	3	6	NA	0	0	33.8	10.6	NA
10	4	6	NA	0	0	27.2	17.7	NA
20	1	6	NA	0.05	0.087	36.3	17.5	NA
20	2	6	NA	0	0	28.6	15.1	NA
20	3	6	NA	0	0	33.9	7.9	NA
20	4	6	NA	0.033	0.058	24.4	15.6	NA
30	1	6	NA	0.022	0.019	34	14.9	NA
30	2	6	NA	0.078	0.051	26.8	14	NA
30	3	6	NA	0.022	0.019	38.6	13.9	NA
30	4	6	NA	0.067	0.088	31.7	11	NA
40	1	6	NA	0.1	0.109	20.6	7	NA
40	2	6	NA	0.008	0.014	19.9	7.6	NA
40	3	6	NA	0.025	0.025	21	6.8	NA

40	4	6	NA	0.025	0.043	33	10.6	NA
60	1	6	NA	0.044	0.025	24.5	13.4	NA
10	1	6	1	NA	NA	NA	NA	2
10	2	6	1	NA	NA	NA	NA	2
10	3	6	1	NA	NA	NA	NA	1
10	4	6	1	NA	NA	NA	NA	1
20	1	6	1	NA	NA	NA	NA	7
20	2	6	1	NA	NA	NA	NA	2
20	3	6	1	NA	NA	NA	NA	2
20	4	6	1	NA	NA	NA	NA	3
30	1	6	1	NA	NA	NA	NA	10
30	2	6	1	NA	NA	NA	NA	6
30	3	6	1	NA	NA	NA	NA	2
30	4	6	1	NA	NA	NA	NA	5
40	1	6	1	NA	NA	NA	NA	10
40	2	6	1	NA	NA	NA	NA	9
40	3	6	1	NA	NA	NA	NA	6
40	4	6	1	NA	NA	NA	NA	8
60	1	6	1	NA	NA	NA	NA	7
10	1	6	2	NA	NA	NA	NA	4
10	2	6	2	NA	NA	NA	NA	1
10	3	6	2	NA	NA	NA	NA	4
10	4	6	2	NA	NA	NA	NA	1
20	1	6	2	NA	NA	NA	NA	5
20	2	6	2	NA	NA	NA	NA	1
20	3	6	2	NA	NA	NA	NA	4
20	4	6	2	NA	NA	NA	NA	1
30	1	6	2	NA	NA	NA	NA	6
30	2	6	2	NA	NA	NA	NA	3
30	3	6	2	NA	NA	NA	NA	8
30	4	6	2	NA	NA	NA	NA	5
40	1	6	2	NA	NA	NA	NA	13
40	2	6	2	NA	NA	NA	NA	10
40	3	6	2	NA	NA	NA	NA	11
40	4	6	2	NA	NA	NA	NA	7
60	1	6	2	NA	NA	NA	NA	5
10	1	6	NA	NA	NA	NA	NA	6

10	2	6	NA	NA	NA	NA	NA	3
10	3	6	NA	NA	NA	NA	NA	5
10	4	6	NA	NA	NA	NA	NA	2
20	1	6	NA	NA	NA	NA	NA	12
20	2	6	NA	NA	NA	NA	NA	3
20	3	6	NA	NA	NA	NA	NA	6
20	4	6	NA	NA	NA	NA	NA	7
30	1	6	NA	NA	NA	NA	NA	16
30	2	6	NA	NA	NA	NA	NA	9
30	3	6	NA	NA	NA	NA	NA	10
30	4	6	NA	NA	NA	NA	NA	10
40	1	6	NA	NA	NA	NA	NA	23
40	2	6	NA	NA	NA	NA	NA	19
40	3	6	NA	NA	NA	NA	NA	17
40	4	6	NA	NA	NA	NA	NA	15
60	1	6	NA	NA	NA	NA	NA	12
10	1	5	NA	NA	NA	NA	NA	1
10	1	5	NA	NA	NA	NA	NA	2
10	2	5	NA	NA	NA	NA	NA	2
10	3	5	NA	NA	NA	NA	NA	2
10	4	5	NA	NA	NA	NA	NA	5
20	1	5	NA	NA	NA	NA	NA	9
20	2	5	NA	NA	NA	NA	NA	5
20	3	5	NA	NA	NA	NA	NA	4
20	4	5	NA	NA	NA	NA	NA	12
30	1	5	NA	NA	NA	NA	NA	14
30	2	5	NA	NA	NA	NA	NA	6
30	3	5	NA	NA	NA	NA	NA	4
30	4	5	NA	NA	NA	NA	NA	10
40	1	5	NA	NA	NA	NA	NA	7
40	2	5	NA	NA	NA	NA	NA	13
40	3	5	NA	NA	NA	NA	NA	10
40	4	5	NA	NA	NA	NA	NA	7
60	1	5	NA	NA	NA	NA	NA	10
10	1	6	NA	NA	NA	NA	NA	NA
10	2	6	NA	NA	NA	NA	NA	NA
10	3	6	NA	NA	NA	NA	NA	NA

10	4	6	NA	NA	NA	NA	NA	NA
20	1	6	NA	NA	NA	NA	NA	NA
20	2	6	NA	NA	NA	NA	NA	NA
20	3	6	NA	NA	NA	NA	NA	NA
20	4	6	NA	NA	NA	NA	NA	NA
30	1	6	NA	NA	NA	NA	NA	NA
30	2	6	NA	NA	NA	NA	NA	NA
30	3	6	NA	NA	NA	NA	NA	NA
30	4	6	NA	NA	NA	NA	NA	NA
40	1	6	NA	NA	NA	NA	NA	NA
40	2	6	NA	NA	NA	NA	NA	NA
40	3	6	NA	NA	NA	NA	NA	NA
40	4	6	NA	NA	NA	NA	NA	NA
60	1	6	NA	NA	NA	NA	NA	NA
10	1	5	NA	NA	NA	NA	NA	NA
10	2	5	NA	NA	NA	NA	NA	NA
10	3	5	NA	NA	NA	NA	NA	NA
10	4	5	NA	NA	NA	NA	NA	NA
20	1	5	NA	NA	NA	NA	NA	NA
20	2	5	NA	NA	NA	NA	NA	NA
20	3	5	NA	NA	NA	NA	NA	NA
20	4	5	NA	NA	NA	NA	NA	NA
30	1	5	NA	NA	NA	NA	NA	NA
30	2	5	NA	NA	NA	NA	NA	NA
30	3	5	NA	NA	NA	NA	NA	NA
30	4	5	NA	NA	NA	NA	NA	NA
40	1	5	NA	NA	NA	NA	NA	NA
40	2	5	NA	NA	NA	NA	NA	NA
40	3	5	NA	NA	NA	NA	NA	NA
40	4	5	NA	NA	NA	NA	NA	NA
40	5	5	NA	NA	NA	NA	NA	NA
60	1	5	NA	NA	NA	NA	NA	NA
10	1	6	1	NA	NA	NA	NA	NA
10	2	6	1	NA	NA	NA	NA	NA
10	3	6	1	NA	NA	NA	NA	NA
10	4	6	1	NA	NA	NA	NA	NA
20	1	6	1	NA	NA	NA	NA	NA

20	2	6	1	NA	NA	NA	NA	NA
20	3	6	1	NA	NA	NA	NA	NA
20	4	6	1	NA	NA	NA	NA	NA
30	1	6	1	NA	NA	NA	NA	NA
30	2	6	1	NA	NA	NA	NA	NA
30	3	6	1	NA	NA	NA	NA	NA
30	4	6	1	NA	NA	NA	NA	NA
40	1	6	1	NA	NA	NA	NA	NA
40	2	6	1	NA	NA	NA	NA	NA
40	3	6	1	NA	NA	NA	NA	NA
40	4	6	1	NA	NA	NA	NA	NA
60	1	6	1	NA	NA	NA	NA	NA
10	1	5	NA	NA	NA	NA	NA	NA
10	2	5	NA	NA	NA	NA	NA	NA
10	3	5	NA	NA	NA	NA	NA	NA
10	4	5	NA	NA	NA	NA	NA	NA
20	1	5	NA	NA	NA	NA	NA	NA
20	2	5	NA	NA	NA	NA	NA	NA
20	3	5	NA	NA	NA	NA	NA	NA
20	4	5	NA	NA	NA	NA	NA	NA
30	1	5	NA	NA	NA	NA	NA	NA
30	2	5	NA	NA	NA	NA	NA	NA
30	3	5	NA	NA	NA	NA	NA	NA
30	4	5	NA	NA	NA	NA	NA	NA
40	1	5	NA	NA	NA	NA	NA	NA
40	2	5	NA	NA	NA	NA	NA	NA
40	3	5	NA	NA	NA	NA	NA	NA
40	4	5	NA	NA	NA	NA	NA	NA
40	5	5	NA	NA	NA	NA	NA	NA
60	1	5	NA	NA	NA	NA	NA	NA
10	1	5	NA	NA	NA	NA	NA	NA
10	2	5	NA	NA	NA	NA	NA	NA
10	3	5	NA	NA	NA	NA	NA	NA
10	4	5	NA	NA	NA	NA	NA	NA
20	1	5	NA	NA	NA	NA	NA	NA
20	2	5	NA	NA	NA	NA	NA	NA
20	3	5	NA	NA	NA	NA	NA	NA

20	4	5	NA	NA	NA	NA	NA	NA
30	1	5	NA	NA	NA	NA	NA	NA
30	2	5	NA	NA	NA	NA	NA	NA
30	3	5	NA	NA	NA	NA	NA	NA
30	4	5	NA	NA	NA	NA	NA	NA
40	1	5	NA	NA	NA	NA	NA	NA
40	2	5	NA	NA	NA	NA	NA	NA
40	3	5	NA	NA	NA	NA	NA	NA
40	4	5	NA	NA	NA	NA	NA	NA
40	5	5	NA	NA	NA	NA	NA	NA
60	1	5	NA	NA	NA	NA	NA	NA

Dens: density; Replic: replicate; Stage [Nymph 5 (= 5) or Adult (= 6)]; Sex (1 = females, 2 = males); “NA“ indicates data not applicable or not available for the given combination.

(Part 8)

Dens	Replic	Stage	Sex	N moved Day2	N moved Day3	N moved 3 Days	N fed moved Day1	N fed moved Day2
10	1	5	NA	NA	NA	NA	NA	NA
10	2	5	NA	NA	NA	NA	NA	NA
10	3	5	NA	NA	NA	NA	NA	NA
10	4	5	NA	NA	NA	NA	NA	NA
20	1	5	NA	NA	NA	NA	NA	NA
20	2	5	NA	NA	NA	NA	NA	NA
20	3	5	NA	NA	NA	NA	NA	NA
20	4	5	NA	NA	NA	NA	NA	NA
30	1	5	NA	NA	NA	NA	NA	NA
30	2	5	NA	NA	NA	NA	NA	NA
30	3	5	NA	NA	NA	NA	NA	NA
30	4	5	NA	NA	NA	NA	NA	NA
40	1	5	NA	NA	NA	NA	NA	NA
40	2	5	NA	NA	NA	NA	NA	NA
40	3	5	NA	NA	NA	NA	NA	NA
40	4	5	NA	NA	NA	NA	NA	NA
40	5	5	NA	NA	NA	NA	NA	NA
60	1	5	NA	NA	NA	NA	NA	NA
10	1	6	NA	NA	NA	NA	NA	NA
10	2	6	NA	NA	NA	NA	NA	NA
10	3	6	NA	NA	NA	NA	NA	NA
10	4	6	NA	NA	NA	NA	NA	NA
20	1	6	NA	NA	NA	NA	NA	NA
20	2	6	NA	NA	NA	NA	NA	NA
20	3	6	NA	NA	NA	NA	NA	NA
20	4	6	NA	NA	NA	NA	NA	NA
30	1	6	NA	NA	NA	NA	NA	NA
30	2	6	NA	NA	NA	NA	NA	NA
30	3	6	NA	NA	NA	NA	NA	NA
30	4	6	NA	NA	NA	NA	NA	NA
40	1	6	NA	NA	NA	NA	NA	NA
40	2	6	NA	NA	NA	NA	NA	NA
40	3	6	NA	NA	NA	NA	NA	NA
40	4	6	NA	NA	NA	NA	NA	NA

60	1	6	NA	NA	NA	NA	NA	NA
10	1	6	1	2	4	8	1	2
10	2	6	1	1	3	6	2	1
10	3	6	1	1	3	5	0	1
10	4	6	1	0	1	2	1	0
20	1	6	1	0	1	8	6	0
20	2	6	1	3	5	10	1	1
20	3	6	1	1	2	5	0	1
20	4	6	1	1	3	7	2	0
30	1	6	1	4	5	19	2	2
30	2	6	1	2	2	10	5	1
30	3	6	1	4	1	7	0	3
30	4	6	1	2	1	8	1	3
40	1	6	1	6	5	21	2	4
40	2	6	1	4	6	19	3	3
40	3	6	1	9	3	18	0	7
40	4	6	1	5	3	16	0	4
60	1	6	1	5	3	15	1	2
10	1	6	2	1	2	7	4	1
10	2	6	2	2	4	7	1	2
10	3	6	2	1	1	6	2	1
10	4	6	2	1	1	3	0	0
20	1	6	2	2	5	12	4	2
20	2	6	2	2	2	5	0	2
20	3	6	2	0	1	5	3	0
20	4	6	2	1	3	5	1	1
30	1	6	2	3	3	12	3	2
30	2	6	2	4	2	9	1	2
30	3	6	2	1	1	10	4	1
30	4	6	2	2	1	8	4	1
40	1	6	2	7	3	23	5	6
40	2	6	2	5	4	19	3	3
40	3	6	2	11	3	25	8	10
40	4	6	2	2	5	14	4	2
60	1	6	2	5	6	16	2	4
10	1	6	NA	3	6	15	5	3
10	2	6	NA	3	7	13	3	3

10	3	6	NA	2	4	11	2	2
10	4	6	NA	1	2	5	1	0
20	1	6	NA	2	6	20	10	2
20	2	6	NA	5	7	15	1	3
20	3	6	NA	1	3	10	3	1
20	4	6	NA	2	6	15	3	1
30	1	6	NA	7	8	31	5	4
30	2	6	NA	6	4	19	6	3
30	3	6	NA	5	2	17	4	4
30	4	6	NA	5	1	16	5	4
40	1	6	NA	13	8	44	7	10
40	2	6	NA	9	10	38	6	6
40	3	6	NA	20	6	43	8	17
40	4	6	NA	7	8	30	4	6
60	1	6	NA	10	9	31	3	6
10	1	5	NA	0	3	4	0	0
10	1	5	NA	1	1	4	0	1
10	2	5	NA	5	3	10	0	4
10	3	5	NA	3	1	6	1	3
10	4	5	NA	5	3	13	2	2
20	1	5	NA	5	2	16	6	3
20	2	5	NA	4	4	13	4	4
20	3	5	NA	3	3	10	2	2
20	4	5	NA	4	4	20	7	4
30	1	5	NA	6	3	23	5	3
30	2	5	NA	2	5	13	3	1
30	3	5	NA	4	5	13	4	3
30	4	5	NA	9	10	29	3	8
40	1	5	NA	7	3	17	3	5
40	2	5	NA	10	10	33	8	10
40	3	5	NA	6	3	19	7	4
40	4	5	NA	2	2	11	2	1
60	1	5	NA	14	5	29	8	10
10	1	6	NA	NA	NA	NA	NA	NA
10	2	6	NA	NA	NA	NA	NA	NA
10	3	6	NA	NA	NA	NA	NA	NA
10	4	6	NA	NA	NA	NA	NA	NA

20	1	6	NA	NA	NA	NA	NA	NA
20	2	6	NA	NA	NA	NA	NA	NA
20	3	6	NA	NA	NA	NA	NA	NA
20	4	6	NA	NA	NA	NA	NA	NA
30	1	6	NA	NA	NA	NA	NA	NA
30	2	6	NA	NA	NA	NA	NA	NA
30	3	6	NA	NA	NA	NA	NA	NA
30	4	6	NA	NA	NA	NA	NA	NA
40	1	6	NA	NA	NA	NA	NA	NA
40	2	6	NA	NA	NA	NA	NA	NA
40	3	6	NA	NA	NA	NA	NA	NA
40	4	6	NA	NA	NA	NA	NA	NA
60	1	6	NA	NA	NA	NA	NA	NA
10	1	5	NA	NA	NA	NA	NA	NA
10	2	5	NA	NA	NA	NA	NA	NA
10	3	5	NA	NA	NA	NA	NA	NA
10	4	5	NA	NA	NA	NA	NA	NA
20	1	5	NA	NA	NA	NA	NA	NA
20	2	5	NA	NA	NA	NA	NA	NA
20	3	5	NA	NA	NA	NA	NA	NA
20	4	5	NA	NA	NA	NA	NA	NA
30	1	5	NA	NA	NA	NA	NA	NA
30	2	5	NA	NA	NA	NA	NA	NA
30	3	5	NA	NA	NA	NA	NA	NA
30	4	5	NA	NA	NA	NA	NA	NA
40	1	5	NA	NA	NA	NA	NA	NA
40	2	5	NA	NA	NA	NA	NA	NA
40	3	5	NA	NA	NA	NA	NA	NA
40	4	5	NA	NA	NA	NA	NA	NA
40	5	5	NA	NA	NA	NA	NA	NA
60	1	5	NA	NA	NA	NA	NA	NA
10	1	6	1	NA	NA	NA	NA	NA
10	2	6	1	NA	NA	NA	NA	NA
10	3	6	1	NA	NA	NA	NA	NA
10	4	6	1	NA	NA	NA	NA	NA
20	1	6	1	NA	NA	NA	NA	NA
20	2	6	1	NA	NA	NA	NA	NA

20	3	6	1	NA	NA	NA	NA	NA
20	4	6	1	NA	NA	NA	NA	NA
30	1	6	1	NA	NA	NA	NA	NA
30	2	6	1	NA	NA	NA	NA	NA
30	3	6	1	NA	NA	NA	NA	NA
30	4	6	1	NA	NA	NA	NA	NA
40	1	6	1	NA	NA	NA	NA	NA
40	2	6	1	NA	NA	NA	NA	NA
40	3	6	1	NA	NA	NA	NA	NA
40	4	6	1	NA	NA	NA	NA	NA
60	1	6	1	NA	NA	NA	NA	NA
10	1	5	NA	NA	NA	NA	NA	NA
10	2	5	NA	NA	NA	NA	NA	NA
10	3	5	NA	NA	NA	NA	NA	NA
10	4	5	NA	NA	NA	NA	NA	NA
20	1	5	NA	NA	NA	NA	NA	NA
20	2	5	NA	NA	NA	NA	NA	NA
20	3	5	NA	NA	NA	NA	NA	NA
20	4	5	NA	NA	NA	NA	NA	NA
30	1	5	NA	NA	NA	NA	NA	NA
30	2	5	NA	NA	NA	NA	NA	NA
30	3	5	NA	NA	NA	NA	NA	NA
30	4	5	NA	NA	NA	NA	NA	NA
40	1	5	NA	NA	NA	NA	NA	NA
40	2	5	NA	NA	NA	NA	NA	NA
40	3	5	NA	NA	NA	NA	NA	NA
40	4	5	NA	NA	NA	NA	NA	NA
40	5	5	NA	NA	NA	NA	NA	NA
60	1	5	NA	NA	NA	NA	NA	NA
10	1	5	NA	NA	NA	NA	NA	NA
10	2	5	NA	NA	NA	NA	NA	NA
10	3	5	NA	NA	NA	NA	NA	NA
10	4	5	NA	NA	NA	NA	NA	NA
20	1	5	NA	NA	NA	NA	NA	NA
20	2	5	NA	NA	NA	NA	NA	NA
20	3	5	NA	NA	NA	NA	NA	NA
20	4	5	NA	NA	NA	NA	NA	NA

30	1	5	NA	NA	NA	NA	NA	NA
30	2	5	NA	NA	NA	NA	NA	NA
30	3	5	NA	NA	NA	NA	NA	NA
30	4	5	NA	NA	NA	NA	NA	NA
40	1	5	NA	NA	NA	NA	NA	NA
40	2	5	NA	NA	NA	NA	NA	NA
40	3	5	NA	NA	NA	NA	NA	NA
40	4	5	NA	NA	NA	NA	NA	NA
40	5	5	NA	NA	NA	NA	NA	NA
60	1	5	NA	NA	NA	NA	NA	NA

Dens: density; Replic: replicate; Stage [Nymph 5 (= 5) or Adult (= 6)]; Sex (1 = females, 2 = males); “NA“ indicates data not applicable or not available for the given combination.

(Part 9)

Dens	Replic	Stage	Sex	N moved Day2	N moved Day3	N moved 3 Days	N fed moved Day1	N fed moved Day2
10	1	5	NA	NA	NA	NA	NA	NA
10	2	5	NA	NA	NA	NA	NA	NA
10	3	5	NA	NA	NA	NA	NA	NA
10	4	5	NA	NA	NA	NA	NA	NA
20	1	5	NA	NA	NA	NA	NA	NA
20	2	5	NA	NA	NA	NA	NA	NA
20	3	5	NA	NA	NA	NA	NA	NA
20	4	5	NA	NA	NA	NA	NA	NA
30	1	5	NA	NA	NA	NA	NA	NA
30	2	5	NA	NA	NA	NA	NA	NA
30	3	5	NA	NA	NA	NA	NA	NA
30	4	5	NA	NA	NA	NA	NA	NA
40	1	5	NA	NA	NA	NA	NA	NA
40	2	5	NA	NA	NA	NA	NA	NA
40	3	5	NA	NA	NA	NA	NA	NA
40	4	5	NA	NA	NA	NA	NA	NA
40	5	5	NA	NA	NA	NA	NA	NA
60	1	5	NA	NA	NA	NA	NA	NA
10	1	6	NA	NA	NA	NA	NA	NA
10	2	6	NA	NA	NA	NA	NA	NA
10	3	6	NA	NA	NA	NA	NA	NA
10	4	6	NA	NA	NA	NA	NA	NA
20	1	6	NA	NA	NA	NA	NA	NA
20	2	6	NA	NA	NA	NA	NA	NA
20	3	6	NA	NA	NA	NA	NA	NA
20	4	6	NA	NA	NA	NA	NA	NA
30	1	6	NA	NA	NA	NA	NA	NA
30	2	6	NA	NA	NA	NA	NA	NA
30	3	6	NA	NA	NA	NA	NA	NA
30	4	6	NA	NA	NA	NA	NA	NA
40	1	6	NA	NA	NA	NA	NA	NA
40	2	6	NA	NA	NA	NA	NA	NA
40	3	6	NA	NA	NA	NA	NA	NA
40	4	6	NA	NA	NA	NA	NA	NA
60	1	6	NA	NA	NA	NA	NA	NA

10	1	6	1	2	4	8	1	2
10	2	6	1	1	3	6	2	1
10	3	6	1	1	3	5	0	1
10	4	6	1	0	1	2	1	0
20	1	6	1	0	1	8	6	0
20	2	6	1	3	5	10	1	1
20	3	6	1	1	2	5	0	1
20	4	6	1	1	3	7	2	0
30	1	6	1	4	5	19	2	2
30	2	6	1	2	2	10	5	1
30	3	6	1	4	1	7	0	3
30	4	6	1	2	1	8	1	3
40	1	6	1	6	5	21	2	4
40	2	6	1	4	6	19	3	3
40	3	6	1	9	3	18	0	7
40	4	6	1	5	3	16	0	4
60	1	6	1	5	3	15	1	2
10	1	6	2	1	2	7	4	1
10	2	6	2	2	4	7	1	2
10	3	6	2	1	1	6	2	1
10	4	6	2	1	1	3	0	0
20	1	6	2	2	5	12	4	2
20	2	6	2	2	2	5	0	2
20	3	6	2	0	1	5	3	0
20	4	6	2	1	3	5	1	1
30	1	6	2	3	3	12	3	2
30	2	6	2	4	2	9	1	2
30	3	6	2	1	1	10	4	1
30	4	6	2	2	1	8	4	1
40	1	6	2	7	3	23	5	6
40	2	6	2	5	4	19	3	3
40	3	6	2	11	3	25	8	10
40	4	6	2	2	5	14	4	2
60	1	6	2	5	6	16	2	4
10	1	6	NA	3	6	15	5	3
10	2	6	NA	3	7	13	3	3
10	3	6	NA	2	4	11	2	2

10	4	6	NA	1	2	5	1	0
20	1	6	NA	2	6	20	10	2
20	2	6	NA	5	7	15	1	3
20	3	6	NA	1	3	10	3	1
20	4	6	NA	2	6	15	3	1
30	1	6	NA	7	8	31	5	4
30	2	6	NA	6	4	19	6	3
30	3	6	NA	5	2	17	4	4
30	4	6	NA	5	1	16	5	4
40	1	6	NA	13	8	44	7	10
40	2	6	NA	9	10	38	6	6
40	3	6	NA	20	6	43	8	17
40	4	6	NA	7	8	30	4	6
60	1	6	NA	10	9	31	3	6
10	1	5	NA	0	3	4	0	0
10	1	5	NA	1	1	4	0	1
10	2	5	NA	5	3	10	0	4
10	3	5	NA	3	1	6	1	3
10	4	5	NA	5	3	13	2	2
20	1	5	NA	5	2	16	6	3
20	2	5	NA	4	4	13	4	4
20	3	5	NA	3	3	10	2	2
20	4	5	NA	4	4	20	7	4
30	1	5	NA	6	3	23	5	3
30	2	5	NA	2	5	13	3	1
30	3	5	NA	4	5	13	4	3
30	4	5	NA	9	10	29	3	8
40	1	5	NA	7	3	17	3	5
40	2	5	NA	10	10	33	8	10
40	3	5	NA	6	3	19	7	4
40	4	5	NA	2	2	11	2	1
60	1	5	NA	14	5	29	8	10
10	1	6	NA	NA	NA	NA	NA	NA
10	2	6	NA	NA	NA	NA	NA	NA
10	3	6	NA	NA	NA	NA	NA	NA
10	4	6	NA	NA	NA	NA	NA	NA
20	1	6	NA	NA	NA	NA	NA	NA

20	2	6	NA	NA	NA	NA	NA	NA
20	3	6	NA	NA	NA	NA	NA	NA
20	4	6	NA	NA	NA	NA	NA	NA
30	1	6	NA	NA	NA	NA	NA	NA
30	2	6	NA	NA	NA	NA	NA	NA
30	3	6	NA	NA	NA	NA	NA	NA
30	4	6	NA	NA	NA	NA	NA	NA
40	1	6	NA	NA	NA	NA	NA	NA
40	2	6	NA	NA	NA	NA	NA	NA
40	3	6	NA	NA	NA	NA	NA	NA
40	4	6	NA	NA	NA	NA	NA	NA
60	1	6	NA	NA	NA	NA	NA	NA
10	1	5	NA	NA	NA	NA	NA	NA
10	2	5	NA	NA	NA	NA	NA	NA
10	3	5	NA	NA	NA	NA	NA	NA
10	4	5	NA	NA	NA	NA	NA	NA
20	1	5	NA	NA	NA	NA	NA	NA
20	2	5	NA	NA	NA	NA	NA	NA
20	3	5	NA	NA	NA	NA	NA	NA
20	4	5	NA	NA	NA	NA	NA	NA
30	1	5	NA	NA	NA	NA	NA	NA
30	2	5	NA	NA	NA	NA	NA	NA
30	3	5	NA	NA	NA	NA	NA	NA
30	4	5	NA	NA	NA	NA	NA	NA
40	1	5	NA	NA	NA	NA	NA	NA
40	2	5	NA	NA	NA	NA	NA	NA
40	3	5	NA	NA	NA	NA	NA	NA
40	4	5	NA	NA	NA	NA	NA	NA
40	5	5	NA	NA	NA	NA	NA	NA
60	1	5	NA	NA	NA	NA	NA	NA
10	1	6	1	NA	NA	NA	NA	NA
10	2	6	1	NA	NA	NA	NA	NA
10	3	6	1	NA	NA	NA	NA	NA
10	4	6	1	NA	NA	NA	NA	NA
20	1	6	1	NA	NA	NA	NA	NA
20	2	6	1	NA	NA	NA	NA	NA
20	3	6	1	NA	NA	NA	NA	NA

20	4	6	1	NA	NA	NA	NA	NA
30	1	6	1	NA	NA	NA	NA	NA
30	2	6	1	NA	NA	NA	NA	NA
30	3	6	1	NA	NA	NA	NA	NA
30	4	6	1	NA	NA	NA	NA	NA
40	1	6	1	NA	NA	NA	NA	NA
40	2	6	1	NA	NA	NA	NA	NA
40	3	6	1	NA	NA	NA	NA	NA
40	4	6	1	NA	NA	NA	NA	NA
60	1	6	1	NA	NA	NA	NA	NA
10	1	5	NA	NA	NA	NA	NA	NA
10	2	5	NA	NA	NA	NA	NA	NA
10	3	5	NA	NA	NA	NA	NA	NA
10	4	5	NA	NA	NA	NA	NA	NA
20	1	5	NA	NA	NA	NA	NA	NA
20	2	5	NA	NA	NA	NA	NA	NA
20	3	5	NA	NA	NA	NA	NA	NA
20	4	5	NA	NA	NA	NA	NA	NA
30	1	5	NA	NA	NA	NA	NA	NA
30	2	5	NA	NA	NA	NA	NA	NA
30	3	5	NA	NA	NA	NA	NA	NA
30	4	5	NA	NA	NA	NA	NA	NA
40	1	5	NA	NA	NA	NA	NA	NA
40	2	5	NA	NA	NA	NA	NA	NA
40	3	5	NA	NA	NA	NA	NA	NA
40	4	5	NA	NA	NA	NA	NA	NA
40	5	5	NA	NA	NA	NA	NA	NA
60	1	5	NA	NA	NA	NA	NA	NA
10	1	5	NA	NA	NA	NA	NA	NA
10	2	5	NA	NA	NA	NA	NA	NA
10	3	5	NA	NA	NA	NA	NA	NA
10	4	5	NA	NA	NA	NA	NA	NA
20	1	5	NA	NA	NA	NA	NA	NA
20	2	5	NA	NA	NA	NA	NA	NA
20	3	5	NA	NA	NA	NA	NA	NA
20	4	5	NA	NA	NA	NA	NA	NA
30	1	5	NA	NA	NA	NA	NA	NA

30	2	5	NA	NA	NA	NA	NA	NA
30	3	5	NA	NA	NA	NA	NA	NA
30	4	5	NA	NA	NA	NA	NA	NA
40	1	5	NA	NA	NA	NA	NA	NA
40	2	5	NA	NA	NA	NA	NA	NA
40	3	5	NA	NA	NA	NA	NA	NA
40	4	5	NA	NA	NA	NA	NA	NA
40	5	5	NA	NA	NA	NA	NA	NA
60	1	5	NA	NA	NA	NA	NA	NA

Dens: density; Replic: replicate; Stage [Nymph 5 (= 5) or Adult (= 6)]; Sex (1 = females, 2 = males); “NA“ indicates data not applicable or not available for the given combination.

(Part 10)

Dens	Replic	Stage	Sex	N fed moved Day3	N fed moved 3 Days	Pct disp feeding on day 2 and fed 1 day before	Pct disp feeding on day 3 and fed 1 day before	Pct disp feeding on day 3 and fed 2 days before
10	1	5	NA	NA	NA	NA	NA	NA
10	2	5	NA	NA	NA	NA	NA	NA
10	3	5	NA	NA	NA	NA	NA	NA
10	4	5	NA	NA	NA	NA	NA	NA
20	1	5	NA	NA	NA	NA	NA	NA
20	2	5	NA	NA	NA	NA	NA	NA
20	3	5	NA	NA	NA	NA	NA	NA
20	4	5	NA	NA	NA	NA	NA	NA
30	1	5	NA	NA	NA	NA	NA	NA
30	2	5	NA	NA	NA	NA	NA	NA
30	3	5	NA	NA	NA	NA	NA	NA
30	4	5	NA	NA	NA	NA	NA	NA
40	1	5	NA	NA	NA	NA	NA	NA
40	2	5	NA	NA	NA	NA	NA	NA
40	3	5	NA	NA	NA	NA	NA	NA
40	4	5	NA	NA	NA	NA	NA	NA
40	5	5	NA	NA	NA	NA	NA	NA
60	1	5	NA	NA	NA	NA	NA	NA
10	1	6	NA	NA	NA	NA	NA	NA
10	2	6	NA	NA	NA	NA	NA	NA
10	3	6	NA	NA	NA	NA	NA	NA
10	4	6	NA	NA	NA	NA	NA	NA
20	1	6	NA	NA	NA	NA	NA	NA
20	2	6	NA	NA	NA	NA	NA	NA
20	3	6	NA	NA	NA	NA	NA	NA
20	4	6	NA	NA	NA	NA	NA	NA
30	1	6	NA	NA	NA	NA	NA	NA
30	2	6	NA	NA	NA	NA	NA	NA
30	3	6	NA	NA	NA	NA	NA	NA
30	4	6	NA	NA	NA	NA	NA	NA
40	1	6	NA	NA	NA	NA	NA	NA
40	2	6	NA	NA	NA	NA	NA	NA
40	3	6	NA	NA	NA	NA	NA	NA

40	4	6	NA	NA	NA	NA	NA	NA
60	1	6	NA	NA	NA	NA	NA	NA
10	1	6	1	4	7	33.3	16.7	50
10	2	6	1	3	6	20	0	60
10	3	6	1	3	4	0	40	20
10	4	6	1	1	2	0	0	33.3
20	1	6	1	1	7	0	0	12.5
20	2	6	1	3	5	12.5	0	37.5
20	3	6	1	1	2	10	0	10
20	4	6	1	2	4	0	14.3	14.3
30	1	6	1	5	9	8.3	16.7	8.3
30	2	6	1	2	8	9.1	0	11.1
30	3	6	1	1	4	33.3	0	11.1
30	4	6	1	0	4	25	0	0
40	1	6	1	4	10	26.7	6.7	20
40	2	6	1	6	12	16.7	3.9	19.6
40	3	6	1	2	9	20	7.2	7.2
40	4	6	1	3	7	12.5	7.1	14.3
60	1	6	1	3	6	11.1	14.3	7.1
10	1	6	2	2	7	25	0	50
10	2	6	2	4	7	40	0	60
10	3	6	2	1	4	20	20	0
10	4	6	2	1	1	0	0	50
20	1	6	2	5	11	25	0	57.1
20	2	6	2	2	4	33.3	0	16.7
20	3	6	2	1	4	0	11.1	0
20	4	6	2	3	5	11.1	11.1	22.2
30	1	6	2	1	6	7.2	0	7.1
30	2	6	2	2	5	14.3	0	16.7
30	3	6	2	1	6	9.1	0	10
30	4	6	2	1	6	8.3	0	9.1
40	1	6	2	2	13	18.8	7.2	7.2
40	2	6	2	3	9	10.5	5.9	11.8
40	3	6	2	3	21	52.9	6.3	12.5
40	4	6	2	5	11	13.3	6.7	20
60	1	6	2	4	10	11.8	13.4	6.7
10	1	6	NA	6	14	30	10	50

10	2	6	NA	7	13	30	0	60
10	3	6	NA	4	8	10	30	10
10	4	6	NA	2	3	0	0	20
20	1	6	NA	6	18	11	0	33.3
20	2	6	NA	5	9	21.4	0	28.6
20	3	6	NA	2	6	5.3	5.3	5.3
20	4	6	NA	5	9	5.9	11.8	17.6
30	1	6	NA	6	15	5.8	6.7	6.7
30	2	6	NA	4	13	12.5	0	14.3
30	3	6	NA	2	10	20	0	10.5
30	4	6	NA	1	10	16.7	0	4.6
40	1	6	NA	6	23	22.6	9.5	19.1
40	2	6	NA	9	21	21.4	6.5	22.9
40	3	6	NA	5	30	37.5	8.3	12.4
40	4	6	NA	8	18	13.3	6.5	16.1
60	1	6	NA	7	16	14.5	12.9	6.4
10	1	5	NA	2	2	0	0	10
10	1	5	NA	1	2	10	0	10
10	2	5	NA	3	7	40	0	30
10	3	5	NA	1	5	30	0	10
10	4	5	NA	3	7	16.5	0	33.3
20	1	5	NA	1	10	30	0	16.7
20	2	5	NA	4	12	40	16.7	50
20	3	5	NA	3	7	14.3	11.1	22.2
20	4	5	NA	4	15	21.5	0	33.3
30	1	5	NA	0	8	15.4	0	0
30	2	5	NA	5	9	5.3	7.1	21.4
30	3	5	NA	4	11	18.8	0	0
30	4	5	NA	10	21	34.7	5.3	26.3
40	1	5	NA	2	10	19.2	0	4.8
40	2	5	NA	9	27	26.6	0	40
40	3	5	NA	2	13	15.4	0	0
40	4	5	NA	2	5	2.9	0	6.3
60	1	5	NA	4	22	10.5	12.9	6.5
10	1	6	NA	NA	NA	NA	NA	NA
10	2	6	NA	NA	NA	NA	NA	NA
10	3	6	NA	NA	NA	NA	NA	NA

10	4	6	NA	NA	NA	NA	NA	NA
20	1	6	NA	NA	NA	NA	NA	NA
20	2	6	NA	NA	NA	NA	NA	NA
20	3	6	NA	NA	NA	NA	NA	NA
20	4	6	NA	NA	NA	NA	NA	NA
30	1	6	NA	NA	NA	NA	NA	NA
30	2	6	NA	NA	NA	NA	NA	NA
30	3	6	NA	NA	NA	NA	NA	NA
30	4	6	NA	NA	NA	NA	NA	NA
40	1	6	NA	NA	NA	NA	NA	NA
40	2	6	NA	NA	NA	NA	NA	NA
40	3	6	NA	NA	NA	NA	NA	NA
40	4	6	NA	NA	NA	NA	NA	NA
60	1	6	NA	NA	NA	NA	NA	NA
10	1	5	NA	NA	NA	NA	NA	NA
10	2	5	NA	NA	NA	NA	NA	NA
10	3	5	NA	NA	NA	NA	NA	NA
10	4	5	NA	NA	NA	NA	NA	NA
20	1	5	NA	NA	NA	NA	NA	NA
20	2	5	NA	NA	NA	NA	NA	NA
20	3	5	NA	NA	NA	NA	NA	NA
20	4	5	NA	NA	NA	NA	NA	NA
30	1	5	NA	NA	NA	NA	NA	NA
30	2	5	NA	NA	NA	NA	NA	NA
30	3	5	NA	NA	NA	NA	NA	NA
30	4	5	NA	NA	NA	NA	NA	NA
40	1	5	NA	NA	NA	NA	NA	NA
40	2	5	NA	NA	NA	NA	NA	NA
40	3	5	NA	NA	NA	NA	NA	NA
40	4	5	NA	NA	NA	NA	NA	NA
40	5	5	NA	NA	NA	NA	NA	NA
60	1	5	NA	NA	NA	NA	NA	NA
10	1	6	1	NA	NA	NA	NA	NA
10	2	6	1	NA	NA	NA	NA	NA
10	3	6	1	NA	NA	NA	NA	NA
10	4	6	1	NA	NA	NA	NA	NA
20	1	6	1	NA	NA	NA	NA	NA

20	2	6	1	NA	NA	NA	NA	NA
20	3	6	1	NA	NA	NA	NA	NA
20	4	6	1	NA	NA	NA	NA	NA
30	1	6	1	NA	NA	NA	NA	NA
30	2	6	1	NA	NA	NA	NA	NA
30	3	6	1	NA	NA	NA	NA	NA
30	4	6	1	NA	NA	NA	NA	NA
40	1	6	1	NA	NA	NA	NA	NA
40	2	6	1	NA	NA	NA	NA	NA
40	3	6	1	NA	NA	NA	NA	NA
40	4	6	1	NA	NA	NA	NA	NA
60	1	6	1	NA	NA	NA	NA	NA
10	1	5	NA	NA	NA	NA	NA	NA
10	2	5	NA	NA	NA	NA	NA	NA
10	3	5	NA	NA	NA	NA	NA	NA
10	4	5	NA	NA	NA	NA	NA	NA
20	1	5	NA	NA	NA	NA	NA	NA
20	2	5	NA	NA	NA	NA	NA	NA
20	3	5	NA	NA	NA	NA	NA	NA
20	4	5	NA	NA	NA	NA	NA	NA
30	1	5	NA	NA	NA	NA	NA	NA
30	2	5	NA	NA	NA	NA	NA	NA
30	3	5	NA	NA	NA	NA	NA	NA
30	4	5	NA	NA	NA	NA	NA	NA
40	1	5	NA	NA	NA	NA	NA	NA
40	2	5	NA	NA	NA	NA	NA	NA
40	3	5	NA	NA	NA	NA	NA	NA
40	4	5	NA	NA	NA	NA	NA	NA
40	5	5	NA	NA	NA	NA	NA	NA
60	1	5	NA	NA	NA	NA	NA	NA
10	1	5	NA	NA	NA	NA	NA	NA
10	2	5	NA	NA	NA	NA	NA	NA
10	3	5	NA	NA	NA	NA	NA	NA
10	4	5	NA	NA	NA	NA	NA	NA
20	1	5	NA	NA	NA	NA	NA	NA
20	2	5	NA	NA	NA	NA	NA	NA
20	3	5	NA	NA	NA	NA	NA	NA

20	4	5	NA	NA	NA	NA	NA	NA
30	1	5	NA	NA	NA	NA	NA	NA
30	2	5	NA	NA	NA	NA	NA	NA
30	3	5	NA	NA	NA	NA	NA	NA
30	4	5	NA	NA	NA	NA	NA	NA
40	1	5	NA	NA	NA	NA	NA	NA
40	2	5	NA	NA	NA	NA	NA	NA
40	3	5	NA	NA	NA	NA	NA	NA
40	4	5	NA	NA	NA	NA	NA	NA
40	5	5	NA	NA	NA	NA	NA	NA
60	1	5	NA	NA	NA	NA	NA	NA

Dens: density; Replic: replicate; Stage [Nymph 5 (= 5) or Adult (= 6)]; Sex (1 = females, 2 = males); “NA“ indicates data not applicable or not available for the given combination.

(Part 11)

Dens	Replic	Stage	Sex	Pct disp feeding on day 3 and fed 1 and 2 days before	N feeding Day1	N feeding Day2	N feeding Day3	N feeding 3 Days
10	1	5	NA	NA	NA	NA	NA	NA
10	2	5	NA	NA	NA	NA	NA	NA
10	3	5	NA	NA	NA	NA	NA	NA
10	4	5	NA	NA	NA	NA	NA	NA
20	1	5	NA	NA	NA	NA	NA	NA
20	2	5	NA	NA	NA	NA	NA	NA
20	3	5	NA	NA	NA	NA	NA	NA
20	4	5	NA	NA	NA	NA	NA	NA
30	1	5	NA	NA	NA	NA	NA	NA
30	2	5	NA	NA	NA	NA	NA	NA
30	3	5	NA	NA	NA	NA	NA	NA
30	4	5	NA	NA	NA	NA	NA	NA
40	1	5	NA	NA	NA	NA	NA	NA
40	2	5	NA	NA	NA	NA	NA	NA
40	3	5	NA	NA	NA	NA	NA	NA
40	4	5	NA	NA	NA	NA	NA	NA
40	5	5	NA	NA	NA	NA	NA	NA
60	1	5	NA	NA	NA	NA	NA	NA
10	1	6	NA	NA	NA	NA	NA	NA
10	2	6	NA	NA	NA	NA	NA	NA
10	3	6	NA	NA	NA	NA	NA	NA
10	4	6	NA	NA	NA	NA	NA	NA
20	1	6	NA	NA	NA	NA	NA	NA
20	2	6	NA	NA	NA	NA	NA	NA
20	3	6	NA	NA	NA	NA	NA	NA
20	4	6	NA	NA	NA	NA	NA	NA
30	1	6	NA	NA	NA	NA	NA	NA
30	2	6	NA	NA	NA	NA	NA	NA
30	3	6	NA	NA	NA	NA	NA	NA
30	4	6	NA	NA	NA	NA	NA	NA
40	1	6	NA	NA	NA	NA	NA	NA
40	2	6	NA	NA	NA	NA	NA	NA
40	3	6	NA	NA	NA	NA	NA	NA
40	4	6	NA	NA	NA	NA	NA	NA

60	1	6	NA	NA	NA	NA	NA	NA
10	1	6	1	0	NA	NA	NA	NA
10	2	6	1	0	NA	NA	NA	NA
10	3	6	1	0	NA	NA	NA	NA
10	4	6	1	0	NA	NA	NA	NA
20	1	6	1	0	NA	NA	NA	NA
20	2	6	1	0	NA	NA	NA	NA
20	3	6	1	0	NA	NA	NA	NA
20	4	6	1	0	NA	NA	NA	NA
30	1	6	1	16.7	NA	NA	NA	NA
30	2	6	1	11.1	NA	NA	NA	NA
30	3	6	1	0	NA	NA	NA	NA
30	4	6	1	0	NA	NA	NA	NA
40	1	6	1	0	NA	NA	NA	NA
40	2	6	1	0	NA	NA	NA	NA
40	3	6	1	0	NA	NA	NA	NA
40	4	6	1	0	NA	NA	NA	NA
60	1	6	1	0	NA	NA	NA	NA
10	1	6	2	0	NA	NA	NA	NA
10	2	6	2	0	NA	NA	NA	NA
10	3	6	2	0	NA	NA	NA	NA
10	4	6	2	0	NA	NA	NA	NA
20	1	6	2	0	NA	NA	NA	NA
20	2	6	2	0	NA	NA	NA	NA
20	3	6	2	0	NA	NA	NA	NA
20	4	6	2	0	NA	NA	NA	NA
30	1	6	2	0	NA	NA	NA	NA
30	2	6	2	0	NA	NA	NA	NA
30	3	6	2	0	NA	NA	NA	NA
30	4	6	2	0	NA	NA	NA	NA
40	1	6	2	0	NA	NA	NA	NA
40	2	6	2	0	NA	NA	NA	NA
40	3	6	2	0	NA	NA	NA	NA
40	4	6	2	0	NA	NA	NA	NA
60	1	6	2	6.7	NA	NA	NA	NA
10	1	6	NA	0	NA	NA	NA	NA
10	2	6	NA	10	NA	NA	NA	NA

10	3	6	NA	0	NA	NA	NA	NA
10	4	6	NA	0	NA	NA	NA	NA
20	1	6	NA	0	NA	NA	NA	NA
20	2	6	NA	0	NA	NA	NA	NA
20	3	6	NA	0	NA	NA	NA	NA
20	4	6	NA	0	NA	NA	NA	NA
30	1	6	NA	6.7	NA	NA	NA	NA
30	2	6	NA	4.8	NA	NA	NA	NA
30	3	6	NA	0	NA	NA	NA	NA
30	4	6	NA	0	NA	NA	NA	NA
40	1	6	NA	0	NA	NA	NA	NA
40	2	6	NA	0	NA	NA	NA	NA
40	3	6	NA	0	NA	NA	NA	NA
40	4	6	NA	0	NA	NA	NA	NA
60	1	6	NA	3.2	NA	NA	NA	NA
10	1	5	NA	10	NA	NA	NA	NA
10	1	5	NA	0	NA	NA	NA	NA
10	2	5	NA	0	NA	NA	NA	NA
10	3	5	NA	0	NA	NA	NA	NA
10	4	5	NA	0	NA	NA	NA	NA
20	1	5	NA	0	NA	NA	NA	NA
20	2	5	NA	0	NA	NA	NA	NA
20	3	5	NA	0	NA	NA	NA	NA
20	4	5	NA	0	NA	NA	NA	NA
30	1	5	NA	0	NA	NA	NA	NA
30	2	5	NA	7.1	NA	NA	NA	NA
30	3	5	NA	27.3	NA	NA	NA	NA
30	4	5	NA	21	NA	NA	NA	NA
40	1	5	NA	4.8	NA	NA	NA	NA
40	2	5	NA	5	NA	NA	NA	NA
40	3	5	NA	4.5	NA	NA	NA	NA
40	4	5	NA	0	NA	NA	NA	NA
60	1	5	NA	3.3	NA	NA	NA	NA
10	1	6	NA	NA	9	1	0	10
10	2	6	NA	NA	10	1	1	12
10	3	6	NA	NA	7	3	2	12
10	4	6	NA	NA	7	0	1	8

20	1	6	NA	NA	16	3	2	21
20	2	6	NA	NA	11	1	2	14
20	3	6	NA	NA	10	5	1	16
20	4	6	NA	NA	13	6	1	20
30	1	6	NA	NA	16	9	1	26
30	2	6	NA	NA	19	7	2	28
30	3	6	NA	NA	16	4	3	23
30	4	6	NA	NA	23	5	3	31
40	1	6	NA	NA	15	9	7	31
40	2	6	NA	NA	23	12	6	41
40	3	6	NA	NA	23	9	2	34
40	4	6	NA	NA	22	10	6	38
60	1	6	NA	NA	24	12	9	45
10	1	5	NA	NA	7	1	1	9
10	2	5	NA	NA	8	6	1	15
10	3	5	NA	NA	8	3	0	11
10	4	5	NA	NA	9	2	0	11
20	1	5	NA	NA	7	4	0	11
20	2	5	NA	NA	11	1	0	12
20	3	5	NA	NA	13	2	0	15
20	4	5	NA	NA	11	2	1	14
30	1	5	NA	NA	18	1	1	20
30	2	5	NA	NA	14	4	0	18
30	3	5	NA	NA	18	6	2	26
30	4	5	NA	NA	14	5	1	20
40	1	5	NA	NA	25	10	2	37
40	2	5	NA	NA	27	9	6	42
40	3	5	NA	NA	29	7	2	38
40	4	5	NA	NA	24	9	8	41
40	5	5	NA	NA	22	10	7	39
60	1	5	NA	NA	39	19	12	48
10	1	6	1	NA	NA	NA	NA	NA
10	2	6	1	NA	NA	NA	NA	NA
10	3	6	1	NA	NA	NA	NA	NA
10	4	6	1	NA	NA	NA	NA	NA
20	1	6	1	NA	NA	NA	NA	NA
20	2	6	1	NA	NA	NA	NA	NA

20	3	6	1	NA	NA	NA	NA	NA
20	4	6	1	NA	NA	NA	NA	NA
30	1	6	1	NA	NA	NA	NA	NA
30	2	6	1	NA	NA	NA	NA	NA
30	3	6	1	NA	NA	NA	NA	NA
30	4	6	1	NA	NA	NA	NA	NA
40	1	6	1	NA	NA	NA	NA	NA
40	2	6	1	NA	NA	NA	NA	NA
40	3	6	1	NA	NA	NA	NA	NA
40	4	6	1	NA	NA	NA	NA	NA
60	1	6	1	NA	NA	NA	NA	NA
10	1	5	NA	NA	7	1	1	9
10	2	5	NA	NA	8	6	1	15
10	3	5	NA	NA	8	3	0	11
10	4	5	NA	NA	9	2	0	11
20	1	5	NA	NA	7	4	0	11
20	2	5	NA	NA	11	1	0	12
20	3	5	NA	NA	13	2	0	15
20	4	5	NA	NA	11	2	1	14
30	1	5	NA	NA	18	1	1	20
30	2	5	NA	NA	14	4	0	18
30	3	5	NA	NA	18	6	2	26
30	4	5	NA	NA	14	5	1	20
40	1	5	NA	NA	25	10	2	37
40	2	5	NA	NA	27	9	6	42
40	3	5	NA	NA	29	7	2	38
40	4	5	NA	NA	24	9	8	41
40	5	5	NA	NA	22	10	7	39
60	1	5	NA	NA	39	19	12	70
10	1	5	NA	NA	NA	NA	NA	NA
10	2	5	NA	NA	NA	NA	NA	NA
10	3	5	NA	NA	NA	NA	NA	NA
10	4	5	NA	NA	NA	NA	NA	NA
20	1	5	NA	NA	NA	NA	NA	NA
20	2	5	NA	NA	NA	NA	NA	NA
20	3	5	NA	NA	NA	NA	NA	NA
20	4	5	NA	NA	NA	NA	NA	NA

30	1	5	NA	NA	NA	NA	NA	NA
30	2	5	NA	NA	NA	NA	NA	NA
30	3	5	NA	NA	NA	NA	NA	NA
30	4	5	NA	NA	NA	NA	NA	NA
40	1	5	NA	NA	NA	NA	NA	NA
40	2	5	NA	NA	NA	NA	NA	NA
40	3	5	NA	NA	NA	NA	NA	NA
40	4	5	NA	NA	NA	NA	NA	NA
40	5	5	NA	NA	NA	NA	NA	NA
60	1	5	NA	NA	NA	NA	NA	NA

Dens: density; Replic: replicate; Stage [Nymph 5 (= 5) or Adult (= 6)]; Sex (1 = females, 2 = males); “NA“ indicates data not applicable or not available for the given combination.

(Part 12)

Dens	Replic	Stage	Sex	Pct feeding once in 3d	Pct feeding twice in 3d	Pct feeding thrice in 3d	N Fem Alive week1	N Fem Alive week2
10	1	5	NA	NA	NA	NA	NA	NA
10	2	5	NA	NA	NA	NA	NA	NA
10	3	5	NA	NA	NA	NA	NA	NA
10	4	5	NA	NA	NA	NA	NA	NA
20	1	5	NA	NA	NA	NA	NA	NA
20	2	5	NA	NA	NA	NA	NA	NA
20	3	5	NA	NA	NA	NA	NA	NA
20	4	5	NA	NA	NA	NA	NA	NA
30	1	5	NA	NA	NA	NA	NA	NA
30	2	5	NA	NA	NA	NA	NA	NA
30	3	5	NA	NA	NA	NA	NA	NA
30	4	5	NA	NA	NA	NA	NA	NA
40	1	5	NA	NA	NA	NA	NA	NA
40	2	5	NA	NA	NA	NA	NA	NA
40	3	5	NA	NA	NA	NA	NA	NA
40	4	5	NA	NA	NA	NA	NA	NA
40	5	5	NA	NA	NA	NA	NA	NA
60	1	5	NA	NA	NA	NA	NA	NA
10	1	6	NA	NA	NA	NA	NA	NA
10	2	6	NA	NA	NA	NA	NA	NA
10	3	6	NA	NA	NA	NA	NA	NA
10	4	6	NA	NA	NA	NA	NA	NA
20	1	6	NA	NA	NA	NA	NA	NA
20	2	6	NA	NA	NA	NA	NA	NA
20	3	6	NA	NA	NA	NA	NA	NA
20	4	6	NA	NA	NA	NA	NA	NA
30	1	6	NA	NA	NA	NA	NA	NA
30	2	6	NA	NA	NA	NA	NA	NA
30	3	6	NA	NA	NA	NA	NA	NA
30	4	6	NA	NA	NA	NA	NA	NA
40	1	6	NA	NA	NA	NA	NA	NA
40	2	6	NA	NA	NA	NA	NA	NA
40	3	6	NA	NA	NA	NA	NA	NA
40	4	6	NA	NA	NA	NA	NA	NA

60	1	6	NA	NA	NA	NA	NA	NA
10	1	6	1	NA	NA	NA	NA	NA
10	2	6	1	NA	NA	NA	NA	NA
10	3	6	1	NA	NA	NA	NA	NA
10	4	6	1	NA	NA	NA	NA	NA
20	1	6	1	NA	NA	NA	NA	NA
20	2	6	1	NA	NA	NA	NA	NA
20	3	6	1	NA	NA	NA	NA	NA
20	4	6	1	NA	NA	NA	NA	NA
30	1	6	1	NA	NA	NA	NA	NA
30	2	6	1	NA	NA	NA	NA	NA
30	3	6	1	NA	NA	NA	NA	NA
30	4	6	1	NA	NA	NA	NA	NA
40	1	6	1	NA	NA	NA	NA	NA
40	2	6	1	NA	NA	NA	NA	NA
40	3	6	1	NA	NA	NA	NA	NA
40	4	6	1	NA	NA	NA	NA	NA
60	1	6	1	NA	NA	NA	NA	NA
10	1	6	2	NA	NA	NA	NA	NA
10	2	6	2	NA	NA	NA	NA	NA
10	3	6	2	NA	NA	NA	NA	NA
10	4	6	2	NA	NA	NA	NA	NA
20	1	6	2	NA	NA	NA	NA	NA
20	2	6	2	NA	NA	NA	NA	NA
20	3	6	2	NA	NA	NA	NA	NA
20	4	6	2	NA	NA	NA	NA	NA
30	1	6	2	NA	NA	NA	NA	NA
30	2	6	2	NA	NA	NA	NA	NA
30	3	6	2	NA	NA	NA	NA	NA
30	4	6	2	NA	NA	NA	NA	NA
40	1	6	2	NA	NA	NA	NA	NA
40	2	6	2	NA	NA	NA	NA	NA
40	3	6	2	NA	NA	NA	NA	NA
40	4	6	2	NA	NA	NA	NA	NA
60	1	6	2	NA	NA	NA	NA	NA
10	1	6	NA	NA	NA	NA	NA	NA
10	2	6	NA	NA	NA	NA	NA	NA

10	3	6	NA	NA	NA	NA	NA	NA
10	4	6	NA	NA	NA	NA	NA	NA
20	1	6	NA	NA	NA	NA	NA	NA
20	2	6	NA	NA	NA	NA	NA	NA
20	3	6	NA	NA	NA	NA	NA	NA
20	4	6	NA	NA	NA	NA	NA	NA
30	1	6	NA	NA	NA	NA	NA	NA
30	2	6	NA	NA	NA	NA	NA	NA
30	3	6	NA	NA	NA	NA	NA	NA
30	4	6	NA	NA	NA	NA	NA	NA
40	1	6	NA	NA	NA	NA	NA	NA
40	2	6	NA	NA	NA	NA	NA	NA
40	3	6	NA	NA	NA	NA	NA	NA
40	4	6	NA	NA	NA	NA	NA	NA
60	1	6	NA	NA	NA	NA	NA	NA
10	1	5	NA	NA	NA	NA	NA	NA
10	1	5	NA	NA	NA	NA	NA	NA
10	2	5	NA	NA	NA	NA	NA	NA
10	3	5	NA	NA	NA	NA	NA	NA
10	4	5	NA	NA	NA	NA	NA	NA
20	1	5	NA	NA	NA	NA	NA	NA
20	2	5	NA	NA	NA	NA	NA	NA
20	3	5	NA	NA	NA	NA	NA	NA
20	4	5	NA	NA	NA	NA	NA	NA
30	1	5	NA	NA	NA	NA	NA	NA
30	2	5	NA	NA	NA	NA	NA	NA
30	3	5	NA	NA	NA	NA	NA	NA
30	4	5	NA	NA	NA	NA	NA	NA
40	1	5	NA	NA	NA	NA	NA	NA
40	2	5	NA	NA	NA	NA	NA	NA
40	3	5	NA	NA	NA	NA	NA	NA
40	4	5	NA	NA	NA	NA	NA	NA
60	1	5	NA	NA	NA	NA	NA	NA
10	1	6	NA	100	0	0	NA	NA
10	2	6	NA	80	20	0	NA	NA
10	3	6	NA	80	20	0	NA	NA
10	4	6	NA	100	0	0	NA	NA

20	1	6	NA	83.3	16.7	0	NA	NA
20	2	6	NA	71.4	14.3	0	NA	NA
20	3	6	NA	84.2	0	0	NA	NA
20	4	6	NA	88.9	11.1	0	NA	NA
30	1	6	NA	66.7	14.8	0	NA	NA
30	2	6	NA	66.7	18.5	0	NA	NA
30	3	6	NA	71.4	19.1	0	NA	NA
30	4	6	NA	82.1	10.7	3.6	NA	NA
40	1	6	NA	69.7	12.1	0	NA	NA
40	2	6	NA	68.6	19.4	2.9	NA	NA
40	3	6	NA	71	19.4	0	NA	NA
40	4	6	NA	78.1	18.9	0	NA	NA
60	1	6	NA	51.4	27	5.4	NA	NA
10	1	5	NA	62.5	25	0	NA	NA
10	2	5	NA	50	50	0	NA	NA
10	3	5	NA	90	10	0	NA	NA
10	4	5	NA	77.8	22.2	0	NA	NA
20	1	5	NA	75	8.3	0	NA	NA
20	2	5	NA	76.9	7.5	0	NA	NA
20	3	5	NA	92.9	7.1	0	NA	NA
20	4	5	NA	76.9	15.4	0	NA	NA
30	1	5	NA	85.7	4.7	0	NA	NA
30	2	5	NA	76.2	4.7	0	NA	NA
30	3	5	NA	70	15	10	NA	NA
30	4	5	NA	58.8	29.4	0	NA	NA
40	1	5	NA	75.9	20.7	3.4	NA	NA
40	2	5	NA	51.4	33.2	3	NA	NA
40	3	5	NA	71.1	14.3	2.8	NA	NA
40	4	5	NA	63.9	26.7	6.7	NA	NA
40	5	5	NA	48.6	22.8	5.7	NA	NA
60	1	5	NA	46.6	34.5	1.7	NA	NA
10	1	6	1	NA	NA	NA	6	4
10	2	6	1	NA	NA	NA	5	5
10	3	6	1	NA	NA	NA	5	5
10	4	6	1	NA	NA	NA	3	3
20	1	6	1	NA	NA	NA	6	8
20	2	6	1	NA	NA	NA	7	7

20	3	6	1	NA	NA	NA	10	9
20	4	6	1	NA	NA	NA	6	5
30	1	6	1	NA	NA	NA	11	8
30	2	6	1	NA	NA	NA	9	6
30	3	6	1	NA	NA	NA	9	8
30	4	6	1	NA	NA	NA	10	9
40	1	6	1	NA	NA	NA	13	9
40	2	6	1	NA	NA	NA	16	13
40	3	6	1	NA	NA	NA	12	8
40	4	6	1	NA	NA	NA	14	13
60	1	6	1	NA	NA	NA	14	12
10	1	5	NA	9	62.5	25	0	NA
10	2	5	NA	15	50	50	0	NA
10	3	5	NA	11	90	10	0	NA
10	4	5	NA	11	77.8	22.2	0	NA
20	1	5	NA	11	75	8.3	0	NA
20	2	5	NA	12	76.9	7.5	0	NA
20	3	5	NA	15	92.9	7.1	0	NA
20	4	5	NA	14	76.9	15.4	0	NA
30	1	5	NA	20	85.7	4.7	0	NA
30	2	5	NA	18	76.2	4.7	0	NA
30	3	5	NA	26	70	15	10	NA
30	4	5	NA	20	58.8	29.4	0	NA
40	1	5	NA	37	75.9	20.7	3.4	NA
40	2	5	NA	42	51.4	33.2	3	NA
40	3	5	NA	38	71.1	14.3	2.8	NA
40	4	5	NA	41	63.9	26.7	6.7	NA
40	5	5	NA	39	48.6	22.8	5.7	NA
60	1	5	NA	48	46.6	34.5	1.7	NA
10	1	5	NA	NA	NA	NA	NA	NA
10	2	5	NA	NA	NA	NA	NA	NA
10	3	5	NA	NA	NA	NA	NA	NA
10	4	5	NA	NA	NA	NA	NA	NA
20	1	5	NA	NA	NA	NA	NA	NA
20	2	5	NA	NA	NA	NA	NA	NA
20	3	5	NA	NA	NA	NA	NA	NA
20	4	5	NA	NA	NA	NA	NA	NA

30	1	5	NA	NA	NA	NA	NA	NA
30	2	5	NA	NA	NA	NA	NA	NA
30	3	5	NA	NA	NA	NA	NA	NA
30	4	5	NA	NA	NA	NA	NA	NA
40	1	5	NA	NA	NA	NA	NA	NA
40	2	5	NA	NA	NA	NA	NA	NA
40	3	5	NA	NA	NA	NA	NA	NA
40	4	5	NA	NA	NA	NA	NA	NA
40	5	5	NA	NA	NA	NA	NA	NA
60	1	5	NA	NA	NA	NA	NA	NA

Dens: density; Replic: replicate; Stage [Nymph 5 (= 5) or Adult (= 6)]; Sex (1 = females, 2 = males); “NA“ indicates data not applicable or not available for the given combination.

(Part 13)

Dens	Replic	Stage	Sex	N Fem Alive week3	Eggs laid w1	Eggs laid w2	Eggs laid w3	Eggs Fem mean w1
10	1	5	NA	NA	NA	NA	NA	NA
10	2	5	NA	NA	NA	NA	NA	NA
10	3	5	NA	NA	NA	NA	NA	NA
10	4	5	NA	NA	NA	NA	NA	NA
20	1	5	NA	NA	NA	NA	NA	NA
20	2	5	NA	NA	NA	NA	NA	NA
20	3	5	NA	NA	NA	NA	NA	NA
20	4	5	NA	NA	NA	NA	NA	NA
30	1	5	NA	NA	NA	NA	NA	NA
30	2	5	NA	NA	NA	NA	NA	NA
30	3	5	NA	NA	NA	NA	NA	NA
30	4	5	NA	NA	NA	NA	NA	NA
40	1	5	NA	NA	NA	NA	NA	NA
40	2	5	NA	NA	NA	NA	NA	NA
40	3	5	NA	NA	NA	NA	NA	NA
40	4	5	NA	NA	NA	NA	NA	NA
40	5	5	NA	NA	NA	NA	NA	NA
60	1	5	NA	NA	NA	NA	NA	NA
10	1	6	NA	NA	NA	NA	NA	NA
10	2	6	NA	NA	NA	NA	NA	NA
10	3	6	NA	NA	NA	NA	NA	NA
10	4	6	NA	NA	NA	NA	NA	NA
20	1	6	NA	NA	NA	NA	NA	NA
20	2	6	NA	NA	NA	NA	NA	NA
20	3	6	NA	NA	NA	NA	NA	NA
20	4	6	NA	NA	NA	NA	NA	NA
30	1	6	NA	NA	NA	NA	NA	NA
30	2	6	NA	NA	NA	NA	NA	NA
30	3	6	NA	NA	NA	NA	NA	NA
30	4	6	NA	NA	NA	NA	NA	NA
40	1	6	NA	NA	NA	NA	NA	NA
40	2	6	NA	NA	NA	NA	NA	NA
40	3	6	NA	NA	NA	NA	NA	NA
40	4	6	NA	NA	NA	NA	NA	NA

60	1	6	NA	NA	NA	NA	NA	NA
10	1	6	1	NA	NA	NA	NA	NA
10	2	6	1	NA	NA	NA	NA	NA
10	3	6	1	NA	NA	NA	NA	NA
10	4	6	1	NA	NA	NA	NA	NA
20	1	6	1	NA	NA	NA	NA	NA
20	2	6	1	NA	NA	NA	NA	NA
20	3	6	1	NA	NA	NA	NA	NA
20	4	6	1	NA	NA	NA	NA	NA
30	1	6	1	NA	NA	NA	NA	NA
30	2	6	1	NA	NA	NA	NA	NA
30	3	6	1	NA	NA	NA	NA	NA
30	4	6	1	NA	NA	NA	NA	NA
40	1	6	1	NA	NA	NA	NA	NA
40	2	6	1	NA	NA	NA	NA	NA
40	3	6	1	NA	NA	NA	NA	NA
40	4	6	1	NA	NA	NA	NA	NA
60	1	6	1	NA	NA	NA	NA	NA
10	1	6	2	NA	NA	NA	NA	NA
10	2	6	2	NA	NA	NA	NA	NA
10	3	6	2	NA	NA	NA	NA	NA
10	4	6	2	NA	NA	NA	NA	NA
20	1	6	2	NA	NA	NA	NA	NA
20	2	6	2	NA	NA	NA	NA	NA
20	3	6	2	NA	NA	NA	NA	NA
20	4	6	2	NA	NA	NA	NA	NA
30	1	6	2	NA	NA	NA	NA	NA
30	2	6	2	NA	NA	NA	NA	NA
30	3	6	2	NA	NA	NA	NA	NA
30	4	6	2	NA	NA	NA	NA	NA
40	1	6	2	NA	NA	NA	NA	NA
40	2	6	2	NA	NA	NA	NA	NA
40	3	6	2	NA	NA	NA	NA	NA
40	4	6	2	NA	NA	NA	NA	NA
60	1	6	2	NA	NA	NA	NA	NA
10	1	6	NA	NA	NA	NA	NA	NA
10	2	6	NA	NA	NA	NA	NA	NA

10	3	6	NA	NA	NA	NA	NA	NA
10	4	6	NA	NA	NA	NA	NA	NA
20	1	6	NA	NA	NA	NA	NA	NA
20	2	6	NA	NA	NA	NA	NA	NA
20	3	6	NA	NA	NA	NA	NA	NA
20	4	6	NA	NA	NA	NA	NA	NA
30	1	6	NA	NA	NA	NA	NA	NA
30	2	6	NA	NA	NA	NA	NA	NA
30	3	6	NA	NA	NA	NA	NA	NA
30	4	6	NA	NA	NA	NA	NA	NA
40	1	6	NA	NA	NA	NA	NA	NA
40	2	6	NA	NA	NA	NA	NA	NA
40	3	6	NA	NA	NA	NA	NA	NA
40	4	6	NA	NA	NA	NA	NA	NA
60	1	6	NA	NA	NA	NA	NA	NA
10	1	5	NA	NA	NA	NA	NA	NA
10	1	5	NA	NA	NA	NA	NA	NA
10	2	5	NA	NA	NA	NA	NA	NA
10	3	5	NA	NA	NA	NA	NA	NA
10	4	5	NA	NA	NA	NA	NA	NA
20	1	5	NA	NA	NA	NA	NA	NA
20	2	5	NA	NA	NA	NA	NA	NA
20	3	5	NA	NA	NA	NA	NA	NA
20	4	5	NA	NA	NA	NA	NA	NA
30	1	5	NA	NA	NA	NA	NA	NA
30	2	5	NA	NA	NA	NA	NA	NA
30	3	5	NA	NA	NA	NA	NA	NA
30	4	5	NA	NA	NA	NA	NA	NA
40	1	5	NA	NA	NA	NA	NA	NA
40	2	5	NA	NA	NA	NA	NA	NA
40	3	5	NA	NA	NA	NA	NA	NA
40	4	5	NA	NA	NA	NA	NA	NA
60	1	5	NA	NA	NA	NA	NA	NA
10	1	6	NA	NA	NA	NA	NA	NA
10	2	6	NA	NA	NA	NA	NA	NA
10	3	6	NA	NA	NA	NA	NA	NA
10	4	6	NA	NA	NA	NA	NA	NA

20	1	6	NA	NA	NA	NA	NA	NA
20	2	6	NA	NA	NA	NA	NA	NA
20	3	6	NA	NA	NA	NA	NA	NA
20	4	6	NA	NA	NA	NA	NA	NA
30	1	6	NA	NA	NA	NA	NA	NA
30	2	6	NA	NA	NA	NA	NA	NA
30	3	6	NA	NA	NA	NA	NA	NA
30	4	6	NA	NA	NA	NA	NA	NA
40	1	6	NA	NA	NA	NA	NA	NA
40	2	6	NA	NA	NA	NA	NA	NA
40	3	6	NA	NA	NA	NA	NA	NA
40	4	6	NA	NA	NA	NA	NA	NA
60	1	6	NA	NA	NA	NA	NA	NA
10	1	5	NA	NA	NA	NA	NA	NA
10	2	5	NA	NA	NA	NA	NA	NA
10	3	5	NA	NA	NA	NA	NA	NA
10	4	5	NA	NA	NA	NA	NA	NA
20	1	5	NA	NA	NA	NA	NA	NA
20	2	5	NA	NA	NA	NA	NA	NA
20	3	5	NA	NA	NA	NA	NA	NA
20	4	5	NA	NA	NA	NA	NA	NA
30	1	5	NA	NA	NA	NA	NA	NA
30	2	5	NA	NA	NA	NA	NA	NA
30	3	5	NA	NA	NA	NA	NA	NA
30	4	5	NA	NA	NA	NA	NA	NA
40	1	5	NA	NA	NA	NA	NA	NA
40	2	5	NA	NA	NA	NA	NA	NA
40	3	5	NA	NA	NA	NA	NA	NA
40	4	5	NA	NA	NA	NA	NA	NA
40	5	5	NA	NA	NA	NA	NA	NA
60	1	5	NA	NA	NA	NA	NA	NA
10	1	6	1	2	86	50	8	14.3
10	2	6	1	5	97	78	19	19.4
10	3	6	1	5	84	80	44	16.8
10	4	6	1	1	53	32	2	17.7
20	1	6	1	4	120	115	19	15
20	2	6	1	5	128	36	36	18.3

20	3	6	1	9	153	69	14	15.3
20	4	6	1	5	94	61	6	15.7
30	1	6	1	6	185	32	1	16.8
30	2	6	1	4	130	64	0	58.5
30	3	6	1	7	126	79	23	14
30	4	6	1	9	111	133	33	11.1
40	1	6	1	5	87	27	5	6.7
40	2	6	1	7	126	49	7	7.9
40	3	6	1	5	75	35	5	6.3
40	4	6	1	11	151	66	11	10.8
60	1	6	1	9	52	97	6	3.7
10	1	5	NA	NA	NA	NA	NA	NA
10	2	5	NA	NA	NA	NA	NA	NA
10	3	5	NA	NA	NA	NA	NA	NA
10	4	5	NA	NA	NA	NA	NA	NA
20	1	5	NA	NA	NA	NA	NA	NA
20	2	5	NA	NA	NA	NA	NA	NA
20	3	5	NA	NA	NA	NA	NA	NA
20	4	5	NA	NA	NA	NA	NA	NA
30	1	5	NA	NA	NA	NA	NA	NA
30	2	5	NA	NA	NA	NA	NA	NA
30	3	5	NA	NA	NA	NA	NA	NA
30	4	5	NA	NA	NA	NA	NA	NA
40	1	5	NA	NA	NA	NA	NA	NA
40	2	5	NA	NA	NA	NA	NA	NA
40	3	5	NA	NA	NA	NA	NA	NA
40	4	5	NA	NA	NA	NA	NA	NA
40	5	5	NA	NA	NA	NA	NA	NA
60	1	5	NA	NA	NA	NA	NA	NA
10	1	5	NA	NA	NA	NA	NA	NA
10	2	5	NA	NA	NA	NA	NA	NA
10	3	5	NA	NA	NA	NA	NA	NA
10	4	5	NA	NA	NA	NA	NA	NA
20	1	5	NA	NA	NA	NA	NA	NA
20	2	5	NA	NA	NA	NA	NA	NA
20	3	5	NA	NA	NA	NA	NA	NA
20	4	5	NA	NA	NA	NA	NA	NA

30	1	5	NA	NA	NA	NA	NA	NA
30	2	5	NA	NA	NA	NA	NA	NA
30	3	5	NA	NA	NA	NA	NA	NA
30	4	5	NA	NA	NA	NA	NA	NA
40	1	5	NA	NA	NA	NA	NA	NA
40	2	5	NA	NA	NA	NA	NA	NA
40	3	5	NA	NA	NA	NA	NA	NA
40	4	5	NA	NA	NA	NA	NA	NA
40	5	5	NA	NA	NA	NA	NA	NA
60	1	5	NA	NA	NA	NA	NA	NA

Dens: density; Replic: replicate; Stage [Nymph 5 (= 5) or Adult (= 6)]; Sex (1 = females, 2 = males); “NA“ indicates data not applicable or not available for the given combination.

(Part 14)

Dens	Replic	Stage	Sex	N Fem Alive week3	Eggs laid w1	Eggs laid w2	Eggs laid w3	Eggs Fem mean w1
10	1	5	NA	NA	NA	NA	NA	NA
10	2	5	NA	NA	NA	NA	NA	NA
10	3	5	NA	NA	NA	NA	NA	NA
10	4	5	NA	NA	NA	NA	NA	NA
20	1	5	NA	NA	NA	NA	NA	NA
20	2	5	NA	NA	NA	NA	NA	NA
20	3	5	NA	NA	NA	NA	NA	NA
20	4	5	NA	NA	NA	NA	NA	NA
30	1	5	NA	NA	NA	NA	NA	NA
30	2	5	NA	NA	NA	NA	NA	NA
30	3	5	NA	NA	NA	NA	NA	NA
30	4	5	NA	NA	NA	NA	NA	NA
40	1	5	NA	NA	NA	NA	NA	NA
40	2	5	NA	NA	NA	NA	NA	NA
40	3	5	NA	NA	NA	NA	NA	NA
40	4	5	NA	NA	NA	NA	NA	NA
40	5	5	NA	NA	NA	NA	NA	NA
60	1	5	NA	NA	NA	NA	NA	NA
10	1	6	NA	NA	NA	NA	NA	NA
10	2	6	NA	NA	NA	NA	NA	NA
10	3	6	NA	NA	NA	NA	NA	NA
10	4	6	NA	NA	NA	NA	NA	NA
20	1	6	NA	NA	NA	NA	NA	NA
20	2	6	NA	NA	NA	NA	NA	NA
20	3	6	NA	NA	NA	NA	NA	NA
20	4	6	NA	NA	NA	NA	NA	NA
30	1	6	NA	NA	NA	NA	NA	NA
30	2	6	NA	NA	NA	NA	NA	NA
30	3	6	NA	NA	NA	NA	NA	NA
30	4	6	NA	NA	NA	NA	NA	NA
40	1	6	NA	NA	NA	NA	NA	NA
40	2	6	NA	NA	NA	NA	NA	NA
40	3	6	NA	NA	NA	NA	NA	NA
40	4	6	NA	NA	NA	NA	NA	NA
60	1	6	NA	NA	NA	NA	NA	NA

10	1	6	1	NA	NA	NA	NA	NA
10	2	6	1	NA	NA	NA	NA	NA
10	3	6	1	NA	NA	NA	NA	NA
10	4	6	1	NA	NA	NA	NA	NA
20	1	6	1	NA	NA	NA	NA	NA
20	2	6	1	NA	NA	NA	NA	NA
20	3	6	1	NA	NA	NA	NA	NA
20	4	6	1	NA	NA	NA	NA	NA
30	1	6	1	NA	NA	NA	NA	NA
30	2	6	1	NA	NA	NA	NA	NA
30	3	6	1	NA	NA	NA	NA	NA
30	4	6	1	NA	NA	NA	NA	NA
40	1	6	1	NA	NA	NA	NA	NA
40	2	6	1	NA	NA	NA	NA	NA
40	3	6	1	NA	NA	NA	NA	NA
40	4	6	1	NA	NA	NA	NA	NA
60	1	6	1	NA	NA	NA	NA	NA
10	1	6	2	NA	NA	NA	NA	NA
10	2	6	2	NA	NA	NA	NA	NA
10	3	6	2	NA	NA	NA	NA	NA
10	4	6	2	NA	NA	NA	NA	NA
20	1	6	2	NA	NA	NA	NA	NA
20	2	6	2	NA	NA	NA	NA	NA
20	3	6	2	NA	NA	NA	NA	NA
20	4	6	2	NA	NA	NA	NA	NA
30	1	6	2	NA	NA	NA	NA	NA
30	2	6	2	NA	NA	NA	NA	NA
30	3	6	2	NA	NA	NA	NA	NA
30	4	6	2	NA	NA	NA	NA	NA
40	1	6	2	NA	NA	NA	NA	NA
40	2	6	2	NA	NA	NA	NA	NA
40	3	6	2	NA	NA	NA	NA	NA
40	4	6	2	NA	NA	NA	NA	NA
60	1	6	2	NA	NA	NA	NA	NA
10	1	6	NA	NA	NA	NA	NA	NA
10	2	6	NA	NA	NA	NA	NA	NA
10	3	6	NA	NA	NA	NA	NA	NA

10	4	6	NA	NA	NA	NA	NA	NA
20	1	6	NA	NA	NA	NA	NA	NA
20	2	6	NA	NA	NA	NA	NA	NA
20	3	6	NA	NA	NA	NA	NA	NA
20	4	6	NA	NA	NA	NA	NA	NA
30	1	6	NA	NA	NA	NA	NA	NA
30	2	6	NA	NA	NA	NA	NA	NA
30	3	6	NA	NA	NA	NA	NA	NA
30	4	6	NA	NA	NA	NA	NA	NA
40	1	6	NA	NA	NA	NA	NA	NA
40	2	6	NA	NA	NA	NA	NA	NA
40	3	6	NA	NA	NA	NA	NA	NA
40	4	6	NA	NA	NA	NA	NA	NA
60	1	6	NA	NA	NA	NA	NA	NA
10	1	5	NA	NA	NA	NA	NA	NA
10	1	5	NA	NA	NA	NA	NA	NA
10	2	5	NA	NA	NA	NA	NA	NA
10	3	5	NA	NA	NA	NA	NA	NA
10	4	5	NA	NA	NA	NA	NA	NA
20	1	5	NA	NA	NA	NA	NA	NA
20	2	5	NA	NA	NA	NA	NA	NA
20	3	5	NA	NA	NA	NA	NA	NA
20	4	5	NA	NA	NA	NA	NA	NA
30	1	5	NA	NA	NA	NA	NA	NA
30	2	5	NA	NA	NA	NA	NA	NA
30	3	5	NA	NA	NA	NA	NA	NA
30	4	5	NA	NA	NA	NA	NA	NA
40	1	5	NA	NA	NA	NA	NA	NA
40	2	5	NA	NA	NA	NA	NA	NA
40	3	5	NA	NA	NA	NA	NA	NA
40	4	5	NA	NA	NA	NA	NA	NA
60	1	5	NA	NA	NA	NA	NA	NA
10	1	6	NA	NA	NA	NA	NA	NA
10	2	6	NA	NA	NA	NA	NA	NA
10	3	6	NA	NA	NA	NA	NA	NA
10	4	6	NA	NA	NA	NA	NA	NA
20	1	6	NA	NA	NA	NA	NA	NA

20	2	6	NA	NA	NA	NA	NA	NA
20	3	6	NA	NA	NA	NA	NA	NA
20	4	6	NA	NA	NA	NA	NA	NA
30	1	6	NA	NA	NA	NA	NA	NA
30	2	6	NA	NA	NA	NA	NA	NA
30	3	6	NA	NA	NA	NA	NA	NA
30	4	6	NA	NA	NA	NA	NA	NA
40	1	6	NA	NA	NA	NA	NA	NA
40	2	6	NA	NA	NA	NA	NA	NA
40	3	6	NA	NA	NA	NA	NA	NA
40	4	6	NA	NA	NA	NA	NA	NA
60	1	6	NA	NA	NA	NA	NA	NA
10	1	5	NA	NA	NA	NA	NA	NA
10	2	5	NA	NA	NA	NA	NA	NA
10	3	5	NA	NA	NA	NA	NA	NA
10	4	5	NA	NA	NA	NA	NA	NA
20	1	5	NA	NA	NA	NA	NA	NA
20	2	5	NA	NA	NA	NA	NA	NA
20	3	5	NA	NA	NA	NA	NA	NA
20	4	5	NA	NA	NA	NA	NA	NA
30	1	5	NA	NA	NA	NA	NA	NA
30	2	5	NA	NA	NA	NA	NA	NA
30	3	5	NA	NA	NA	NA	NA	NA
30	4	5	NA	NA	NA	NA	NA	NA
40	1	5	NA	NA	NA	NA	NA	NA
40	2	5	NA	NA	NA	NA	NA	NA
40	3	5	NA	NA	NA	NA	NA	NA
40	4	5	NA	NA	NA	NA	NA	NA
40	5	5	NA	NA	NA	NA	NA	NA
60	1	5	NA	NA	NA	NA	NA	NA
10	1	6	1	2	86	50	8	14.3
10	2	6	1	5	97	78	19	19.4
10	3	6	1	5	84	80	44	16.8
10	4	6	1	1	53	32	2	17.7
20	1	6	1	4	120	115	19	15
20	2	6	1	5	128	36	36	18.3
20	3	6	1	9	153	69	14	15.3

20	4	6	1	5	94	61	6	15.7
30	1	6	1	6	185	32	1	16.8
30	2	6	1	4	130	64	0	58.5
30	3	6	1	7	126	79	23	14
30	4	6	1	9	111	133	33	11.1
40	1	6	1	5	87	27	5	6.7
40	2	6	1	7	126	49	7	7.9
40	3	6	1	5	75	35	5	6.3
40	4	6	1	11	151	66	11	10.8
60	1	6	1	9	52	97	6	3.7
10	1	5	NA	NA	NA	NA	NA	NA
10	2	5	NA	NA	NA	NA	NA	NA
10	3	5	NA	NA	NA	NA	NA	NA
10	4	5	NA	NA	NA	NA	NA	NA
20	1	5	NA	NA	NA	NA	NA	NA
20	2	5	NA	NA	NA	NA	NA	NA
20	3	5	NA	NA	NA	NA	NA	NA
20	4	5	NA	NA	NA	NA	NA	NA
30	1	5	NA	NA	NA	NA	NA	NA
30	2	5	NA	NA	NA	NA	NA	NA
30	3	5	NA	NA	NA	NA	NA	NA
30	4	5	NA	NA	NA	NA	NA	NA
40	1	5	NA	NA	NA	NA	NA	NA
40	2	5	NA	NA	NA	NA	NA	NA
40	3	5	NA	NA	NA	NA	NA	NA
40	4	5	NA	NA	NA	NA	NA	NA
40	5	5	NA	NA	NA	NA	NA	NA
60	1	5	NA	NA	NA	NA	NA	NA
10	1	5	NA	NA	NA	NA	NA	NA
10	2	5	NA	NA	NA	NA	NA	NA
10	3	5	NA	NA	NA	NA	NA	NA
10	4	5	NA	NA	NA	NA	NA	NA
20	1	5	NA	NA	NA	NA	NA	NA
20	2	5	NA	NA	NA	NA	NA	NA
20	3	5	NA	NA	NA	NA	NA	NA
20	4	5	NA	NA	NA	NA	NA	NA
30	1	5	NA	NA	NA	NA	NA	NA

30	2	5	NA	NA	NA	NA	NA	NA
30	3	5	NA	NA	NA	NA	NA	NA
30	4	5	NA	NA	NA	NA	NA	NA
40	1	5	NA	NA	NA	NA	NA	NA
40	2	5	NA	NA	NA	NA	NA	NA
40	3	5	NA	NA	NA	NA	NA	NA
40	4	5	NA	NA	NA	NA	NA	NA
40	5	5	NA	NA	NA	NA	NA	NA
60	1	5	NA	NA	NA	NA	NA	NA

Dens: density; Replic: replicate; Stage [Nymph 5 (= 5) or Adult (= 6)]; Sex (1 = females, 2 = males); “NA“ indicates data not applicable or not available for the given combination.

(Part 15)

Dens	Replic	Stage	Sex	Eggs Fem mean w2	Eggs Fem mean w3	Eggs Fem sd w1	Eggs Fem sd w2	Eggs Fem sd w3
10	1	5	NA	NA	NA	NA	NA	NA
10	2	5	NA	NA	NA	NA	NA	NA
10	3	5	NA	NA	NA	NA	NA	NA
10	4	5	NA	NA	NA	NA	NA	NA
20	1	5	NA	NA	NA	NA	NA	NA
20	2	5	NA	NA	NA	NA	NA	NA
20	3	5	NA	NA	NA	NA	NA	NA
20	4	5	NA	NA	NA	NA	NA	NA
30	1	5	NA	NA	NA	NA	NA	NA
30	2	5	NA	NA	NA	NA	NA	NA
30	3	5	NA	NA	NA	NA	NA	NA
30	4	5	NA	NA	NA	NA	NA	NA
40	1	5	NA	NA	NA	NA	NA	NA
40	2	5	NA	NA	NA	NA	NA	NA
40	3	5	NA	NA	NA	NA	NA	NA
40	4	5	NA	NA	NA	NA	NA	NA
40	5	5	NA	NA	NA	NA	NA	NA
60	1	5	NA	NA	NA	NA	NA	NA
10	1	6	NA	NA	NA	NA	NA	NA
10	2	6	NA	NA	NA	NA	NA	NA
10	3	6	NA	NA	NA	NA	NA	NA
10	4	6	NA	NA	NA	NA	NA	NA
20	1	6	NA	NA	NA	NA	NA	NA
20	2	6	NA	NA	NA	NA	NA	NA
20	3	6	NA	NA	NA	NA	NA	NA
20	4	6	NA	NA	NA	NA	NA	NA
30	1	6	NA	NA	NA	NA	NA	NA
30	2	6	NA	NA	NA	NA	NA	NA
30	3	6	NA	NA	NA	NA	NA	NA
30	4	6	NA	NA	NA	NA	NA	NA
40	1	6	NA	NA	NA	NA	NA	NA
40	2	6	NA	NA	NA	NA	NA	NA
40	3	6	NA	NA	NA	NA	NA	NA
40	4	6	NA	NA	NA	NA	NA	NA
60	1	6	NA	NA	NA	NA	NA	NA

10	1	6	1	NA	NA	NA	NA	NA
10	2	6	1	NA	NA	NA	NA	NA
10	3	6	1	NA	NA	NA	NA	NA
10	4	6	1	NA	NA	NA	NA	NA
20	1	6	1	NA	NA	NA	NA	NA
20	2	6	1	NA	NA	NA	NA	NA
20	3	6	1	NA	NA	NA	NA	NA
20	4	6	1	NA	NA	NA	NA	NA
30	1	6	1	NA	NA	NA	NA	NA
30	2	6	1	NA	NA	NA	NA	NA
30	3	6	1	NA	NA	NA	NA	NA
30	4	6	1	NA	NA	NA	NA	NA
40	1	6	1	NA	NA	NA	NA	NA
40	2	6	1	NA	NA	NA	NA	NA
40	3	6	1	NA	NA	NA	NA	NA
40	4	6	1	NA	NA	NA	NA	NA
60	1	6	1	NA	NA	NA	NA	NA
10	1	6	2	NA	NA	NA	NA	NA
10	2	6	2	NA	NA	NA	NA	NA
10	3	6	2	NA	NA	NA	NA	NA
10	4	6	2	NA	NA	NA	NA	NA
20	1	6	2	NA	NA	NA	NA	NA
20	2	6	2	NA	NA	NA	NA	NA
20	3	6	2	NA	NA	NA	NA	NA
20	4	6	2	NA	NA	NA	NA	NA
30	1	6	2	NA	NA	NA	NA	NA
30	2	6	2	NA	NA	NA	NA	NA
30	3	6	2	NA	NA	NA	NA	NA
30	4	6	2	NA	NA	NA	NA	NA
40	1	6	2	NA	NA	NA	NA	NA
40	2	6	2	NA	NA	NA	NA	NA
40	3	6	2	NA	NA	NA	NA	NA
40	4	6	2	NA	NA	NA	NA	NA
60	1	6	2	NA	NA	NA	NA	NA
10	1	6	NA	NA	NA	NA	NA	NA
10	2	6	NA	NA	NA	NA	NA	NA
10	3	6	NA	NA	NA	NA	NA	NA

10	4	6	NA	NA	NA	NA	NA	NA
20	1	6	NA	NA	NA	NA	NA	NA
20	2	6	NA	NA	NA	NA	NA	NA
20	3	6	NA	NA	NA	NA	NA	NA
20	4	6	NA	NA	NA	NA	NA	NA
30	1	6	NA	NA	NA	NA	NA	NA
30	2	6	NA	NA	NA	NA	NA	NA
30	3	6	NA	NA	NA	NA	NA	NA
30	4	6	NA	NA	NA	NA	NA	NA
40	1	6	NA	NA	NA	NA	NA	NA
40	2	6	NA	NA	NA	NA	NA	NA
40	3	6	NA	NA	NA	NA	NA	NA
40	4	6	NA	NA	NA	NA	NA	NA
60	1	6	NA	NA	NA	NA	NA	NA
10	1	5	NA	NA	NA	NA	NA	NA
10	1	5	NA	NA	NA	NA	NA	NA
10	2	5	NA	NA	NA	NA	NA	NA
10	3	5	NA	NA	NA	NA	NA	NA
10	4	5	NA	NA	NA	NA	NA	NA
20	1	5	NA	NA	NA	NA	NA	NA
20	2	5	NA	NA	NA	NA	NA	NA
20	3	5	NA	NA	NA	NA	NA	NA
20	4	5	NA	NA	NA	NA	NA	NA
30	1	5	NA	NA	NA	NA	NA	NA
30	2	5	NA	NA	NA	NA	NA	NA
30	3	5	NA	NA	NA	NA	NA	NA
30	4	5	NA	NA	NA	NA	NA	NA
40	1	5	NA	NA	NA	NA	NA	NA
40	2	5	NA	NA	NA	NA	NA	NA
40	3	5	NA	NA	NA	NA	NA	NA
40	4	5	NA	NA	NA	NA	NA	NA
60	1	5	NA	NA	NA	NA	NA	NA
10	1	6	NA	NA	NA	NA	NA	NA
10	2	6	NA	NA	NA	NA	NA	NA
10	3	6	NA	NA	NA	NA	NA	NA
10	4	6	NA	NA	NA	NA	NA	NA
20	1	6	NA	NA	NA	NA	NA	NA

20	2	6	NA	NA	NA	NA	NA	NA
20	3	6	NA	NA	NA	NA	NA	NA
20	4	6	NA	NA	NA	NA	NA	NA
30	1	6	NA	NA	NA	NA	NA	NA
30	2	6	NA	NA	NA	NA	NA	NA
30	3	6	NA	NA	NA	NA	NA	NA
30	4	6	NA	NA	NA	NA	NA	NA
40	1	6	NA	NA	NA	NA	NA	NA
40	2	6	NA	NA	NA	NA	NA	NA
40	3	6	NA	NA	NA	NA	NA	NA
40	4	6	NA	NA	NA	NA	NA	NA
60	1	6	NA	NA	NA	NA	NA	NA
10	1	5	NA	NA	NA	NA	NA	NA
10	2	5	NA	NA	NA	NA	NA	NA
10	3	5	NA	NA	NA	NA	NA	NA
10	4	5	NA	NA	NA	NA	NA	NA
20	1	5	NA	NA	NA	NA	NA	NA
20	2	5	NA	NA	NA	NA	NA	NA
20	3	5	NA	NA	NA	NA	NA	NA
20	4	5	NA	NA	NA	NA	NA	NA
30	1	5	NA	NA	NA	NA	NA	NA
30	2	5	NA	NA	NA	NA	NA	NA
30	3	5	NA	NA	NA	NA	NA	NA
30	4	5	NA	NA	NA	NA	NA	NA
40	1	5	NA	NA	NA	NA	NA	NA
40	2	5	NA	NA	NA	NA	NA	NA
40	3	5	NA	NA	NA	NA	NA	NA
40	4	5	NA	NA	NA	NA	NA	NA
40	5	5	NA	NA	NA	NA	NA	NA
60	1	5	NA	NA	NA	NA	NA	NA
10	1	6	1	12.5	4	4.3	4.7	5.7
10	2	6	1	15.6	3.8	7.6	6.5	7.4
10	3	6	1	16	8.8	10.3	NA	9.9
10	4	6	1	10.7	2	5	5.5	0
20	1	6	1	14.8	3.8	11	9.7	6.9
20	2	6	1	5.1	7.2	18.5	5.2	6.1
20	3	6	1	7.7	1.6	11.8	6.6	2.5

20	4	6	1	12.2	1.2	12.6	10.9	2.2
30	1	6	1	4	0.2	14.3	5.5	0.4
30	2	6	1	82.4	0	12.5	6.7	0
30	3	6	1	9.9	3.3	5.3	11.7	5.9
30	4	6	1	14.8	3.9	7.2	10.1	4.3
40	1	6	1	3	0	6.7	2.6	0
40	2	6	1	3.8	0	7.5	4.2	0
40	3	6	1	4.4	0	31.1	32.4	0
40	4	6	1	5.1	0.2	9.9	5.5	0.4
60	1	6	1	8.1	0.7	6.7	10.7	1.3
10	1	5	NA	NA	NA	NA	NA	NA
10	2	5	NA	NA	NA	NA	NA	NA
10	3	5	NA	NA	NA	NA	NA	NA
10	4	5	NA	NA	NA	NA	NA	NA
20	1	5	NA	NA	NA	NA	NA	NA
20	2	5	NA	NA	NA	NA	NA	NA
20	3	5	NA	NA	NA	NA	NA	NA
20	4	5	NA	NA	NA	NA	NA	NA
30	1	5	NA	NA	NA	NA	NA	NA
30	2	5	NA	NA	NA	NA	NA	NA
30	3	5	NA	NA	NA	NA	NA	NA
30	4	5	NA	NA	NA	NA	NA	NA
40	1	5	NA	NA	NA	NA	NA	NA
40	2	5	NA	NA	NA	NA	NA	NA
40	3	5	NA	NA	NA	NA	NA	NA
40	4	5	NA	NA	NA	NA	NA	NA
40	5	5	NA	NA	NA	NA	NA	NA
60	1	5	NA	NA	NA	NA	NA	NA
10	1	5	NA	NA	NA	NA	NA	NA
10	2	5	NA	NA	NA	NA	NA	NA
10	3	5	NA	NA	NA	NA	NA	NA
10	4	5	NA	NA	NA	NA	NA	NA
20	1	5	NA	NA	NA	NA	NA	NA
20	2	5	NA	NA	NA	NA	NA	NA
20	3	5	NA	NA	NA	NA	NA	NA
20	4	5	NA	NA	NA	NA	NA	NA
30	1	5	NA	NA	NA	NA	NA	NA

30	2	5	NA	NA	NA	NA	NA	NA
30	3	5	NA	NA	NA	NA	NA	NA
30	4	5	NA	NA	NA	NA	NA	NA
40	1	5	NA	NA	NA	NA	NA	NA
40	2	5	NA	NA	NA	NA	NA	NA
40	3	5	NA	NA	NA	NA	NA	NA
40	4	5	NA	NA	NA	NA	NA	NA
40	5	5	NA	NA	NA	NA	NA	NA
60	1	5	NA	NA	NA	NA	NA	NA

Dens: density; Replic: replicate; Stage [Nymph 5 (= 5) or Adult (= 6)]; Sex (1 = females, 2 = males); "NA" indicates data not applicable or not available for the given combination.

(Part 16)

Dens	Replic	Stage	Sex	Pct Hatch w1 mean	Pct Hatch w2 mean	Pct Hatch w3 mean	Pct Hatch w1 sd	Pct Hatch w2 sd
10	1	5	NA	NA	NA	NA	NA	NA
10	2	5	NA	NA	NA	NA	NA	NA
10	3	5	NA	NA	NA	NA	NA	NA
10	4	5	NA	NA	NA	NA	NA	NA
20	1	5	NA	NA	NA	NA	NA	NA
20	2	5	NA	NA	NA	NA	NA	NA
20	3	5	NA	NA	NA	NA	NA	NA
20	4	5	NA	NA	NA	NA	NA	NA
30	1	5	NA	NA	NA	NA	NA	NA
30	2	5	NA	NA	NA	NA	NA	NA
30	3	5	NA	NA	NA	NA	NA	NA
30	4	5	NA	NA	NA	NA	NA	NA
40	1	5	NA	NA	NA	NA	NA	NA
40	2	5	NA	NA	NA	NA	NA	NA
40	3	5	NA	NA	NA	NA	NA	NA
40	4	5	NA	NA	NA	NA	NA	NA
40	5	5	NA	NA	NA	NA	NA	NA
60	1	5	NA	NA	NA	NA	NA	NA
10	1	6	NA	NA	NA	NA	NA	NA
10	2	6	NA	NA	NA	NA	NA	NA
10	3	6	NA	NA	NA	NA	NA	NA
10	4	6	NA	NA	NA	NA	NA	NA
20	1	6	NA	NA	NA	NA	NA	NA
20	2	6	NA	NA	NA	NA	NA	NA
20	3	6	NA	NA	NA	NA	NA	NA
20	4	6	NA	NA	NA	NA	NA	NA
30	1	6	NA	NA	NA	NA	NA	NA
30	2	6	NA	NA	NA	NA	NA	NA
30	3	6	NA	NA	NA	NA	NA	NA
30	4	6	NA	NA	NA	NA	NA	NA
40	1	6	NA	NA	NA	NA	NA	NA
40	2	6	NA	NA	NA	NA	NA	NA
40	3	6	NA	NA	NA	NA	NA	NA
40	4	6	NA	NA	NA	NA	NA	NA
60	1	6	NA	NA	NA	NA	NA	NA

10	1	6	1	NA	NA	NA	NA	NA
10	2	6	1	NA	NA	NA	NA	NA
10	3	6	1	NA	NA	NA	NA	NA
10	4	6	1	NA	NA	NA	NA	NA
20	1	6	1	NA	NA	NA	NA	NA
20	2	6	1	NA	NA	NA	NA	NA
20	3	6	1	NA	NA	NA	NA	NA
20	4	6	1	NA	NA	NA	NA	NA
30	1	6	1	NA	NA	NA	NA	NA
30	2	6	1	NA	NA	NA	NA	NA
30	3	6	1	NA	NA	NA	NA	NA
30	4	6	1	NA	NA	NA	NA	NA
40	1	6	1	NA	NA	NA	NA	NA
40	2	6	1	NA	NA	NA	NA	NA
40	3	6	1	NA	NA	NA	NA	NA
40	4	6	1	NA	NA	NA	NA	NA
60	1	6	1	NA	NA	NA	NA	NA
10	1	6	2	NA	NA	NA	NA	NA
10	2	6	2	NA	NA	NA	NA	NA
10	3	6	2	NA	NA	NA	NA	NA
10	4	6	2	NA	NA	NA	NA	NA
20	1	6	2	NA	NA	NA	NA	NA
20	2	6	2	NA	NA	NA	NA	NA
20	3	6	2	NA	NA	NA	NA	NA
20	4	6	2	NA	NA	NA	NA	NA
30	1	6	2	NA	NA	NA	NA	NA
30	2	6	2	NA	NA	NA	NA	NA
30	3	6	2	NA	NA	NA	NA	NA
30	4	6	2	NA	NA	NA	NA	NA
40	1	6	2	NA	NA	NA	NA	NA
40	2	6	2	NA	NA	NA	NA	NA
40	3	6	2	NA	NA	NA	NA	NA
40	4	6	2	NA	NA	NA	NA	NA
60	1	6	2	NA	NA	NA	NA	NA
10	1	6	NA	NA	NA	NA	NA	NA
10	2	6	NA	NA	NA	NA	NA	NA
10	3	6	NA	NA	NA	NA	NA	NA

10	4	6	NA	NA	NA	NA	NA	NA
20	1	6	NA	NA	NA	NA	NA	NA
20	2	6	NA	NA	NA	NA	NA	NA
20	3	6	NA	NA	NA	NA	NA	NA
20	4	6	NA	NA	NA	NA	NA	NA
30	1	6	NA	NA	NA	NA	NA	NA
30	2	6	NA	NA	NA	NA	NA	NA
30	3	6	NA	NA	NA	NA	NA	NA
30	4	6	NA	NA	NA	NA	NA	NA
40	1	6	NA	NA	NA	NA	NA	NA
40	2	6	NA	NA	NA	NA	NA	NA
40	3	6	NA	NA	NA	NA	NA	NA
40	4	6	NA	NA	NA	NA	NA	NA
60	1	6	NA	NA	NA	NA	NA	NA
10	1	5	NA	NA	NA	NA	NA	NA
10	1	5	NA	NA	NA	NA	NA	NA
10	2	5	NA	NA	NA	NA	NA	NA
10	3	5	NA	NA	NA	NA	NA	NA
10	4	5	NA	NA	NA	NA	NA	NA
20	1	5	NA	NA	NA	NA	NA	NA
20	2	5	NA	NA	NA	NA	NA	NA
20	3	5	NA	NA	NA	NA	NA	NA
20	4	5	NA	NA	NA	NA	NA	NA
30	1	5	NA	NA	NA	NA	NA	NA
30	2	5	NA	NA	NA	NA	NA	NA
30	3	5	NA	NA	NA	NA	NA	NA
30	4	5	NA	NA	NA	NA	NA	NA
40	1	5	NA	NA	NA	NA	NA	NA
40	2	5	NA	NA	NA	NA	NA	NA
40	3	5	NA	NA	NA	NA	NA	NA
40	4	5	NA	NA	NA	NA	NA	NA
60	1	5	NA	NA	NA	NA	NA	NA
10	1	6	NA	NA	NA	NA	NA	NA
10	2	6	NA	NA	NA	NA	NA	NA
10	3	6	NA	NA	NA	NA	NA	NA
10	4	6	NA	NA	NA	NA	NA	NA
20	1	6	NA	NA	NA	NA	NA	NA

20	2	6	NA	NA	NA	NA	NA	NA
20	3	6	NA	NA	NA	NA	NA	NA
20	4	6	NA	NA	NA	NA	NA	NA
30	1	6	NA	NA	NA	NA	NA	NA
30	2	6	NA	NA	NA	NA	NA	NA
30	3	6	NA	NA	NA	NA	NA	NA
30	4	6	NA	NA	NA	NA	NA	NA
40	1	6	NA	NA	NA	NA	NA	NA
40	2	6	NA	NA	NA	NA	NA	NA
40	3	6	NA	NA	NA	NA	NA	NA
40	4	6	NA	NA	NA	NA	NA	NA
60	1	6	NA	NA	NA	NA	NA	NA
10	1	5	NA	NA	NA	NA	NA	NA
10	2	5	NA	NA	NA	NA	NA	NA
10	3	5	NA	NA	NA	NA	NA	NA
10	4	5	NA	NA	NA	NA	NA	NA
20	1	5	NA	NA	NA	NA	NA	NA
20	2	5	NA	NA	NA	NA	NA	NA
20	3	5	NA	NA	NA	NA	NA	NA
20	4	5	NA	NA	NA	NA	NA	NA
30	1	5	NA	NA	NA	NA	NA	NA
30	2	5	NA	NA	NA	NA	NA	NA
30	3	5	NA	NA	NA	NA	NA	NA
30	4	5	NA	NA	NA	NA	NA	NA
40	1	5	NA	NA	NA	NA	NA	NA
40	2	5	NA	NA	NA	NA	NA	NA
40	3	5	NA	NA	NA	NA	NA	NA
40	4	5	NA	NA	NA	NA	NA	NA
40	5	5	NA	NA	NA	NA	NA	NA
60	1	5	NA	NA	NA	NA	NA	NA
10	1	6	1	79.7	NA	50	17.4	NA
10	2	6	1	95.6	82.6	100	7.9	18.9
10	3	6	1	93.2	71.6	66.5	10.1	41.7
10	4	6	1	97.7	87.5.	100	4.6	9.6
20	1	6	1	83.9	68.6	43.8	32.7	32.3
20	2	6	1	82	85.6	89.4	22.1	20.8
20	3	6	1	82.2	81.2	85.7	21.4	20.2

20	4	6	1	89.2	93.6	100	9.1	9.4
30	1	6	1	85.3	65.5	0	10.7	36.1
30	2	6	1	58.5	82.4	NAP	34.5	51.2
30	3	6	1	72.6	63.2	65.8	17.3	45.1
30	4	6	1	82.9	70.9	62.3	15.8	32.2
40	1	6	1	64.2	NA	88.2	22.3	NA
40	2	6	1	71.1	68.3	0	30.1	33.6
40	3	6	1	68.3	64.6	NAP	31.1	32.4
40	4	6	1	67.2	81.4	50	39.6	33
60	1	6	1	55.2	96.3	100	27.1	9.1
10	1	5	NA	NA	NA	NA	NA	NA
10	2	5	NA	NA	NA	NA	NA	NA
10	3	5	NA	NA	NA	NA	NA	NA
10	4	5	NA	NA	NA	NA	NA	NA
20	1	5	NA	NA	NA	NA	NA	NA
20	2	5	NA	NA	NA	NA	NA	NA
20	3	5	NA	NA	NA	NA	NA	NA
20	4	5	NA	NA	NA	NA	NA	NA
30	1	5	NA	NA	NA	NA	NA	NA
30	2	5	NA	NA	NA	NA	NA	NA
30	3	5	NA	NA	NA	NA	NA	NA
30	4	5	NA	NA	NA	NA	NA	NA
40	1	5	NA	NA	NA	NA	NA	NA
40	2	5	NA	NA	NA	NA	NA	NA
40	3	5	NA	NA	NA	NA	NA	NA
40	4	5	NA	NA	NA	NA	NA	NA
40	5	5	NA	NA	NA	NA	NA	NA
60	1	5	NA	NA	NA	NA	NA	NA
10	1	5	NA	NA	NA	NA	NA	NA
10	2	5	NA	NA	NA	NA	NA	NA
10	3	5	NA	NA	NA	NA	NA	NA
10	4	5	NA	NA	NA	NA	NA	NA
20	1	5	NA	NA	NA	NA	NA	NA
20	2	5	NA	NA	NA	NA	NA	NA
20	3	5	NA	NA	NA	NA	NA	NA
20	4	5	NA	NA	NA	NA	NA	NA
30	1	5	NA	NA	NA	NA	NA	NA

30	2	5	NA	NA	NA	NA	NA	NA
30	3	5	NA	NA	NA	NA	NA	NA
30	4	5	NA	NA	NA	NA	NA	NA
40	1	5	NA	NA	NA	NA	NA	NA
40	2	5	NA	NA	NA	NA	NA	NA
40	3	5	NA	NA	NA	NA	NA	NA
40	4	5	NA	NA	NA	NA	NA	NA
40	5	5	NA	NA	NA	NA	NA	NA
60	1	5	NA	NA	NA	NA	NA	NA

Dens: density; Replic: replicate; Stage [Nymph 5 (= 5) or Adult (= 6)]; Sex (1 = females, 2 = males); “NA“ indicates data not applicable or not available for the given combination.

(Part 17)

Dens	Replic	Stage	Sex	Pct Hatch w3 sd	Eggs per Fem per life	R'o	BMS N D1	BMS mean mg D1
10	1	5	NA	NA	NA	NA	9	56.5
10	2	5	NA	NA	NA	NA	10	67.1
10	3	5	NA	NA	NA	NA	10	104.5
10	4	5	NA	NA	NA	NA	10	108.7
20	1	5	NA	NA	NA	NA	15	33.3
20	2	5	NA	NA	NA	NA	16	64.2
20	3	5	NA	NA	NA	NA	14	79.3
20	4	5	NA	NA	NA	NA	16	46.3
30	1	5	NA	NA	NA	NA	24	55.3
30	2	5	NA	NA	NA	NA	23	50.8
30	3	5	NA	NA	NA	NA	24	49.7
30	4	5	NA	NA	NA	NA	20	43.9
40	1	5	NA	NA	NA	NA	35	32.5
40	2	5	NA	NA	NA	NA	34	47.6
40	3	5	NA	NA	NA	NA	36	58.6
40	4	5	NA	NA	NA	NA	30	28.4
40	5	5	NA	NA	NA	NA	38	42.2
60	1	5	NA	NA	NA	NA	58	36.8
10	1	6	NA	NA	NA	NA	NA	NA
10	2	6	NA	NA	NA	NA	NA	NA
10	3	6	NA	NA	NA	NA	NA	NA
10	4	6	NA	NA	NA	NA	NA	NA
20	1	6	NA	NA	NA	NA	NA	NA
20	2	6	NA	NA	NA	NA	NA	NA
20	3	6	NA	NA	NA	NA	NA	NA
20	4	6	NA	NA	NA	NA	NA	NA
30	1	6	NA	NA	NA	NA	NA	NA
30	2	6	NA	NA	NA	NA	NA	NA
30	3	6	NA	NA	NA	NA	NA	NA
30	4	6	NA	NA	NA	NA	NA	NA
40	1	6	NA	NA	NA	NA	NA	NA
40	2	6	NA	NA	NA	NA	NA	NA
40	3	6	NA	NA	NA	NA	NA	NA
40	4	6	NA	NA	NA	NA	NA	NA

60	1	6	NA	NA	NA	NA	NA	NA
10	1	6	1	NA	NA	NA	6	47.5
10	2	6	1	NA	NA	NA	5	91.6
10	3	6	1	NA	NA	NA	5	39.9
10	4	6	1	NA	NA	NA	5	80.3
20	1	6	1	NA	NA	NA	10	68.8
20	2	6	1	NA	NA	NA	8	46.3
20	3	6	1	NA	NA	NA	10	29.7
20	4	6	1	NA	NA	NA	9	40.7
30	1	6	1	NA	NA	NA	13	18.2
30	2	6	1	NA	NA	NA	14	42.3
30	3	6	1	NA	NA	NA	11	36.8
30	4	6	1	NA	NA	NA	15	57.4
40	1	6	1	NA	NA	NA	16	18.6
40	2	6	1	NA	NA	NA	18	20.5
40	3	6	1	NA	NA	NA	15	19.7
40	4	6	1	NA	NA	NA	17	27.0
60	1	6	1	NA	NA	NA	22	25.9
10	1	6	2	NA	NA	NA	4	71.8
10	2	6	2	NA	NA	NA	5	49.6
10	3	6	2	NA	NA	NA	5	27.8
10	4	6	2	NA	NA	NA	3	36.5
20	1	6	2	NA	NA	NA	8	53.3
20	2	6	2	NA	NA	NA	6	61.3
20	3	6	2	NA	NA	NA	9	32.3
20	4	6	2	NA	NA	NA	10	39.4
30	1	6	2	NA	NA	NA	15	40.1
30	2	6	2	NA	NA	NA	15	31.4
30	3	6	2	NA	NA	NA	11	49.2
30	4	6	2	NA	NA	NA	NA	NA
40	1	6	2	NA	NA	NA	18	16.1
40	2	6	2	NA	NA	NA	18	16.1
40	3	6	2	NA	NA	NA	19	22.9
40	4	6	2	NA	NA	NA	17	34.3
60	1	6	2	NA	NA	NA	22	23.8
10	1	6	NA	NA	NA	NA	NA	NA
10	2	6	NA	NA	NA	NA	NA	NA

10	3	6	NA	NA	NA	NA	NA	NA
10	4	6	NA	NA	NA	NA	NA	NA
20	1	6	NA	NA	NA	NA	NA	NA
20	2	6	NA	NA	NA	NA	NA	NA
20	3	6	NA	NA	NA	NA	NA	NA
20	4	6	NA	NA	NA	NA	NA	NA
30	1	6	NA	NA	NA	NA	NA	NA
30	2	6	NA	NA	NA	NA	NA	NA
30	3	6	NA	NA	NA	NA	NA	NA
30	4	6	NA	NA	NA	NA	NA	NA
40	1	6	NA	NA	NA	NA	NA	NA
40	2	6	NA	NA	NA	NA	NA	NA
40	3	6	NA	NA	NA	NA	NA	NA
40	4	6	NA	NA	NA	NA	NA	NA
60	1	6	NA	NA	NA	NA	NA	NA
10	1	5	NA	NA	NA	NA	NA	NA
10	1	5	NA	NA	NA	NA	NA	NA
10	2	5	NA	NA	NA	NA	NA	NA
10	3	5	NA	NA	NA	NA	NA	NA
10	4	5	NA	NA	NA	NA	NA	NA
20	1	5	NA	NA	NA	NA	NA	NA
20	2	5	NA	NA	NA	NA	NA	NA
20	3	5	NA	NA	NA	NA	NA	NA
20	4	5	NA	NA	NA	NA	NA	NA
30	1	5	NA	NA	NA	NA	NA	NA
30	2	5	NA	NA	NA	NA	NA	NA
30	3	5	NA	NA	NA	NA	NA	NA
30	4	5	NA	NA	NA	NA	NA	NA
40	1	5	NA	NA	NA	NA	NA	NA
40	2	5	NA	NA	NA	NA	NA	NA
40	3	5	NA	NA	NA	NA	NA	NA
40	4	5	NA	NA	NA	NA	NA	NA
60	1	5	NA	NA	NA	NA	NA	NA
10	1	6	NA	NA	NA	NA	NA	NA
10	2	6	NA	NA	NA	NA	NA	NA
10	3	6	NA	NA	NA	NA	NA	NA
10	4	6	NA	NA	NA	NA	NA	NA

20	1	6	NA	NA	NA	NA	NA	NA
20	2	6	NA	NA	NA	NA	NA	NA
20	3	6	NA	NA	NA	NA	NA	NA
20	4	6	NA	NA	NA	NA	NA	NA
30	1	6	NA	NA	NA	NA	NA	NA
30	2	6	NA	NA	NA	NA	NA	NA
30	3	6	NA	NA	NA	NA	NA	NA
30	4	6	NA	NA	NA	NA	NA	NA
40	1	6	NA	NA	NA	NA	NA	NA
40	2	6	NA	NA	NA	NA	NA	NA
40	3	6	NA	NA	NA	NA	NA	NA
40	4	6	NA	NA	NA	NA	NA	NA
60	1	6	NA	NA	NA	NA	NA	NA
10	1	5	NA	NA	NA	NA	NA	NA
10	2	5	NA	NA	NA	NA	NA	NA
10	3	5	NA	NA	NA	NA	NA	NA
10	4	5	NA	NA	NA	NA	NA	NA
20	1	5	NA	NA	NA	NA	NA	NA
20	2	5	NA	NA	NA	NA	NA	NA
20	3	5	NA	NA	NA	NA	NA	NA
20	4	5	NA	NA	NA	NA	NA	NA
30	1	5	NA	NA	NA	NA	NA	NA
30	2	5	NA	NA	NA	NA	NA	NA
30	3	5	NA	NA	NA	NA	NA	NA
30	4	5	NA	NA	NA	NA	NA	NA
40	1	5	NA	NA	NA	NA	NA	NA
40	2	5	NA	NA	NA	NA	NA	NA
40	3	5	NA	NA	NA	NA	NA	NA
40	4	5	NA	NA	NA	NA	NA	NA
40	5	5	NA	NA	NA	NA	NA	NA
60	1	5	NA	NA	NA	NA	NA	NA
10	1	6	1	0	24	6.24	NA	NA
10	2	6	1	0	38.4	10.1	NA	NA
10	3	6	1	42.3	41.4	10.8	NA	NA
10	4	6	1	0	28.7	7.56	NA	NA
20	1	6	1	41.8	31.8	8.24	NA	NA
20	2	6	1	7.7	28.6	7.15	NA	NA

20	3	6	1	28.6	23.6	6.15	NA	NA
20	4	6	1	0	26.8	6.97	NA	NA
30	1	6	1	0	19.8	5.16	NA	NA
30	2	6	1	NAP	21.6	5.61	NA	NA
30	3	6	1	19.4	25.3	6.6	NA	NA
30	4	6	1	46.7	25.2	7.22	NA	NA
40	1	6	1	NA	10.1	2.12	NA	NA
40	2	6	1	0	11	2.68	NA	NA
40	3	6	1	NAP	8.9	2.18	NA	NA
40	4	6	1	50	15.6	4.08	NA	NA
60	1	6	1	0	11.1	2.93	NA	NA
10	1	5	NA	NA	NA	NA	NA	NA
10	2	5	NA	NA	NA	NA	NA	NA
10	3	5	NA	NA	NA	NA	NA	NA
10	4	5	NA	NA	NA	NA	NA	NA
20	1	5	NA	NA	NA	NA	NA	NA
20	2	5	NA	NA	NA	NA	NA	NA
20	3	5	NA	NA	NA	NA	NA	NA
20	4	5	NA	NA	NA	NA	NA	NA
30	1	5	NA	NA	NA	NA	NA	NA
30	2	5	NA	NA	NA	NA	NA	NA
30	3	5	NA	NA	NA	NA	NA	NA
30	4	5	NA	NA	NA	NA	NA	NA
40	1	5	NA	NA	NA	NA	NA	NA
40	2	5	NA	NA	NA	NA	NA	NA
40	3	5	NA	NA	NA	NA	NA	NA
40	4	5	NA	NA	NA	NA	NA	NA
40	5	5	NA	NA	NA	NA	NA	NA
60	1	5	NA	NA	NA	NA	NA	NA
10	1	5	NA	NA	NA	NA	NA	NA
10	2	5	NA	NA	NA	NA	NA	NA
10	3	5	NA	NA	NA	NA	NA	NA
10	4	5	NA	NA	NA	NA	NA	NA
20	1	5	NA	NA	NA	NA	NA	NA
20	2	5	NA	NA	NA	NA	NA	NA
20	3	5	NA	NA	NA	NA	NA	NA
20	4	5	NA	NA	NA	NA	NA	NA

30	1	5	NA	NA	NA	NA	NA	NA
30	2	5	NA	NA	NA	NA	NA	NA
30	3	5	NA	NA	NA	NA	NA	NA
30	4	5	NA	NA	NA	NA	NA	NA
40	1	5	NA	NA	NA	NA	NA	NA
40	2	5	NA	NA	NA	NA	NA	NA
40	3	5	NA	NA	NA	NA	NA	NA
40	4	5	NA	NA	NA	NA	NA	NA
40	5	5	NA	NA	NA	NA	NA	NA
60	1	5	NA	NA	NA	NA	NA	NA

Dens: density; Replic: replicate; Stage [Nymph 5 (= 5) or Adult (= 6)]; Sex (1 = females, 2 = males); “NA“ indicates data not applicable or not available for the given combination.

(Part 18)

Dens	Replic	Stage	Sex	BMS sd mg D1	BMS N D2	BMS mean mg D2	BMS sd mg D2	BMS N D3
10	1	5	NA	36.9	8	1.9	5.3	9
10	2	5	NA	34.4	10	0.8	2.7	10
10	3	5	NA	61.2	10	0	0	10
10	4	5	NA	18.0	8	0	0	8
20	1	5	NA	45.0	8	0	0	12
20	2	5	NA	9.4	6	0	0	10
20	3	5	NA	32.8	5	0	0	10
20	4	5	NA	18.4	9	6.7	20.2	13
30	1	5	NA	3.0	12	0.5	1.7	15
30	2	5	NA	30.7	7	0	0	13
30	3	5	NA	41.4	14	14.8	36.3	19
30	4	5	NA	30.3	11	0.5	1.6	15
40	1	5	NA	42.4	16	4.1	13.7	23
40	2	5	NA	23.8	21	17.6	33.3	26
40	3	5	NA	40.1	18	5.9	22.0	29
40	4	5	NA	44.7	22	24.8	42.3	25
40	5	5	NA	27.1	33	5.9	14.5	34
60	1	5	NA	29.6	52	11.3	28.2	56
10	1	6	NA	NA	NA	NA	NA	NA
10	2	6	NA	NA	NA	NA	NA	NA
10	3	6	NA	NA	NA	NA	NA	NA
10	4	6	NA	NA	NA	NA	NA	NA
20	1	6	NA	NA	NA	NA	NA	NA
20	2	6	NA	NA	NA	NA	NA	NA
20	3	6	NA	NA	NA	NA	NA	NA
20	4	6	NA	NA	NA	NA	NA	NA
30	1	6	NA	NA	NA	NA	NA	NA
30	2	6	NA	NA	NA	NA	NA	NA
30	3	6	NA	NA	NA	NA	NA	NA
30	4	6	NA	NA	NA	NA	NA	NA
40	1	6	NA	NA	NA	NA	NA	NA
40	2	6	NA	NA	NA	NA	NA	NA
40	3	6	NA	NA	NA	NA	NA	NA
40	4	6	NA	NA	NA	NA	NA	NA
60	1	6	NA	NA	NA	NA	NA	NA

10	1	6	1	39.0	6	12.3	30.0	6
10	2	6	1	23.6	5	0	0	5
10	3	6	1	48.0	5	47.7	67.9	5
10	4	6	1	29.9	4	0	0	3
20	1	6	1	40.1	10	18.4	41.0	8
20	2	6	1	46.4	8	5.3	14.9	8
20	3	6	1	40.9	10	22.8	41.8	10
20	4	6	1	41.2	8	11.0	25.8	7
30	1	6	1	27.2	12	38.8	42.0	11
30	2	6	1	39.4	11	22.4	33.9	9
30	3	6	1	31.2	9	19.1	38.0	9
30	4	6	1	51.1	11	36.9	49.3	11
40	1	6	1	27.8	15	3.8	9.1	14
40	2	6	1	28.3	18	13.8	25.9	18
40	3	6	1	24.2	15	9.8	29.4	14
40	4	6	1	33.1	15	27.3	36.5	14
60	1	6	1	38.5	17	13.7	29.8	14
10	1	6	2	12.1	4	0	0	4
10	2	6	2	33.0	5	2.8	6.3	5
10	3	6	2	26.1	5	11.2	25.1	5
10	4	6	2	38.7	3	0	0	2
20	1	6	2	30.0	8	0	0	7
20	2	6	2	46.6	6	0	0	6
20	3	6	2	34.4	9	10.2	21.9	9
20	4	6	2	36.7	9	24.6	37.9	9
30	1	6	2	32.6	14	6.5	18.6	14
30	2	6	2	32.2	13	8.9	21.9	12
30	3	6	2	40.3	11	12.5	28.3	10
30	4	6	2	NA	NA	NA	NA	NA
40	1	6	2	26.9	16	5.5	11.7	14
40	2	6	2	26.9	16	5.5	11.7	14
40	3	6	2	22.0	17	8.5	15.2	15
40	4	6	2	23.6	15	5.2	9.8	15
60	1	6	2	29.8	17	8.9	14.5	15
10	1	6	NA	NA	NA	NA	NA	NA
10	2	6	NA	NA	NA	NA	NA	NA
10	3	6	NA	NA	NA	NA	NA	NA

10	4	6	NA	NA	NA	NA	NA	NA
20	1	6	NA	NA	NA	NA	NA	NA
20	2	6	NA	NA	NA	NA	NA	NA
20	3	6	NA	NA	NA	NA	NA	NA
20	4	6	NA	NA	NA	NA	NA	NA
30	1	6	NA	NA	NA	NA	NA	NA
30	2	6	NA	NA	NA	NA	NA	NA
30	3	6	NA	NA	NA	NA	NA	NA
30	4	6	NA	NA	NA	NA	NA	NA
40	1	6	NA	NA	NA	NA	NA	NA
40	2	6	NA	NA	NA	NA	NA	NA
40	3	6	NA	NA	NA	NA	NA	NA
40	4	6	NA	NA	NA	NA	NA	NA
60	1	6	NA	NA	NA	NA	NA	NA
10	1	5	NA	NA	NA	NA	NA	NA
10	1	5	NA	NA	NA	NA	NA	NA
10	2	5	NA	NA	NA	NA	NA	NA
10	3	5	NA	NA	NA	NA	NA	NA
10	4	5	NA	NA	NA	NA	NA	NA
20	1	5	NA	NA	NA	NA	NA	NA
20	2	5	NA	NA	NA	NA	NA	NA
20	3	5	NA	NA	NA	NA	NA	NA
20	4	5	NA	NA	NA	NA	NA	NA
30	1	5	NA	NA	NA	NA	NA	NA
30	2	5	NA	NA	NA	NA	NA	NA
30	3	5	NA	NA	NA	NA	NA	NA
30	4	5	NA	NA	NA	NA	NA	NA
40	1	5	NA	NA	NA	NA	NA	NA
40	2	5	NA	NA	NA	NA	NA	NA
40	3	5	NA	NA	NA	NA	NA	NA
40	4	5	NA	NA	NA	NA	NA	NA
60	1	5	NA	NA	NA	NA	NA	NA
10	1	6	NA	NA	NA	NA	NA	NA
10	2	6	NA	NA	NA	NA	NA	NA
10	3	6	NA	NA	NA	NA	NA	NA
10	4	6	NA	NA	NA	NA	NA	NA
20	1	6	NA	NA	NA	NA	NA	NA

20	2	6	NA	NA	NA	NA	NA	NA
20	3	6	NA	NA	NA	NA	NA	NA
20	4	6	NA	NA	NA	NA	NA	NA
30	1	6	NA	NA	NA	NA	NA	NA
30	2	6	NA	NA	NA	NA	NA	NA
30	3	6	NA	NA	NA	NA	NA	NA
30	4	6	NA	NA	NA	NA	NA	NA
40	1	6	NA	NA	NA	NA	NA	NA
40	2	6	NA	NA	NA	NA	NA	NA
40	3	6	NA	NA	NA	NA	NA	NA
40	4	6	NA	NA	NA	NA	NA	NA
60	1	6	NA	NA	NA	NA	NA	NA
10	1	5	NA	NA	NA	NA	NA	NA
10	2	5	NA	NA	NA	NA	NA	NA
10	3	5	NA	NA	NA	NA	NA	NA
10	4	5	NA	NA	NA	NA	NA	NA
20	1	5	NA	NA	NA	NA	NA	NA
20	2	5	NA	NA	NA	NA	NA	NA
20	3	5	NA	NA	NA	NA	NA	NA
20	4	5	NA	NA	NA	NA	NA	NA
30	1	5	NA	NA	NA	NA	NA	NA
30	2	5	NA	NA	NA	NA	NA	NA
30	3	5	NA	NA	NA	NA	NA	NA
30	4	5	NA	NA	NA	NA	NA	NA
40	1	5	NA	NA	NA	NA	NA	NA
40	2	5	NA	NA	NA	NA	NA	NA
40	3	5	NA	NA	NA	NA	NA	NA
40	4	5	NA	NA	NA	NA	NA	NA
40	5	5	NA	NA	NA	NA	NA	NA
60	1	5	NA	NA	NA	NA	NA	NA
10	1	6	1	NA	NA	NA	NA	NA
10	2	6	1	NA	NA	NA	NA	NA
10	3	6	1	NA	NA	NA	NA	NA
10	4	6	1	NA	NA	NA	NA	NA
20	1	6	1	NA	NA	NA	NA	NA
20	2	6	1	NA	NA	NA	NA	NA
20	3	6	1	NA	NA	NA	NA	NA

20	4	6	1	NA	NA	NA	NA	NA
30	1	6	1	NA	NA	NA	NA	NA
30	2	6	1	NA	NA	NA	NA	NA
30	3	6	1	NA	NA	NA	NA	NA
30	4	6	1	NA	NA	NA	NA	NA
40	1	6	1	NA	NA	NA	NA	NA
40	2	6	1	NA	NA	NA	NA	NA
40	3	6	1	NA	NA	NA	NA	NA
40	4	6	1	NA	NA	NA	NA	NA
60	1	6	1	NA	NA	NA	NA	NA
10	1	5	NA	NA	NA	NA	NA	NA
10	2	5	NA	NA	NA	NA	NA	NA
10	3	5	NA	NA	NA	NA	NA	NA
10	4	5	NA	NA	NA	NA	NA	NA
20	1	5	NA	NA	NA	NA	NA	NA
20	2	5	NA	NA	NA	NA	NA	NA
20	3	5	NA	NA	NA	NA	NA	NA
20	4	5	NA	NA	NA	NA	NA	NA
30	1	5	NA	NA	NA	NA	NA	NA
30	2	5	NA	NA	NA	NA	NA	NA
30	3	5	NA	NA	NA	NA	NA	NA
30	4	5	NA	NA	NA	NA	NA	NA
40	1	5	NA	NA	NA	NA	NA	NA
40	2	5	NA	NA	NA	NA	NA	NA
40	3	5	NA	NA	NA	NA	NA	NA
40	4	5	NA	NA	NA	NA	NA	NA
40	5	5	NA	NA	NA	NA	NA	NA
60	1	5	NA	NA	NA	NA	NA	NA
10	1	5	NA	NA	NA	NA	NA	NA
10	2	5	NA	NA	NA	NA	NA	NA
10	3	5	NA	NA	NA	NA	NA	NA
10	4	5	NA	NA	NA	NA	NA	NA
20	1	5	NA	NA	NA	NA	NA	NA
20	2	5	NA	NA	NA	NA	NA	NA
20	3	5	NA	NA	NA	NA	NA	NA
20	4	5	NA	NA	NA	NA	NA	NA
30	1	5	NA	NA	NA	NA	NA	NA

30	2	5	NA	NA	NA	NA	NA	NA
30	3	5	NA	NA	NA	NA	NA	NA
30	4	5	NA	NA	NA	NA	NA	NA
40	1	5	NA	NA	NA	NA	NA	NA
40	2	5	NA	NA	NA	NA	NA	NA
40	3	5	NA	NA	NA	NA	NA	NA
40	4	5	NA	NA	NA	NA	NA	NA
40	5	5	NA	NA	NA	NA	NA	NA
60	1	5	NA	NA	NA	NA	NA	NA

Dens: density; Replic: replicate; Stage [Nymph 5 (= 5) or Adult (= 6)]; Sex (1 = females, 2 = males); “NA“ indicates data not applicable or not available for the given combination.

(Part 19)

Dens	Replic	Stage	Sex	BMS mean mg D3	BMS sd mg D3	BMS mg D1	BMS mg D2	BMS mg D3
10	1	5	NA	12.3	46.3	NA	NA	NA
10	2	5	NA	31.0	51.8	NA	NA	NA
10	3	5	NA	29.0	61.8	NA	NA	NA
10	4	5	NA	7.3	45.8	NA	NA	NA
20	1	5	NA	24.5	49.1	NA	NA	NA
20	2	5	NA	3.0	66.6	NA	NA	NA
20	3	5	NA	12.9	65.6	NA	NA	NA
20	4	5	NA	7.5	44.1	NA	NA	NA
30	1	5	NA	0.8	54.3	NA	NA	NA
30	2	5	NA	11.5	68.6	NA	NA	NA
30	3	5	NA	18.8	48.0	NA	NA	NA
30	4	5	NA	17.2	47.0	NA	NA	NA
40	1	5	NA	23.4	35.5	NA	NA	NA
40	2	5	NA	13.5	44.7	NA	NA	NA
40	3	5	NA	12.9	57.0	NA	NA	NA
40	4	5	NA	22.7	35.3	NA	NA	NA
40	5	5	NA	11.9	52.4	NA	NA	NA
60	1	5	NA	14.9	42.8	NA	NA	NA
10	1	6	NA	NA	NA	NA	NA	NA
10	2	6	NA	NA	NA	NA	NA	NA
10	3	6	NA	NA	NA	NA	NA	NA
10	4	6	NA	NA	NA	NA	NA	NA
20	1	6	NA	NA	NA	NA	NA	NA
20	2	6	NA	NA	NA	NA	NA	NA
20	3	6	NA	NA	NA	NA	NA	NA
20	4	6	NA	NA	NA	NA	NA	NA
30	1	6	NA	NA	NA	NA	NA	NA
30	2	6	NA	NA	NA	NA	NA	NA
30	3	6	NA	NA	NA	NA	NA	NA
30	4	6	NA	NA	NA	NA	NA	NA
40	1	6	NA	NA	NA	NA	NA	NA
40	2	6	NA	NA	NA	NA	NA	NA
40	3	6	NA	NA	NA	NA	NA	NA
40	4	6	NA	NA	NA	NA	NA	NA
60	1	6	NA	NA	NA	NA	NA	NA

10	1	6	1	0	0	NA	NA	NA
10	2	6	1	0	0	NA	NA	NA
10	3	6	1	17.4	38.9	NA	NA	NA
10	4	6	1	0	0	NA	NA	NA
20	1	6	1	0	0	NA	NA	NA
20	2	6	1	0.3	0.8	NA	NA	NA
20	3	6	1	5.5	17.2	NA	NA	NA
20	4	6	1	11.7	30.8	NA	NA	NA
30	1	6	1	0	0	NA	NA	NA
30	2	6	1	6.1	18.3	NA	NA	NA
30	3	6	1	10.4	26.8	NA	NA	NA
30	4	6	1	2.0	5.0	NA	NA	NA
40	1	6	1	5.1	8.9	NA	NA	NA
40	2	6	1	5.2	12.4	NA	NA	NA
40	3	6	1	3.6	13.6	NA	NA	NA
40	4	6	1	4.7	14.7	NA	NA	NA
60	1	6	1	6.0	15.3	NA	NA	NA
10	1	6	2	0	0	NA	NA	NA
10	2	6	2	0	0	NA	NA	NA
10	3	6	2	18.3	40.8	NA	NA	NA
10	4	6	2	31.5	44.5	NA	NA	NA
20	1	6	2	18.8	32.2	NA	NA	NA
20	2	6	2	7.4	18.2	NA	NA	NA
20	3	6	2	0	0	NA	NA	NA
20	4	6	2	0	0	NA	NA	NA
30	1	6	2	0.9	3.5	NA	NA	NA
30	2	6	2	3.0	10.3	NA	NA	NA
30	3	6	2	1.3	4.2	NA	NA	NA
30	4	6	2	NA	NA	NA	NA	NA
40	1	6	2	6.8	14.8	NA	NA	NA
40	2	6	2	6.8	14.8	NA	NA	NA
40	3	6	2	0.3	1.3	NA	NA	NA
40	4	6	2	6.3	14.7	NA	NA	NA
60	1	6	2	12.2	22.3	NA	NA	NA
10	1	6	NA	NA	NA	NA	NA	NA
10	2	6	NA	NA	NA	NA	NA	NA
10	3	6	NA	NA	NA	NA	NA	NA

10	4	6	NA	NA	NA	NA	NA	NA
20	1	6	NA	NA	NA	NA	NA	NA
20	2	6	NA	NA	NA	NA	NA	NA
20	3	6	NA	NA	NA	NA	NA	NA
20	4	6	NA	NA	NA	NA	NA	NA
30	1	6	NA	NA	NA	NA	NA	NA
30	2	6	NA	NA	NA	NA	NA	NA
30	3	6	NA	NA	NA	NA	NA	NA
30	4	6	NA	NA	NA	NA	NA	NA
40	1	6	NA	NA	NA	NA	NA	NA
40	2	6	NA	NA	NA	NA	NA	NA
40	3	6	NA	NA	NA	NA	NA	NA
40	4	6	NA	NA	NA	NA	NA	NA
60	1	6	NA	NA	NA	NA	NA	NA
10	1	5	NA	NA	NA	NA	NA	NA
10	1	5	NA	NA	NA	NA	NA	NA
10	2	5	NA	NA	NA	NA	NA	NA
10	3	5	NA	NA	NA	NA	NA	NA
10	4	5	NA	NA	NA	NA	NA	NA
20	1	5	NA	NA	NA	NA	NA	NA
20	2	5	NA	NA	NA	NA	NA	NA
20	3	5	NA	NA	NA	NA	NA	NA
20	4	5	NA	NA	NA	NA	NA	NA
30	1	5	NA	NA	NA	NA	NA	NA
30	2	5	NA	NA	NA	NA	NA	NA
30	3	5	NA	NA	NA	NA	NA	NA
30	4	5	NA	NA	NA	NA	NA	NA
40	1	5	NA	NA	NA	NA	NA	NA
40	2	5	NA	NA	NA	NA	NA	NA
40	3	5	NA	NA	NA	NA	NA	NA
40	4	5	NA	NA	NA	NA	NA	NA
60	1	5	NA	NA	NA	NA	NA	NA
10	1	6	NA	NA	NA	NA	NA	NA
10	2	6	NA	NA	NA	NA	NA	NA
10	3	6	NA	NA	NA	NA	NA	NA
10	4	6	NA	NA	NA	NA	NA	NA
20	1	6	NA	NA	NA	NA	NA	NA

20	2	6	NA	NA	NA	NA	NA	NA
20	3	6	NA	NA	NA	NA	NA	NA
20	4	6	NA	NA	NA	NA	NA	NA
30	1	6	NA	NA	NA	NA	NA	NA
30	2	6	NA	NA	NA	NA	NA	NA
30	3	6	NA	NA	NA	NA	NA	NA
30	4	6	NA	NA	NA	NA	NA	NA
40	1	6	NA	NA	NA	NA	NA	NA
40	2	6	NA	NA	NA	NA	NA	NA
40	3	6	NA	NA	NA	NA	NA	NA
40	4	6	NA	NA	NA	NA	NA	NA
60	1	6	NA	NA	NA	NA	NA	NA
10	1	5	NA	NA	NA	NA	NA	NA
10	2	5	NA	NA	NA	NA	NA	NA
10	3	5	NA	NA	NA	NA	NA	NA
10	4	5	NA	NA	NA	NA	NA	NA
20	1	5	NA	NA	NA	NA	NA	NA
20	2	5	NA	NA	NA	NA	NA	NA
20	3	5	NA	NA	NA	NA	NA	NA
20	4	5	NA	NA	NA	NA	NA	NA
30	1	5	NA	NA	NA	NA	NA	NA
30	2	5	NA	NA	NA	NA	NA	NA
30	3	5	NA	NA	NA	NA	NA	NA
30	4	5	NA	NA	NA	NA	NA	NA
40	1	5	NA	NA	NA	NA	NA	NA
40	2	5	NA	NA	NA	NA	NA	NA
40	3	5	NA	NA	NA	NA	NA	NA
40	4	5	NA	NA	NA	NA	NA	NA
40	5	5	NA	NA	NA	NA	NA	NA
60	1	5	NA	NA	NA	NA	NA	NA
10	1	6	1	NA	NA	NA	NA	NA
10	2	6	1	NA	NA	NA	NA	NA
10	3	6	1	NA	NA	NA	NA	NA
10	4	6	1	NA	NA	NA	NA	NA
20	1	6	1	NA	NA	NA	NA	NA
20	2	6	1	NA	NA	NA	NA	NA
20	3	6	1	NA	NA	NA	NA	NA

20	4	6	1	NA	NA	NA	NA	NA
30	1	6	1	NA	NA	NA	NA	NA
30	2	6	1	NA	NA	NA	NA	NA
30	3	6	1	NA	NA	NA	NA	NA
30	4	6	1	NA	NA	NA	NA	NA
40	1	6	1	NA	NA	NA	NA	NA
40	2	6	1	NA	NA	NA	NA	NA
40	3	6	1	NA	NA	NA	NA	NA
40	4	6	1	NA	NA	NA	NA	NA
60	1	6	1	NA	NA	NA	NA	NA
10	1	5	NA	NA	NA	72.7	110.7	14.9
10	2	5	NA	NA	NA	83.9	51.7	8.4
10	3	5	NA	NA	NA	130.6	96.8	0
10	4	5	NA	NA	NA	120.7	29	0
20	1	5	NA	NA	NA	71.4	73.5	0
20	2	5	NA	NA	NA	93.4	29.6	0
20	3	5	NA	NA	NA	86.3	64.7	0
20	4	5	NA	NA	NA	67.4	49	60.7
30	1	5	NA	NA	NA	74.4	11.6	6
30	2	5	NA	NA	NA	85.4	37.8	0
30	3	5	NA	NA	NA	70.2	59.5	69.1
30	4	5	NA	NA	NA	62.7	55.5	5.2
40	1	5	NA	NA	NA	45.5	53.9	33
40	2	5	NA	NA	NA	56.5	38.9	61.5
40	3	5	NA	NA	NA	70.4	53.6	53.1
40	4	5	NA	NA	NA	42.7	63.3	68.2
40	5	5	NA	NA	NA	72.9	40.5	27.7
60	1	5	NA	NA	NA	54.2	43.8	49.2
10	1	5	NA	NA	NA	NA	NA	NA
10	2	5	NA	NA	NA	NA	NA	NA
10	3	5	NA	NA	NA	NA	NA	NA
10	4	5	NA	NA	NA	NA	NA	NA
20	1	5	NA	NA	NA	NA	NA	NA
20	2	5	NA	NA	NA	NA	NA	NA
20	3	5	NA	NA	NA	NA	NA	NA
20	4	5	NA	NA	NA	NA	NA	NA
30	1	5	NA	NA	NA	NA	NA	NA

30	2	5	NA	NA	NA	NA	NA	NA
30	3	5	NA	NA	NA	NA	NA	NA
30	4	5	NA	NA	NA	NA	NA	NA
40	1	5	NA	NA	NA	NA	NA	NA
40	2	5	NA	NA	NA	NA	NA	NA
40	3	5	NA	NA	NA	NA	NA	NA
40	4	5	NA	NA	NA	NA	NA	NA
40	5	5	NA	NA	NA	NA	NA	NA
60	1	5	NA	NA	NA	NA	NA	NA

Dens: density; Replic: replicate; Stage [Nymph 5 (= 5) or Adult (= 6)]; Sex (1 = females, 2 = males); “NA“ indicates data not applicable or not available for the given combination.

(Part 20)

Dens	Replic	Stage	Sex	Tot BMS mg 3 Days	Mean BMS mg 3 Days	BMS mg NOT Disp D1	BMS mg NOT Disp D2	BMS mg NOT Disp D3
10	1	5	NA	NA	NA	NA	NA	NA
10	2	5	NA	NA	NA	NA	NA	NA
10	3	5	NA	NA	NA	NA	NA	NA
10	4	5	NA	NA	NA	NA	NA	NA
20	1	5	NA	NA	NA	NA	NA	NA
20	2	5	NA	NA	NA	NA	NA	NA
20	3	5	NA	NA	NA	NA	NA	NA
20	4	5	NA	NA	NA	NA	NA	NA
30	1	5	NA	NA	NA	NA	NA	NA
30	2	5	NA	NA	NA	NA	NA	NA
30	3	5	NA	NA	NA	NA	NA	NA
30	4	5	NA	NA	NA	NA	NA	NA
40	1	5	NA	NA	NA	NA	NA	NA
40	2	5	NA	NA	NA	NA	NA	NA
40	3	5	NA	NA	NA	NA	NA	NA
40	4	5	NA	NA	NA	NA	NA	NA
40	5	5	NA	NA	NA	NA	NA	NA
60	1	5	NA	NA	NA	NA	NA	NA
10	1	6	NA	NA	NA	NA	NA	NA
10	2	6	NA	NA	NA	NA	NA	NA
10	3	6	NA	NA	NA	NA	NA	NA
10	4	6	NA	NA	NA	NA	NA	NA
20	1	6	NA	NA	NA	NA	NA	NA
20	2	6	NA	NA	NA	NA	NA	NA
20	3	6	NA	NA	NA	NA	NA	NA
20	4	6	NA	NA	NA	NA	NA	NA
30	1	6	NA	NA	NA	NA	NA	NA
30	2	6	NA	NA	NA	NA	NA	NA
30	3	6	NA	NA	NA	NA	NA	NA
30	4	6	NA	NA	NA	NA	NA	NA
40	1	6	NA	NA	NA	NA	NA	NA
40	2	6	NA	NA	NA	NA	NA	NA
40	3	6	NA	NA	NA	NA	NA	NA

40	4	6	NA	NA	NA	NA	NA	NA
60	1	6	NA	NA	NA	NA	NA	NA
10	1	6	1	NA	NA	NA	NA	NA
10	2	6	1	NA	NA	NA	NA	NA
10	3	6	1	NA	NA	NA	NA	NA
10	4	6	1	NA	NA	NA	NA	NA
20	1	6	1	NA	NA	NA	NA	NA
20	2	6	1	NA	NA	NA	NA	NA
20	3	6	1	NA	NA	NA	NA	NA
20	4	6	1	NA	NA	NA	NA	NA
30	1	6	1	NA	NA	NA	NA	NA
30	2	6	1	NA	NA	NA	NA	NA
30	3	6	1	NA	NA	NA	NA	NA
30	4	6	1	NA	NA	NA	NA	NA
40	1	6	1	NA	NA	NA	NA	NA
40	2	6	1	NA	NA	NA	NA	NA
40	3	6	1	NA	NA	NA	NA	NA
40	4	6	1	NA	NA	NA	NA	NA
60	1	6	1	NA	NA	NA	NA	NA
10	1	6	2	NA	NA	NA	NA	NA
10	2	6	2	NA	NA	NA	NA	NA
10	3	6	2	NA	NA	NA	NA	NA
10	4	6	2	NA	NA	NA	NA	NA
20	1	6	2	NA	NA	NA	NA	NA
20	2	6	2	NA	NA	NA	NA	NA
20	3	6	2	NA	NA	NA	NA	NA
20	4	6	2	NA	NA	NA	NA	NA
30	1	6	2	NA	NA	NA	NA	NA
30	2	6	2	NA	NA	NA	NA	NA
30	3	6	2	NA	NA	NA	NA	NA
30	4	6	2	NA	NA	NA	NA	NA
40	1	6	2	NA	NA	NA	NA	NA
40	2	6	2	NA	NA	NA	NA	NA
40	3	6	2	NA	NA	NA	NA	NA
40	4	6	2	NA	NA	NA	NA	NA
60	1	6	2	NA	NA	NA	NA	NA
10	1	6	NA	NA	NA	NA	NA	NA

10	2	6	NA	NA	NA	NA	NA	NA
10	3	6	NA	NA	NA	NA	NA	NA
10	4	6	NA	NA	NA	NA	NA	NA
20	1	6	NA	NA	NA	NA	NA	NA
20	2	6	NA	NA	NA	NA	NA	NA
20	3	6	NA	NA	NA	NA	NA	NA
20	4	6	NA	NA	NA	NA	NA	NA
30	1	6	NA	NA	NA	NA	NA	NA
30	2	6	NA	NA	NA	NA	NA	NA
30	3	6	NA	NA	NA	NA	NA	NA
30	4	6	NA	NA	NA	NA	NA	NA
40	1	6	NA	NA	NA	NA	NA	NA
40	2	6	NA	NA	NA	NA	NA	NA
40	3	6	NA	NA	NA	NA	NA	NA
40	4	6	NA	NA	NA	NA	NA	NA
60	1	6	NA	NA	NA	NA	NA	NA
10	1	5	NA	NA	NA	NA	NA	NA
10	1	5	NA	NA	NA	NA	NA	NA
10	2	5	NA	NA	NA	NA	NA	NA
10	3	5	NA	NA	NA	NA	NA	NA
10	4	5	NA	NA	NA	NA	NA	NA
20	1	5	NA	NA	NA	NA	NA	NA
20	2	5	NA	NA	NA	NA	NA	NA
20	3	5	NA	NA	NA	NA	NA	NA
20	4	5	NA	NA	NA	NA	NA	NA
30	1	5	NA	NA	NA	NA	NA	NA
30	2	5	NA	NA	NA	NA	NA	NA
30	3	5	NA	NA	NA	NA	NA	NA
30	4	5	NA	NA	NA	NA	NA	NA
40	1	5	NA	NA	NA	NA	NA	NA
40	2	5	NA	NA	NA	NA	NA	NA
40	3	5	NA	NA	NA	NA	NA	NA
40	4	5	NA	NA	NA	NA	NA	NA
60	1	5	NA	NA	NA	NA	NA	NA
10	1	6	NA	NA	NA	NA	NA	NA
10	2	6	NA	NA	NA	NA	NA	NA
10	3	6	NA	NA	NA	NA	NA	NA

10	4	6	NA	NA	NA	NA	NA	NA
20	1	6	NA	NA	NA	NA	NA	NA
20	2	6	NA	NA	NA	NA	NA	NA
20	3	6	NA	NA	NA	NA	NA	NA
20	4	6	NA	NA	NA	NA	NA	NA
30	1	6	NA	NA	NA	NA	NA	NA
30	2	6	NA	NA	NA	NA	NA	NA
30	3	6	NA	NA	NA	NA	NA	NA
30	4	6	NA	NA	NA	NA	NA	NA
40	1	6	NA	NA	NA	NA	NA	NA
40	2	6	NA	NA	NA	NA	NA	NA
40	3	6	NA	NA	NA	NA	NA	NA
40	4	6	NA	NA	NA	NA	NA	NA
60	1	6	NA	NA	NA	NA	NA	NA
10	1	5	NA	NA	NA	NA	NA	NA
10	2	5	NA	NA	NA	NA	NA	NA
10	3	5	NA	NA	NA	NA	NA	NA
10	4	5	NA	NA	NA	NA	NA	NA
20	1	5	NA	NA	NA	NA	NA	NA
20	2	5	NA	NA	NA	NA	NA	NA
20	3	5	NA	NA	NA	NA	NA	NA
20	4	5	NA	NA	NA	NA	NA	NA
30	1	5	NA	NA	NA	NA	NA	NA
30	2	5	NA	NA	NA	NA	NA	NA
30	3	5	NA	NA	NA	NA	NA	NA
30	4	5	NA	NA	NA	NA	NA	NA
40	1	5	NA	NA	NA	NA	NA	NA
40	2	5	NA	NA	NA	NA	NA	NA
40	3	5	NA	NA	NA	NA	NA	NA
40	4	5	NA	NA	NA	NA	NA	NA
40	5	5	NA	NA	NA	NA	NA	NA
60	1	5	NA	NA	NA	NA	NA	NA
10	1	6	1	NA	NA	NA	NA	NA
10	2	6	1	NA	NA	NA	NA	NA
10	3	6	1	NA	NA	NA	NA	NA
10	4	6	1	NA	NA	NA	NA	NA
20	1	6	1	NA	NA	NA	NA	NA

20	2	6	1	NA	NA	NA	NA	NA
20	3	6	1	NA	NA	NA	NA	NA
20	4	6	1	NA	NA	NA	NA	NA
30	1	6	1	NA	NA	NA	NA	NA
30	2	6	1	NA	NA	NA	NA	NA
30	3	6	1	NA	NA	NA	NA	NA
30	4	6	1	NA	NA	NA	NA	NA
40	1	6	1	NA	NA	NA	NA	NA
40	2	6	1	NA	NA	NA	NA	NA
40	3	6	1	NA	NA	NA	NA	NA
40	4	6	1	NA	NA	NA	NA	NA
60	1	6	1	NA	NA	NA	NA	NA
10	1	5	NA	198.3	66.1	72.7	110.7	14.9
10	2	5	NA	144	48.0	83.9	54.0	8.4
10	3	5	NA	227.4	75.8	130.6	60.2	0.0
10	4	5	NA	149.7	49.9	115.0	29.0	0.0
20	1	5	NA	144.9	48.3	50.3	73.5	0.0
20	2	5	NA	123	41.0	73.9	29.6	0.0
20	3	5	NA	151	50.3	101.7	64.7	0.0
20	4	5	NA	177.1	59.0	75.4	49.0	60.7
30	1	5	NA	92	30.7	55.7	0.0	6.0
30	2	5	NA	123.2	41.1	80.5	9.7	0.0
30	3	5	NA	198.8	66.3	73.8	68.2	84.3
30	4	5	NA	123.4	41.1	60.9	53.3	5.2
40	1	5	NA	132.4	44.1	41.9	58.9	33.0
40	2	5	NA	156.9	52.3	61.6	39.0	61.5
40	3	5	NA	177.1	59.0	73.0	36.4	53.1
40	4	5	NA	174.2	58.1	44.3	72.4	71.1
40	5	5	NA	141.1	47.0	73.9	41.1	27.7
60	1	5	NA	147.2	49.1	52.7	50.4	42.9
10	1	5	NA	NA	NA	NA	NA	NA
10	2	5	NA	NA	NA	NA	NA	NA
10	3	5	NA	NA	NA	NA	NA	NA
10	4	5	NA	NA	NA	NA	NA	NA
20	1	5	NA	NA	NA	NA	NA	NA
20	2	5	NA	NA	NA	NA	NA	NA
20	3	5	NA	NA	NA	NA	NA	NA

20	4	5	NA	NA	NA	NA	NA	NA
30	1	5	NA	NA	NA	NA	NA	NA
30	2	5	NA	NA	NA	NA	NA	NA
30	3	5	NA	NA	NA	NA	NA	NA
30	4	5	NA	NA	NA	NA	NA	NA
40	1	5	NA	NA	NA	NA	NA	NA
40	2	5	NA	NA	NA	NA	NA	NA
40	3	5	NA	NA	NA	NA	NA	NA
40	4	5	NA	NA	NA	NA	NA	NA
40	5	5	NA	NA	NA	NA	NA	NA
60	1	5	NA	NA	NA	NA	NA	NA

Dens: density; Replic: replicate; Stage [Nymph 5 (= 5) or Adult (= 6)]; Sex (1 = females, 2 = males); "NA" indicates data not applicable or not available for the given combination.

(Part 21)

Dens	Replic	Stage	Sex	BMS mg NOT Disp 3 days	BMS mg Disp D1	BMS mg Disp D2	BMS mg Disp D3	BMS mg Disp 3 days
10	1	5	NA	NA	NA	NA	NA	NA
10	2	5	NA	NA	NA	NA	NA	NA
10	3	5	NA	NA	NA	NA	NA	NA
10	4	5	NA	NA	NA	NA	NA	NA
20	1	5	NA	NA	NA	NA	NA	NA
20	2	5	NA	NA	NA	NA	NA	NA
20	3	5	NA	NA	NA	NA	NA	NA
20	4	5	NA	NA	NA	NA	NA	NA
30	1	5	NA	NA	NA	NA	NA	NA
30	2	5	NA	NA	NA	NA	NA	NA
30	3	5	NA	NA	NA	NA	NA	NA
30	4	5	NA	NA	NA	NA	NA	NA
40	1	5	NA	NA	NA	NA	NA	NA
40	2	5	NA	NA	NA	NA	NA	NA
40	3	5	NA	NA	NA	NA	NA	NA
40	4	5	NA	NA	NA	NA	NA	NA
40	5	5	NA	NA	NA	NA	NA	NA
60	1	5	NA	NA	NA	NA	NA	NA
10	1	6	NA	NA	NA	NA	NA	NA
10	2	6	NA	NA	NA	NA	NA	NA
10	3	6	NA	NA	NA	NA	NA	NA
10	4	6	NA	NA	NA	NA	NA	NA
20	1	6	NA	NA	NA	NA	NA	NA
20	2	6	NA	NA	NA	NA	NA	NA
20	3	6	NA	NA	NA	NA	NA	NA
20	4	6	NA	NA	NA	NA	NA	NA
30	1	6	NA	NA	NA	NA	NA	NA
30	2	6	NA	NA	NA	NA	NA	NA
30	3	6	NA	NA	NA	NA	NA	NA
30	4	6	NA	NA	NA	NA	NA	NA
40	1	6	NA	NA	NA	NA	NA	NA
40	2	6	NA	NA	NA	NA	NA	NA
40	3	6	NA	NA	NA	NA	NA	NA
40	4	6	NA	NA	NA	NA	NA	NA

60	1	6	NA	NA	NA	NA	NA	NA
10	1	6	1	NA	NA	NA	NA	NA
10	2	6	1	NA	NA	NA	NA	NA
10	3	6	1	NA	NA	NA	NA	NA
10	4	6	1	NA	NA	NA	NA	NA
20	1	6	1	NA	NA	NA	NA	NA
20	2	6	1	NA	NA	NA	NA	NA
20	3	6	1	NA	NA	NA	NA	NA
20	4	6	1	NA	NA	NA	NA	NA
30	1	6	1	NA	NA	NA	NA	NA
30	2	6	1	NA	NA	NA	NA	NA
30	3	6	1	NA	NA	NA	NA	NA
30	4	6	1	NA	NA	NA	NA	NA
40	1	6	1	NA	NA	NA	NA	NA
40	2	6	1	NA	NA	NA	NA	NA
40	3	6	1	NA	NA	NA	NA	NA
40	4	6	1	NA	NA	NA	NA	NA
60	1	6	1	NA	NA	NA	NA	NA
10	1	6	2	NA	NA	NA	NA	NA
10	2	6	2	NA	NA	NA	NA	NA
10	3	6	2	NA	NA	NA	NA	NA
10	4	6	2	NA	NA	NA	NA	NA
20	1	6	2	NA	NA	NA	NA	NA
20	2	6	2	NA	NA	NA	NA	NA
20	3	6	2	NA	NA	NA	NA	NA
20	4	6	2	NA	NA	NA	NA	NA
30	1	6	2	NA	NA	NA	NA	NA
30	2	6	2	NA	NA	NA	NA	NA
30	3	6	2	NA	NA	NA	NA	NA
30	4	6	2	NA	NA	NA	NA	NA
40	1	6	2	NA	NA	NA	NA	NA
40	2	6	2	NA	NA	NA	NA	NA
40	3	6	2	NA	NA	NA	NA	NA
40	4	6	2	NA	NA	NA	NA	NA
60	1	6	2	NA	NA	NA	NA	NA
10	1	6	NA	NA	NA	NA	NA	NA
10	2	6	NA	NA	NA	NA	NA	NA

10	3	6	NA	NA	NA	NA	NA	NA
10	4	6	NA	NA	NA	NA	NA	NA
20	1	6	NA	NA	NA	NA	NA	NA
20	2	6	NA	NA	NA	NA	NA	NA
20	3	6	NA	NA	NA	NA	NA	NA
20	4	6	NA	NA	NA	NA	NA	NA
30	1	6	NA	NA	NA	NA	NA	NA
30	2	6	NA	NA	NA	NA	NA	NA
30	3	6	NA	NA	NA	NA	NA	NA
30	4	6	NA	NA	NA	NA	NA	NA
40	1	6	NA	NA	NA	NA	NA	NA
40	2	6	NA	NA	NA	NA	NA	NA
40	3	6	NA	NA	NA	NA	NA	NA
40	4	6	NA	NA	NA	NA	NA	NA
60	1	6	NA	NA	NA	NA	NA	NA
10	1	5	NA	NA	NA	NA	NA	NA
10	1	5	NA	NA	NA	NA	NA	NA
10	2	5	NA	NA	NA	NA	NA	NA
10	3	5	NA	NA	NA	NA	NA	NA
10	4	5	NA	NA	NA	NA	NA	NA
20	1	5	NA	NA	NA	NA	NA	NA
20	2	5	NA	NA	NA	NA	NA	NA
20	3	5	NA	NA	NA	NA	NA	NA
20	4	5	NA	NA	NA	NA	NA	NA
30	1	5	NA	NA	NA	NA	NA	NA
30	2	5	NA	NA	NA	NA	NA	NA
30	3	5	NA	NA	NA	NA	NA	NA
30	4	5	NA	NA	NA	NA	NA	NA
40	1	5	NA	NA	NA	NA	NA	NA
40	2	5	NA	NA	NA	NA	NA	NA
40	3	5	NA	NA	NA	NA	NA	NA
40	4	5	NA	NA	NA	NA	NA	NA
60	1	5	NA	NA	NA	NA	NA	NA
10	1	6	NA	NA	NA	NA	NA	NA
10	2	6	NA	NA	NA	NA	NA	NA
10	3	6	NA	NA	NA	NA	NA	NA
10	4	6	NA	NA	NA	NA	NA	NA

20	1	6	NA	NA	NA	NA	NA	NA
20	2	6	NA	NA	NA	NA	NA	NA
20	3	6	NA	NA	NA	NA	NA	NA
20	4	6	NA	NA	NA	NA	NA	NA
30	1	6	NA	NA	NA	NA	NA	NA
30	2	6	NA	NA	NA	NA	NA	NA
30	3	6	NA	NA	NA	NA	NA	NA
30	4	6	NA	NA	NA	NA	NA	NA
40	1	6	NA	NA	NA	NA	NA	NA
40	2	6	NA	NA	NA	NA	NA	NA
40	3	6	NA	NA	NA	NA	NA	NA
40	4	6	NA	NA	NA	NA	NA	NA
60	1	6	NA	NA	NA	NA	NA	NA
10	1	5	NA	NA	NA	NA	NA	NA
10	2	5	NA	NA	NA	NA	NA	NA
10	3	5	NA	NA	NA	NA	NA	NA
10	4	5	NA	NA	NA	NA	NA	NA
20	1	5	NA	NA	NA	NA	NA	NA
20	2	5	NA	NA	NA	NA	NA	NA
20	3	5	NA	NA	NA	NA	NA	NA
20	4	5	NA	NA	NA	NA	NA	NA
30	1	5	NA	NA	NA	NA	NA	NA
30	2	5	NA	NA	NA	NA	NA	NA
30	3	5	NA	NA	NA	NA	NA	NA
30	4	5	NA	NA	NA	NA	NA	NA
40	1	5	NA	NA	NA	NA	NA	NA
40	2	5	NA	NA	NA	NA	NA	NA
40	3	5	NA	NA	NA	NA	NA	NA
40	4	5	NA	NA	NA	NA	NA	NA
40	5	5	NA	NA	NA	NA	NA	NA
60	1	5	NA	NA	NA	NA	NA	NA
10	1	6	1	NA	NA	NA	NA	NA
10	2	6	1	NA	NA	NA	NA	NA
10	3	6	1	NA	NA	NA	NA	NA
10	4	6	1	NA	NA	NA	NA	NA
20	1	6	1	NA	NA	NA	NA	NA
20	2	6	1	NA	NA	NA	NA	NA

20	3	6	1	NA	NA	NA	NA	NA
20	4	6	1	NA	NA	NA	NA	NA
30	1	6	1	NA	NA	NA	NA	NA
30	2	6	1	NA	NA	NA	NA	NA
30	3	6	1	NA	NA	NA	NA	NA
30	4	6	1	NA	NA	NA	NA	NA
40	1	6	1	NA	NA	NA	NA	NA
40	2	6	1	NA	NA	NA	NA	NA
40	3	6	1	NA	NA	NA	NA	NA
40	4	6	1	NA	NA	NA	NA	NA
60	1	6	1	NA	NA	NA	NA	NA
10	1	5	NA	198.3	0.0	0.0	112.3	112.3
10	2	5	NA	146.3	0.0	156.1	140.3	296.4
10	3	5	NA	190.8	0.0	135.1	122.7	257.8
10	4	5	NA	144.0	166.0	143.8	123.1	432.9
20	1	5	NA	123.8	124.2	88.0	81.1	293.3
20	2	5	NA	103.5	109.6	109.8	68.4	287.8
20	3	5	NA	166.4	51.7	147.4	99.5	298.6
20	4	5	NA	185.1	31.7	77.4	83.9	193.0
30	1	5	NA	61.7	80.4	115.9	90.9	287.2
30	2	5	NA	90.2	94.0	90.6	113.1	297.7
30	3	5	NA	226.3	53.6	45.0	97.3	195.9
30	4	5	NA	119.4	67.2	108.4	89.9	265.5
40	1	5	NA	133.8	57.8	80.0	97.2	235.0
40	2	5	NA	162.1	15.5	57.1	121.2	193.8
40	3	5	NA	162.5	55.1	105.8	109.6	270.5
40	4	5	NA	187.8	38.7	37.9	75.4	152.0
40	5	5	NA	142.7	62.9	49.2	90.7	202.8
60	1	5	NA	146.0	60.1	54.8	91.1	206.0
10	1	5	NA	NA	NA	NA	NA	NA
10	2	5	NA	NA	NA	NA	NA	NA
10	3	5	NA	NA	NA	NA	NA	NA
10	4	5	NA	NA	NA	NA	NA	NA
20	1	5	NA	NA	NA	NA	NA	NA
20	2	5	NA	NA	NA	NA	NA	NA
20	3	5	NA	NA	NA	NA	NA	NA
20	4	5	NA	NA	NA	NA	NA	NA

30	1	5	NA	NA	NA	NA	NA	NA
30	2	5	NA	NA	NA	NA	NA	NA
30	3	5	NA	NA	NA	NA	NA	NA
30	4	5	NA	NA	NA	NA	NA	NA
40	1	5	NA	NA	NA	NA	NA	NA
40	2	5	NA	NA	NA	NA	NA	NA
40	3	5	NA	NA	NA	NA	NA	NA
40	4	5	NA	NA	NA	NA	NA	NA
40	5	5	NA	NA	NA	NA	NA	NA
60	1	5	NA	NA	NA	NA	NA	NA

Dens: density; Replic: replicate; Stage [Nymph 5 (= 5) or Adult (= 6)]; Sex (1 = females, 2 = males); “NA“ indicates data not applicable or not available for the given combination.

(Part 22)

Dens	Replic	Stage	Sex	Pct Fed D1	Pct Fed D2	Pct Fed D3	Pct Fed 3 Days	Pct feeding on day 2 and fed 1 day before
10	1	5	NA	NA	NA	NA	NA	NA
10	2	5	NA	NA	NA	NA	NA	NA
10	3	5	NA	NA	NA	NA	NA	NA
10	4	5	NA	NA	NA	NA	NA	NA
20	1	5	NA	NA	NA	NA	NA	NA
20	2	5	NA	NA	NA	NA	NA	NA
20	3	5	NA	NA	NA	NA	NA	NA
20	4	5	NA	NA	NA	NA	NA	NA
30	1	5	NA	NA	NA	NA	NA	NA
30	2	5	NA	NA	NA	NA	NA	NA
30	3	5	NA	NA	NA	NA	NA	NA
30	4	5	NA	NA	NA	NA	NA	NA
40	1	5	NA	NA	NA	NA	NA	NA
40	2	5	NA	NA	NA	NA	NA	NA
40	3	5	NA	NA	NA	NA	NA	NA
40	4	5	NA	NA	NA	NA	NA	NA
40	5	5	NA	NA	NA	NA	NA	NA
60	1	5	NA	NA	NA	NA	NA	NA
10	1	6	NA	NA	NA	NA	NA	NA
10	2	6	NA	NA	NA	NA	NA	NA
10	3	6	NA	NA	NA	NA	NA	NA
10	4	6	NA	NA	NA	NA	NA	NA
20	1	6	NA	NA	NA	NA	NA	NA
20	2	6	NA	NA	NA	NA	NA	NA
20	3	6	NA	NA	NA	NA	NA	NA
20	4	6	NA	NA	NA	NA	NA	NA
30	1	6	NA	NA	NA	NA	NA	NA
30	2	6	NA	NA	NA	NA	NA	NA
30	3	6	NA	NA	NA	NA	NA	NA
30	4	6	NA	NA	NA	NA	NA	NA
40	1	6	NA	NA	NA	NA	NA	NA
40	2	6	NA	NA	NA	NA	NA	NA
40	3	6	NA	NA	NA	NA	NA	NA
40	4	6	NA	NA	NA	NA	NA	NA
60	1	6	NA	NA	NA	NA	NA	NA

10	1	6	1	NA	NA	NA	NA	NA
10	2	6	1	NA	NA	NA	NA	NA
10	3	6	1	NA	NA	NA	NA	NA
10	4	6	1	NA	NA	NA	NA	NA
20	1	6	1	NA	NA	NA	NA	NA
20	2	6	1	NA	NA	NA	NA	NA
20	3	6	1	NA	NA	NA	NA	NA
20	4	6	1	NA	NA	NA	NA	NA
30	1	6	1	NA	NA	NA	NA	NA
30	2	6	1	NA	NA	NA	NA	NA
30	3	6	1	NA	NA	NA	NA	NA
30	4	6	1	NA	NA	NA	NA	NA
40	1	6	1	NA	NA	NA	NA	NA
40	2	6	1	NA	NA	NA	NA	NA
40	3	6	1	NA	NA	NA	NA	NA
40	4	6	1	NA	NA	NA	NA	NA
60	1	6	1	NA	NA	NA	NA	NA
10	1	6	2	NA	NA	NA	NA	NA
10	2	6	2	NA	NA	NA	NA	NA
10	3	6	2	NA	NA	NA	NA	NA
10	4	6	2	NA	NA	NA	NA	NA
20	1	6	2	NA	NA	NA	NA	NA
20	2	6	2	NA	NA	NA	NA	NA
20	3	6	2	NA	NA	NA	NA	NA
20	4	6	2	NA	NA	NA	NA	NA
30	1	6	2	NA	NA	NA	NA	NA
30	2	6	2	NA	NA	NA	NA	NA
30	3	6	2	NA	NA	NA	NA	NA
30	4	6	2	NA	NA	NA	NA	NA
40	1	6	2	NA	NA	NA	NA	NA
40	2	6	2	NA	NA	NA	NA	NA
40	3	6	2	NA	NA	NA	NA	NA
40	4	6	2	NA	NA	NA	NA	NA
60	1	6	2	NA	NA	NA	NA	NA
10	1	6	NA	NA	NA	NA	NA	NA
10	2	6	NA	NA	NA	NA	NA	NA
10	3	6	NA	NA	NA	NA	NA	NA

10	4	6	NA	NA	NA	NA	NA	NA
20	1	6	NA	NA	NA	NA	NA	NA
20	2	6	NA	NA	NA	NA	NA	NA
20	3	6	NA	NA	NA	NA	NA	NA
20	4	6	NA	NA	NA	NA	NA	NA
30	1	6	NA	NA	NA	NA	NA	NA
30	2	6	NA	NA	NA	NA	NA	NA
30	3	6	NA	NA	NA	NA	NA	NA
30	4	6	NA	NA	NA	NA	NA	NA
40	1	6	NA	NA	NA	NA	NA	NA
40	2	6	NA	NA	NA	NA	NA	NA
40	3	6	NA	NA	NA	NA	NA	NA
40	4	6	NA	NA	NA	NA	NA	NA
60	1	6	NA	NA	NA	NA	NA	NA
10	1	5	NA	NA	NA	NA	NA	NA
10	1	5	NA	NA	NA	NA	NA	NA
10	2	5	NA	NA	NA	NA	NA	NA
10	3	5	NA	NA	NA	NA	NA	NA
10	4	5	NA	NA	NA	NA	NA	NA
20	1	5	NA	NA	NA	NA	NA	NA
20	2	5	NA	NA	NA	NA	NA	NA
20	3	5	NA	NA	NA	NA	NA	NA
20	4	5	NA	NA	NA	NA	NA	NA
30	1	5	NA	NA	NA	NA	NA	NA
30	2	5	NA	NA	NA	NA	NA	NA
30	3	5	NA	NA	NA	NA	NA	NA
30	4	5	NA	NA	NA	NA	NA	NA
40	1	5	NA	NA	NA	NA	NA	NA
40	2	5	NA	NA	NA	NA	NA	NA
40	3	5	NA	NA	NA	NA	NA	NA
40	4	5	NA	NA	NA	NA	NA	NA
60	1	5	NA	NA	NA	NA	NA	NA
10	1	6	NA	NA	NA	NA	NA	NA
10	2	6	NA	NA	NA	NA	NA	NA
10	3	6	NA	NA	NA	NA	NA	NA
10	4	6	NA	NA	NA	NA	NA	NA
20	1	6	NA	NA	NA	NA	NA	NA

20	2	6	NA	NA	NA	NA	NA	NA
20	3	6	NA	NA	NA	NA	NA	NA
20	4	6	NA	NA	NA	NA	NA	NA
30	1	6	NA	NA	NA	NA	NA	NA
30	2	6	NA	NA	NA	NA	NA	NA
30	3	6	NA	NA	NA	NA	NA	NA
30	4	6	NA	NA	NA	NA	NA	NA
40	1	6	NA	NA	NA	NA	NA	NA
40	2	6	NA	NA	NA	NA	NA	NA
40	3	6	NA	NA	NA	NA	NA	NA
40	4	6	NA	NA	NA	NA	NA	NA
60	1	6	NA	NA	NA	NA	NA	NA
10	1	5	NA	NA	NA	NA	NA	NA
10	2	5	NA	NA	NA	NA	NA	NA
10	3	5	NA	NA	NA	NA	NA	NA
10	4	5	NA	NA	NA	NA	NA	NA
20	1	5	NA	NA	NA	NA	NA	NA
20	2	5	NA	NA	NA	NA	NA	NA
20	3	5	NA	NA	NA	NA	NA	NA
20	4	5	NA	NA	NA	NA	NA	NA
30	1	5	NA	NA	NA	NA	NA	NA
30	2	5	NA	NA	NA	NA	NA	NA
30	3	5	NA	NA	NA	NA	NA	NA
30	4	5	NA	NA	NA	NA	NA	NA
40	1	5	NA	NA	NA	NA	NA	NA
40	2	5	NA	NA	NA	NA	NA	NA
40	3	5	NA	NA	NA	NA	NA	NA
40	4	5	NA	NA	NA	NA	NA	NA
40	5	5	NA	NA	NA	NA	NA	NA
60	1	5	NA	NA	NA	NA	NA	NA
10	1	6	1	NA	NA	NA	NA	NA
10	2	6	1	NA	NA	NA	NA	NA
10	3	6	1	NA	NA	NA	NA	NA
10	4	6	1	NA	NA	NA	NA	NA
20	1	6	1	NA	NA	NA	NA	NA
20	2	6	1	NA	NA	NA	NA	NA
20	3	6	1	NA	NA	NA	NA	NA

20	4	6	1	NA	NA	NA	NA	NA
30	1	6	1	NA	NA	NA	NA	NA
30	2	6	1	NA	NA	NA	NA	NA
30	3	6	1	NA	NA	NA	NA	NA
30	4	6	1	NA	NA	NA	NA	NA
40	1	6	1	NA	NA	NA	NA	NA
40	2	6	1	NA	NA	NA	NA	NA
40	3	6	1	NA	NA	NA	NA	NA
40	4	6	1	NA	NA	NA	NA	NA
60	1	6	1	NA	NA	NA	NA	NA
10	1	5	NA	70	10	10	90	12.5
10	2	5	NA	80	60	10	150	40
10	3	5	NA	80	30	0	110	30
10	4	5	NA	90	20	0	110	25
20	1	5	NA	35	20	0	55	8.2
20	2	5	NA	55	5	0	60	10
20	3	5	NA	65	10	0	75	20
20	4	5	NA	55	10	5	70	7.7
30	1	5	NA	60	3.33	3.33	66.67	0
30	2	5	NA	46.67	13.33	0	60	7.7
30	3	5	NA	60	20	6.67	86.67	26.3
30	4	5	NA	46.67	16.67	3.33	66.67	26.6
40	1	5	NA	62.5	25	5	92.5	26.1
40	2	5	NA	67.5	22.5	15	105	26.9
40	3	5	NA	72.5	17.5	5	95	17.2
40	4	5	NA	60	22.5	20	102.5	24
40	5	5	NA	55	25	17.5	97.5	17.6
60	1	5	NA	65	31.67	20	116.67	23.2
10	1	5	NA	NA	NA	NA	NA	NA
10	2	5	NA	NA	NA	NA	NA	NA
10	3	5	NA	NA	NA	NA	NA	NA
10	4	5	NA	NA	NA	NA	NA	NA
20	1	5	NA	NA	NA	NA	NA	NA
20	2	5	NA	NA	NA	NA	NA	NA
20	3	5	NA	NA	NA	NA	NA	NA
20	4	5	NA	NA	NA	NA	NA	NA
30	1	5	NA	NA	NA	NA	NA	NA

30	2	5	NA	NA	NA	NA	NA	NA
30	3	5	NA	NA	NA	NA	NA	NA
30	4	5	NA	NA	NA	NA	NA	NA
40	1	5	NA	NA	NA	NA	NA	NA
40	2	5	NA	NA	NA	NA	NA	NA
40	3	5	NA	NA	NA	NA	NA	NA
40	4	5	NA	NA	NA	NA	NA	NA
40	5	5	NA	NA	NA	NA	NA	NA
60	1	5	NA	NA	NA	NA	NA	NA

Dens: density; Replic: replicate; Stage [Nymph 5 (= 5) or Adult (= 6)]; Sex (1 = females, 2 = males); "NA" indicates data not applicable or not available for the given combination.

(Part 23)

Dens	Replic	Stage	Sex	Pct feeding on day 3 and fed 1 day before	Pct feeding on day 3 and fed 2 days before	Pct feeding on day 3 and fed 1 and 2 days before	Molting Time N	Molting Time mean
10	1	5	NA	NA	NA	NA	NA	NA
10	2	5	NA	NA	NA	NA	NA	NA
10	3	5	NA	NA	NA	NA	NA	NA
10	4	5	NA	NA	NA	NA	NA	NA
20	1	5	NA	NA	NA	NA	NA	NA
20	2	5	NA	NA	NA	NA	NA	NA
20	3	5	NA	NA	NA	NA	NA	NA
20	4	5	NA	NA	NA	NA	NA	NA
30	1	5	NA	NA	NA	NA	NA	NA
30	2	5	NA	NA	NA	NA	NA	NA
30	3	5	NA	NA	NA	NA	NA	NA
30	4	5	NA	NA	NA	NA	NA	NA
40	1	5	NA	NA	NA	NA	NA	NA
40	2	5	NA	NA	NA	NA	NA	NA
40	3	5	NA	NA	NA	NA	NA	NA
40	4	5	NA	NA	NA	NA	NA	NA
40	5	5	NA	NA	NA	NA	NA	NA
60	1	5	NA	NA	NA	NA	NA	NA
10	1	6	NA	NA	NA	NA	NA	NA
10	2	6	NA	NA	NA	NA	NA	NA
10	3	6	NA	NA	NA	NA	NA	NA
10	4	6	NA	NA	NA	NA	NA	NA
20	1	6	NA	NA	NA	NA	NA	NA
20	2	6	NA	NA	NA	NA	NA	NA
20	3	6	NA	NA	NA	NA	NA	NA
20	4	6	NA	NA	NA	NA	NA	NA
30	1	6	NA	NA	NA	NA	NA	NA
30	2	6	NA	NA	NA	NA	NA	NA
30	3	6	NA	NA	NA	NA	NA	NA
30	4	6	NA	NA	NA	NA	NA	NA
40	1	6	NA	NA	NA	NA	NA	NA

40	2	6	NA	NA	NA	NA	NA	NA
40	3	6	NA	NA	NA	NA	NA	NA
40	4	6	NA	NA	NA	NA	NA	NA
60	1	6	NA	NA	NA	NA	NA	NA
10	1	6	1	NA	NA	NA	NA	NA
10	2	6	1	NA	NA	NA	NA	NA
10	3	6	1	NA	NA	NA	NA	NA
10	4	6	1	NA	NA	NA	NA	NA
20	1	6	1	NA	NA	NA	NA	NA
20	2	6	1	NA	NA	NA	NA	NA
20	3	6	1	NA	NA	NA	NA	NA
20	4	6	1	NA	NA	NA	NA	NA
30	1	6	1	NA	NA	NA	NA	NA
30	2	6	1	NA	NA	NA	NA	NA
30	3	6	1	NA	NA	NA	NA	NA
30	4	6	1	NA	NA	NA	NA	NA
40	1	6	1	NA	NA	NA	NA	NA
40	2	6	1	NA	NA	NA	NA	NA
40	3	6	1	NA	NA	NA	NA	NA
40	4	6	1	NA	NA	NA	NA	NA
60	1	6	1	NA	NA	NA	NA	NA
10	1	6	2	NA	NA	NA	NA	NA
10	2	6	2	NA	NA	NA	NA	NA
10	3	6	2	NA	NA	NA	NA	NA
10	4	6	2	NA	NA	NA	NA	NA
20	1	6	2	NA	NA	NA	NA	NA
20	2	6	2	NA	NA	NA	NA	NA
20	3	6	2	NA	NA	NA	NA	NA
20	4	6	2	NA	NA	NA	NA	NA
30	1	6	2	NA	NA	NA	NA	NA
30	2	6	2	NA	NA	NA	NA	NA
30	3	6	2	NA	NA	NA	NA	NA
30	4	6	2	NA	NA	NA	NA	NA
40	1	6	2	NA	NA	NA	NA	NA
40	2	6	2	NA	NA	NA	NA	NA
40	3	6	2	NA	NA	NA	NA	NA
40	4	6	2	NA	NA	NA	NA	NA

60	1	6	2	NA	NA	NA	NA	NA
10	1	6	NA	NA	NA	NA	NA	NA
10	2	6	NA	NA	NA	NA	NA	NA
10	3	6	NA	NA	NA	NA	NA	NA
10	4	6	NA	NA	NA	NA	NA	NA
20	1	6	NA	NA	NA	NA	NA	NA
20	2	6	NA	NA	NA	NA	NA	NA
20	3	6	NA	NA	NA	NA	NA	NA
20	4	6	NA	NA	NA	NA	NA	NA
30	1	6	NA	NA	NA	NA	NA	NA
30	2	6	NA	NA	NA	NA	NA	NA
30	3	6	NA	NA	NA	NA	NA	NA
30	4	6	NA	NA	NA	NA	NA	NA
40	1	6	NA	NA	NA	NA	NA	NA
40	2	6	NA	NA	NA	NA	NA	NA
40	3	6	NA	NA	NA	NA	NA	NA
40	4	6	NA	NA	NA	NA	NA	NA
60	1	6	NA	NA	NA	NA	NA	NA
10	1	5	NA	NA	NA	NA	NA	NA
10	1	5	NA	NA	NA	NA	NA	NA
10	2	5	NA	NA	NA	NA	NA	NA
10	3	5	NA	NA	NA	NA	NA	NA
10	4	5	NA	NA	NA	NA	NA	NA
20	1	5	NA	NA	NA	NA	NA	NA
20	2	5	NA	NA	NA	NA	NA	NA
20	3	5	NA	NA	NA	NA	NA	NA
20	4	5	NA	NA	NA	NA	NA	NA
30	1	5	NA	NA	NA	NA	NA	NA
30	2	5	NA	NA	NA	NA	NA	NA
30	3	5	NA	NA	NA	NA	NA	NA
30	4	5	NA	NA	NA	NA	NA	NA
40	1	5	NA	NA	NA	NA	NA	NA
40	2	5	NA	NA	NA	NA	NA	NA
40	3	5	NA	NA	NA	NA	NA	NA
40	4	5	NA	NA	NA	NA	NA	NA
60	1	5	NA	NA	NA	NA	NA	NA
10	1	6	NA	NA	NA	NA	NA	NA

10	2	6	NA	NA	NA	NA	NA	NA
10	3	6	NA	NA	NA	NA	NA	NA
10	4	6	NA	NA	NA	NA	NA	NA
20	1	6	NA	NA	NA	NA	NA	NA
20	2	6	NA	NA	NA	NA	NA	NA
20	3	6	NA	NA	NA	NA	NA	NA
20	4	6	NA	NA	NA	NA	NA	NA
30	1	6	NA	NA	NA	NA	NA	NA
30	2	6	NA	NA	NA	NA	NA	NA
30	3	6	NA	NA	NA	NA	NA	NA
30	4	6	NA	NA	NA	NA	NA	NA
40	1	6	NA	NA	NA	NA	NA	NA
40	2	6	NA	NA	NA	NA	NA	NA
40	3	6	NA	NA	NA	NA	NA	NA
40	4	6	NA	NA	NA	NA	NA	NA
60	1	6	NA	NA	NA	NA	NA	NA
10	1	5	NA	NA	NA	NA	NA	NA
10	2	5	NA	NA	NA	NA	NA	NA
10	3	5	NA	NA	NA	NA	NA	NA
10	4	5	NA	NA	NA	NA	NA	NA
20	1	5	NA	NA	NA	NA	NA	NA
20	2	5	NA	NA	NA	NA	NA	NA
20	3	5	NA	NA	NA	NA	NA	NA
20	4	5	NA	NA	NA	NA	NA	NA
30	1	5	NA	NA	NA	NA	NA	NA
30	2	5	NA	NA	NA	NA	NA	NA
30	3	5	NA	NA	NA	NA	NA	NA
30	4	5	NA	NA	NA	NA	NA	NA
40	1	5	NA	NA	NA	NA	NA	NA
40	2	5	NA	NA	NA	NA	NA	NA
40	3	5	NA	NA	NA	NA	NA	NA
40	4	5	NA	NA	NA	NA	NA	NA
40	5	5	NA	NA	NA	NA	NA	NA
60	1	5	NA	NA	NA	NA	NA	NA
10	1	6	1	NA	NA	NA	NA	NA
10	2	6	1	NA	NA	NA	NA	NA
10	3	6	1	NA	NA	NA	NA	NA

10	4	6	1	NA	NA	NA	NA	NA
20	1	6	1	NA	NA	NA	NA	NA
20	2	6	1	NA	NA	NA	NA	NA
20	3	6	1	NA	NA	NA	NA	NA
20	4	6	1	NA	NA	NA	NA	NA
30	1	6	1	NA	NA	NA	NA	NA
30	2	6	1	NA	NA	NA	NA	NA
30	3	6	1	NA	NA	NA	NA	NA
30	4	6	1	NA	NA	NA	NA	NA
40	1	6	1	NA	NA	NA	NA	NA
40	2	6	1	NA	NA	NA	NA	NA
40	3	6	1	NA	NA	NA	NA	NA
40	4	6	1	NA	NA	NA	NA	NA
60	1	6	1	NA	NA	NA	NA	NA
10	1	5	NA	0	12.5	0	NA	NA
10	2	5	NA	10	0	0	NA	NA
10	3	5	NA	0	0	0	NA	NA
10	4	5	NA	0	0	0	NA	NA
20	1	5	NA	0	0	0	NA	NA
20	2	5	NA	0	0	0	NA	NA
20	3	5	NA	0	0	0	NA	NA
20	4	5	NA	0	11.1	0	NA	NA
30	1	5	NA	0	8.3	0	NA	NA
30	2	5	NA	0	0	0	NA	NA
30	3	5	NA	0	0	14.3	NA	NA
30	4	5	NA	0	9.1	0	NA	NA
40	1	5	NA	0	0	6.3	NA	NA
40	2	5	NA	0	19.1	4.8	NA	NA
40	3	5	NA	0	5.6	5.6	NA	NA
40	4	5	NA	4.6	13.7	9.1	NA	NA
40	5	5	NA	9.4	6.3	6.3	NA	NA
60	1	5	NA	1.9	13.5	1.9	NA	NA
10	1	5	NA	NA	NA	NA	7	18.86
10	2	5	NA	NA	NA	NA	9	20.22
10	3	5	NA	NA	NA	NA	6	20.33
10	4	5	NA	NA	NA	NA	6	22.17
20	1	5	NA	NA	NA	NA	6	20.17

20	2	5	NA	NA	NA	NA	6	20.83
20	3	5	NA	NA	NA	NA	4	22.75
20	4	5	NA	NA	NA	NA	8	21.75
30	1	5	NA	NA	NA	NA	9	20.00
30	2	5	NA	NA	NA	NA	4	20.00
30	3	5	NA	NA	NA	NA	6	22.67
30	4	5	NA	NA	NA	NA	8	21.13
40	1	5	NA	NA	NA	NA	13	19.69
40	2	5	NA	NA	NA	NA	15	19.13
40	3	5	NA	NA	NA	NA	14	18.93
40	4	5	NA	NA	NA	NA	14	19.36
40	5	5	NA	NA	NA	NA	23	21.22
60	1	5	NA	NA	NA	NA	32	21.34

Dens: density; Replic: replicate; Stage [Nymph 5 (= 5) or Adult (= 6)]; Sex (1 = females, 2 = males); “NA“ indicates data not applicable or not available for the given combination.

(Part 24)

Dens	Replic	Stage	Sex	Molting Time_sd	N molted	N dying in_molt	N not molted	Pct molted
10	1	5	NA	NA	6	1	1	60
10	2	5	NA	NA	6	1	3	60
10	3	5	NA	NA	6	4	0	60
10	4	5	NA	NA	5	2	1	50
20	1	5	NA	NA	2	2	4	10
20	2	5	NA	NA	2	2	4	10
20	3	5	NA	NA	3	1	1	15
20	4	5	NA	NA	4	1	4	20
30	1	5	NA	NA	4	3	5	13.3
30	2	5	NA	NA	1	1	5	3.3
30	3	5	NA	NA	5	7	2	16.7
30	4	5	NA	NA	4	2	5	13.3
40	1	5	NA	NA	6	3	7	15
40	2	5	NA	NA	9	6	6	22.5
40	3	5	NA	NA	6	4	8	15
40	4	5	NA	NA	6	7	9	15
40	5	5	NA	NA	10	10	12	25
60	1	5	NA	NA	15	20	17	25
10	1	6	NA	NA	NA	NA	NA	NA
10	2	6	NA	NA	NA	NA	NA	NA
10	3	6	NA	NA	NA	NA	NA	NA
10	4	6	NA	NA	NA	NA	NA	NA
20	1	6	NA	NA	NA	NA	NA	NA
20	2	6	NA	NA	NA	NA	NA	NA
20	3	6	NA	NA	NA	NA	NA	NA
20	4	6	NA	NA	NA	NA	NA	NA
30	1	6	NA	NA	NA	NA	NA	NA
30	2	6	NA	NA	NA	NA	NA	NA
30	3	6	NA	NA	NA	NA	NA	NA
30	4	6	NA	NA	NA	NA	NA	NA
40	1	6	NA	NA	NA	NA	NA	NA
40	2	6	NA	NA	NA	NA	NA	NA
40	3	6	NA	NA	NA	NA	NA	NA
40	4	6	NA	NA	NA	NA	NA	NA
60	1	6	NA	NA	NA	NA	NA	NA

10	1	6	1	NA	NA	NA	NA	NA
10	2	6	1	NA	NA	NA	NA	NA
10	3	6	1	NA	NA	NA	NA	NA
10	4	6	1	NA	NA	NA	NA	NA
20	1	6	1	NA	NA	NA	NA	NA
20	2	6	1	NA	NA	NA	NA	NA
20	3	6	1	NA	NA	NA	NA	NA
20	4	6	1	NA	NA	NA	NA	NA
30	1	6	1	NA	NA	NA	NA	NA
30	2	6	1	NA	NA	NA	NA	NA
30	3	6	1	NA	NA	NA	NA	NA
30	4	6	1	NA	NA	NA	NA	NA
40	1	6	1	NA	NA	NA	NA	NA
40	2	6	1	NA	NA	NA	NA	NA
40	3	6	1	NA	NA	NA	NA	NA
40	4	6	1	NA	NA	NA	NA	NA
60	1	6	1	NA	NA	NA	NA	NA
10	1	6	2	NA	NA	NA	NA	NA
10	2	6	2	NA	NA	NA	NA	NA
10	3	6	2	NA	NA	NA	NA	NA
10	4	6	2	NA	NA	NA	NA	NA
20	1	6	2	NA	NA	NA	NA	NA
20	2	6	2	NA	NA	NA	NA	NA
20	3	6	2	NA	NA	NA	NA	NA
20	4	6	2	NA	NA	NA	NA	NA
30	1	6	2	NA	NA	NA	NA	NA
30	2	6	2	NA	NA	NA	NA	NA
30	3	6	2	NA	NA	NA	NA	NA
30	4	6	2	NA	NA	NA	NA	NA
40	1	6	2	NA	NA	NA	NA	NA
40	2	6	2	NA	NA	NA	NA	NA
40	3	6	2	NA	NA	NA	NA	NA
40	4	6	2	NA	NA	NA	NA	NA
60	1	6	2	NA	NA	NA	NA	NA
10	1	6	NA	NA	NA	NA	NA	NA
10	2	6	NA	NA	NA	NA	NA	NA
10	3	6	NA	NA	NA	NA	NA	NA

10	4	6	NA	NA	NA	NA	NA	NA
20	1	6	NA	NA	NA	NA	NA	NA
20	2	6	NA	NA	NA	NA	NA	NA
20	3	6	NA	NA	NA	NA	NA	NA
20	4	6	NA	NA	NA	NA	NA	NA
30	1	6	NA	NA	NA	NA	NA	NA
30	2	6	NA	NA	NA	NA	NA	NA
30	3	6	NA	NA	NA	NA	NA	NA
30	4	6	NA	NA	NA	NA	NA	NA
40	1	6	NA	NA	NA	NA	NA	NA
40	2	6	NA	NA	NA	NA	NA	NA
40	3	6	NA	NA	NA	NA	NA	NA
40	4	6	NA	NA	NA	NA	NA	NA
60	1	6	NA	NA	NA	NA	NA	NA
10	1	5	NA	NA	NA	NA	NA	NA
10	1	5	NA	NA	NA	NA	NA	NA
10	2	5	NA	NA	NA	NA	NA	NA
10	3	5	NA	NA	NA	NA	NA	NA
10	4	5	NA	NA	NA	NA	NA	NA
20	1	5	NA	NA	NA	NA	NA	NA
20	2	5	NA	NA	NA	NA	NA	NA
20	3	5	NA	NA	NA	NA	NA	NA
20	4	5	NA	NA	NA	NA	NA	NA
30	1	5	NA	NA	NA	NA	NA	NA
30	2	5	NA	NA	NA	NA	NA	NA
30	3	5	NA	NA	NA	NA	NA	NA
30	4	5	NA	NA	NA	NA	NA	NA
40	1	5	NA	NA	NA	NA	NA	NA
40	2	5	NA	NA	NA	NA	NA	NA
40	3	5	NA	NA	NA	NA	NA	NA
40	4	5	NA	NA	NA	NA	NA	NA
60	1	5	NA	NA	NA	NA	NA	NA
10	1	6	NA	NA	NA	NA	NA	NA
10	2	6	NA	NA	NA	NA	NA	NA
10	3	6	NA	NA	NA	NA	NA	NA
10	4	6	NA	NA	NA	NA	NA	NA
20	1	6	NA	NA	NA	NA	NA	NA

20	2	6	NA	NA	NA	NA	NA	NA
20	3	6	NA	NA	NA	NA	NA	NA
20	4	6	NA	NA	NA	NA	NA	NA
30	1	6	NA	NA	NA	NA	NA	NA
30	2	6	NA	NA	NA	NA	NA	NA
30	3	6	NA	NA	NA	NA	NA	NA
30	4	6	NA	NA	NA	NA	NA	NA
40	1	6	NA	NA	NA	NA	NA	NA
40	2	6	NA	NA	NA	NA	NA	NA
40	3	6	NA	NA	NA	NA	NA	NA
40	4	6	NA	NA	NA	NA	NA	NA
60	1	6	NA	NA	NA	NA	NA	NA
10	1	5	NA	NA	NA	NA	NA	NA
10	2	5	NA	NA	NA	NA	NA	NA
10	3	5	NA	NA	NA	NA	NA	NA
10	4	5	NA	NA	NA	NA	NA	NA
20	1	5	NA	NA	NA	NA	NA	NA
20	2	5	NA	NA	NA	NA	NA	NA
20	3	5	NA	NA	NA	NA	NA	NA
20	4	5	NA	NA	NA	NA	NA	NA
30	1	5	NA	NA	NA	NA	NA	NA
30	2	5	NA	NA	NA	NA	NA	NA
30	3	5	NA	NA	NA	NA	NA	NA
30	4	5	NA	NA	NA	NA	NA	NA
40	1	5	NA	NA	NA	NA	NA	NA
40	2	5	NA	NA	NA	NA	NA	NA
40	3	5	NA	NA	NA	NA	NA	NA
40	4	5	NA	NA	NA	NA	NA	NA
40	5	5	NA	NA	NA	NA	NA	NA
60	1	5	NA	NA	NA	NA	NA	NA
10	1	6	1	NA	NA	NA	NA	NA
10	2	6	1	NA	NA	NA	NA	NA
10	3	6	1	NA	NA	NA	NA	NA
10	4	6	1	NA	NA	NA	NA	NA
20	1	6	1	NA	NA	NA	NA	NA
20	2	6	1	NA	NA	NA	NA	NA
20	3	6	1	NA	NA	NA	NA	NA

20	4	6	1	NA	NA	NA	NA	NA
30	1	6	1	NA	NA	NA	NA	NA
30	2	6	1	NA	NA	NA	NA	NA
30	3	6	1	NA	NA	NA	NA	NA
30	4	6	1	NA	NA	NA	NA	NA
40	1	6	1	NA	NA	NA	NA	NA
40	2	6	1	NA	NA	NA	NA	NA
40	3	6	1	NA	NA	NA	NA	NA
40	4	6	1	NA	NA	NA	NA	NA
60	1	6	1	NA	NA	NA	NA	NA
10	1	5	NA	NA	NA	NA	NA	NA
10	2	5	NA	NA	NA	NA	NA	NA
10	3	5	NA	NA	NA	NA	NA	NA
10	4	5	NA	NA	NA	NA	NA	NA
20	1	5	NA	NA	NA	NA	NA	NA
20	2	5	NA	NA	NA	NA	NA	NA
20	3	5	NA	NA	NA	NA	NA	NA
20	4	5	NA	NA	NA	NA	NA	NA
30	1	5	NA	NA	NA	NA	NA	NA
30	2	5	NA	NA	NA	NA	NA	NA
30	3	5	NA	NA	NA	NA	NA	NA
30	4	5	NA	NA	NA	NA	NA	NA
40	1	5	NA	NA	NA	NA	NA	NA
40	2	5	NA	NA	NA	NA	NA	NA
40	3	5	NA	NA	NA	NA	NA	NA
40	4	5	NA	NA	NA	NA	NA	NA
40	5	5	NA	NA	NA	NA	NA	NA
60	1	5	NA	NA	NA	NA	NA	NA
10	1	5	NA	1.46	NA	NA	NA	NA
10	2	5	NA	2.05	NA	NA	NA	NA
10	3	5	NA	2.07	NA	NA	NA	NA
10	4	5	NA	2.23	NA	NA	NA	NA
20	1	5	NA	1.60	NA	NA	NA	NA
20	2	5	NA	1.33	NA	NA	NA	NA
20	3	5	NA	4.27	NA	NA	NA	NA
20	4	5	NA	3.45	NA	NA	NA	NA
30	1	5	NA	1.73	NA	NA	NA	NA

30	2	5	NA	0.00	NA	NA	NA	NA
30	3	5	NA	4.27	NA	NA	NA	NA
30	4	5	NA	1.46	NA	NA	NA	NA
40	1	5	NA	1.03	NA	NA	NA	NA
40	2	5	NA	1.06	NA	NA	NA	NA
40	3	5	NA	1.33	NA	NA	NA	NA
40	4	5	NA	1.01	NA	NA	NA	NA
40	5	5	NA	1.48	NA	NA	NA	NA
60	1	5	NA	1.56	NA	NA	NA	NA

Dens: density; Replic: replicate; Stage [Nymph 5 (= 5) or Adult (= 6)]; Sex (1 = females, 2 = males); "NA" indicates data not applicable or not available for the given combination.

(Part 25)

Dens	Replic	Stage	Sex	Pct dying in molt	Pct not molted	Mean time feed to molt days	Sd time feed to molt days
10	1	5	NA	10	10	18.7	1.5
10	2	5	NA	10	30	20.3	2.6
10	3	5	NA	40	0	20.3	2.2
10	4	5	NA	20	10	22.6	2.2
20	1	5	NA	10	20	20.5	3.5
20	2	5	NA	10	20	22.5	0.7
20	3	5	NA	5	5	23.7	4.7
20	4	5	NA	5	20	23.5	4.4
30	1	5	NA	10	16.7	20	2.8
30	2	5	NA	3.3	16.7	20	NA
30	3	5	NA	23.3	6.7	23.2	4.5
30	4	5	NA	6.7	16.7	22.3	1.3
40	1	5	NA	7.5	17.5	19.3	1.5
40	2	5	NA	15	15	18.6	1
40	3	5	NA	10	20	17.5	0.5
40	4	5	NA	17.5	22.5	18.5	1
40	5	5	NA	25	30	22.6	4.8
60	1	5	NA	33.3	28.3	22.9	0.8
10	1	6	NA	NA	NA	NA	NA
10	2	6	NA	NA	NA	NA	NA
10	3	6	NA	NA	NA	NA	NA
10	4	6	NA	NA	NA	NA	NA
20	1	6	NA	NA	NA	NA	NA
20	2	6	NA	NA	NA	NA	NA
20	3	6	NA	NA	NA	NA	NA
20	4	6	NA	NA	NA	NA	NA
30	1	6	NA	NA	NA	NA	NA
30	2	6	NA	NA	NA	NA	NA
30	3	6	NA	NA	NA	NA	NA
30	4	6	NA	NA	NA	NA	NA
40	1	6	NA	NA	NA	NA	NA
40	2	6	NA	NA	NA	NA	NA
40	3	6	NA	NA	NA	NA	NA

40	4	6	NA	NA	NA	NA	NA
60	1	6	NA	NA	NA	NA	NA
10	1	6	1	NA	NA	NA	NA
10	2	6	1	NA	NA	NA	NA
10	3	6	1	NA	NA	NA	NA
10	4	6	1	NA	NA	NA	NA
20	1	6	1	NA	NA	NA	NA
20	2	6	1	NA	NA	NA	NA
20	3	6	1	NA	NA	NA	NA
20	4	6	1	NA	NA	NA	NA
30	1	6	1	NA	NA	NA	NA
30	2	6	1	NA	NA	NA	NA
30	3	6	1	NA	NA	NA	NA
30	4	6	1	NA	NA	NA	NA
40	1	6	1	NA	NA	NA	NA
40	2	6	1	NA	NA	NA	NA
40	3	6	1	NA	NA	NA	NA
40	4	6	1	NA	NA	NA	NA
60	1	6	1	NA	NA	NA	NA
10	1	6	2	NA	NA	NA	NA
10	2	6	2	NA	NA	NA	NA
10	3	6	2	NA	NA	NA	NA
10	4	6	2	NA	NA	NA	NA
20	1	6	2	NA	NA	NA	NA
20	2	6	2	NA	NA	NA	NA
20	3	6	2	NA	NA	NA	NA
20	4	6	2	NA	NA	NA	NA
30	1	6	2	NA	NA	NA	NA
30	2	6	2	NA	NA	NA	NA
30	3	6	2	NA	NA	NA	NA
30	4	6	2	NA	NA	NA	NA
40	1	6	2	NA	NA	NA	NA
40	2	6	2	NA	NA	NA	NA
40	3	6	2	NA	NA	NA	NA
40	4	6	2	NA	NA	NA	NA
60	1	6	2	NA	NA	NA	NA
10	1	6	NA	NA	NA	NA	NA

10	2	6	NA	NA	NA	NA	NA
10	3	6	NA	NA	NA	NA	NA
10	4	6	NA	NA	NA	NA	NA
20	1	6	NA	NA	NA	NA	NA
20	2	6	NA	NA	NA	NA	NA
20	3	6	NA	NA	NA	NA	NA
20	4	6	NA	NA	NA	NA	NA
30	1	6	NA	NA	NA	NA	NA
30	2	6	NA	NA	NA	NA	NA
30	3	6	NA	NA	NA	NA	NA
30	4	6	NA	NA	NA	NA	NA
40	1	6	NA	NA	NA	NA	NA
40	2	6	NA	NA	NA	NA	NA
40	3	6	NA	NA	NA	NA	NA
40	4	6	NA	NA	NA	NA	NA
60	1	6	NA	NA	NA	NA	NA
10	1	5	NA	NA	NA	NA	NA
10	1	5	NA	NA	NA	NA	NA
10	2	5	NA	NA	NA	NA	NA
10	3	5	NA	NA	NA	NA	NA
10	4	5	NA	NA	NA	NA	NA
20	1	5	NA	NA	NA	NA	NA
20	2	5	NA	NA	NA	NA	NA
20	3	5	NA	NA	NA	NA	NA
20	4	5	NA	NA	NA	NA	NA
30	1	5	NA	NA	NA	NA	NA
30	2	5	NA	NA	NA	NA	NA
30	3	5	NA	NA	NA	NA	NA
30	4	5	NA	NA	NA	NA	NA
40	1	5	NA	NA	NA	NA	NA
40	2	5	NA	NA	NA	NA	NA
40	3	5	NA	NA	NA	NA	NA
40	4	5	NA	NA	NA	NA	NA
60	1	5	NA	NA	NA	NA	NA
10	1	6	NA	NA	NA	NA	NA
10	2	6	NA	NA	NA	NA	NA
10	3	6	NA	NA	NA	NA	NA

10	4	6	NA	NA	NA	NA	NA
20	1	6	NA	NA	NA	NA	NA
20	2	6	NA	NA	NA	NA	NA
20	3	6	NA	NA	NA	NA	NA
20	4	6	NA	NA	NA	NA	NA
30	1	6	NA	NA	NA	NA	NA
30	2	6	NA	NA	NA	NA	NA
30	3	6	NA	NA	NA	NA	NA
30	4	6	NA	NA	NA	NA	NA
40	1	6	NA	NA	NA	NA	NA
40	2	6	NA	NA	NA	NA	NA
40	3	6	NA	NA	NA	NA	NA
40	4	6	NA	NA	NA	NA	NA
60	1	6	NA	NA	NA	NA	NA
10	1	5	NA	NA	NA	NA	NA
10	2	5	NA	NA	NA	NA	NA
10	3	5	NA	NA	NA	NA	NA
10	4	5	NA	NA	NA	NA	NA
20	1	5	NA	NA	NA	NA	NA
20	2	5	NA	NA	NA	NA	NA
20	3	5	NA	NA	NA	NA	NA
20	4	5	NA	NA	NA	NA	NA
30	1	5	NA	NA	NA	NA	NA
30	2	5	NA	NA	NA	NA	NA
30	3	5	NA	NA	NA	NA	NA
30	4	5	NA	NA	NA	NA	NA
40	1	5	NA	NA	NA	NA	NA
40	2	5	NA	NA	NA	NA	NA
40	3	5	NA	NA	NA	NA	NA
40	4	5	NA	NA	NA	NA	NA
40	5	5	NA	NA	NA	NA	NA
60	1	5	NA	NA	NA	NA	NA
10	1	6	1	NA	NA	NA	NA
10	2	6	1	NA	NA	NA	NA
10	3	6	1	NA	NA	NA	NA
10	4	6	1	NA	NA	NA	NA
20	1	6	1	NA	NA	NA	NA

20	2	6	1	NA	NA	NA	NA
20	3	6	1	NA	NA	NA	NA
20	4	6	1	NA	NA	NA	NA
30	1	6	1	NA	NA	NA	NA
30	2	6	1	NA	NA	NA	NA
30	3	6	1	NA	NA	NA	NA
30	4	6	1	NA	NA	NA	NA
40	1	6	1	NA	NA	NA	NA
40	2	6	1	NA	NA	NA	NA
40	3	6	1	NA	NA	NA	NA
40	4	6	1	NA	NA	NA	NA
60	1	6	1	NA	NA	NA	NA
10	1	5	NA	NA	NA	NA	NA
10	2	5	NA	NA	NA	NA	NA
10	3	5	NA	NA	NA	NA	NA
10	4	5	NA	NA	NA	NA	NA
20	1	5	NA	NA	NA	NA	NA
20	2	5	NA	NA	NA	NA	NA
20	3	5	NA	NA	NA	NA	NA
20	4	5	NA	NA	NA	NA	NA
30	1	5	NA	NA	NA	NA	NA
30	2	5	NA	NA	NA	NA	NA
30	3	5	NA	NA	NA	NA	NA
30	4	5	NA	NA	NA	NA	NA
40	1	5	NA	NA	NA	NA	NA
40	2	5	NA	NA	NA	NA	NA
40	3	5	NA	NA	NA	NA	NA
40	4	5	NA	NA	NA	NA	NA
40	5	5	NA	NA	NA	NA	NA
60	1	5	NA	NA	NA	NA	NA
10	1	5	NA	NA	NA	NA	NA
10	2	5	NA	NA	NA	NA	NA
10	3	5	NA	NA	NA	NA	NA
10	4	5	NA	NA	NA	NA	NA
20	1	5	NA	NA	NA	NA	NA
20	2	5	NA	NA	NA	NA	NA
20	3	5	NA	NA	NA	NA	NA

20	4	5	NA	NA	NA	NA	NA
30	1	5	NA	NA	NA	NA	NA
30	2	5	NA	NA	NA	NA	NA
30	3	5	NA	NA	NA	NA	NA
30	4	5	NA	NA	NA	NA	NA
40	1	5	NA	NA	NA	NA	NA
40	2	5	NA	NA	NA	NA	NA
40	3	5	NA	NA	NA	NA	NA
40	4	5	NA	NA	NA	NA	NA
40	5	5	NA	NA	NA	NA	NA
60	1	5	NA	NA	NA	NA	NA

Dens: density; Replic: replicate; Stage [Nymph 5 (= 5) or Adult (= 6)]; Sex (1 = females, 2 = males); “NA“ indicates data not applicable or not available for the given combination.

SUPPLEMENTARY DATA VI

```
##### R Computer code for the analysis of the paper #####
#### Population size regulation is density-dependent ####
##### in Rhodnius prolixus (Hemiptera: Reduviidae) #####
########## through an irritability mechanism ##########
###### by Enrique H. Weir and Jorge E. Rabinovich ######

# We read the Irritability results at the level of each day (of the 3 days of observations)

setwd("xxxxxxx") #### NOTE: The corresponding folder where the data file is located, should be inserted here
datir <- read.csv(file="Irritability as Database with Adults and Nymphs V.csv")
datir_ad <- subset(datir, datir$Stage == "A")
datir_ni <- subset(datir, datir$Stage == "V")
# We read all the data results compiled in one file
setwd("D:\\Vinchucas\\Luca\\Denso-Dep\\Efecto DD Weir\\MS Weir\\Manuscrito\\New calculations\\All_Data_One_File")
dat <- read.csv(file="All_Data_One_File2.csv")
names(dat)
# We select the variables of the data set into five categories:
# (1) Feeding, (2) Mortality, (3) Reproduction, (4) Molting/longevity, and (5) Dispersal
# For some questions of interest there are some "combined" or "mixed" variables
# combining three single variables: mortality, feeding and movement:
# (a) mort_in_bugs_that_moved
# (b) mort_in_bugs_that_fed
# (c) fed_bugs_that _moved
# Only feeding
position <- c(1:5, 47:53, 74:86, 91:103)
feeding <- dat[,position]
names(feeding)

# Only mortality
position <- c(1:5, 6:14)
mortality <- dat[,position]
names(mortality)

# Only reproduction
position <- c(1:5, 57:73)
reproduction <- dat[,position]
names(reproduction)

# Only molting_longevity
position <- c(1:5, 33:34, 54:56, 103:105)
molting_longevity <- dat[,position]
names(molting_longevity)

# Only dispersal
position <- c(1:5, 35:38)
dispersal <- dat[,position]
names(dispersal)

# (a) mort_moved (mort_in_bugs_that_moved)
position <- c(1:5, 15:23)
mort_moved <- dat[,position]
names(mort_moved)
```

CC BY

```
# (b) mort_fed (mort_in_bugs_that_fed)
position <- c(1:5, 24:32)
mort_fed <- dat[,position]
names(mort_fed)

# (c) mort_molt (mort_in_bugs_that_molted)
position <- c(1:5, 112,115)
mort_molt <- dat[,position]
names(mort_molt)

# (d) fed_moved (fed_bugs_that _moved)
position <- c(1:5, 39:46, 83:90)
fed_moved <- dat[,position]
names(fed_moved)



# In summary we have 9 new datasets:
##############################################################
# [Number of variables does not include Density, Replicate, Stage, Sex, and Irritability] # feeding [27
variables]
# mortality [9 variables]
# reproduction [17 variables]
# molting_longevity [8 variables]
# dispersal [4 variables]
# mort_moved (mort_in_bugs_that_moved) [9 variables]
# mort_fed  (mort_in_bugs_that_fed) [9 variables]
# mort_molt (mort_in_bugs_that_molted)  [2 variables]
# fed_moved (fed_bugs_that _moved) [16 variables]
###############################################################
#####
# Load libraries
library(ggplot2)
library(lattice)
library(latticeExtra)
library(ggeasy)
library(dplyr)
library(tidyverse)
library(tidyr)
library(cowplot)
library(psych)
library(reshape2)
library(ggpubr)
library(rstatix)
library(AICcmodavg)
library(betareg)
###############################################################
####
names(datir_ad)
qplot(x = Density, y = jitter(Irritability), facets = ~ Day, data = datir_ad) +
geom_smooth(method = "lm")
qplot(x = Density, y = jitter(Irritability), facets = ~ Day, data = datir_ni) +
geom_smooth(method = "lm")
##### Checking linear relationships  in Irritability ~ Density #####
datir_ni_linear <- datir_ni %>%
group_by(Density) %>%
```

```
summarise_at(vars(Irritability), funs(n(), mean, sd))
datir_ni_linear
reg_irr_ni <- lm(mean ~ Density, data= datir_ni_linear)
summary(reg_irr_ni)
##################################################
# We compare the linear regressions between days for each Stage
dat_ni_D1 <- subset(datir_ni, datir_ni$Day == 1)
dat_ni_D2 <- subset(datir_ni, datir_ni$Day == 2)
dat_ni_D3 <- subset(datir_ni, datir_ni$Day == 3)
reg_ni_D1 <- lm(Irritability ~ Density, dat=dat_ni_D1)
summary(reg_ni_D1)
reg_ni_D2 <- lm(Irritability ~ Density, dat=dat_ni_D2)
summary(reg_ni_D2)
reg_ni_D3 <- lm(Irritability ~ Density, dat=dat_ni_D3)
summary(reg_ni_D3)

#define list of models
models <- list(reg_ni_D1, reg_ni_D2, reg_ni_D3)
#specify model names
mod.names <- c('Linear_Day1', 'Linear_Day2',' Linear_Day3' )
#calculate AIC of each model
aictab(cand.set = models, modnames = mod.names)
dat_ad_D1 <- subset(datir_ad, datir_ad$Day == 1)
dat_ad_D2 <- subset(datir_ad, datir_ad$Day == 2)
dat_ad_D3 <- subset(datir_ad, datir_ad$Day == 3)
reg_ad_D1 <- lm(Irritability ~ Density, dat=dat_ad_D1)
summary(reg_ad_D1)
reg_ad_D2 <- lm(Irritability ~ Density, dat=dat_ad_D2)
summary(reg_ad_D2)
reg_ad_D3 <- lm(Irritability ~ Density, dat=dat_ad_D3)
summary(reg_ad_D3)

#define list of models
models <- list(reg_ad_D1, reg_ad_D2, reg_ad_D3)
#specify model names
mod.names <- c('Linear_Day1', 'Linear_Day2',' Linear_Day3' )
#calculate AIC of each model
aictab(cand.set = models, modnames = mod.names)
# We look at the interaction between Day and Density
reg_dens_day_ni <- lm(Irritability ~ Density* Day, data = datir_ni) summary(reg_dens_day_ni)
reg_dens_day_ad <- lm(Irritability ~ Density* Day, data = datir_ad) summary(reg_dens_day_ad)
##############################################
##### Analysis pooling stage 5 nymphs and adults #####
##############################################
datir_ni_ad <- rbind(datir_ni, datir_ad)
length(datir_ni_ad$Density)
length(datir_ni$Density)
length(datir_ad$Density)
reg_S5_ad_AllDays <- lm(Irritability ~ Density*Day, data = datir_ni_ad) summary(reg_S5_ad_AllDays)
############################################################
#######

########################################
##### Producing irritability means for all days #####
########################################
n5_irr_all_days <- datir_ni %>% group_by(Density, Replicate) %>%
summarise(Irr_mean_n5_all_days= mean(Irritability))
```

```
n5_irr_all_days
ad_irr_all_days <- datir_ad %>% group_by(Density, Replicate) %>%
summarise(Irr_mean_ad_all_days= mean(Irritability))
ad_irr_all_days

###########################################
##### Inserting the irritability analysis by day ####
###########################################
##### For stage 5 nymphs
n5_irr_by_day <- datir_ni %>% group_by(Density, Day) %>%
summarise(Irr_mean_n5= mean(Irritability))
n5_irr_by_day qplot(x = Density, y = Irr_mean_n5, facets = ~Day, data = n5_irr_by_day) +
geom_smooth(method = "lm")
Density_names <- as.factor(c("Density_10", "Density_10", "Density_10", "Density_20",
"Density_20", "Density_20", "Density_30", "Density_30", "Density_30", "Density_40",
"Density_40", "Density_40", "Density_60", "Density_60", "Density_60"))
n5_irr_by_day2 <- cbind(n5_irr_by_day,Density_names)
names(n5_irr_by_day2)[names(n5_irr_by_day2) == "...4"] <- "Density_level" n5_irr_by_day2
qplot(x = Day, y = Irr_mean_n5, facets = ~Density_level, data = n5_irr_by_day2) + labs(x = "Trial
day", y = "Irritability level of stage 5 nymphs") + geom_smooth(method = "lm")
reg1_n5 <- lm(Irr_mean_n5 ~ Day * Density, data= n5_irr_by_day2) summary(reg1_n5)

#################################################################
#### We select the datasets for each experimental density level ####
#################################################################
##### For stage 5 nymphs
# For Density_10
n5_irr_by_day_density_10 <- subset(n5_irr_by_day2, n5_irr_by_day2$Density_level ==
"Density_10")
n5_irr_by_day_density_10
reg1_n5_by_day_density_10 <- lm(Irr_mean_n5 ~ Day, data= n5_irr_by_day_density_10)
summary(reg1_n5_by_day_density_10)
# For Density_20
n5_irr_by_day_density_20 <- subset(n5_irr_by_day2, n5_irr_by_day2$Density_level ==
"Density_20")
n5_irr_by_day_density_20
reg1_n5_by_day_density_20 <- lm(Irr_mean_n5 ~ Day, data= n5_irr_by_day_density_20)
summary(reg1_n5_by_day_density_20)
# For Density_30
n5_irr_by_day_density_30 <- subset(n5_irr_by_day2, n5_irr_by_day2$Density_level ==
"Density_30")
n5_irr_by_day_density_30
reg1_n5_by_day_density_30 <- lm(Irr_mean_n5 ~ Day, data= n5_irr_by_day_density_30)
summary(reg1_n5_by_day_density_30)
# For Density_40
n5_irr_by_day_density_40 <- subset(n5_irr_by_day2, n5_irr_by_day2$Density_level ==
"Density_40")
n5_irr_by_day_density_40
reg1_n5_by_day_density_40 <- lm(Irr_mean_n5 ~ Day, data= n5_irr_by_day_density_40)
summary(reg1_n5_by_day_density_40)
# For Density_60
n5_irr_by_day_density_60 <- subset(n5_irr_by_day2, n5_irr_by_day2$Density_level ==
"Density_60")
n5_irr_by_day_density_60
reg1_n5_by_day_density_60 <- lm(Irr_mean_n5 ~ Day, data= n5_irr_by_day_density_60)
summary(reg1_n5_by_day_density_60)
```

```
##### For adults
ad_irr_by_day <- datir_ad %>% group_by(Density, Day) %>%
summarise(Irr_mean_ad= mean(Irritability))
ad_irr_by_day
ad_irr_by_day2 <- cbind(ad_irr_by_day,Density_names)
names(ad_irr_by_day2)[names(ad_irr_by_day2) == "...4"] <- "Density_level" ad_irr_by_day2
qplot(x = Day, y = Irr_mean_ad, facets = ~Density_level, data = ad_irr_by_day2) + labs(x = "Trial
day", y = "Irritability level of adults") + geom_smooth(method = "lm")
reg1_ad <- lm(Irr_mean_ad ~ Day * Density, data= ad_irr_by_day2) summary(reg1_ad)

##########################################################
#### We select the datasets for each experimental density level ####
##########################################################
# For Density_10
ad_irr_by_day_density_10 <- subset(ad_irr_by_day2, ad_irr_by_day2$Density_level ==
"Density_10")
ad_irr_by_day_density_10
reg1_ad_by_day_density_10 <- lm(Irr_mean_ad ~ Day, data= ad_irr_by_day_density_10)
summary(reg1_ad_by_day_density_10)
# For Density_20
ad_irr_by_day_density_20 <- subset(ad_irr_by_day2, ad_irr_by_day2$Density_level ==
"Density_20")
ad_irr_by_day_density_20
reg1_ad_by_day_density_20 <- lm(Irr_mean_ad ~ Day, data= ad_irr_by_day_density_20)
summary(reg1_ad_by_day_density_20)
# For Density_30
ad_irr_by_day_density_30 <- subset(ad_irr_by_day2, ad_irr_by_day2$Density_level ==
"Density_30")
ad_irr_by_day_density_30
reg1_ad_by_day_density_30 <- lm(Irr_mean_ad ~ Day, data= ad_irr_by_day_density_30)
summary(reg1_ad_by_day_density_30)
# For Density_40
ad_irr_by_day_density_40 <- subset(ad_irr_by_day2, ad_irr_by_day2$Density_level ==
"Density_40")
ad_irr_by_day_density_40
reg1_ad_by_day_density_40 <- lm(Irr_mean_ad ~ Day, data= ad_irr_by_day_density_40)
summary(reg1_ad_by_day_density_40)
# For Density_60
ad_irr_by_day_density_60 <- subset(ad_irr_by_day2, ad_irr_by_day2$Density_level ==
"Density_60")
ad_irr_by_day_density_60
reg1_ad_by_day_density_60 <- lm(Irr_mean_ad ~ Day, data= ad_irr_by_day_density_60)
summary(reg1_ad_by_day_density_60)

#####################################################
##### We look at the possible differences between sexes #####
##### in the hamster's irritability response to the adults ######
#####################################################
################################################################
#######
#### NO RESULTS FOUND: There are no  irritability values for males (only for females) ##
################################################################
#######

#####################################################
##### We analyze the average response of the irritability #####
##### of the hamster to increased density of kissing bugs #####
```

```
#####################################################
##################################################################
########
##### We look now at the average relationship irritability as a function of density #####
###### By average it is meant over all three experimental days and all replicates ######
##################################################################
########
##### For stage 5 nymphs
n5_irr_by_dens <- datir_ni %>% group_by(Density) %>%
summarise(Irr_mean_n5= mean(Irritability))
n5_irr_by_dens
##### For adults
ad_irr_by_dens <- datir_ad %>% group_by(Density) %>%
summarise(Irr_mean_ad= mean(Irritability))
ad_irr_by_dens
# Look at a quick and dirty graph
par(mfrow = c(1, 1))
plot(n5_irr_by_dens$Irr_mean_n5 ~ n5_irr_by_dens$Density, pch=16, xlab= "Density
(Number of stage 5 nymphs/hamster)", ylab= "Irritability level of hamster")
plot(ad_irr_by_dens$Irr_mean_ad ~ ad_irr_by_dens$Density, pch=16, xlab= "Density (Number of
adults/hamster)", ylab= "Irritability level of hamster")

##################################################################
#
##### We fit the irritability of the hamster to the density to two models: #####
##### the monomolecular model (= natural growth function or negative #####
############### exponential) and a 2nd degree polynomial ##### #########
##################################################################
#

#### For Stage 5 nymphs
##### Fit to the monomolecular model
##### Using average of all replicates and days
ModelFunction <- function(x, a, b) (a*(1-exp(-b*x)))
fityn5_mon <- nls(Irr_mean_n5 ~ ModelFunction(Density,a,b), data= n5_irr_by_dens, start=list(a= 6,
b= 0.03))
summary(fityn5_mon) xaxis <- seq(10,100)
expected_n5_mon <- ModelFunction(xaxis,5.45173,0.04942)
plot(n5_irr_by_dens$Irr_mean_n5 ~ n5_irr_by_dens$Density, pch=16, xlim= c(0,100), ylim= c(0,6))
lines(expected_n5_mon ~ xaxis)

### 2nd degree polynomial
##### Using average of all replicates and days
ModelFunction <- function(x, a, b, c) (a*x^2+b*x+c)
fityn5_pol <- nls(Irr_mean_n5 ~ ModelFunction(Density,a,b,c), data= n5_irr_by_dens, start=list(a= 6,
b= 0.03, c= 1))
summary(fityn5_pol) xaxis <- seq(10,100)
expected_n5_pol <- ModelFunction(xaxis,-0.003581,0.315372,-1.515464)
plot(n5_irr_by_dens$Irr_mean_n5 ~ n5_irr_by_dens$Density, pch=16, xlim= c(0,100), ylim= c(0,6),
xlab="Density (number of stage 5 nymphs/hamster)", ylab= "Irritability level of hamster")
lines(expected_n5_pol ~ xaxis, col="red") lines(expected_n5_mon ~ xaxis, col="blue")
legend(3, 1, legend=c("2nd degree polynomial", "Monomolecular model"), col=c("red", "blue"), lty=1:1,
cex=0.8, box.lty=0)

##################################################################
#######
##### We repeat the analysis using the standard deviation on the average irritability ##
```

```
#################################################################
#######
#### For stage 5 nymphs
n5_irr_by_dens_sd <- datir_ni %>%
group_by(Density) %>%
summarise_at(vars(Irritability), funs(n(), mean, sd))
colnames(n5_irr_by_dens_sd) <- c("Density", "N5_Irrit_N", "N5_Irrit_mean",
"N5_Irrit_sd") n5_irr_by_dens_sd n <- n5_irr_by_dens_sd$N5_Irrit_N s <-
n5_irr_by_dens_sd$N5_Irrit_sd xmean <- n5_irr_by_dens_sd$N5_Irrit_mean
margin <- qt(0.975,df=n-1)*s/sqrt(n) lowerinterval <- xmean - margin upperinterval <- xmean +
margin
lowerinterval xmean
upperinterval xaxis <- seq(0,80, by=0.1) plot.new() plot(n5_irr_by_dens_sd$N5_Irrit_mean ~
n5_irr_by_dens_sd$Density, xlab="Density (number of bugs/hamster", ylab="Hamster's irritability level",
font.main = 1, main="Stage 5 nymphs", pch=15, cex=1.5,ylim= c(-3,10), xlim= c(0,70))

# Monomol model
ModelFunction <- function(x, a, b) (a*(1-exp(-b*x)))
N5_Irrit_mean_est <- ModelFunction(xaxis,5.45173,0.04942)
lines(N5_Irrit_mean_est ~ xaxis, col= "blue", lwd= 2)
# 2nd degree polynomial
ModelFunction <- function(x, a, b, c) (a*x^2+b*x+c)
expected_n5_pol <- ModelFunction(xaxis,-0.003581,0.315372,-1.515464) lines(expected_n5_pol ~
xaxis, col= "red", lwd= 2)
points(Irritability ~ jitter(Density), pch = 1, col ="black", data= datir_ni)
arrows(x0=n5_irr_by_dens_sd$Density, y0=n5_irr_by_dens_sd$N5_Irrit_mean - margin,
x1=n5_irr_by_dens_sd$Density, y1=n5_irr_by_dens_sd$N5_Irrit_mean + margin, angle=90, code=3,
length=0.05)

#### For adults
##### Fit to the monomolecular model
##### Using average of all replicates and days
ModelFunction <- function(x, a, b) (a*(1-exp(-b*x)))
fityad_mon <- nls(Irr_mean_ad ~ ModelFunction(Density,a,b), data= ad_irr_by_dens, start=list(a= 6,
b= 0.03))
summary(fityad_mon) xaxis <- seq(10,80)
expected_ad_mon <- ModelFunction(xaxis,5.09029,0.04658)
plot(ad_irr_by_dens$Irr_mean_ad ~ ad_irr_by_dens$Density, pch=16, xlim= c(0,100), ylim= c(0,6))
lines(expected_ad_mon ~ xaxis)
### 2nd degree polynomial
##### Using average of all replicates and days
ModelFunction <- function(x, a, b, c) (a*x^2+b*x+c)
fityad_pol <- nls(Irr_mean_ad ~ ModelFunction(Density,a,b,c), data= ad_irr_by_dens, start=list(a= 6,
b= 0.03, c= 1)) summary(fityad_pol) xaxis <- seq(10,80)
expected_ad_pol <- ModelFunction(xaxis,-0.0034751,0.3033935,-1.6048110)
plot(ad_irr_by_dens$Irr_mean_ad ~ ad_irr_by_dens$Density, pch=16, xlim= c(0,100), ylim= c(0,6),
xlab="Density (number of adults/hamster)", ylab= "Irritability level of hamster") lines(expected_ad_pol ~
xaxis, col="red") lines(expected_ad_mon ~ xaxis, col="blue")
legend(3, 1, legend=c("2nd degree polynomial", "Monomolecular model"), col=c("red", "blue"), lty=1:1,
cex=0.8, box.lty=0)

#################################################################
######
##### We repeat the analysis using the standard deviation on the average irritability ##
#################################################################
#######
ad_irr_by_dens_sd <- datir_ad %>%
```

```
group_by(Density) %>%
summarise_at(vars(Irritability), funs(n(), mean, sd))
colnames(ad_irr_by_dens_sd) <- c("Density", "ad_Irrit_N", "ad_Irrit_mean", "ad_Irrit_sd"
)
ad_irr_by_dens_sd n <- ad_irr_by_dens_sd$ad_Irrit_N s <- ad_irr_by_dens_sd$ad_Irrit_sd xmean <-
ad_irr_by_dens_sd$ad_Irrit_mean margin <- qt(0.975,df=n-1)*s/sqrt(n) lowerinterval <- xmean -
margin upperinterval <- xmean + margin
lowerinterval xmean
upperinterval xaxis <- seq(0,80, by=0.1) plot.new() plot(ad_irr_by_dens_sd$ad_Irrit_mean ~
ad_irr_by_dens_sd$Density, xlab="Density
(number of bugs/hamster", ylab="Hamster's irritability level", font.main = 1, main= "Adults", pch=15,
cex=1.5,ylim= c(-3,10), xlim= c(0,70))
# Monomol model
ModelFunction <- function(x, a, b) (a*(1-exp(-b*x))) ad_Irrit_mean_est <-
ModelFunction(xaxis,5.09029,0.04658) lines(ad_Irrit_mean_est ~ xaxis, col= "blue", lwd= 2)
# 2nd degree polynomial
ModelFunction <- function(x, a, b, c) (a*x^2+b*x+c)
expected_ad_pol <- ModelFunction(xaxis,-0.0034751,0.3033935,-1.6048110) lines(expected_ad_pol ~
xaxis, col= "red", lwd= 2)
points(Irritability ~ jitter(Density), pch = 1, col ="black", data= datir_ad)
arrows(x0=ad_irr_by_dens_sd$Density, y0=ad_irr_by_dens_sd$ad_Irrit_mean - margin,
x1=ad_irr_by_dens_sd$Density, y1=ad_irr_by_dens_sd$ad_Irrit_mean + margin, angle=90, code=3,
length=0.05)

#################################################################
#######
#### We plot the expected values of the two models for stage 5 nymphs and adults ####
#################################################################
#######
xaxis <- seq(0,80, by=0.1) plot.new() plot(ad_irr_by_dens_sd$ad_Irrit_mean ~
ad_irr_by_dens_sd$Density, xlab="Density
(number of bugs/hamster)", ylab="Hamster's irritability level", pch=16, cex=1.5, col=
"blue", ylim= c(-3,10), xlim= c(0,70))
points(n5_irr_by_dens_sd$N5_Irrit_mean ~ n5_irr_by_dens_sd$Density, pch=16, cex=1.5 , col= "red")
###################
## Monomol model ##
###################
ModelFunction <- function(x, a, b) (a*(1-exp(-b*x))) ad_Irrit_mean_est <-
ModelFunction(xaxis,5.09029,0.04658)
lines(ad_Irrit_mean_est ~ xaxis, col= "blue", lwd= 2)
N5_Irrit_mean_est <- ModelFunction(xaxis,5.09029,0.04942)
lines(N5_Irrit_mean_est ~ xaxis, col= "red", lwd= 2)
########################
## 2nd degree polynomial ##
########################
ModelFunction <- function(x, a, b, c) (a*x^2+b*x+c)
expected_ad_pol <- ModelFunction(xaxis,-0.0034751,0.3033935,-1.6048110) lines(expected_ad_pol ~
xaxis, col= "blue", lty= "dashed", lwd= 2) expected_n5_pol <- ModelFunction(xaxis,-
0.003581,0.315372,-1.515464)
lines(expected_n5_pol ~ xaxis, col= "red", lty= "dashed", lwd= 2)

#################################################################
#######
###### We fit the data to the two models for stage 5 nymphs and adults pooled ######
#################################################################
#######
pool_stages_irr_2_models <- datir_ni_ad %>%
```

```
group_by(Density) %>%
summarise_at(vars(Irritability), funs(n(), mean, sd))
pool_stages_irr_2_models
n <- pool_stages_irr_2_models$n
s <- pool_stages_irr_2_models$sd
xmean <- pool_stages_irr_2_models$mean
margin <- qt(0.975,df=n-1)*s/sqrt(n)
lowerinterval <- xmean - margin
upperinterval <- xmean + margin
lowerinterval
xmean
upperinterval

##### Fit of the pooled data to the monomolecular model
##### Using average of all replicates and days
ModelFunction <- function(x, a, b) (a*(1-exp(-b*x)))
fitypool_mon <- nls(mean ~ ModelFunction(Density,a,b), data=
pool_stages_irr_2_models, start=list(a= 6, b= 0.03))
summary(fitypool_mon)
### 2nd degree polynomial
##### Using average of all replicates and days
ModelFunction <- function(x, a, b, c) (a*x^2+b*x+c)
fitypool_pol <- nls(mean ~ ModelFunction(Density,a,b,c), data= pool_stages_irr_2_models, start=list(a=
6, b= 0.03, c= 1))
summary(fitypool_pol) xaxis <- seq(0,80, by=0.1)
plot.new()
plot(pool_stages_irr_2_models$mean ~ pool_stages_irr_2_models$Density, xlab= "Density (number of
bugs/hamster", ylab="Hamster's irritability level", font.main = 1, main="Pool of stage 5 nymphs and
adults", pch=15, cex=1.5,ylim= c(-3,10), xlim= c(0,70))
# Monomol model
ModelFunction <- function(x, a, b) (a*(1-exp(-b*x)))
Pool_Irrit_mean_est <- ModelFunction(xaxis,5.27031, 0.04804)
lines(Pool_Irrit_mean_est ~ xaxis, col= "blue", lwd= 2)
# 2nd degree polynomial
ModelFunction <- function(x, a, b, c) (a*x^2+b*x+c)
expected_Pool_pol <- ModelFunction(xaxis,-0.0035279, 0.3093827, -1.5601374)
lines(expected_Pool_pol ~ xaxis, col= "red", lwd= 2)
points(Irritability ~ jitter(Density), pch = 1, col ="black", data= datir_ni_ad)
arrows(x0=pool_stages_irr_2_models$Density,y0=pool_stages_irr_2_models$mean margin,
x1=pool_stages_irr_2_models$Density, y1=pool_stages_irr_2_models$mean + margin, angle=90,
code=3, length=0.05)
#################################################################
######

######################################
##### Analysis of only feeding variables #####
###################################### names(feeding)
##### We select Stage 5 nymphs
feeding_n5_0 <- subset(feeding, feeding$Stage == 5)
feeding_n5 <- feeding_n5_0[,-4] feeding_n5

#################################################################
##
##### Analysis 1: percent of individuals feeding each of the first three days #####
#################################################################
##
```

```
###########################
##### For stage 5 nymphs #####
###########################
feeding_n5_by_day <- feeding_n5[37:72,]
feeding_n5_by_day
names(feeding_n5_by_day)
position <- c(1,2,4:8)
feeding_n5_by_day_Pct <- feeding_n5_by_day[,position]
feeding_n5_by_day_Pct
feeding_n5_by_day_Pct2 <- feeding_n5_by_day_Pct %>%
        mutate(Pct_feeding_Day1 = N_feeding_Day1/Density*100, Pct_feeding_Day2 =
        N_feeding_Day2/Density*100, Pct_feeding_Day3 = N_feeding_Day3/Density*100,
        Pct_feeding_3_Days = N_feeding_3_Days/Density*100)
feeding_n5_by_day_Pct2
position <- c(4:7)
feeding_n5_by_day_Pct3 <- feeding_n5_by_day_Pct2[,-position] feeding_n5_by_day_Pct3

# Delete all the rows containing any NAs:
feeding_n5_by_day_Pct4 <- feeding_n5_by_day_Pct3[complete.cases( feeding_n5_by_day_Pct3), ]
feeding_n5_by_day_Pct4
feeding_n5_by_day_Pct5 <- feeding_n5_by_day_Pct4 %>%
group_by(Density) %>%
summarise_at(vars(Irritability, Pct_feeding_Day1, Pct_feeding_Day2, Pct_feeding_Day3,
Pct_feeding_3_Days), funs(n(), mean, sd))
feeding_n5_by_day_Pct5
names(feeding_n5_by_day_Pct5)
position <- c(3:5)
feeding_n5_by_day_Pct6 <- feeding_n5_by_day_Pct5[,-position] feeding_n5_by_day_Pct7 <-
as.data.frame(feeding_n5_by_day_Pct6[,-3]) feeding_n5_by_day_Pct7
plot(Pct_feeding_Day1_mean ~ Density, data= feeding_n5_by_day_Pct7, lty=1, col= "black")
###############################################################
##### Effect of day in percent of bugs feeding, for each density #####
###############################################################
###########################
##### For stage 5 nymphs #####
###########################
names(feeding_n5_by_day_Pct7)
Long_feed_n5_day <- feeding_n5_by_day_Pct7 %>%
pivot_longer(Pct_feeding_Day1_mean:Pct_feeding_Day2_mean:
Pct_feeding_Day3_mean, names_to = "Pct_feeding_by_time", values_to =
"response_values")
print(Long_feed_n5_day)
Long_feed_n5_day_2 <- as.data.frame(Long_feed_n5_day)
Long_feed_n5_day_2
# Create the day number for each density
day_num <- rep(c(1,2,3),5)
day_num
Long_feed_n5_day_3 <- cbind(Long_feed_n5_day_2,day_num)
Long_feed_n5_day_3
Density_level <- as.factor(c("Density_10", "Density_10", "Density_10", "Density_20",
"Density_20", "Density_20", "Density_30", "Density_30", "Density_30", "Density_40",
"Density_40", "Density_40", "Density_60", "Density_60", "Density_60"))
Long_feed_n5_day_4 <- cbind(Long_feed_n5_day_3,Density_level) Long_feed_n5_day_4
qplot(x = day_num, y = response_values, facets = ~Density_level, data =
Long_feed_n5_day_4) + labs(x = "Trial day", y = "Percent of stage 5 nymphs feeding") +
geom_smooth(method = "lm")
```

```
###########################
##### For the adult stage ######
###########################
##### We select the adult stage
feeding_ad_0 <- subset(feeding, feeding$Stage == 6)
feeding_ad <- feeding_ad_0[,-4] feeding_ad
names(feeding_ad)
feeding_ad2 <- subset(feeding_ad, feeding_ad$N_feeding_Day1 != 0)
position <- c(1:2,4:8)
feeding_ad_by_day <- feeding_ad2[,position]
feeding_ad_by_day names(feeding_ad_by_day)
feeding_ad_by_day_Pct2 <- feeding_ad_by_day %>%
mutate(Pct_feeding_Day1 = N_feeding_Day1/Density*100, Pct_feeding_Day2 =
N_feeding_Day2/Density*100, Pct_feeding_Day3 = N_feeding_Day3/Density*100,
Pct_feeding_3_Days = N_feeding_3_Days/Density*100)
feeding_ad_by_day_Pct2
position <- c(4:7)
feeding_ad_by_day_Pct3 <- feeding_ad_by_day_Pct2[,-position] feeding_ad_by_day_Pct3
# Delete all the rows containing any NAs:
feeding_ad_by_day_Pct5 <- feeding_ad_by_day_Pct3 %>%
        group_by(Density) %>%
        summarise_at(vars(Irritability, Pct_feeding_Day1, Pct_feeding_Day2,
        Pct_feeding_Day3, Pct_feeding_3_Days), funs(n(), mean, sd))
feeding_ad_by_day_Pct5
names(feeding_ad_by_day_Pct5)
position <- c(3:5)
feeding_ad_by_day_Pct6 <- feeding_ad_by_day_Pct5[,-position] feeding_ad_by_day_Pct7 <-
as.data.frame(feeding_ad_by_day_Pct6[,-3]) feeding_ad_by_day_Pct7
plot(Pct_feeding_Day1_mean ~ Density, data= feeding_ad_by_day_Pct7, pch=16, col= "black",
main="Adults")
#############################################################
######
names(feeding_ad_by_day_Pct7)
Long_feed_day_ad <- feeding_ad_by_day_Pct7 %>%
pivot_longer(Pct_feeding_Day1_mean:Pct_feeding_Day2_mean: Pct_feeding_Day3_mean, names_to =
"Pct_feeding_by_day", values_to =
"response_values")
print(Long_feed_day_ad)
Long_feed_day_ad_2 <- as.data.frame(Long_feed_day_ad)
Long_feed_day_ad_2
# Create the day number for each density
day_num <- rep(c(1,2,3),5)
day_num
Long_feed_day_ad_3 <- cbind(Long_feed_day_ad_2,day_num)
Long_feed_day_ad_3
Density_level <- as.factor(c("Density_10", "Density_10", "Density_10", "Density_20",
"Density_20", "Density_20", "Density_30", "Density_30", "Density_30", "Density_40",
"Density_40", "Density_40", "Density_60", "Density_60", "Density_60"))
Long_feed_day_ad_4 <- cbind(Long_feed_day_ad_3,Density_level)
Long_feed_day_ad_4
qplot(x = day_num, y = response_values, facets = ~Density_level, data =
Long_feed_day_ad_4) + labs(x = "Trial day", y = "Percent of adults feeding") + geom_smooth(method
= "lm")
Long_feed_day_ad_5 <- Long_feed_day_ad_4 %>%
        group_by(Density,day_num) %>%
        summarise_at(vars(Irritability_mean, response_values,
        Pct_feeding_3_Days_mean), funs(n(), mean, sd))
```

```
names(Long_feed_day_ad_5)
position <- c(1,2,6:8)
Long_feed_day_ad_6 <- Long_feed_day_ad_5[,position]
Long_feed_day_ad_6
# Create data by day
Long_feed_day_ad_6_Day1 <- subset(Long_feed_day_ad_6, Long_feed_day_ad_6[,2] ==
1)
names(Long_feed_day_ad_6_Day1)[names(Long_feed_day_ad_6_Day1) ==
"response_values_mean"] <- "Pct_fed_Day1"
Long_feed_day_ad_6_Day1
Long_feed_day_ad_6_Day2 <- subset(Long_feed_day_ad_6, Long_feed_day_ad_6[,2] ==
2)
names(Long_feed_day_ad_6_Day2)[names(Long_feed_day_ad_6_Day2) ==
"response_values_mean"] <- "Pct_fed_Day2"
Long_feed_day_ad_6_Day2
Long_feed_day_ad_6_Day3 <- subset(Long_feed_day_ad_6, Long_feed_day_ad_6[,2] ==
3)
names(Long_feed_day_ad_6_Day3)[names(Long_feed_day_ad_6_Day3) ==
"response_values_mean"] <- "Pct_fed_Day3"
Long_feed_day_ad_6_Day3
Long_feed_day_ad_6_Day1 <- Long_feed_day_ad_6_Day1 [,-2]
Long_feed_day_ad_6_Day2 <- Long_feed_day_ad_6_Day2 [,-2]
Long_feed_day_ad_6_Day3 <- Long_feed_day_ad_6_Day3 [,-2]
Long_feed_day_ad_6_Day1
Long_feed_day_ad_6_Day2 Long_feed_day_ad_6_Day3
reg_pct_fed_D1 <- lm(Pct_fed_Day1 ~ Density, data= Long_feed_day_ad_6_Day1)
summary(reg_pct_fed_D1)
reg_pct_fed_D2 <- lm(Pct_fed_Day2 ~ Density, data= Long_feed_day_ad_6_Day2)
summary(reg_pct_fed_D2)
reg_pct_fed_D3 <- lm(Pct_fed_Day3 ~ Density, data= Long_feed_day_ad_6_Day3)
summary(reg_pct_fed_D3)
plot(Pct_fed_Day1 ~ Density, data= Long_feed_day_ad_6_Day1, pch=16, xlab= "Density
(Number of adults/hamster", ylab="Percent bugs feeding on day 1") abline(reg_pct_fed_D1)
plot(Pct_fed_Day2 ~ Density, data= Long_feed_day_ad_6_Day2, pch=16, xlab= "Density
(Number of adults/hamster", ylab="Percent bugs feeding on day 2") abline(reg_pct_fed_D2)
plot(Pct_fed_Day3 ~ Density, data= Long_feed_day_ad_6_Day3, pch=16, xlab= "Density
(Number of adults/hamster", ylab="Percent bugs feeding on day 3") abline(reg_pct_fed_D3)

#########################################
#####   Evaluation   of   the   trial   day   effect   in   adults   #####
#########################################
reg_day_effect_ad <- lm(response_values_mean ~ Density * day_num, data= Long_feed_day_ad_6)
summary(reg_day_effect_ad)

###########################################################
#####
####  We  repeat  the  analysis  of  the  trial  day  effect  in  adults  pooling  densities  ######
###########################################################
##### day_effect_pct_feed_ad_all_dens <- Long_feed_day_ad_6 %>%
group_by(day_num) %>%
summarise(Pct_feed_all_dens = mean(response_values_mean))
day_effect_pct_feed_ad_all_dens <- as.data.frame(day_effect_pct_feed_ad_all_dens)
day_effect_pct_feed_ad_all_dens
reg_day_effect_ad_all_dens <- lm(Pct_feed_all_dens ~ day_num, data=
day_effect_pct_feed_ad_all_dens)
summary(reg_day_effect_ad_all_dens)
```

```
#############################################
###### Density effect by pooling the trial day ######
#############################################
Density_effect_pct_feed_AD_all_days <- Long_feed_day_ad_6 %>%
group_by(Density) %>%
summarise(Pct_feed_all_days_AD = mean(Pct_feeding_3_Days_mean_mean))
Density_effect_pct_feed_AD_all_days <- as.data.frame(
Density_effect_pct_feed_AD_all_days)
reg_density_effect_AD_all_days <- lm(Pct_feed_all_days_AD ~ Density, data=
Density_effect_pct_feed_AD_all_days)
summary(reg_density_effect_AD_all_days)
Density_effect_pct_feed_AD_all_days_PROP <- Density_effect_pct_feed_AD_all_days
Density_effect_pct_feed_AD_all_days_PROP
Density_effect_pct_feed_AD_all_days_PROP <- Density_effect_pct_feed_AD_all_days
Density_effect_pct_feed_AD_all_days_PROP$Pct_feed_all_days_AD <-
Density_effect_pct_feed_AD_all_days_PROP$Pct_feed_all_days_AD/100
Density_effect_pct_feed_AD_all_days_PROP
Density_effect_pct_feed_AD_all_days_PROP$Pct_feed_all_days_AD <- as.numeric(
Density_effect_pct_feed_AD_all_days_PROP$Pct_feed_all_days_AD)
for (j in 1:5) {
if (Density_effect_pct_feed_AD_all_days_PROP$Pct_feed_all_days_AD[j] > 1)
Density_effect_pct_feed_AD_all_days_PROP$Pct_feed_all_days_AD[j] <-0.99
if (Density_effect_pct_feed_AD_all_days_PROP$Pct_feed_all_days_AD[j] < 0)
Density_effect_pct_feed_AD_all_days_PROP$Pct_feed_all_days_AD[j] <-0 }
Density_effect_pct_feed_AD_all_days_PROP
library(betareg)
Density_effect_pct_feed_AD_all_days_PROP_logit <- betareg(Pct_feed_all_days_AD ~ Density, data =
Density_effect_pct_feed_AD_all_days_PROP)
summary(Density_effect_pct_feed_AD_all_days_PROP_logit)
position <- c(1:2, 35:38)
per_day <- feeding[158:174,position]
per_day
per_day2 <- per_day %>%
group_by(Density) %>%
summarise(Prop_feed_all_days = mean(Pct_Fed_3Days/100)) per_day2 <- as.data.frame(per_day2)
per_day2
for (j in 1:5) {
if (per_day2$Prop_feed_all_days[j] > 1) per_day2$Prop_feed_all_days[j] <-0.99
if (per_day2$Prop_feed_all_days[j] < 0) per_day2$Prop_feed_all_days[j] <-0 }
per_day2

##### REGULAR LINEAR REGRESSION #####
reg_per_day2 <- lm(Prop_feed_all_days ~ Density, data=per_day2) summary(reg_per_day2)
##### BETA LINEAR REGRESSION #####
reg_per_day3 <- betareg(Prop_feed_all_days ~ Density, data = per_day2) summary(reg_per_day3)

#####################################
##### Regressions for stage 5 nymphs #####
#####################################
names(Long_feed_n5_day_4)
reg1_n5_feed <- lm(response_values ~ day_num * Density, data= Long_feed_n5_day_4)
summary(reg1_n5_feed)
reg1_n5_feed_2 <- lm(response_values ~ day_num, data= Long_feed_n5_day_4)
summary(reg1_n5_feed_2)
#####################################
```

```
##### Regressions for the adult stage #####
######################################
names(Long_feed_day_ad_4)
reg1_ad_feed <- lm(response_values ~ day_num * Density, data= Long_feed_day_ad_4)
summary(reg1_ad_feed)
reg1_ad_feed_2 <- lm(response_values ~ day_num, data= Long_feed_day_ad_4)
summary(reg1_ad_feed_2)
#######################################################
##### We check the significance of the slope of the linear ######
######## regression of Pct feeding as a function of day ########
#######################################################
# We select the datasets for each experimental density level
# For Density_10
ad_feed_by_day_density_10 <- subset(Long_feed_day_ad_4, Long_feed_day_ad_4$
Density_level == "Density_10") ad_feed_by_day_density_10
reg1_feed_ad_by_day_density_10 <- lm(response_values ~ day_num, data=
ad_feed_by_day_density_10)
summary(reg1_ad_by_day_density_10)
# For Density_20
ad_feed_by_day_density_20 <- subset(Long_feed_day_ad_4, Long_feed_day_ad_4$
Density_level == "Density_20") ad_feed_by_day_density_20
reg1_feed_ad_by_day_density_20 <- lm(response_values ~ day_num, data=
ad_feed_by_day_density_20)
summary(reg1_ad_by_day_density_20)
# For Density_30
ad_feed_by_day_density_30 <- subset(Long_feed_day_ad_4, Long_feed_day_ad_4$
Density_level == "Density_30") ad_feed_by_day_density_30
reg1_feed_ad_by_day_density_30 <- lm(response_values ~ day_num, data=
ad_feed_by_day_density_30)
summary(reg1_ad_by_day_density_30)
# For Density_40
ad_feed_by_day_density_40 <- subset(Long_feed_day_ad_4, Long_feed_day_ad_4$
Density_level == "Density_40") ad_feed_by_day_density_40
reg1_feed_ad_by_day_density_40 <- lm(response_values ~ day_num, data=
ad_feed_by_day_density_40)
summary(reg1_ad_by_day_density_40)
# For Density_60
ad_feed_by_day_density_60 <- subset(Long_feed_day_ad_4, Long_feed_day_ad_4$
Density_level == "Density_60")
ad_feed_by_day_density_60
reg1_feed_ad_by_day_density_60 <- lm(response_values ~ day_num, data=
ad_feed_by_day_density_60)
summary(reg1_ad_by_day_density_60)

###############################################
##### Plot of the effects of Density on the overall #####
##### Pct bugs feeding in three consecutive days #####
###############################################


############################
##### For stage 5 nymphs ######
############################
plot(Pct_feeding_3_Days_mean ~ Density, pch=16, data=Long_feed_n5_day_4, main=
"Stage 5 nymphs")
plot(Pct_feeding_3_Days_mean ~ Irritability_mean, pch=16, data=Long_feed_n5_day_4, main= "Stage
5 nymphs")
```

```
############################
###### For the adult stage ######
############################
plot(Pct_feeding_3_Days_mean ~ Density, pch=16,  data= Long_feed_day_ad_4, main=
"Adults")
plot(Pct_feeding_3_Days_mean  ~  Irritability_mean,  pch=16,   data=  Long_feed_day_ad_4  ,  main=
"Adults")
###################################
############### Analyse ##############
####### Pct_feeding_once_in_3d #######
####### Pct_feeding_twice_in_3d #######
####### Pct_feeding_thrice_in_3d ######
###################################
# We select the appropriate variables from N5 and Ad position <- c(1:4, 9:11)
feeding_n5_freq <- feeding_n5[,position] feeding_n5_freq
feeding_n5_freq2 <- subset(feeding_n5_freq, feeding_n5_freq$Pct_feeding_once_in_3d
!= "NA")
feeding_ad_freq <- feeding_ad[,position] feeding_ad_freq
feeding_ad_freq2 <- subset(feeding_ad_freq, feeding_ad_freq$Pct_feeding_once_in_3d != "NA")
##########################################
##### Inserting the Pct feeding by day analysis ####
##########################################
###########################
##### For stage 5 nymphs #####
###########################
Long_feed_n5_times_in_3_days                    <-                    feeding_n5_freq2                    %>%
pivot_longer(Pct_feeding_once_in_3d:Pct_feeding_twice_in_3d:
Pct_feeding_thrice_in_3d, names_to = "Pct_feeding_x_times_in_3_days", values_to =
"response_values")
print(Long_feed_n5_times_in_3_days)
Long_feed_n5_times_in_3_days <- as.data.frame(Long_feed_n5_times_in_3_days)
Long_feed_n5_times_in_3_days
Long_feed_n5_times_in_3_days2 <- Long_feed_n5_times_in_3_days %>% group_by(Density, Replicate)
%>%
summarise(Irr_mean_n5_all= mean(Irritability), mean_response_values= mean(response_values))
Long_feed_n5_times_in_3_days2
Density_level <- as.factor(c("Density_10", "Density_10", "Density_10", "Density_10",
"Density_20", "Density_20", "Density_20", "Density_20",  "Density_30", "Density_30",
"Density_30", "Density_30", "Density_40", "Density_40", "Density_40",  "Density_40",
"Density_40", "Density_60"))
Long_feed_n5_times_in_3_days3 <- cbind(Long_feed_n5_times_in_3_days2, Density_level)
Long_feed_n5_times_in_3_days3 # Rename the "...5" column
colnames(Long_feed_n5_times_in_3_days3) <- c("Density", "Replicate",
"Irr_mean_n5_all", "mean_response_values", "Pct_feeding_x_times_in_3_days") qplot(x = Density, y =
response_values, facets = ~ Pct_feeding_x_times_in_3_days, data=
Long_feed_n5_times_in_3_days) + labs(x = "Density (stage5 nymphs/hamster)", y = "Percent of stage 5
nymphs feeding in
1, 2, or 3 d ays") +
geom_smooth(method = "lm")
reg1_n5_times_in_3_days     <-     lm(response_values     ~     Replicate     *     Density,     data=
Long_feed_n5_times_in_3_days) summary(reg1_n5_times_in_3_days)
##################
##### For Adults ####
##################
Long_feed_ad_times_in_3_days                    <-                    feeding_ad_freq2                    %>%
pivot_longer(Pct_feeding_once_in_3d:Pct_feeding_twice_in_3d:
Pct_feeding_thrice_in_3d, names_to = "Pct_feeding_x_times_in_3_days", values_to ="response_values")
```

```
print(Long_feed_ad_times_in_3_days)
Long_feed_ad_times_in_3_days <- as.data.frame(Long_feed_ad_times_in_3_days)
Long_feed_ad_times_in_3_days
Long_feed_ad_times_in_3_days2 <- Long_feed_ad_times_in_3_days %>% group_by(Density, Replicate)
%>%
summarise(Irr_mean_ad_all= mean(Irritability), mean_response_values= mean(response_values))
Long_feed_ad_times_in_3_days2
Density_level <- as.factor(c("Density_10", "Density_10", "Density_10", "Density_10",
"Density_20", "Density_20", "Density_20", "Density_20",  "Density_30", "Density_30",
"Density_30", "Density_30", "Density_40", "Density_40", "Density_40",  "Density_40",  "Density_60"))
Long_feed_ad_times_in_3_days3 <- cbind(Long_feed_ad_times_in_3_days2, Density_level)
Long_feed_ad_times_in_3_days3 # Rename the "...5" column
colnames(Long_feed_ad_times_in_3_days3) <- c("Density", "Replicate",
"Irr_mean_ad_all", "mean_response_values", "Pct_feeding_x_times_in_3_days") qplot(x = Density, y =
response_values, facets = ~ Pct_feeding_x_times_in_3_days, data=
Long_feed_ad_times_in_3_days) + labs(x = "Density (adults/hamster)", y = "Percent of adults feeding in
1, 2, or 3 d ays") + geom_smooth(method = "lm")
reg1_ad_times_in_3_days <- lm(response_values ~ Replicate * Density, data=
Long_feed_ad_times_in_3_days)
summary(reg1_ad_times_in_3_days)
# Regression using only the subset of Pct_feeding_once_in_3d (FOR ADULTS)
Long_feed_ad_times_in_3_days_once <- subset(Long_feed_ad_times_in_3_days,
Long_feed_ad_times_in_3_days$Pct_feeding_x_times_in_3_days ==
"Pct_feeding_once_in_3d") reg1_ad_times_in_3_days_once <- lm(response_values ~ Density, data=
Long_feed_ad_times_in_3_days_once) summary(reg1_ad_times_in_3_days_once)
# Regression using only the subset of Pct_feeding_twice_in_3d (FOR ADULTS)
Long_feed_ad_times_in_3_days_twice <- subset(Long_feed_ad_times_in_3_days,
Long_feed_ad_times_in_3_days$Pct_feeding_x_times_in_3_days ==
"Pct_feeding_twice_in_3d") reg1_ad_times_in_3_days_twice <- lm(response_values ~ Density, data=
Long_feed_ad_times_in_3_days_twice) summary(reg1_ad_times_in_3_days_twice)
# Regression using only the subset of Pct_feeding_thrice_in_3d (FOR ADULTS)
Long_feed_ad_times_in_3_days_thrice <- subset(Long_feed_ad_times_in_3_days,
Long_feed_ad_times_in_3_days$Pct_feeding_x_times_in_3_days ==
"Pct_feeding_thrice_in_3d") reg1_ad_times_in_3_days_thrice <- lm(response_values ~ Density, data=
Long_feed_ad_times_in_3_days_thrice) summary(reg1_ad_times_in_3_days_thrice)
############################################################
#####
##### Regression using only the subset of Pct_feeding_once_in_3d (FOR N5) #####
############################################################
#####
Long_feed_n5_times_in_3_days_once <- subset(Long_feed_n5_times_in_3_days,
Long_feed_n5_times_in_3_days$Pct_feeding_x_times_in_3_days ==
"Pct_feeding_once_in_3d") reg1_n5_times_in_3_days_once <- lm(response_values ~ Density, data=
Long_feed_n5_times_in_3_days_once) summary(reg1_n5_times_in_3_days_once)
############################################################
#####
##### Regression using only the subset of Pct_feeding_twice_in_3d (FOR N5) #####
############################################################
#####
Long_feed_n5_times_in_3_days_twice <- subset(Long_feed_n5_times_in_3_days,
Long_feed_n5_times_in_3_days$Pct_feeding_x_times_in_3_days ==
"Pct_feeding_twice_in_3d") reg1_n5_times_in_3_days_twice <- lm(response_values ~ Density, data=
Long_feed_n5_times_in_3_days_twice) summary(reg1_n5_times_in_3_days_twice)
############################################################
#####
##### Regression using only the subset of Pct_feeding_thrice_in_3d (FOR N5) #####
```

```
#################################################################
#####      Long_feed_n5_times_in_3_days_thrice      <-      subset(Long_feed_n5_times_in_3_days,
Long_feed_n5_times_in_3_days$Pct_feeding_x_times_in_3_days ==
"Pct_feeding_thrice_in_3d") reg1_n5_times_in_3_days_thrice <- lm(response_values ~ Density, data=
Long_feed_n5_times_in_3_days_thrice) summary(reg1_n5_times_in_3_days_thrice)
#################################################################
######
#### We analyse BMS_mg_D1, BMS_mg_D2, BMS_mg_D3, Mean_BMS_mg_3Days  ###
#################################################################
######
 names(feeding_n5)
# We select the appropriate variables from N5 and Ad position <- c(1:4, 12:20)
feeding_n5_BMS_d <- feeding_n5[,position]
names(feeding_n5_BMS_d)
feeding_n5_BMS_d2 <- subset(feeding_n5_BMS_d, feeding_n5_BMS_d$
BMS_mean_mg_D1 != "NA") feeding_n5_BMS_d2
feeding_ad_BMS_d <- feeding_ad[,position]
names(feeding_ad_BMS_d)
feeding_ad_BMS_d2 <- subset(feeding_ad_BMS_d, feeding_ad_BMS_d$
BMS_mean_mg_D1 != "NA") feeding_ad_BMS_d2
###########################################
##### Inserting the Pct feeding by day analysis #####
###########################################
###########################
##### For stage 5 nymphs #####
###########################
feeding_n5_BMS_d3 <- feeding_n5_BMS_d2 %>%
        group_by(Density, Replicate) %>%
        summarise(Irr_mean_n5_all=        mean(Irritability),        mean_N=        mean(BMS_N_D1),
        Mean_BMS_mg_D1= mean(BMS_mean_mg_D1), Mean_BMS_mg_D2= mean(
        BMS_mean_mg_D2), Mean_BMS_mg_D3= mean(BMS_mean_mg_D3))
feeding_n5_BMS_d3
feeding_n5_BMS_d3 <- as.data.frame(feeding_n5_BMS_d3) feeding_n5_BMS_d3
        Long_feeding_n5_BMS_d                <-                feeding_n5_BMS_d3                %>%
        pivot_longer(Mean_BMS_mg_D1:Mean_BMS_mg_D2:Mean_BMS_mg_D3,              names_to
        ="BMS_mean_per_day_x", values_to = "response_values")
        print(Long_feeding_n5_BMS_d)
Long_feeding_n5_BMS_d2 <- as.data.frame(Long_feeding_n5_BMS_d)
Long_feeding_n5_BMS_d2
Day <- as.factor(c("Day_1", "Day_2", "Day_3", "Day_1", "Day_2", "Day_3", "Day_1",
"Day_2", "Day_3", "Day_1", "Day_2", "Day_3",
"Day_1", "Day_2", "Day_3", "Day_1", "Day_2", "Day_3", "Day_1", "Day_2", "Day_3",
"Day_1", "Day_2", "Day_3",
"Day_1", "Day_2", "Day_3", "Day_1", "Day_2", "Day_3", "Day_1", "Day_2", "Day_3",
"Day_1", "Day_2", "Day_3",
"Day_1", "Day_2", "Day_3", "Day_1", "Day_2", "Day_3", "Day_1", "Day_2", "Day_3", "Day_1", "Day_2",
"Day_3",  "Day_1", "Day_2", "Day_3",  "Day_1", "Day_2", "Day_3")) as.matrix(Day)
Long_feeding_n5_BMS_d3 <- cbind(Long_feeding_n5_BMS_d2, Day)
Long_feeding_n5_BMS_d3
qplot(x = Density, y = response_values, facets = ~ Day, data= Long_feeding_n5_BMS_d3)
+ labs(x = "Density (stage5 nymphs/hamster)", y = "Blood meal size (BMS) ingested (mg) per day") +
geom_smooth(method = "lm")
reg1_n5_BMS_day <- lm(response_values ~ Replicate * Density, data= Long_feeding_n5_BMS_d3)
summary(reg1_n5_BMS_day)
reg2_n5_BMS_day     <-     lm(response_values     ~     Density,     data=     Long_feeding_n5_BMS_d3)
summary(reg2_n5_BMS_day)
##########################################################
```

```
##### Lok at the association between Pct_feed and BMS_feed #####
############################################################
###########################
##### For Stage 5 nymhs #####
###########################
Pct_feed <- Long_feed_n5_times_in_3_days$response_values Pct_feed
BMS_feed <- Long_feeding_n5_BMS_d3$response_values
BMS_feed
length(BMS_feed)
length(Pct_feed)
Pct_feed_BMS_feed <- cbind(Pct_feed, BMS_feed)
Pct_feed_BMS_feed
head(Pct_feed_BMS_feed)
Pct_feed_BMS_feed2 <- cbind(Pct_feed_BMS_feed, Long_feed_n5_times_in_3_days$
Density, Long_feed_n5_times_in_3_days$Replicate, Long_feed_n5_times_in_3_days$ Stage,
Long_feed_n5_times_in_3_days$Irritability)
colnames(Pct_feed_BMS_feed2) <- c("Pct_feed", "BMS_feed", "Density", "Replicate", "Stage",
"Irritability")
Pct_feed_BMS_feed2 <- Pct_feed_BMS_feed2[complete.cases(Pct_feed_BMS_feed2), ]
Pct_feed_BMS_feed2
reg1_BMS_feed_Pct_feed <- lm(BMS_feed ~ Pct_feed) summary(reg1_BMS_feed_Pct_feed)
plot(BMS_feed ~ Pct_feed, pch=16)
abline(reg1_BMS_feed_Pct_feed)
###################
##### For Adults #####
###################
feeding_ad_BMS_d3 <- feeding_ad_BMS_d2 %>% group_by(Density, Replicate) %>%
summarise(Irr_mean_ad_all= mean(Irritability), mean_N= mean(BMS_N_D1),
Mean_BMS_mg_D1= mean(BMS_mean_mg_D1), Mean_BMS_mg_D2= mean(
BMS_mean_mg_D2), Mean_BMS_mg_D3= mean(BMS_mean_mg_D3))
feeding_ad_BMS_d3
feeding_ad_BMS_d3 <- as.data.frame(feeding_ad_BMS_d3) feeding_ad_BMS_d3
    Long_feeding_ad_BMS_d <- feeding_ad_BMS_d3 %>%
    pivot_longer(Mean_BMS_mg_D1:Mean_BMS_mg_D2:Mean_BMS_mg_D3, names_to =
    "BMS_mean_per_day_x", values_to = "response_values")
    print(Long_feeding_ad_BMS_d)
Long_feeding_ad_BMS_d2 <- as.data.frame(Long_feeding_ad_BMS_d)
Long_feeding_ad_BMS_d2
Day2 <- as.factor(c("Day_1", "Day_2", "Day_3", "Day_1", "Day_2", "Day_3", "Day_1",
"Day_2", "Day_3", "Day_1", "Day_2", "Day_3",
"Day_1", "Day_2", "Day_3", "Day_1", "Day_2", "Day_3", "Day_1", "Day_2", "Day_3",
"Day_1", "Day_2", "Day_3",
"Day_1", "Day_2", "Day_3", "Day_1", "Day_2", "Day_3", "Day_1", "Day_2", "Day_3",
"Day_1", "Day_2", "Day_3",
"Day_1", "Day_2", "Day_3", "Day_1", "Day_2", "Day_3", "Day_1", "Day_2", "Day_3",
"Day_1", "Day_2", "Day_3",  "Day_1", "Day_2", "Day_3")) as.matrix(Day2)
Long_feeding_ad_BMS_d3 <- cbind(Long_feeding_ad_BMS_d2, Day2)
Long_feeding_ad_BMS_d3 qplot(x = Density, y = response_values, facets = ~ Day2, data=
Long_feeding_ad_BMS_d3)
+ labs(x = "Density (stage5 nymphs/hamster)", y = "Blood meal size (BMS) ingested (mg) per day") +
geom_smooth(method = "lm")
reg1_ad_BMS_day <- lm(response_values ~ Replicate * Density, data= Long_feeding_ad_BMS_d3)
summary(reg1_ad_BMS_day)
Long_feed_ad_times_in_3_days <- feeding_ad_freq2 %>%
pivot_longer(Pct_feeding_once_in_3d:Pct_feeding_twice_in_3d:
Pct_feeding_thrice_in_3d, names_to = "Pct_feeding_x_times_in_3_days", values_to =
"response_values")
```

```
print(Long_feed_ad_times_in_3_days)
Long_feed_ad_times_in_3_days <- as.data.frame(Long_feed_ad_times_in_3_days)
Long_feed_ad_times_in_3_days
Long_feed_ad_times_in_3_days2 <- Long_feed_ad_times_in_3_days %>% group_by(Density, Replicate)
%>%
    summarise(Irr_mean_ad_all= mean(Irritability), mean_response_values=
mean(response_values))
Long_feed_ad_times_in_3_days2
Density_level <- as.factor(c("Density_10", "Density_10", "Density_10", "Density_10",
"Density_20", "Density_20", "Density_20", "Density_20", "Density_30", "Density_30",
"Density_30", "Density_30", "Density_40", "Density_40", "Density_40", "Density_40", "Density_60"))
Day2 <- as.factor(c("Day_1", "Day_2", "Day_3", "Day_1", "Day_2", "Day_3", "Day_1",
"Day_2", "Day_3", "Day_1", "Day_2", "Day_3", "Day_1", "Day_2", "Day_3", "Day_1", "Day_2"))
as.matrix(Day2)
Long_feed_ad_times_in_3_days3 <- cbind(Long_feed_ad_times_in_3_days2,Day2)
Long_feed_ad_times_in_3_days3 # Rename the "...5" column
colnames(Long_feed_ad_times_in_3_days3) <- c("Density", "Replicate",
"Irr_mean_ad_all", "mean_response_values", "Pct_feeding_x_times_in_3_days") qplot(x = Density, y =
response_values, facets = ~ Pct_feeding_x_times_in_3_days, data=
Long_feed_ad_times_in_3_days) + labs(x = "Density (adults/hamster)", y = "Percent of adults feeding in
1, 2, or 3 d ays") + geom_smooth(method = "lm")
reg1_ad_times_in_3_days <- lm(response_values ~ Replicate * Density, data=
Long_feed_ad_times_in_3_days)
summary(reg1_ad_times_in_3_days)
#######################################################
##### 2nd Look at the association between Pct_feed and BMS_feed ####
#######################################################
#########################
##### For Stage 5 nymhs #####
#########################
Pct_feed_BMS_feed2 <- as.data.frame(Pct_feed_BMS_feed2)
qplot(x = BMS_feed , y = Pct_feed, facets = ~ Density, data= Pct_feed_BMS_feed2) + labs(x =
"Average percent of stage 5 nymphs that succeded feeding per day", y = "Average blood meal size (BMS)
ingested (mg) per day", title= "Stage5 nymphs") + theme(strip.background = element_rect(fill = '571'))
+
geom_smooth(method = "lm")
qplot(x = BMS_feed , y = Pct_feed, facets= ~ round(Irritability,1), data=
Pct_feed_BMS_feed2) + labs(x = "Average percent of stage 5 nymphs that succeded feeding per day", y
= "Average blood meal size (BMS) ingested (mg) per day", title= "Stage5 nymphs") +
theme(strip.background = element_rect(fill = '575')) +
geom_smooth(method = "lm")
Pct_feed_BMS_feed2_Dens10 <- subset(Pct_feed_BMS_feed2, Pct_feed_BMS_feed2$ Density == 10)
Pct_feed_BMS_feed2_Dens10
reg1_Pct_feed_BMS_feed2_Dens10<- lm(BMS_feed ~ Pct_feed, data= Pct_feed_BMS_feed2_Dens10)
summary(reg1_Pct_feed_BMS_feed2_Dens10)
Pct_feed_BMS_feed2_Dens20 <- subset(Pct_feed_BMS_feed2, Pct_feed_BMS_feed2$ Density == 20)
Pct_feed_BMS_feed2_Dens20
reg1_Pct_feed_BMS_feed2_Dens20<- lm(BMS_feed ~ Pct_feed, data= Pct_feed_BMS_feed2_Dens20)
summary(reg1_Pct_feed_BMS_feed2_Dens20)
Pct_feed_BMS_feed2_Dens30 <- subset(Pct_feed_BMS_feed2, Pct_feed_BMS_feed2$ Density == 30)
Pct_feed_BMS_feed2_Dens30
reg1_Pct_feed_BMS_feed2_Dens30<- lm(BMS_feed ~ Pct_feed, data= Pct_feed_BMS_feed2_Dens30)
summary(reg1_Pct_feed_BMS_feed2_Dens30)
Pct_feed_BMS_feed2_Dens40 <- subset(Pct_feed_BMS_feed2, Pct_feed_BMS_feed2$ Density == 40)
Pct_feed_BMS_feed2_Dens40
reg1_Pct_feed_BMS_feed2_Dens40<- lm(BMS_feed ~ Pct_feed, data= Pct_feed_BMS_feed2_Dens40)
summary(reg1_Pct_feed_BMS_feed2_Dens40)
```

```
Pct_feed_BMS_feed2_Dens60 <- subset(Pct_feed_BMS_feed2, Pct_feed_BMS_feed2$ Density == 60)
Pct_feed_BMS_feed2_Dens60
reg1_Pct_feed_BMS_feed2_Dens60<- lm(BMS_feed ~ Pct_feed, data= Pct_feed_BMS_feed2_Dens60)
summary(reg1_Pct_feed_BMS_feed2_Dens60)


#############################################################
##### 2nd Look at the association between Pct_feed and BMS_feed ####
#############################################################
##########################
######## For Adults #########
##########################
feeding_ad_BMS_d3 <- feeding_ad_BMS_d2 %>% group_by(Density, Replicate) %>%
summarise(Irr_mean_ad_all= mean(Irritability), mean_N= mean(BMS_N_D1),
Mean_BMS_mg_D1= mean(BMS_mean_mg_D1), Mean_BMS_mg_D2= mean(
BMS_mean_mg_D2), Mean_BMS_mg_D3= mean(BMS_mean_mg_D3))
feeding_ad_BMS_d3
feeding_ad_BMS_d3 <- as.data.frame(feeding_ad_BMS_d3) feeding_ad_BMS_d3
Long_feeding_ad_BMS_d <- feeding_ad_BMS_d3 %>%
pivot_longer(Mean_BMS_mg_D1:Mean_BMS_mg_D2:Mean_BMS_mg_D3, names_to =
"BMS_mean_per_day_x", values_to = "response_values") print(Long_feeding_ad_BMS_d)
Long_feeding_ad_BMS_d2 <- as.data.frame(Long_feeding_ad_BMS_d)
Long_feeding_ad_BMS_d2
Day <- as.factor(c("Day_1", "Day_2", "Day_3", "Day_1", "Day_2", "Day_3", "Day_1",
"Day_2", "Day_3", "Day_1", "Day_2", "Day_3",
"Day_1", "Day_2", "Day_3", "Day_1", "Day_2", "Day_3", "Day_1", "Day_2", "Day_3",
"Day_1", "Day_2", "Day_3",
"Day_1", "Day_2", "Day_3", "Day_1", "Day_2", "Day_3", "Day_1", "Day_2", "Day_3",
"Day_1", "Day_2", "Day_3",
"Day_1", "Day_2", "Day_3", "Day_1", "Day_2", "Day_3", "Day_1", "Day_2", "Day_3",
"Day_1", "Day_2", "Day_3",  "Day_1", "Day_2", "Day_3")) as.matrix(Day)
Long_feeding_ad_BMS_d3 <- cbind(Long_feeding_ad_BMS_d2, Day)
Long_feeding_ad_BMS_d3
qplot(x = Density, y = response_values, facets = ~ Day, data= Long_feeding_ad_BMS_d3) + labs(x =
"Density (adults/hamster)", y = "Blood meal size (BMS) ingested
(mg/adult/day)", title= "Adults") + geom_smooth(method = "lm")
reg1_ad_BMS_day <- lm(response_values ~ Replicate * Density, data= Long_feeding_ad_BMS_d3)
summary(reg1_ad_BMS_day)
reg2_ad_BMS_day <- lm(response_values ~ Density, data= Long_feeding_ad_BMS_d3)
summary(reg2_ad_BMS_day)
###############################################
##### Analysis of the following feeding variables: #####
############ BMS_mg_NOT_Disp_D1 ############
############ BMS_mg_NOT_Disp_D2 ############
############ BMS_mg_NOT_Disp_D3 ############
############ BMS_mg_NOT_Disp_3days ###########
############################################### position <- c(1:5,19:21)
fed_NOT_moved_0 <- fed_moved[,position] fed_NOT_moved_0
# Separate Stage 5 nymphs from adults
fed_NOT_moved_n5 <- subset(fed_NOT_moved_0, fed_NOT_moved_0$Stage == 5) fed_NOT_moved_ad
<- subset(fed_NOT_moved_0, fed_NOT_moved_0$Stage == 6)
fed_NOT_moved_n5
fed_NOT_moved_ad
# Delete all the rows in the two dataframes containing any NAs
fed_NOT_moved_n5_2 <- fed_NOT_moved_n5[!is.na(fed_NOT_moved_n5$ BMS_mg_NOT_Disp_D1), ]
fed_NOT_moved_ad_2 <- fed_NOT_moved_ad[!is.na(fed_NOT_moved_ad$
BMS_mg_NOT_Disp_D1), ] fed_NOT_moved_n5_2
```

```
fed_NOT_moved_ad_2
# Create the accumulated BMS of the three days as a new variable for n5 fed_NOT_moved_n5_3 <-
fed_NOT_moved_n5_2 %>%
mutate(BMS_mg_NOT_Disp_3days = BMS_mg_NOT_Disp_D1+BMS_mg_NOT_Disp_D2
+BMS_mg_NOT_Disp_D3) fed_NOT_moved_n5_3
# Create the BMS_mg_NOT_Disp_Dx as a % of the total of the 3 days for n5 fed_NOT_moved_n5_4 <-
fed_NOT_moved_n5_3 %>%
mutate(BMS_Pct_NOT_Disp_D1= BMS_mg_NOT_Disp_D1/BMS_mg_NOT_Disp_3days* 100,
BMS_Pct_NOT_Disp_D2= BMS_mg_NOT_Disp_D2/BMS_mg_NOT_Disp_3days* 100,
BMS_Pct_NOT_Disp_D3= BMS_mg_NOT_Disp_D3/BMS_mg_NOT_Disp_3days*
100) fed_NOT_moved_n5_4
# Create the accumulated BMS of the three days as a new variable for ad fed_NOT_moved_ad_3 <-
fed_NOT_moved_ad_2 %>%
mutate(BMS_mg_NOT_Disp_3days = BMS_mg_NOT_Disp_D1+BMS_mg_NOT_Disp_D2
+BMS_mg_NOT_Disp_D3) fed_NOT_moved_ad_3
# Create the BMS_mg_NOT_Disp_Dx as a % of the total of the 3 days for ad fed_NOT_moved_ad_4 <-
fed_NOT_moved_ad_3 %>%
mutate(BMS_Pct_NOT_Disp_D1= BMS_mg_NOT_Disp_D1/BMS_mg_NOT_Disp_3days* 100,
BMS_Pct_NOT_Disp_D2= BMS_mg_NOT_Disp_D2/BMS_mg_NOT_Disp_3days* 100,
BMS_Pct_NOT_Disp_D3= BMS_mg_NOT_Disp_D3/BMS_mg_NOT_Disp_3days*
100) fed_NOT_moved_ad_4
# Delete Sex which has all NAs
fed_NOT_moved_n5_4 <- fed_NOT_moved_n5_4[,-4] fed_NOT_moved_ad_4 <-
fed_NOT_moved_ad_4[,-4] fed_NOT_moved_n5_4 fed_NOT_moved_ad_4
#################################################################
##### Inserting the Pct feeding in bugs that moved by day analysis ####
#################################################################
#############################
##### For stage 5 nymphs #####
#############################
fed_NOT_moved_n5_5 <- fed_NOT_moved_n5_4 %>%
group_by(Density, Replicate) %>%
summarise(Irr_mean_n5_all= mean(Irritability),
mean_BMS_Pct_NOT_Disp_D1= mean(BMS_Pct_NOT_Disp_D1), mean_BMS_Pct_NOT_Disp_D2=
mean(BMS_Pct_NOT_Disp_D2), mean_BMS_Pct_NOT_Disp_D3= mean(BMS_Pct_NOT_Disp_D3))
fed_NOT_moved_n5_5
feeding_n5_BMSfed_NOT_moved_n5_5 <- as.data.frame(fed_NOT_moved_n5_5)
fed_NOT_moved_n5_5
Long_fed_NOT_moved_n5_6 <- fed_NOT_moved_n5_5 %>%
pivot_longer(mean_BMS_Pct_NOT_Disp_D1:mean_BMS_Pct_NOT_Disp_D2:
mean_BMS_Pct_NOT_Disp_D3, names_to = "BMS_moved_mean_Pct_per_day_x", values_to =
"response_values")
print(Long_fed_NOT_moved_n5_6)
Long_fed_NOT_moved_n5_6 <- as.data.frame(Long_fed_NOT_moved_n5_6)
Long_fed_NOT_moved_n5_6
Day <- as.factor(c("Day_1", "Day_2", "Day_3", "Day_1", "Day_2", "Day_3", "Day_1",
"Day_2", "Day_3", "Day_1", "Day_2", "Day_3",
"Day_1", "Day_2", "Day_3", "Day_1", "Day_2", "Day_3", "Day_1", "Day_2", "Day_3",
"Day_1", "Day_2", "Day_3",
"Day_1", "Day_2", "Day_3", "Day_1", "Day_2", "Day_3", "Day_1", "Day_2", "Day_3",
"Day_1", "Day_2", "Day_3",
"Day_1", "Day_2", "Day_3", "Day_1", "Day_2", "Day_3", "Day_1", "Day_2", "Day_3", "Day_1", "Day_2",
"Day_3",  "Day_1", "Day_2", "Day_3",  "Day_1", "Day_2", "Day_3")) as.matrix(Day)
Long_fed_NOT_moved_n5_7 <- cbind(Long_fed_NOT_moved_n5_6, Day)
Long_fed_NOT_moved_n5_7
qplot(x = Density, y = response_values, facets = ~ Day, data= Long_fed_NOT_moved_n5_7
```

```
) + labs(x = "Density (stage5 nymphs/hamster)", y = "Blood meal size (BMS) each day as percent of the total\ningested in 3 days in bugs that did not move", title="Stage 5 nymphs") + geom_smooth(method = "lm")
qplot(x = Irr_mean_n5_all, y = response_values, facets = ~ Day, data= Long_fed_NOT_moved_n5_7) +
labs(x = "Irritability level of the hamster", y = "Blood meal size (BMS) each day as percent of the total\ningested in 3 days in bugs that did not move", title="Stage 5 nymphs") +
geom_smooth(method = "lm")
reg1_Long_fed_NOT_moved_n5_7 <- lm(response_values ~ Replicate * Density, data= Long_fed_NOT_moved_n5_7)
summary(reg1_Long_fed_NOT_moved_n5_7)
reg1_Long_fed_NOT_moved_n5_7 <- lm(response_values ~ Replicate, data= Long_fed_NOT_moved_n5_7)
summary(reg1_Long_fed_NOT_moved_n5_7)
############################################################
##### Inserting the Pct feeding in bugs that moved by day analysis ####
############################################################
###################
##### For adults #####
###################
fed_NOT_moved_ad_5 <- fed_NOT_moved_ad_4 %>%
group_by(Density, Replicate) %>%
summarise(Irr_mean_ad_all= mean(Irritability),
mean_BMS_Pct_NOT_Disp_D1= mean(BMS_Pct_NOT_Disp_D1), mean_BMS_Pct_NOT_Disp_D2= mean(BMS_Pct_NOT_Disp_D2), mean_BMS_Pct_NOT_Disp_D3= mean(BMS_Pct_NOT_Disp_D3))
fed_NOT_moved_ad_5
feeding_ad_BMSfed_NOT_moved_ad_5 <- as.data.frame(fed_NOT_moved_ad_5) fed_NOT_moved_ad_5
Long_fed_NOT_moved_ad_6 <- fed_NOT_moved_ad_5 %>% pivot_longer(mean_BMS_Pct_NOT_Disp_D1:mean_BMS_Pct_NOT_Disp_D2:
mean_BMS_Pct_NOT_Disp_D3, names_to = "BMS_moved_mean_Pct_per_day_x", values_to = "response_values")
print(Long_fed_NOT_moved_ad_6)
Long_fed_NOT_moved_ad_6 <- as.data.frame(Long_fed_NOT_moved_ad_6)
Long_fed_NOT_moved_ad_6
Day <- as.factor(c("Day_1", "Day_2", "Day_3", "Day_1", "Day_2", "Day_3", "Day_1",
"Day_2", "Day_3", "Day_1", "Day_2", "Day_3",
"Day_1", "Day_2", "Day_3", "Day_1", "Day_2", "Day_3", "Day_1", "Day_2", "Day_3",
"Day_1", "Day_2", "Day_3",
"Day_1", "Day_2", "Day_3", "Day_1", "Day_2", "Day_3", "Day_1", "Day_2", "Day_3",
"Day_1", "Day_2", "Day_3",
"Day_1", "Day_2", "Day_3", "Day_1", "Day_2", "Day_3", "Day_1", "Day_2", "Day_3",
"Day_1", "Day_2", "Day_3",  "Day_1", "Day_2", "Day_3")) as.matrix(Day)
Long_fed_NOT_moved_ad_7 <- cbind(Long_fed_NOT_moved_ad_6, Day)
Long_fed_NOT_moved_ad_7
qplot(x = Density, y = response_values, facets = ~ Day, data= Long_fed_NOT_moved_ad_7
) +
labs(x = "Density (adults/hamster)", y = "Blood meal size (BMS) each day as percent of the total\ningested in 3 days in bugs that did not move", title="Adults") + geom_smooth(method = "lm")
qplot(x = Irr_mean_ad_all, y = response_values, facets = ~ Day, data= Long_fed_NOT_moved_ad_7) +
labs(x = "Irritability level of the hamster", y = "Blood meal size (BMS) each day as percent of the total\ningested in 3 days in bugs that did not move", title="Adults") + geom_smooth(method = "lm")
reg1_Long_fed_NOT_moved_ad_7 <- lm(response_values ~ Replicate * Density, data= Long_fed_NOT_moved_ad_7)
summary(reg1_Long_fed_NOT_moved_ad_7)
reg1_Long_fed_NOT_moved_ad_7 <- lm(response_values ~ Density, data= Long_fed_NOT_moved_ad_7)
summary(reg1_Long_fed_NOT_moved_ad_7)
```

```
#################################################################
##### We recalculate the number of bugs feeding each day to see if there ####
##### is a relationship with BMS of bugs that did not move between boxes ####
#################################################################
N_feeding <- feeding[,1:9]
# Delete Sex which has all NAs
N_feeding <- N_feeding[!is.na(N_feeding$N_feeding_Day1), ]
N_feeding <- N_feeding[,-9]
N_feeding <- N_feeding[,-4]
N_feeding2 <- N_feeding %>% mutate(N_feeding_3_Days =
N_feeding_Day1+N_feeding_Day2+N_feeding_Day3)
N_feeding2
N_feeding2_n5 <- subset(N_feeding2, N_feeding2$Stage == 5)
N_feeding2_ad <- subset(N_feeding2, N_feeding2$Stage == 6)
N_feeding2_n5 N_feeding2_ad fed_NOT_moved_n5_6 <- fed_NOT_moved_n5_5[-17,]
fed_NOT_moved_n5_6$mean_BMS_Pct_NOT_Disp_D1
fed_NOT_moved_ad_5$mean_BMS_Pct_NOT_Disp_D1
N_feeding2_n5_2 <- N_feeding2_n5[-17,]
N_feeding2_n5_2$N_feeding_Day1 N_feeding2_ad$N_feeding_Day1
##########################
##### For Stage 5 nymhs #####
##########################
# Regression for day 1
#=================
reg_da1_n5 <- lm(fed_NOT_moved_n5_6$mean_BMS_Pct_NOT_Disp_D1 ~
N_feeding2_n5_2$N_feeding_Day1)
summary(reg_da1_n5)
# Regression for day 2
#=================
reg_da2_n5 <- lm(fed_NOT_moved_n5_6$mean_BMS_Pct_NOT_Disp_D2 ~
N_feeding2_n5_2$N_feeding_Day2)
summary(reg_da2_n5)
# Regression for day 3
#=================
reg_da3_n5 <- lm(fed_NOT_moved_n5_6$mean_BMS_Pct_NOT_Disp_D3 ~
N_feeding2_n5_2$N_feeding_Day3)
summary(reg_da3_n5)
qplot(x = N_feeding2_n5_2$N_feeding_Day1, y = fed_NOT_moved_n5_6$
mean_BMS_Pct_NOT_Disp_D1) +
labs(x = "Number of individuals feeding on day 1", y = "Number of individuals fed on day 1\nthat did not
move to another cage", title="Stage 5 nymphs") +
geom_smooth(method = "lm")
qplot(x = N_feeding2_n5_2$N_feeding_Day2, y = fed_NOT_moved_n5_6$
mean_BMS_Pct_NOT_Disp_D2) +
labs(x = "Number of individuals feeding on day 2", y = "Number of individuals fed on day 2\nthat did not
move to another cage", title="Stage 5 nymphs") +
geom_smooth(method = "lm")
qplot(x = N_feeding2_n5_2$N_feeding_Day3, y = fed_NOT_moved_n5_6$
mean_BMS_Pct_NOT_Disp_D3) +
labs(x = "Number of individuals feeding on day 3", y = "Number of individuals fed on day 3\nthat did not
move to another cage", title="Stage 5 nymphs") +
geom_smooth(method = "lm")
###################
##### For adults #####
###################
```

```
# Regression for day 1
#=================
reg_da1_ad <- lm(fed_NOT_moved_ad_5$mean_BMS_Pct_NOT_Disp_D1 ~
N_feeding2_ad$N_feeding_Day1) summary(reg_da1_ad)
# Regression for day 2
#=================
reg_da2_ad <- lm(fed_NOT_moved_ad_5$mean_BMS_Pct_NOT_Disp_D2 ~
N_feeding2_ad$N_feeding_Day2) summary(reg_da2_ad)
# Regression for day 3
#=================
reg_da3_ad <- lm(fed_NOT_moved_ad_5$mean_BMS_Pct_NOT_Disp_D3 ~
N_feeding2_ad$N_feeding_Day3) summary(reg_da3_ad)
qplot(x = N_feeding2_ad$N_feeding_Day1, y = fed_NOT_moved_ad_5$ mean_BMS_Pct_NOT_Disp_D1)
+
labs(x = "Number of individuals feeding on day 1", y = "Number of individuals fed on day 1\nthat did not
move to another cage", title="Adults") +
geom_smooth(method = "lm")
qplot(x = N_feeding2_ad$N_feeding_Day2, y = fed_NOT_moved_ad_5$ mean_BMS_Pct_NOT_Disp_D2)
+
labs(x = "Number of individuals feeding on day 2", y = "Number of individuals fed on day 2\nthat did not
move to another cage", title="Adults") +
geom_smooth(method = "lm")
qplot(x = N_feeding2_ad$N_feeding_Day3, y = fed_NOT_moved_ad_5$ mean_BMS_Pct_NOT_Disp_D3)
+
labs(x = "Number of individuals feeding on day 3", y = "Number of individuals fed on day 3\nthat did not
move to another cage", title="Adults") +
geom_smooth(method = "lm")

##############################################################
##
#### We repeat the previous analysis but with bugs that dispersed (moved) ####
##############################################################
##
############################################
##### Analysis of the following feeding variables: #####
############### BMS_mg_Disp_D1 ###############
############### BMS_mg_Disp_D2 ###############
############### BMS_mg_Disp_D3 ###############
##############              BMS_mg_Disp_3days              ############
############################################
position <- c(1:5,22:25)
fed_moved_00 <- feeding[,position]
fed_moved_00 fed_moved_00 <- fed_moved_00[19:35,]
fed_moved_00 <- fed_moved_00[,-9]
fed_moved_00
BMS_fed_moved_ad <- fed_moved_00 %>%
    mutate(Tot_BMS_mg_3Days= BMS_mg_D1+BMS_mg_D2+BMS_mg_D3)
BMS_fed_moved_ad
BMS_fed_moved_ad <- BMS_fed_moved_ad[,-4]
length(BMS_fed_moved_ad$Density)
# Separate Stage 5 nymphs from adults
position <- c(1:5,22:25)
fed_moved_00 <- feeding[,position]
BMS_fed_moved_n5 <- subset(fed_moved_00, fed_moved_00$Stage == 5)
BMS_fed_moved_n5
# Delete all the rows in the n5 dataframe containing any NAs
BMS_fed_moved_n5_2 <- BMS_fed_moved_n5[!is.na(BMS_fed_moved_n5$BMS_mg_D1
```

```
), ]
BMS_fed_moved_n5_2
BMS_fed_moved_n5_2 <- BMS_fed_moved_n5_2[,-4]
length(BMS_fed_moved_n5_2$Density)
# Create the BMS_mg_Disp_Dx as a % of the total of the 3 days for n5
BMS_fed_moved_n5_3 <- BMS_fed_moved_n5_2 %>% mutate(BMS_Pct_Disp_D1=
BMS_mg_D1/Tot_BMS_mg_3Days*100, BMS_Pct_Disp_D2
= BMS_mg_D2/Tot_BMS_mg_3Days*100, BMS_Pct_Disp_D3= BMS_mg_D3/ Tot_BMS_mg_3Days*100)
BMS_fed_moved_n5_3
# Create the BMS_mg_Disp_Dx as a % of the total of the 3 days for ad
BMS_fed_moved_ad_3 <- BMS_fed_moved_ad %>% mutate(BMS_Pct_Disp_D1=
BMS_mg_D1/Tot_BMS_mg_3Days*100, BMS_Pct_Disp_D2
= BMS_mg_D2/Tot_BMS_mg_3Days*100, BMS_Pct_Disp_D3= BMS_mg_D3/ Tot_BMS_mg_3Days*100)
BMS_fed_moved_ad_3
###########################
##### For stage 5 nymphs ####
###########################
    Long_fed_moved_n5_6 <- BMS_fed_moved_n5_3 %>%
    pivot_longer(BMS_Pct_Disp_D1:BMS_Pct_Disp_D2:BMS_Pct_Disp_D3, names_to =
    "Mean_BMS_Pct_per_day_x_moved", values_to = "response_values")
print(Long_fed_moved_n5_6)
Long_fed_moved_n5_6 <- as.data.frame(Long_fed_moved_n5_6)
Long_fed_moved_n5_6
length(Long_fed_moved_n5_6$Density)
Day <- as.factor(c("Day_1", "Day_2", "Day_3", "Day_1", "Day_2", "Day_3", "Day_1",
"Day_2", "Day_3", "Day_1", "Day_2", "Day_3",
"Day_1", "Day_2", "Day_3", "Day_1", "Day_2", "Day_3", "Day_1", "Day_2", "Day_3",
"Day_1", "Day_2", "Day_3",
"Day_1", "Day_2", "Day_3", "Day_1", "Day_2", "Day_3", "Day_1", "Day_2", "Day_3",
"Day_1", "Day_2", "Day_3",
"Day_1", "Day_2", "Day_3", "Day_1", "Day_2", "Day_3", "Day_1", "Day_2", "Day_3", "Day_1", "Day_2",
"Day_3",  "Day_1", "Day_2", "Day_3",  "Day_1", "Day_2", "Day_3")) length(Day)
Long_fed_moved_n5_7 <- cbind(Long_fed_moved_n5_6, Day)
Long_fed_moved_n5_7
qplot(x = Density, y = response_values, facets = ~ Day, data= Long_fed_moved_n5_7) + labs(x =
"Density (stage5 nymphs/hamster)", y = "Blood meal size (BMS) each day as percent of the
total\ningested in 3 days in bugs that moved to another cage", title=
"Stage 5 nymphs") + geom_smooth(method = "lm")
qplot(x = Irritability, y = response_values, facets = ~ Day, data= Long_fed_moved_n5_7) + labs(x =
"Irritability level of the hamster", y = "Blood meal size (BMS) each day as percent of the total\ningested
in 3 days in bugs that moved to another cage", title=
"Stage 5 nymphs") + geom_smooth(method = "lm") reg1_Long_fed_moved_n5_7 <-
lm(response_values ~ Replicate * Density, data= Long_fed_moved_n5_7)
summary(reg1_Long_fed_moved_n5_7)
reg1_Long_fed_moved_n5_7 <- lm(response_values ~ Density, data= Long_fed_moved_n5_7)
summary(reg1_Long_fed_moved_n5_7)
reg1_Long_fed_moved_n5_7 <- lm(response_values ~ Irritability, data= Long_fed_moved_n5_7)
summary(reg1_Long_fed_moved_n5_7)

###################
##### For Adults #####
###################
    Long_fed_moved_ad_6 <- BMS_fed_moved_ad_3 %>%
    pivot_longer(BMS_Pct_Disp_D1:BMS_Pct_Disp_D2:BMS_Pct_Disp_D3, names_to =
    "Mean_BMS_Pct_per_day_x_moved", values_to = "response_values")
print(Long_fed_moved_ad_6)
Long_fed_moved_ad_6 <- as.data.frame(Long_fed_moved_ad_6)
```

```
Long_fed_moved_ad_6
length(Long_fed_moved_ad_6$Density)
Day <- as.factor(c("Day_1", "Day_2", "Day_3", "Day_1", "Day_2", "Day_3", "Day_1",
"Day_2", "Day_3", "Day_1", "Day_2", "Day_3",
"Day_1", "Day_2", "Day_3", "Day_1", "Day_2", "Day_3", "Day_1", "Day_2", "Day_3",
"Day_1", "Day_2", "Day_3",
"Day_1", "Day_2", "Day_3", "Day_1", "Day_2", "Day_3", "Day_1", "Day_2", "Day_3",
"Day_1", "Day_2", "Day_3",
"Day_1", "Day_2", "Day_3", "Day_1", "Day_2", "Day_3", "Day_1", "Day_2", "Day_3",
"Day_1", "Day_2", "Day_3",  "Day_1", "Day_2", "Day_3"))
length(Day)
Long_fed_moved_ad_7 <- cbind(Long_fed_moved_ad_6, Day)
Long_fed_moved_ad_7
qplot(x = Density, y = response_values, facets = ~ Day, data= Long_fed_moved_ad_7) + labs(x =
"Density (adults/hamster)", y = "Blood meal size (BMS) each day as percent of the total\ningested in 3
days in bugs that moved to another cage", title="Adults") + geom_smooth(method = "lm")
qplot(x = Irritability, y = response_values, facets = ~ Day, data= Long_fed_moved_ad_7) + labs(x =
"Irritability level of the hamster", y = "Blood meal size (BMS) each day as percent of the total\ningested
in 3 days in bugs that moved to another cage", title= "Adults") + geom_smooth(method = "lm")
reg1_Long_fed_moved_ad_7 <- lm(response_values ~ Replicate * Density, data=
Long_fed_moved_ad_7)
summary(reg1_Long_fed_moved_ad_7)
reg1_Long_fed_moved_ad_7 <- lm(response_values ~ Density, data= Long_fed_moved_ad_7)
summary(reg1_Long_fed_moved_ad_7)
reg1_Long_fed_moved_ad_7 <- lm(response_values ~ Irritability, data= Long_fed_moved_ad_7)
summary(reg1_Long_fed_moved_ad_7)

#####################################################
#### Repeat the analysis using average among replicates #####
#####################################################
###########################
##### For stage 5 nymphs #####
###########################
BMS_fed_moved_n5_7 <- Long_fed_moved_n5_7 %>% group_by(Density, Day) %>%
summarise_at(vars(Irritability,response_values), funs(n(), mean, sd))
BMS_fed_moved_n5_7 <- as.data.frame(BMS_fed_moved_n5_7)
BMS_fed_moved_n5_7
names(BMS_fed_moved_n5_7)
n<-BMS_fed_moved_n5_7$response_values_n
s<-BMS_fed_moved_n5_7$response_values_sd
xmean <- BMS_fed_moved_n5_7$response_values_mean
margin <- qt(0.975,df=n-1)*s/sqrt(n)
lowerinterval <- xmean - margin upperinterval <- xmean + margin
lowerinterval
xmean
upperinterval
qplot(x = Density, y = response_values_mean, facets = ~ Day, data= BMS_fed_moved_n5_7) +
labs(x = "Density (stage5 nymphs/hamster)", y = "Blood meal size (BMS) each day as percent of the
total\ningested in 3 days in bugs that moved to another cage", title=
"Stage 5 nymphs") + geom_smooth(method = "lm") +
geom_errorbar(aes(ymin=lowerinterval, ymax=upperinterval))
qplot(x = Irritability_mean, y = response_values_mean, facets = ~ Day, data=
BMS_fed_moved_n5_7) +
labs(x = "Irritability level of the hamster", y = "Blood meal size (BMS) each day as percent of the
total\ningested in 3 days in bugs that moved to another cage", title=
"Stage 5 nymphs") + geom_smooth(method = "lm") +
geom_errorbar(aes(ymin=lowerinterval, ymax=upperinterval))
```

```
reg1_Long_fed_moved_n5_7_mean<- lm(response_values_mean ~ Density, data=
BMS_fed_moved_n5_7)
summary(reg1_Long_fed_moved_n5_7_mean)

###################
##### For Adults #####
###################
Long_fed_moved_ad_8 <- Long_fed_moved_ad_7 %>%
	group_by(Density, Day) %>%
	summarise_at(vars(Irritability,response_values), funs(n(), mean, sd))
Long_fed_moved_ad_8 <- as.data.frame(Long_fed_moved_ad_8)
Long_fed_moved_ad_8
names(Long_fed_moved_ad_8)
n<-Long_fed_moved_ad_8$response_values_n
s<-Long_fed_moved_ad_8$response_values_sd
xmean <- Long_fed_moved_ad_8$response_values_mean
margin <- qt(0.975,df=n-1)*s/sqrt(n)
lowerinterval <- xmean - margin
upperinterval <- xmean + margin
lowerinterval
xmean
upperinterval qplot(x = Density, y = response_values_mean, facets = ~ Day, data=
Long_fed_moved_ad_8) + labs(x = "Density (adults/hamster)", y = "Blood meal size (BMS) each day as
percent of the total\ningested in 3 days in bugs that moved to another cage", title="Adults") +
geom_smooth(method = "lm") +
geom_errorbar(aes(ymin=lowerinterval, ymax=upperinterval))
qplot(x = Irritability_mean, y = response_values_mean, facets = ~ Day, data=
Long_fed_moved_ad_8) +
labs(x = "Irritability level of the hamster", y = "Blood meal size (BMS) each day as percent of the
total\ningested in 3 days in bugs that moved to another cage", title=
"Adults") + geom_smooth(method = "lm") +
geom_errorbar(aes(ymin=lowerinterval, ymax=upperinterval))
reg1_Long_fed_moved_ad_8_mean<- lm(response_values_mean ~ Density, data=
Long_fed_moved_ad_8)
summary(reg1_Long_fed_moved_ad_8_mean)

################################################################
##### We recalculate the number of bugs feeding each day to see if there ####
######## is a relationship with BMS of bugs that moved between cages ######
################################################################
N_feeding <- feeding[,1:9]
# Delete Sex which has all NAs
N_feeding <- N_feeding[!is.na(N_feeding$N_feeding_Day1), ]
N_feeding <- N_feeding[,-9]
N_feeding <- N_feeding[,-4]
N_feeding2 <- N_feeding %>%
	mutate(N_feeding_3_Days = N_feeding_Day1+N_feeding_Day2+N_feeding_Day3)
N_feeding2
N_feeding2_n5 <- subset(N_feeding2, N_feeding2$Stage == 5)
N_feeding2_ad <- subset(N_feeding2, N_feeding2$Stage == 6)
N_feeding2_n5 N_feeding2_ad fed_NOT_moved_n5_6 <- fed_NOT_moved_n5_5[-17,]
fed_NOT_moved_n5_6$mean_BMS_Pct_NOT_Disp_D1
fed_NOT_moved_ad_5$mean_BMS_Pct_NOT_Disp_D1
N_feeding2_n5_2 <- N_feeding2_n5[-17,]
N_feeding2_n5_2$N_feeding_Day1
N_feeding2_ad$N_feeding_Day1
```

```
#########################
##### For Stage 5 nymhs #####
#########################
# Regression for day 1
#=================
reg_da1_n5 <- lm(fed_NOT_moved_n5_6$mean_BMS_Pct_NOT_Disp_D1 ~
N_feeding2_n5_2$N_feeding_Day1)
summary(reg_da1_n5)
# Regression for day 2
#=================
reg_da2_n5 <- lm(fed_NOT_moved_n5_6$mean_BMS_Pct_NOT_Disp_D2 ~
N_feeding2_n5_2$N_feeding_Day2)
summary(reg_da2_n5)
# Regression for day 3
#=================
reg_da3_n5 <- lm(fed_NOT_moved_n5_6$mean_BMS_Pct_NOT_Disp_D3 ~
N_feeding2_n5_2$N_feeding_Day3)
summary(reg_da3_n5)
qplot(x = N_feeding2_n5_2$N_feeding_Day1, y = fed_NOT_moved_n5_6$
mean_BMS_Pct_NOT_Disp_D1) +
labs(x = "Number of individuals feeding on day 1", y = "Number of individuals fed on day 1\nthat moved
to another cage", title="Stage 5 nymphs") +
geom_smooth(method = "lm")
qplot(x = N_feeding2_n5_2$N_feeding_Day2, y = fed_NOT_moved_n5_6$
mean_BMS_Pct_NOT_Disp_D2) +
labs(x = "Number of individuals feeding on day 2", y = "Number of individuals fed on day 2\nthat moved
to another cage", title="Stage 5 nymphs") +
geom_smooth(method = "lm")
qplot(x = N_feeding2_n5_2$N_feeding_Day3, y = fed_NOT_moved_n5_6$
mean_BMS_Pct_NOT_Disp_D3) +
labs(x = "Number of individuals feeding on day 3", y = "Number of individuals fed on day 3\nthat moved
to another cage", title="Stage 5 nymphs") + geom_smooth(method = "lm")

##################
##### For adults #####
##################
# Regression for day 1
#=================
reg_da1_ad <- lm(fed_NOT_moved_ad_5$mean_BMS_Pct_NOT_Disp_D1 ~
N_feeding2_ad$N_feeding_Day1)
summary(reg_da1_ad)
# Regression for day 2
#=================
reg_da2_ad <- lm(fed_NOT_moved_ad_5$mean_BMS_Pct_NOT_Disp_D2 ~
N_feeding2_ad$N_feeding_Day2)
summary(reg_da2_ad)
# Regression for day 3
#=================
reg_da3_ad <- lm(fed_NOT_moved_ad_5$mean_BMS_Pct_NOT_Disp_D3 ~
N_feeding2_ad$N_feeding_Day3)
summary(reg_da3_ad)
qplot(x = N_feeding2_ad$N_feeding_Day1, y = fed_NOT_moved_ad_5$ mean_BMS_Pct_NOT_Disp_D1)
+
labs(x = "Number of individuals feeding on day 1", y = "Number of individuals fed on day 1\nthat moved
to another cage", title="Adults") +
geom_smooth(method = "lm")
```

```
qplot(x = N_feeding2_ad$N_feeding_Day2, y = fed_NOT_moved_ad_5$ mean_BMS_Pct_NOT_Disp_D2)
+
labs(x = "Number of individuals feeding on day 2", y = "Number of individuals fed on day 2\nthat moved
to another cage", title="Adults") +
geom_smooth(method = "lm")
qplot(x = N_feeding2_ad$N_feeding_Day3, y = fed_NOT_moved_ad_5$ mean_BMS_Pct_NOT_Disp_D3)
+
labs(x = "Number of individuals feeding on day 3", y = "Number of individuals fed on day 3\nthat moved
to another cage", title="Adults") + geom_smooth(method = "lm")

##################################################################
########
### We repeat the analysis replacing Tot_BMS_mg_3Days by Tot_BMS_mg_3Days/3 ###
##### that is, we are interested in looking at the average BMS per day per individual ####
##################################################################
########
N_feeding2 <- N_feeding %>% mutate(N_feeding_mean_x_day =
(N_feeding_Day1+N_feeding_Day2+N_feeding_Day3)/3)
N_feeding2
N_feeding2_n5 <- subset(N_feeding2, N_feeding2$Stage == 5)
N_feeding2_ad <- subset(N_feeding2, N_feeding2$Stage == 6)
N_feeding2_n5 N_feeding2_ad fed_NOT_moved_n5_6 <- fed_NOT_moved_n5_5[-17,]
fed_NOT_moved_n5_6$mean_BMS_Pct_NOT_Disp_D1
fed_NOT_moved_ad_5$mean_BMS_Pct_NOT_Disp_D1
N_feeding2_n5_2 <- N_feeding2_n5[-17,]
N_feeding2_n5_2$N_feeding_Day1 N_feeding2_ad$N_feeding_Day1
length(N_feeding2_n5_2$N_feeding_mean_x_day) length(N_feeding2_ad$N_feeding_mean_x_day)
fed_NOT_moved_n5_6 <- fed_NOT_moved_n5_2 %>%
mutate(BMS_mean_x_day = (BMS_mg_NOT_Disp_D1+BMS_mg_NOT_Disp_D2+
BMS_mg_NOT_Disp_D3)/3) fed_NOT_moved_n5_6 <- as.data.frame(fed_NOT_moved_n5_6)
fed_NOT_moved_n5_6
fed_NOT_moved_n5_6 <- fed_NOT_moved_n5_6[-17,] fed_NOT_moved_n5_6

##########################
##### For Stage 5 nymhs #####
##########################
# Regression average N feeding and average BMS fed
#========================================
length(N_feeding2_n5_2$N_feeding_mean_x_day) length(fed_NOT_moved_n5_6$BMS_mean_x_day)
reg_da1_n5 <- lm(fed_NOT_moved_n5_6$BMS_mean_x_day ~ N_feeding2_n5_2$
N_feeding_mean_x_day)
summary(reg_da1_n5)
qplot(x = N_feeding2_n5_2$N_feeding_mean_x_day, y = fed_NOT_moved_n5_6$
BMS_mean_x_day) +
labs(x = "Average number of individuals feeding per day", y = "Average BMS (mg) ingested per day\nof
individuals that moved to another cage", title="Stage 5 nymphs")
+ geom_smooth(method = "lm")

###################
##### For adults #####
###################
fed_NOT_moved_ad_6 <- fed_NOT_moved_ad_4 %>%
mutate(BMS_ad_mean_x_day = (BMS_mg_NOT_Disp_D1+BMS_mg_NOT_Disp_D2+
BMS_mg_NOT_Disp_D3)/3) fed_NOT_moved_ad_6 <- as.data.frame(fed_NOT_moved_ad_6)
length(fed_NOT_moved_ad_6$Density)

# Regression average N feeding and average BMS fed
```

```
#=============================================
reg_da1_ad <- lm(fed_NOT_moved_ad_6$BMS_ad_mean_x_day ~
fed_NOT_moved_n5_6$BMS_mean_x_day)
summary(reg_da1_ad)
qplot(x = fed_NOT_moved_n5_6$BMS_mean_x_day, y = fed_NOT_moved_ad_6$
BMS_ad_mean_x_day) +
labs(x = "Average number of individuals feeding per day", y = "Average BMS (mg) ingested per day\nof
individuals that moved to another cage", title="Adults") + geom_smooth(method = "lm")

#########################################################
#### Look at the association between BMS_feed and Pct_feed ####
#########################################################
##########################
##### For Stage 5 nymhs #####
##########################
Pct_feed <- Long_feed_n5_times_in_3_days$response_values Pct_feed
BMS_feed <- Long_feeding_n5_BMS_d3$response_values
BMS_feed length(BMS_feed) length(Pct_feed)
Pct_feed_BMS_feed <- cbind(Pct_feed, BMS_feed)
Pct_feed_BMS_feed head(Pct_feed_BMS_feed)
Pct_feed_BMS_feed2 <- cbind(Pct_feed_BMS_feed, Long_feed_n5_times_in_3_days$
Density, Long_feed_n5_times_in_3_days$Replicate, Long_feed_n5_times_in_3_days$ Stage,
Long_feed_n5_times_in_3_days$Irritability)
colnames(Pct_feed_BMS_feed2) <- c("Pct_feed", "BMS_feed", "Density", "Replicate", "Stage",
"Irritability")
Pct_feed_BMS_feed2 <- Pct_feed_BMS_feed2[complete.cases(Pct_feed_BMS_feed2), ]
Pct_feed_BMS_feed2
reg1_BMS_feed_Pct_feed <- lm(BMS_feed ~ Pct_feed) summary(reg1_BMS_feed_Pct_feed)
plot(BMS_feed ~ Pct_feed, pch=16) abline(reg1_BMS_feed_Pct_feed)

###################
##### For Adults #####
###################
Long_feed_ad_times_in_3_days <- feeding_ad_freq2 %>%
pivot_longer(Pct_feeding_once_in_3d:Pct_feeding_twice_in_3d:
Pct_feeding_thrice_in_3d, names_to = "Pct_feeding_x_times_in_3_days", values_to =
"response_values")
print(Long_feed_ad_times_in_3_days)
Long_feed_ad_times_in_3_days <- as.data.frame(Long_feed_ad_times_in_3_days)
Long_feed_ad_times_in_3_days
Long_feed_ad_times_in_3_days2 <- Long_feed_ad_times_in_3_days %>% group_by(Density, Replicate)
%>%
     summarise(Irr_mean_ad_all= mean(Irritability), mean_response_values=
     mean(response_values))
Long_feed_ad_times_in_3_days2
Density_level <- as.factor(c("Density_10", "Density_10", "Density_10", "Density_10",
"Density_20", "Density_20", "Density_20", "Density_20",  "Density_30", "Density_30",
"Density_30", "Density_30", "Density_40", "Density_40", "Density_40",  "Density_40",  "Density_60"))
Long_feed_ad_times_in_3_days3 <- cbind(Long_feed_ad_times_in_3_days2, Density_level)
Long_feed_ad_times_in_3_days3 # Rename the "...5" column
colnames(Long_feed_ad_times_in_3_days3) <- c("Density", "Replicate",
"Irr_mean_ad_all", "mean_response_values", "Pct_feeding_x_times_in_3_days") qplot(x = Density, y =
response_values, facets = ~ Pct_feeding_x_times_in_3_days, data=
Long_feed_ad_times_in_3_days) + labs(x = "Density (adults/hamster)", y = "Percent of adults feeding in
1, 2, or 3 d ays") + geom_smooth(method = "lm")
reg1_ad_times_in_3_days <- lm(response_values ~ Replicate * Density, data=
Long_feed_ad_times_in_3_days) summary(reg1_ad_times_in_3_days)
```

```
##############################################################
##### 2nd Look at the association between Pct_feed and BMS_feed #####
##############################################################
##########################
##### For Stage 5 nymhs #####
##########################
Pct_feed_BMS_feed2 <- as.data.frame(Pct_feed_BMS_feed2)
qplot(x = BMS_feed , y = Pct_feed, facets = ~ Density, data= Pct_feed_BMS_feed2) + labs(x = "Average percent of stage 5 nymphs that succeded feeding per day", y = "Average blood meal size (BMS) ingested (mg) per day", title= "Stage5 nymphs") + theme(strip.background = element_rect(fill = '571'))
+
geom_smooth(method = "lm")
qplot(x = BMS_feed , y = Pct_feed, facets= ~ round(Irritability,1), data=
Pct_feed_BMS_feed2) + labs(x = "Average percent of stage 5 nymphs that succeded feeding per day", y = "Average blood meal size (BMS) ingested (mg) per day", title= "Stage5 nymphs") +
theme(strip.background = element_rect(fill = '575')) +
geom_smooth(method = "lm")
Pct_feed_BMS_feed2_Dens10 <- subset(Pct_feed_BMS_feed2, Pct_feed_BMS_feed2$ Density == 10)
Pct_feed_BMS_feed2_Dens10
reg1_Pct_feed_BMS_feed2_Dens10<- lm(BMS_feed ~ Pct_feed, data= Pct_feed_BMS_feed2_Dens10)
summary(reg1_Pct_feed_BMS_feed2_Dens10)
Pct_feed_BMS_feed2_Dens20 <- subset(Pct_feed_BMS_feed2, Pct_feed_BMS_feed2$ Density == 20)
Pct_feed_BMS_feed2_Dens20
reg1_Pct_feed_BMS_feed2_Dens20<- lm(BMS_feed ~ Pct_feed, data= Pct_feed_BMS_feed2_Dens20)
summary(reg1_Pct_feed_BMS_feed2_Dens20)
Pct_feed_BMS_feed2_Dens30 <- subset(Pct_feed_BMS_feed2, Pct_feed_BMS_feed2$ Density == 30)
Pct_feed_BMS_feed2_Dens30
reg1_Pct_feed_BMS_feed2_Dens30<- lm(BMS_feed ~ Pct_feed, data= Pct_feed_BMS_feed2_Dens30)
summary(reg1_Pct_feed_BMS_feed2_Dens30)
Pct_feed_BMS_feed2_Dens40 <- subset(Pct_feed_BMS_feed2, Pct_feed_BMS_feed2$ Density == 40)
Pct_feed_BMS_feed2_Dens40
reg1_Pct_feed_BMS_feed2_Dens40<- lm(BMS_feed ~ Pct_feed, data= Pct_feed_BMS_feed2_Dens40)
summary(reg1_Pct_feed_BMS_feed2_Dens40)
Pct_feed_BMS_feed2_Dens60 <- subset(Pct_feed_BMS_feed2, Pct_feed_BMS_feed2$ Density == 60)
Pct_feed_BMS_feed2_Dens60
reg1_Pct_feed_BMS_feed2_Dens60<- lm(BMS_feed ~ Pct_feed, data= Pct_feed_BMS_feed2_Dens60)
summary(reg1_Pct_feed_BMS_feed2_Dens60)

#######################################################
#################### END OF ANALYSIS ####################
#######################################################
```

SUPPLEMENTARY DATA VII

```
##############################################
####### R Computer code for beta regressions of ####### ####
Population size regulation is density-dependent #### ##### in
Rhodnius prolixus (Hemiptera: Reduviidae) #####
########## through an irritability mechanism ##########
###### by Enrique H. Weir and Jorge E. Rabinovich ######
##############################################
###############################################
# Load libraries
library(ggplot2)
library(lattice)
library(latticeExtra)
library(ggeasy)
library(dplyr)
library(tidyverse)
library(tidyr)
library(cowplot)
library(psych)
library(reshape2)
library(ggpubr)
library(rstatix)
library(AICcmodavg)
library(betareg)
###############################################
# We first read the Irritability results at the level of each day (of the 3 days of observations)
setwd("xxxxxxx") #### NOTE: The corresponding folder where the data file is located, should be inserted here
datir <- read.csv(file="Irritability as Database with Adults and Nymphs V.csv") datir_ad
<- subset(datir, datir$Stage == "A")
datir_ni <- subset(datir, datir$Stage == "V")
# Read the all the data results compiled in one file
dat <- read.csv(file="All_Data_One_File2.csv") names(dat)
# Select the variables of the data set into five categories:
# (1) Feeding, (2) Mortality, (3) Reproduction, (4) Molting/longevity, and (5) Dispersal # For some questions of interest there are some "combined" or "mixed" variables # combining three single
```

```
variables: mortality, feeding and movement:
# (a) mort_in_bugs_that_moved
# (b) mort_in_bugs_that_fed
# (c) fed_bugs_that _moved
# Only feeding
position <- c(1:5, 47:53, 74:86, 91:103)
feeding <- dat[,position] names(feeding)
# Only mortality
position <- c(1:5,
6:14) mortality <-
dat[,position]
names(mortality)
Prop_dead_by_day <- mortality[,10:12]
# Only molting_longevity position <-
c(1:5, 33:34, 54:56, 103:118)
molting_longevity <- dat[,position]
names(molting_longevity)
# Only dispersal
position <- c(1:5,
35:38) dispersal <-
dat[,position]
names(dispersal)
# (a) mort_moved (mort_in_bugs_that_moved)
position <- c(1:5, 15:23)
mort_moved <- dat[,position]
names(mort_moved)
# (b) mort_fed (mort_in_bugs_that_fed)
position <- c(1:5, 24:32) mort_fed <-
dat[,position] names(mort_fed)
# (c) mort_molt (mort_in_bugs_that_molted)
position <- c(1:5, 112,115) mort_molt <-
dat[,position] names(mort_molt)
# (d) fed_moved (fed_bugs_that _moved)
position <- c(1:5, 39:46, 83:90) fed_moved
<- dat[,position] names(fed_moved)
# (d) moved (N_bugs_that _moved)
position <- c(1:5, 35:38)
All_moved <- dat[,position]
```

```
names(All_moved)
#############################################
##### Percent mortality in the three-day trials #####
#############################################
###################################
#####          Beta          regressions          only          for          N5          ####
###################################
names(molting_longevity)
Fema_moved <- subset(dispersal, dispersal$Stage == 6 & dispersal$Sex == 1)
Male_moved <- subset(dispersal, dispersal$Stage == 6 & dispersal$Sex == 2)
Fema_moved
Male_moved
Fema_moved <- Fema_moved[1:17,] Fema_moved
Fema_moved_2 <- Fema_moved %>%
   mutate(prop_moved_day1    =    N_moved_Day1    /Density,
   prop_moved_day2          =          N_moved_Day2/Density,
   prop_moved_day3 = N_moved_Day3/Density,
   prop_moved_3days = prop_moved_day1*prop_moved_day2*prop_moved_day3)
Fema_moved_2
Fema_moved_3 <- Fema_moved_2[,-(6:9)] Fema_moved_3
Fema_moved_4  <-  Fema_moved_3  %>%  group_by(Density)
   %>%
   summarise_at(vars(prop_moved_day1,prop_moved_day2,prop_moved_day3,     prop_moved_3days),
   funs(mean))
Fema_moved_4          <-          as.data.frame(Fema_moved_4)
Fema_moved_4
Male_moved_2            <-            Male_moved            %>%
   mutate(prop_moved_day1    =    N_moved_Day1    /Density,
   prop_moved_day2          =          N_moved_Day2/Density,
   prop_moved_day3 = N_moved_Day3/Density,
   prop_moved_3days = prop_moved_day1*prop_moved_day2*prop_moved_day3)
Male_moved_2
Male_moved_3 <- Male_moved_2[,-(6:9)] Male_moved_3
Male_moved_4  <-  Male_moved_3  %>%  group_by(Density)
   %>%
   summarise_at(vars(prop_moved_day1,prop_moved_day2,prop_moved_day3,     prop_moved_3days),
   funs(mean))
Male_moved_4 <- as.data.frame(Male_moved_4)
```

```
Male_moved_4
############################################################
##### Create a matrix to hold the various beta regression results #####
############################################################
beta_res <- data.frame(Intcpt = rep(0,8), Density = rep(0,8),Phi = rep(0,8), LL = rep(0,8),
DoF = rep(0,8), Ps_R2 = rep(0,8), p_val_Dens = rep(0,8))
str(beta_res)
for (i in
  1:8) {
  if(i ==
  1) {
#################################################
##### Proportion Females that moved on Day 1 ####
#################################################
beta_reg_prop_moved_Day1 <- betareg(prop_moved_day1 ~ Density, data = Fema_moved_4)
summary(beta_reg_prop_moved_Day1)
#&&&&&&&&&&&&&&&&&&&&&&&&&&&&&&&&&&&&&&&&&&&&&&&&
#################################################################
##
##### Extract from the beta regression results the parameters of interest #####
#################################################################
##
thereg <- beta_reg_prop_moved_Day1
# For the Intercept
INT <- thereg[[1]]$mean[1]
names(INT) <-NULL
INT
# For the regressor (Density)
REG <- thereg[[1]]$mean[2]
names(REG) <-NULL
REG
# For the precision (Phi)
PHI <- thereg[[1]]$precision[1]
names(PHI) <- NULL
PHI
# The predicted values (mu)
PRED <- thereg[[3]]
names(PRED) <- NULL
```

```
PRED
# Maximum-likelihood
LL <- thereg[[5]]$value
LL
# Degrees of freedom
DOF <- thereg[[14]]
DOF
# Pseudo R-squared
R_2 <- thereg[[20]]
R_2
# p-value of the regressor "Density"
p_val_Dens     <-     summary(thereg)$coefficients$mean[2,4]
p_val_Dens
#&&&&&&&&&&&&&&&&&&&&&&&&&&&&&&&&&&&&&&&&&&&&&&&&&&
###################################################################
#####
##### Calculate the predicted values using the expected proportion E(y)=μ that #####
#### can be computed by applying the inverse link function to the linear predictor ###
#### Similarly, we can also compute the variance to graph the confidence intervals ###
###################################################################
#####
mu <- PRED mu
phi  <-
PHI
phi
# Prediction of the variance of the predicted proportion fed
Var_y <- mu * (1 - mu)/(1+phi)
Var_y
sd_y <- sqrt(Var_y)
sd_y
plot(prop_moved_d
ay1 ~ Density, data
=   Fema_moved_4,
pch=16,          ylim
=c(0,0.3), ylab= "
Mean proportion of females that moved on day 1", xlab= " Density (number of adults/hamster)")
lines(mu ~ Density, data = Fema_moved_4)
# Confidence Interval = x+tn-1, 1-α/2*(s/√n) n
```

```
<- 5
s <- sd_y
xmean <- mu
margin <- qt(0.975,df=n-1)*s/sqrt(n)
lowerinterval <- xmean - margin upperinterval
<- xmean + margin
for (j in 1:n) if(lowerinterval[j] <0) lowerinterval[j] <- 0.01
lowerinterval xmean
upperinterval
plot.new() plot(prop_moved_day1 ~ Density, data = Fema_moved_4, pch=16, ylim =c(0,0.3), col= "
red", ylab= "Mean proportion of females that moved on day 1", xlab= " Density (number of
adults/hamster)") # Beta regression lines(mu ~ Density, data = Fema_moved_4, col= "blue", lwd= 3)
arrows(x0=Fema_moved_4$Density, y0=xmean - margin, x1=Fema_moved_4$Density, y1=xmean +
margin, angle=90, code=3, length=0.05, lwd= 1, lty= 1, col= "blue")
# NOTE: The confidence interval lines are the ones corresponding to the predictions of the beta regression
# We check if the variance of the Beta regression is a function of density of bugs
Dens_Var <- cbind(Fema_moved_4$Density, Var_y)
Dens_Var
pp <- lm(Var_y ~ Fema_moved_4$Density) summary(pp)
plot(Var_y ~ Fema_moved_4$Density, pch=16) abline(pp)
##############################################################
########
### The bug density has a significant effect on the variance in the results of Day 1 ###
#################################################################
#####
beta_res[i,1] <- INT
beta_res[i,2] <- REG
beta_res[i,3] <- PHI
beta_res[i,4] <- LL
beta_res[i,5] <- DOF
beta_res[i,6] <- R_2
beta_res[i,7] <- p_val_Dens
} # End of the "if" for i=1 (Fem/Day1)
if (i == 2) {
#################################################
##### Proportion Stage 5 nymphs that moved on day 2 #####
#################################################
beta_reg_prop_moved_Day2 <- betareg(prop_moved_day2 ~ Density, data = Fema_moved_4)
```

```
summary(beta_reg_prop_moved_Day2)
#&&&&&&&&&&&&&&&&&&&&&&&&&&&&&&&&&&&&&&&&&&&&&&&&&
#################################################################
#####   Extract  from  the  beta  regression  results  the  parameters  of  interest  #####
################################################################
#
thereg <- beta_reg_prop_moved_Day2
# For the Intercept
INT      <-      thereg[[1]]$mean[1]
names(INT) <-NULL
INT
#  For  the  regressor  (Density)
REG  <-  thereg[[1]]$mean[2]
names(REG) <-NULL
REG
# For the precision  (Phi)
PHI <- thereg[[1]]$precision[1]
names(PHI) <- NULL
PHI
#  The  predicted  values  (mu)
PRED       <-       thereg[[3]]
names(PRED) <- NULL
PRED
# Maximum-likelihood
LL <- thereg[[5]]$value
LL
# Degrees of freedom
DOF <- thereg[[14]]
DOF
# Pseudo R-squared
R_2 <- thereg[[20]]
R_2
# p-value of the regressor "Density"
p_val_Dens    <-    summary(thereg)$coefficients$mean[2,4]
p_val_Dens
#&&&&&&&&&&&&&&&&&&&&&&&&&&&&&&&&&&&&&&&&&&&&&&&&&&
#################################################################
#####
```

```
### Calculate the predicted values using the expected proportion E(y)=μ that #######
#### can be computed by applying the inverse link function to the linear predictor ###
##### Similarly, we can also compute the variance to graph the confidence intervals ##
#################################################################
######
mu      <-
PRED mu
phi  <-
PHI
phi
# Prediction of the variance of the predicted proportion fed
Var_y <- mu * (1 - mu)/(1+phi)
Var_y
sd_y <- sqrt(Var_y)
plot(prop_moved_day2 ~ Density, data = Fema_moved_4, pch=16, ylim =c(0,0.3), ylab= "
Mean proportion of females that moved on day 2", xlab= " Density (number of adults/hamster)")
lines(mu ~ Density, data = Fema_moved_4)
# Confidence Interval = x+tn-1, 1-α/2*(s/√n) n
<- 5
s <- sd_y
xmean <- mu
margin      <-      qt(0.975,df=n-1)*s/sqrt(n)
lowerinterval <- xmean - margin upperinterval
<- xmean + margin
for (j in 1:n) if(lowerinterval[j] <0) lowerinterval[j] <- 0.01
lowerinterval xmean
upperinterval
plot.new() plot(prop_moved_day2 ~ Density, data = Fema_moved_4, pch=16, ylim =c(0,0.2), col= "
red", ylab= "Mean proportion of females that moved on day 2", xlab= " Density (number of
adults/hamster)") # Beta regression lines(mu ~ Density, data = Fema_moved_4, col= "blue", lwd= 2)
arrows(x0=Fema_moved_4$Density, y0=xmean - margin, x1=Fema_moved_4$Density, y1=xmean +
margin,
      angle=90, code=3, length=0.05, lwd= 1, lty= 1, col= "blue")
# NOTE: The confidence interval lines are the ones corresponding to the predictions of the beta regression
# We check if the variance of the Beta regression is a function of density of bugs
Dens_Var <- cbind(Fema_moved_4$Density, Var_y)
Dens_Var
pp <- lm(Var_y ~ Fema_moved_4$Density) summary(pp)
```

```
plot(Var_y ~ Fema_moved_4$Density, pch=16) abline(pp)
#################################################################
#######
#### The bug density has a significant effect on the variance in the results of Day 2 ####
#################################################################
#######
beta_res[i,1]     <-     INT
beta_res[i,2]     <-     REG
beta_res[i,3]     <-     PHI
beta_res[i,4]     <-     LL
beta_res[i,5]     <-     DOF
beta_res[i,6]     <-     R_2
beta_res[i,7] <- p_val_Dens
} # End of the "if" for i=2 (Fem/Day2)
#$$$$$$$$$$$$$$$$$$$$$$$$$$$$$$$$$$$$$$$$$$$$$$$$$$$$$$$$$$$$$$$$$$$$
if (i
==
3)
{
##################################################
##### Proportion females that moved on day 3 #####
##################################################
beta_reg_prop_moved_Day3 <- betareg(prop_moved_day3 ~ Density, data = Fema_moved_4)
summary(beta_reg_prop_moved_Day3)
#&&&&&&&&&&&&&&&&&&&&&&&&&&&&&&&&&&&&&&&&&&&&&&&&
#################################################################
##
#####   Extract   from   the   beta   regression   results   the   parameters   of   interest   #####
################################################################
### thereg <- beta_reg_prop_moved_Day3
# For the Intercept
INT     <-     thereg[[1]]$mean[1]
names(INT) <-NULL
INT
#  For  the  regressor  (Density)
REG  <-  thereg[[1]]$mean[2]
names(REG) <-NULL
REG
```

```
# For the precision  (Phi)
PHI <- thereg[[1]]$precision[1]
names(PHI) <- NULL
PHI
#   The   predicted   values   (mu)
PRED          <-          thereg[[3]]
names(PRED) <- NULL
PRED
# Maximum-likelihood
LL <- thereg[[5]]$value
LL
# Degrees of freedom
DOF <- thereg[[14]]
DOF
# Pseudo R-squared
R_2 <- thereg[[20]]
R_2
# p-value of the regressor "Density"
p_val_Dens      <-      summary(thereg)$coefficients$mean[2,4]
p_val_Dens
#&&&&&&&&&&&&&&&&&&&&&&&&&&&&&&&&&&&&&&&&&&&&&&&&&&&&&&&



#################################################################
######
##### Calculate the predicted values using the expected proportion E(y)=μ that ######
#### can be computed by applying the inverse link function to the linear predictor ####
### Similarly, we can also compute the variance to graph the confidence intervals ####
#################################################################
######
mu       <-
PRED mu
phi   <-
PHI
phi
plot(prop_moved_day3 ~ Density, data = Fema_moved_4, pch=16, ylim =c(0,0.3), ylab= "
Mean proportion of females that moved on day 3", xlab= " Density (number of adults/hamster)")
```

```
lines(mu ~ Density, data = Fema_moved_4)
# Confidence Interval = x+tn-1, 1-α/2*(s/√n) n
<- 5
s <- sd_y
xmean <- mu
margin <- qt(0.975,df=n-1)*s/sqrt(n)
lowerinterval <- xmean - margin upperinterval
<- xmean + margin
for (j in 1:n) if(lowerinterval[j] <0) lowerinterval[j] <- 0.01
lowerinterval xmean
upperinterval
plot.new() plot(prop_moved_day3 ~ Density, data = Fema_moved_4, pch=16, ylim =c(0,0.3),
col=
"red", ylab= "Mean proportion of females that moved on day 3", xlab= " Density
(number of adults/hamster)") lines(mu ~ Density, data =
Fema_moved_4, col= "blue", lwd= 2)
arrows(x0=Fema_moved_4$Density, y0=xmean - margin,
x1=Fema_moved_4$Density, y1=xmean + margin, angle=90,
code=3, length=0.05, lwd= 1, lty= 1, col= "blue")
# We check if the variance of the Beta regression is a function of density of bugs
Dens_Var <- cbind(Fema_moved_4$Density, Var_y)
Dens_Var
pp <- lm(Var_y ~ Fema_moved_4$Density) summary(pp)
plot(Var_y ~ Fema_moved_4$Density, pch=16) abline(pp)
beta_res[i,1] <- INT
beta_res[i,2] <- REG
beta_res[i,3] <- PHI
beta_res[i,4] <- LL
beta_res[i,5] <- DOF
beta_res[i,6] <- R_2
beta_res[i,7] <- p_val_Dens
} # End of the "if" for i=3 (Fem/Day3)
#$$$$$$$$$$$$$$$$$$$$$$$$$$$$$$$$$$$$$$$$$$$$$$$$$$$$$$$$$$$$$$$$$$$$$$$$$$$$$$$$
$$$
if (i
==
4)
{
```

```
############################################
#####     Proportion     females     that     moved     in     3Days     #####
############################################
beta_reg_prop_moved_3days <- betareg(prop_moved_3days ~ Density, data = Fema_moved_4)
summary(beta_reg_prop_moved_3days)
#&&&&&&&&&&&&&&&&&&&&&&&&&&&&&&&&&&&&&&&&&&&&&



################################################################
##
#####  Extract  from  the  beta  regression  results  the  parameters  of  interest  #####
###############################################################
### thereg <- beta_reg_prop_moved_3days
# For the Intercept
INT      <-      thereg[[1]]$mean[1]
names(INT) <-NULL
INT
#  For  the  regressor  (Density)
REG  <-  thereg[[1]]$mean[2]
names(REG) <-NULL
REG
# For the precision  (Phi)
PHI <- thereg[[1]]$precision[1]
names(PHI) <- NULL
PHI
#  The  predicted  values  (mu)
PRED      <-      thereg[[3]]
names(PRED) <- NULL
PRED
# Maximum-likelihood
LL <- thereg[[5]]$value
LL
# Degrees of freedom
DOF <- thereg[[14]]
DOF
# Pseudo R-squared
R_2 <- thereg[[20]]
```

```
R_2
# p-value of the regressor "Density"
p_val_Dens     <-     summary(thereg)$coefficients$mean[2,4]
p_val_Dens
#&&&&&&&&&&&&&&&&&&&&&&&&&&&&&&&&&&&&&&&&&&&&&&
###################################################################
######
##### Calculate the predicted values using the expected proportion E(y)=μ that ######
#### can be computed by applying the inverse link function to the linear predictor ####
#### Similarly, we can also compute the variance to graph the confidence intervals ####
###################################################################
#######
mu      <-
PRED mu
phi  <-
PHI
phi
plot(prop_moved_3days ~ Density, data = Fema_moved_4, pch=16, ylim =c(0,0.01), ylab=
" Mean proportion of females that moved in 3 days", xlab= " Density (number of adults/hamster)")
lines(mu ~ Density, data = Fema_moved_4)
# Confidence Interval = x+tn-1, 1-α/2*(s/√n) n
<- 5
s <- sd_y
xmean <- mu
margin      <-      qt(0.975,df=n-1)*s/sqrt(n)
lowerinterval  <-  xmean  -  margin  upperinterval
<- xmean + margin
for  (j  in  1:n)  if(lowerinterval[j]  <0)  lowerinterval[j]  <-  0.01
lowerinterval
xmean
upperinterval
plot.new() plot(prop_moved_3days ~ Density, data = Fema_moved_4, pch=16, ylim =c(0,0.05), col=
"red",   ylab=  "Mean  proportion  of  females  that  moved  in  3  days",  xlab=  "  Density  (number  of
adults/hamster)")
#  Beta  regression  lines(mu  ~  Density,  data  =  Fema_moved_4,  col=
"blue",  lwd=  2)  arrows(x0=Fema_moved_4$Density,  y0=xmean  -
margin, x1=Fema_moved_4$Density, y1=xmean + margin, angle=90,
code=3, length=0.05, lwd= 1, lty= 1, col= "blue")
```

```
# NOTE: The confidence interval lines are the ones corresponding to the predictions of the beta regression
# We check if the variance of the Beta regression is a function of density of bugs
Dens_Var <- cbind(Fema_moved_4$Density, Var_y)
Dens_Var
pp <- lm(Var_y ~ Fema_moved_4$Density) summary(pp)
plot(Var_y ~ Fema_moved_4$Density, pch=16) abline(pp)
beta_res[i,1]      <-      INT
beta_res[i,2]      <-      REG
beta_res[i,3]      <-      PHI
beta_res[i,4]      <-      LL
beta_res[i,5]      <-      DOF
beta_res[i,6]      <-      R_2
beta_res[i,7] <- p_val_Dens
} # End of the "if" for i=4 (Fem/3Days)
#$$$$$$$$$$$$$$$$$$$$$$$$$$$$$$$$$$$$$$$$$$$$$$$$$$$$$$$$$$$$$$$$$$$$$$$$$
####################################
##### Start with the analysis of Males ######
##############################
###### if (i == 5) {
#########################################
######## Beta regressions only for Adults ########
##### Proportion of adults that moved on day 1 #####
#########################################
beta_reg_prop_moved_males_day1 <- betareg(prop_moved_day1 ~ Density, data = Male_moved_4)
summary(beta_reg_prop_moved_males_day1)
##############################################################
##
##### Extract from the beta regression results the parameters of interest #####
#############################################################
### thereg <- beta_reg_prop_moved_males_day1
# For the Intercept
INT      <-      thereg[[1]]$mean[1]
names(INT) <-NULL
INT
#  For  the  regressor  (Density)
REG  <-  thereg[[1]]$mean[2]
names(REG) <-NULL
REG
```

```
# For the precision (Phi) PHI
<- thereg[[1]]$precision[1]
names(PHI) <- NULL
PHI
# The predicted values (mu)
PRED <- thereg[[3]]
names(PRED) <- NULL
PRED
# Maximum-likelihood
LL <- thereg[[5]]$value
LL
# Degrees of freedom
DOF <- thereg[[14]]
DOF
# Pseudo R-squared
R_2 <- thereg[[20]]
R_2
# p-value of the regressor "Density"
p_val_Dens <- summary(thereg)$coefficients$mean[2,4]
p_val_Dens
#&&&&&&&&&&&&&&&&&&&&&&&&&&&&&&&&&&&&&&&&&&&&&&&&
mu <-
PRED mu
phi <-
PHI
phi
# Prediction of the variance of the predicted proportion fed
Var_y <- mu * (1 - mu)/(1+phi)
Var_y
sd_y <- sqrt(Var_y)
sd_y
plot(prop_moved_day1 ~ Density, data = Male_moved_4, pch=16, ylim =c(0,0.4), ylab= "
Mean proportion of males that moved on day 1", xlab= " Density (number of adults/hamster)")
lines(mu ~ Density, data = Fema_moved_4)
# Confidence Interval = x+tn-1, 1-α/2*(s/√n) n
<- 5
s <- sd_y
xmean <- mu
```

```
margin <- qt(0.975,df=n-1)*s/sqrt(n)
lowerinterval <- xmean - margin upperinterval
<- xmean + margin
for (j in 1:n) if(lowerinterval[j] <0) lowerinterval[j] <- 0.01
lowerinterval
xmean
upperinterval
plot.new() plot(prop_moved_day1 ~ Density, data = Male_moved_4, pch=16, ylim =c(0,0.4), col=
"red", ylab= "Mean proportion of males that moved on day 1", xlab= " Density (number of
adults/hamster)") # Beta regression lines(mu ~ Density, data = Male_moved_4, col= "blue", lwd= 2)
arrows(x0=Male_moved_4$Density, y0=xmean - margin, x1=Male_moved_4$Density, y1=xmean +
margin, angle=90, code=3, length=0.05, lwd= 1, lty= 1, col= "blue")
# NOTE: The confidence interval lines are the ones corresponding to the predictions of the beta regression
# We check if the variance of the Beta regression is a function of density of bugs
Dens_Var <- cbind(Male_moved_4$Density, Var_y)
Dens_Var
pp <- lm(Var_y ~ Male_moved_4$Density) summary(pp)
plot(Var_y ~ Male_moved_4$Density, pch=16) abline(pp)
beta_res[i,1] <- INT
beta_res[i,2] <- REG
beta_res[i,3] <- PHI
beta_res[i,4] <- LL
beta_res[i,5] <- DOF
beta_res[i,6] <- R_2
beta_res[i,7] <- p_val_Dens
} # End of the "if" for i=5 (Males/Day1)
#$$$$$$$$$$$$$$$$$$$$$$$$$$$$$$$$$$$$$$$$$$$$$$$$$$$$$$$$$$$$$$$$$$
if (i == 6) {
########################################
##### Proportion Adults that moved on day 2 ####
########################################
beta_reg_prop_moved_males_day2 <- betareg(prop_moved_day2 ~ Density, data = Male_moved_4)
summary(beta_reg_prop_moved_males_day2)
#&&&&&&&&&&&&&&&&&&&&&&&&&&&&&&&&&&&&&&&&&&&&&&&&&&&&&&

##############################################################
##
###### Extract from the beta regression results the parameters of interest ######
```

```
##################################################################
##
thereg <- beta_reg_prop_moved_males_day2
# For the Intercept
INT <- thereg[[1]]$mean[1]
names(INT) <-NULL
INT
# For the regressor (Density)
REG <- thereg[[1]]$mean[2]
names(REG) <-NULL
REG
# For the precision (Phi)
PHI <- thereg[[1]]$precision[1]
names(PHI) <- NULL
PHI
# The predicted values (mu)
PRED <- thereg[[3]]
names(PRED) <- NULL
PRED
# Maximum-likelihood
LL <- thereg[[5]]$value
LL
# Degrees of freedom
DOF <- thereg[[14]]
DOF
# Pseudo R-squared
R_2 <- thereg[[20]]
R_2
# p-value of the regressor "Density"
p_val_Dens <- summary(thereg)$coefficients$mean[2,4]
p_val_Dens
#&&&&&&&&&&&&&&&&&&&&&&&&&&&&&&&&&&&&&&&&&&&&&&&&&&&&&&&&&&&
##################################################################
######
##### Calculate the predicted values using the expected proportion E(y)=μ that ######
#### can be computed by applying the inverse link function to the linear predictor ####
#### Similarly, we can also compute the variance to graph the confidence intervals ####
##################################################################
```

```
#######
mu
<-
PRED
mu
phi  <-
PHI
phi
# Prediction of the variance of the predicted proportion fed
Var_y <- mu * (1 - mu)/(1+phi)
Var_y
sd_y <- sqrt(Var_y)
sd_y
plot(prop_moved_day2 ~ Density, data = Male_moved_4, pch=16, ylim =c(0,0.3), ylab= " Mean
proportion of males that moved on day 2", xlab= " Density (number of
Adults/hamster)")
lines(mu ~ Density, data = Male_moved_4)
# Confidence Interval = x+tn-1, 1-α/2*(s/√n) n
<- 5
s <- sd_y
xmean <- mu
margin      <-      qt(0.975,df=n-1)*s/sqrt(n)
lowerinterval <- xmean - margin upperinterval
<- xmean + margin
for (j in 1:n) if(lowerinterval[j] <0) lowerinterval[j] <- 0.01
lowerinterval xmean
upperinterval
plot.new() plot(prop_moved_day2 ~ Density, data = Male_moved_4, pch=16, ylim =c(0,0.2), col= "
red", ylab= "Mean proportion of males that moved on day 2", xlab= " Density (number of
Adults/hamster)") # Beta regression lines(mu ~ Density, data = Male_moved_4, col= "blue", lwd= 3)
arrows(x0=Male_moved_4$Density, y0=xmean - margin, x1=Male_moved_4$Density, y1=xmean +
margin, angle=90, code=3, length=0.05, lwd= 1, lty= 1, col= "blue")
# NOTE: The confidence interval lines are the ones corresponding to the predictions of the beta regression
# We check if the variance of the Beta regression is a function of density of bugs
Dens_Var <- cbind(Male_moved_4$Density, Var_y)
Dens_Var
pp <- lm(Var_y ~ Male_moved_4$Density) summary(pp)
plot(Var_y ~ Male_moved_4$Density, pch=16) abline(pp)
```

```
###################################################################
######
#### The bug density has a significant effect on the variance in the results of Day 1 ###
###################################################################
######
beta_res[i,1]      <-      INT
beta_res[i,2]      <-      REG
beta_res[i,3]      <-      PHI
beta_res[i,4]      <-      LL
beta_res[i,5]      <-      DOF
beta_res[i,6]      <-      R_2
beta_res[i,7] <- p_val_Dens
} # End of the "if" for i=6 (Males/Day2)
if (i == 7) {
##########################################
#####      Proportion      Males      that      moved      on      day      3      ######
##########################################
beta_reg_prop_moved_males_day3 <- betareg(prop_moved_day3 ~ Density, data = Male_moved_4)
summary(beta_reg_prop_moved_males_day3)
#&&&&&&&&&&&&&&&&&&&&&&&&&&&&&&&&&&&&&&&&&&&&&&&&&&&&
###################################################################
##
###### Extract from the beta regression results the parameters of interest ######
##################################################################
### thereg <- beta_reg_prop_moved_males_day3
# For the Intercept
INT      <-      thereg[[1]]$mean[1]
names(INT) <-NULL
INT
# For the regressor (Density)
REG  <-  thereg[[1]]$mean[2]
names(REG) <-NULL
REG
# For the precision  (Phi)
PHI <- thereg[[1]]$precision[1]
names(PHI) <- NULL
PHI
# The predicted values (mu)
```

```
PRED       <-       thereg[[3]]
names(PRED) <- NULL
PRED
# Maximum-likelihood
LL <- thereg[[5]]$value
LL
# Degrees of freedom
DOF <- thereg[[14]]
DOF
# Pseudo R-squared
R_2 <- thereg[[20]]
R_2
# p-value of the regressor "Density"
p_val_Dens    <-    summary(thereg)$coefficients$mean[2,4]
p_val_Dens
#&&&&&&&&&&&&&&&&&&&&&&&&&&&&&&&&&&&&&&&&&&&&&&&&&&&&
###############################################################
######
#### Calculate the predicted values using the expected proportion E(y)=μ that ######
#### can be computed by applying the inverse link function to the linear predictor ###
#### Similarly we can also compute the variance to graph the confidence intervals ####
###############################################################
#######
mu     <-
PRED mu
phi  <-
PHI
phi
# Prediction of the variance of the predicted proportion fed
Var_y <- mu * (1 - mu)/(1+phi)
Var_y
sd_y <- sqrt(Var_y)
plot(prop_moved_day3 ~ Density, data = Male_moved_4, pch=16, ylim =c(0,0.3), ylab= " Mean
proportion of males that moved on day 3", xlab= " Density (number of
Adults/hamster)")
lines(mu ~ Density, data = Male_moved_4)
# Confidence Interval = x+tn-1, 1-α/2*(s/√n) n
<- 5
```

```
s <- sd_y
xmean <- mu
margin     <-     qt(0.975,df=n-1)*s/sqrt(n)
lowerinterval <- xmean - margin upperinterval
<- xmean + margin
for (j in 1:n) if(lowerinterval[j] <0) lowerinterval[j] <- 0.01
lowerinterval xmean
upperinterval
plot.new() plot(prop_moved_day3 ~ Density, data = Male_moved_4, pch=16, ylim =c(0,0.3), col= "
red", ylab= "Mean proportion of males that moved on day 3", xlab= " Density (number of
Adults/hamster)") lines(mu ~ Density, data = Male_moved_4, col= "blue", lwd= 2)
arrows(x0=Male_moved_4$Density, y0=xmean - margin, x1=Male_moved_4$Density, y1=xmean +
margin, angle=90, code=3, length=0.05, lwd= 1, lty= 1, col= "blue")
# We check if the variance of the Beta regression is a function of density of bugs
Dens_Var <- cbind(Male_moved_4$Density, Var_y) Dens_Var
pp <- lm(Var_y ~ Male_moved_4$Density) summary(pp)
plot(Var_y ~ Male_moved_4$Density, pch=16) abline(pp)
beta_res[i,1]     <-     INT
beta_res[i,2]     <-     REG
beta_res[i,3]     <-     PHI
beta_res[i,4]     <-     LL
beta_res[i,5]     <-     DOF
beta_res[i,6]     <-     R_2
beta_res[i,7] <- p_val_Dens
} # End of the "if" for i=7 (AD/Day3)
#$$$$$$$$$$$$$$$$$$$$$$$$$$$$$$$$$$$$$$$$$$$$$$$$$$$$$$$$$$$$$$$$$$$$$$$
if (i == 8) {
##############################################
######### Proportion Adults that moved in 3 days #######
##############################################
beta_reg_prop_moved_males_3days <- betareg(prop_moved_3days ~ Density, data = Male_moved_4)
summary(beta_reg_prop_moved_males_3days)
#&&&&&&&&&&&&&&&&&&&&&&&&&&&&&&&&&&&&&&&&&&&&&&&&

############################################################
##
#####  Extract from the beta regression results the parameters of interest  #####
############################################################
```

```
### thereg <- beta_reg_prop_moved_males_3days
# For the Intercept
INT <- thereg[[1]]$mean[1]
names(INT) <-NULL
INT
#  For  the  regressor  (Density)
REG  <-  thereg[[1]]$mean[2]
names(REG) <-NULL
REG
# For the precision  (Phi)
PHI <- thereg[[1]]$precision[1]
names(PHI) <- NULL
PHI
#  The  predicted  values  (mu)
PRED        <-        thereg[[3]]
names(PRED) <- NULL
PRED
# Maximum-likelihood
LL <- thereg[[5]]$value
LL
# Degrees of freedom
DOF <- thereg[[14]]
DOF
# Pseudo R-squared
R_2 <- thereg[[20]]
R_2
# p-value of the regressor "Density"
p_val_Dens     <-     summary(thereg)$coefficients$mean[2,4]
p_val_Dens
#&&&&&&&&&&&&&&&&&&&&&&&&&&&&&&&&&&&&&&&&&&&&&&&&&&& &&
##################################################################
#######
###### Calculate the predicted values using the expected proportion E(y)=μ that ######
#### can be computed by applying the inverse link function to the linear predictor #####
#### Similarly, we can also compute the variance to graph the confidence intervals ####
##################################################################
#######
mu      <-
```

```
PRED mu
phi  <-
PHI
phi
plot(prop_moved_day3 ~ Density, data = Male_moved_4, pch=16, ylim =c(0,0.25), ylab= " Mean
proportion of males that moved in 3days", xlab= " Density (number of
Adults/hamster)")
lines(mu ~ Density, data = Male_moved_4)
# Confidence Interval = x+tn-1, 1-α/2*(s/√n) n
<- 5
s <- sd_y
xmean <- mu
margin     <-     qt(0.975,df=n-1)*s/sqrt(n)
lowerinterval <- xmean - margin upperinterval
<- xmean + margin
for (j in 1:n) if(lowerinterval[j] <0) lowerinterval[j] <- 0.01
lowerinterval xmean
upperinterval
plot.new() plot(prop_moved_day3 ~ Density, data = Male_moved_4, pch=16, ylim =c(0,0.3), col=
"red",  ylab= "Mean proportion of males that moved on 3 days", xlab= " Density (number of
Adults/hamster)") # Beta regression lines(mu ~ Density, data = Male_moved_4, col= "blue", lwd= 2)
arrows(x0=Male_moved_4$Density, y0=xmean - margin, x1=Male_moved_4$Density, y1=xmean +
margin, angle=90, code=3, length=0.05, lwd= 1, lty= 1, col= "blue")
# NOTE: The confidence interval lines are the ones corresponding to the predictions of the beta regression
# We check if the variance of the Beta regression is a function of density of bugs
Dens_Var <- cbind(Male_moved_4$Density, Var_y)
Dens_Var
pp <- lm(Var_y ~ Male_moved_4$Density) summary(pp)
plot(Var_y ~ Male_moved_4$Density, pch=16) abline(pp)
beta_res[i,1]     <-     INT
beta_res[i,2]     <-     REG
beta_res[i,3]     <-     PHI
beta_res[i,4]     <-     LL
beta_res[i,5]     <-     DOF
beta_res[i,6]     <-     R_2
beta_res[i,7] <- p_val_Dens
  } # End of the "if" for i=8 (Males in 3 days)
} # This is the end of the loop of i= 1:8
```

```
beta_res
#$$$$$$$$$$$$$$$$$$$$$$$$$$$$$$$$$$$$$$$$$$$$$$$$$$$$$$$$$$$$$$$$$$$$
$$$
write.csv(beta_res, file="beta res prop males and females that moved.csv")
#$$$$$$$$$$$$$$$$$$$$$$$$$$$$$$$$$$$$$$$$$$$$$$$$$$$$$$$$$$$$$$$$$$$$
# Analysis of the bugs that moved (fed or not fed) as adults and Stage 5 nymphs
names(All_moved)
All_moved2 <- All_moved[,-4] # Delete Sex
All_moved2_n5 <- subset(All_moved2, All_moved2$Stage == 5)
All_moved2_ad <- subset(All_moved2, All_moved2$Stage == 6)
##### Adults #####
All_moved2_ad2 <- All_moved2_ad[complete.cases(All_moved2_ad), ] # Delete rows with NA
# Covert percentages into proportions and group by density for adults
feeding_ad_freq3_0 <- All_moved2_ad2 %>%
   group_by(Density) %>% mutate(Prop_moved_Day1 =
   N_moved_Day1/100,
   Prop_moved_Day2 = N_moved_Day2/100,
   Prop_moved_Day3 = N_moved_Day3/100,
   Prop_moved_3_Days          =          N_moved_3_Days/100)
feeding_ad_freq3_0     <-     as.data.frame(feeding_ad_freq3_0)
feeding_ad_freq3_0
feeding_ad_freq3_0       <-       feeding_ad_freq3_0[,-(5:8)]
feeding_ad_freq3_0
#  We  calculate  the  means  among  replicates  feeding_ad_freq3  <-
feeding_ad_freq3_0 %>%
   group_by(Density) %>%
   summarise(Prop_moved_in_day1= mean(Prop_moved_Day1),
                  Prop_moved_in_day2= mean(Prop_moved_Day2),
                  Prop_moved_in_day3= mean(Prop_moved_Day3),
Prop_moved_in_3_Days= mean(Prop_moved_3_Days),)
feeding_ad_fre
q3

##### Stage 5 nymphs #####
All_moved2_n52 <- All_moved2_n5[complete.cases(All_moved2_n5), ] # Delete rows with NA
# Covert percentages into proportions and group by density for n5ults
feeding_n5_freq3_0 <- All_moved2_n52 %>%
   group_by(Density) %>% mutate(Prop_moved_Day1 =
```

```
    N_moved_Day1/100,
    Prop_moved_Day2 = N_moved_Day2/100,
    Prop_moved_Day3 = N_moved_Day3/100,
    Prop_moved_3_Days          =          N_moved_3_Days/100)
feeding_n5_freq3_0     <-     as.data.frame(feeding_n5_freq3_0)
feeding_n5_freq3_0
feeding_n5_freq3_0       <-       feeding_n5_freq3_0[,-(5:8)]
feeding_n5_freq3_0
#  We  calculate  the  means  among  replicates  feeding_n5_freq3  <-
feeding_n5_freq3_0 %>%
    group_by(Density) %>%
    summarise(Prop_moved_in_day1=         mean(Prop_moved_Day1),         Prop_moved_in_day2=
                    mean(Prop_moved_Day2),
                    Prop_moved_in_day3= mean(Prop_moved_Day3),
Prop_moved_in_3_Days= mean(Prop_moved_3_Days),)
feeding_n5_fre
q3
#######################################################
##### Create a matrix to hold the various beta regression results #####
#######################################################
# NOTE: Here the 8 cases of the beta_res matrix correspond to the following beta regressions:
# For N5: Prop_moved Day1, Prop_moved Day2, Prop_moved Day3, Prop_moved 3-Days
# For Ad: Prop_moved Day1, Prop_moved Day2, Prop_moved Day3, Prop_moved 3-Days
beta_res <- data.frame(Intcpt = rep(0,8), Density = rep(0,8),Phi = rep(0,8), LL = rep(0,8),
DoF   =   rep(0,8),   Ps_R2   =   rep(0,8),   p_val_Dens   =   rep(0,8))
str(beta_res) for (i in 1:8) { if(i == 1) {
##### Proportion Adults that moved on day 1 #####
varname1 <- paste("day",i, sep = "")
varname <- paste("Proportion of bugs that moved in",varname1, sep = "")
datreg         <-         as.data.frame(feeding_ad_freq3)         actualvar         <-
datreg$Prop_moved_in_day1  regname1  <-  "beta_reg_prop_moved"  regname2  <-
paste("day",i, sep = "_")
regname3 <- paste(regname1, regname2, sep = "_")
xlabel <- "Density (number of Adults/hamster)"
regname3  <-  betareg(actualvar  ~  Density,  data  =  datreg)
summary(regname3)
###########################################################
##
```

```
#####   Extract   from   the   beta   regression   results   the   parameters   of   interest   #####
################################################################
### thereg <- regname3
# For the Intercept
INT <- thereg[[1]]$mean[1]; names(INT) <-NULL; INT
# For the regressor (Density)
REG <- thereg[[1]]$mean[2]; names(REG) <-NULL; REG
# For the precision  (Phi)
PHI <- thereg[[1]]$precision[1]; names(PHI) <- NULL; PHI
# The predicted values (mu)
PRED <- thereg[[3]]; names(PRED) <- NULL; PRED
# Maximum-likelihood
LL <- thereg[[5]]$value; LL
# Degrees of freedom
DOF <- thereg[[14]]; DOF
# Pseudo R-squared
R_2 <- thereg[[20]]; R_2
# p-value of the regressor "Density"
p_val_Dens  <-  summary(thereg)$coefficients$mean[2,4];  p_val_Dens  mu  <-
PRED; mu
phi <- PHI; phi
plot(actualvar ~ Density, data = datreg, pch=16, ylim =c(0,0.4), ylab= varname, xlab= xlabel)
lines(mu ~ Density, data = datreg)
# Confidence Interval = x+tn-1, 1-α/2*(s/√n) n <- 5; s <- sd_y; xmean <-
mu;  margin  <-  qt(0.975,df=n-1)*s/sqrt(n)  lowerinterval  <-  xmean  -
margin; upperinterval <- xmean + margin
for (j in 1:n) if(lowerinterval[j] <0) lowerinterval[j] <- 0.01
lowerinterval ; xmean; upperinterval
plot.new()
plot(actualvar ~ Density, data = datreg, pch=16, ylim =c(0,0.4), col= "red",  ylab= varname
, xlab=  xlabel)  lines(mu  ~  Density,  data  =  datreg,  col=
"blue",  lwd=  2)  arrows(x0=datreg$Density,  y0=xmean  -
margin, x1=datreg$Density, y1=xmean + margin,
      angle=90, code=3, length=0.05, lwd= 1, lty= 1, col= "blue")
# We check if the variance of the Beta regression is a function of density of bugs
Dens_Var <- cbind(datreg$Density, Var_y); Dens_Var pp <-
lm(Var_y  ~  datreg$Density);  summary(pp)  plot(Var_y  ~
datreg$Density, pch=16); abline(pp)
```

```
beta_res[i,1]        <-        INT
beta_res[i,2]        <-        REG
beta_res[i,3]        <-        PHI
beta_res[i,4]        <-        LL
beta_res[i,5]        <-        DOF
beta_res[i,6]        <-        R_2
beta_res[i,7] <- p_val_Dens
  } # end combination 1 (adults day 1) if(i == 2) {
  ##### Proportion Adults that moved on day 1 #####
varname1 <- paste("day",i, sep = "")
varname <- paste("Proportion of bugs that moved in",varname1, sep = " ") datreg
<- as.data.frame(feeding_ad_freq3)
actualvar <- datreg$Prop_moved_in_day2
regname1 <- "beta_reg_prop_moved"
regname2 <- paste("day",i, sep = "_")
regname3 <- paste(regname1, regname2, sep = "_")
xlabel <- "Density (number of Adults/hamster)"
regname3 <- betareg(actualvar ~ Density, data = datreg)
summary(regname3)
##############################################################
##
##### Extract from the beta regression results the parameters of interest #####
#############################################################
### thereg <- regname3
# For the Intercept
INT <- thereg[[1]]$mean[1]; names(INT) <-NULL; INT
# For the regressor (Density)
REG <- thereg[[1]]$mean[2]; names(REG) <-NULL; REG
# For the precision  (Phi)
PHI <- thereg[[1]]$precision[1]; names(PHI) <- NULL; PHI
# The predicted values (mu)
PRED <- thereg[[3]]; names(PRED) <- NULL; PRED
# Maximum-likelihood
LL <- thereg[[5]]$value; LL
# Degrees of freedom
DOF <- thereg[[14]]; DOF
# Pseudo R-squared
R_2 <- thereg[[20]]; R_2
```

```
# p-value of the regressor "Density"
p_val_Dens <- summary(thereg)$coefficients$mean[2,4]; p_val_Dens mu <-
PRED; mu
phi <- PHI; phi
plot(actualvar ~ Density, data = datreg, pch=16, ylim =c(0,0.4), ylab= varname, xlab= xlabel)
lines(mu ~ Density, data = datreg)
# Confidence Interval = x+tn-1, 1-α/2*(s/√n) n <- 5; s <- sd_y; xmean <-
mu; margin <- qt(0.975,df=n-1)*s/sqrt(n) lowerinterval <- xmean -
margin; upperinterval <- xmean + margin
for (j in 1:n) if(lowerinterval[j] <0) lowerinterval[j] <- 0.01
lowerinterval ; xmean; upperinterval
plot.new()
plot(actualvar ~ Density, data = datreg, pch=16, ylim =c(0,0.4), col= "red", ylab= varname
, xlab= xlabel) lines(mu ~ Density, data = datreg, col=
"blue", lwd= 2) arrows(x0=datreg$Density, y0=xmean -
margin, x1=datreg$Density, y1=xmean + margin,
      angle=90, code=3, length=0.05, lwd= 1, lty= 1, col= "blue")
# We check if the variance of the Beta regression is a function of density of bugs
Dens_Var <- cbind(datreg$Density, Var_y); Dens_Var pp <-
lm(Var_y ~ datreg$Density); summary(pp) plot(Var_y ~
datreg$Density, pch=16); abline(pp)
beta_res[i,1]      <-      INT
beta_res[i,2]      <-      REG
beta_res[i,3]      <-      PHI
beta_res[i,4]      <-      LL
beta_res[i,5]      <-      DOF
beta_res[i,6]      <-      R_2
beta_res[i,7] <- p_val_Dens
} # end combination 2
(adults day 2) if(i == 3) {
##### Proportion Adults that moved on day 3 #####
varname1 <- paste("day",i, sep = "")
varname <- paste("Proportion of bugs that moved in",varname1, sep = " ") datreg
<- as.data.frame(feeding_ad_freq3)
actualvar <- datreg$Prop_moved_in_day3
regname1 <- "beta_reg_prop_moved"
regname2 <- paste("day",i, sep = "_")
regname3 <- paste(regname1, regname2, sep = "_")
```

```
xlabel <- "Density (number of Adults/hamster)"
regname3 <- betareg(actualvar ~ Density, data = datreg)
summary(regname3)
################################################################
##
#####  Extract from the beta regression results the parameters of interest  #####
###############################################################
### thereg <- regname3
# For the Intercept
INT <- thereg[[1]]$mean[1]; names(INT) <-NULL; INT
# For the regressor (Density)
REG <- thereg[[1]]$mean[2]; names(REG) <-NULL; REG
# For the precision  (Phi)
PHI <- thereg[[1]]$precision[1]; names(PHI) <- NULL; PHI
# The predicted values (mu)
PRED <- thereg[[3]]; names(PRED) <- NULL; PRED
# Maximum-likelihood
LL <- thereg[[5]]$value; LL
# Degrees of freedom
DOF <- thereg[[14]]; DOF
# Pseudo R-squared
R_2 <- thereg[[20]]; R_2
# p-value of the regressor "Density"
p_val_Dens <- summary(thereg)$coefficients$mean[2,4]; p_val_Dens mu <-
PRED; mu
phi <- PHI; phi
plot(actualvar ~ Density, data = datreg, pch=16, ylim =c(0,0.4), ylab= varname, xlab= xlabel)
lines(mu ~ Density, data = datreg)
# Confidence Interval = x+tn-1, 1-α/2*(s/√n)
n <- 5; s <- sd_y; xmean <- mu; margin <- qt(0.975,df=n-1)*s/sqrt(n)
lowerinterval <- xmean - margin; upperinterval <- xmean + margin
for (j in 1:n) if(lowerinterval[j] <0) lowerinterval[j] <- 0.01
lowerinterval ; xmean; upperinterval
plot.new()
plot(actualvar ~ Density, data = datreg, pch=16, ylim =c(0,0.4), col= "red",  ylab= varname
, xlab= xlabel) lines(mu ~ Density, data = datreg, col=
"blue", lwd= 2) arrows(x0=datreg$Density, y0=xmean -
margin, x1=datreg$Density, y1=xmean + margin,
```

```
    angle=90, code=3, length=0.05, lwd= 1, lty= 1, col= "blue")
# We check if the variance of the Beta regression is a function of density of bugs
Dens_Var <- cbind(datreg$Density, Var_y); Dens_Var pp <-
lm(Var_y ~ datreg$Density); summary(pp) plot(Var_y ~
datreg$Density, pch=16); abline(pp)
beta_res[i,1]     <-     INT
beta_res[i,2]     <-     REG
beta_res[i,3]     <-     PHI
beta_res[i,4]     <-     LL
beta_res[i,5]     <-     DOF
beta_res[i,6]     <-     R_2
beta_res[i,7] <- p_val_Dens
  } # end combination 3 (adults day 3) if(i == 4) {
  ##### Proportion Adults that moved in 3 days #####
  varname <- paste("Proportion of bugs that moved in 3 days") datreg
  <- as.data.frame(feeding_ad_freq3)
  actualvar <- datreg$Prop_moved_in_3_Days
  regname1 <- "beta_reg_prop_moved"
  regname2 <- paste("day",i, sep = "_")
  regname3 <- paste(regname1, regname2, sep = "_")
  xlabel <- "Density (number of Adults/hamster)"
regname3 <- betareg(actualvar ~ Density, data = datreg)
summary(regname3)
#################################################################
##
#####  Extract from the beta regression results the parameters of interest  #####
################################################################
### thereg <- regname3
# For the Intercept
INT <- thereg[[1]]$mean[1]; names(INT) <-NULL; INT
# For the regressor (Density)
REG <- thereg[[1]]$mean[2]; names(REG) <-NULL; REG
# For the precision  (Phi)
PHI <- thereg[[1]]$precision[1]; names(PHI) <- NULL; PHI
# The predicted values (mu)
PRED <- thereg[[3]]; names(PRED) <- NULL; PRED
# Maximum-likelihood
LL <- thereg[[5]]$value; LL
```

```
# Degrees of freedom
DOF <- thereg[[14]]; DOF
# Pseudo R-squared
R_2 <- thereg[[20]]; R_2
# p-value of the regressor "Density"
p_val_Dens  <-  summary(thereg)$coefficients$mean[2,4];  p_val_Dens  mu  <-
PRED; mu
phi <- PHI; phi
plot(actualvar ~ Density, data = datreg, pch=16, ylim =c(0,0.4), ylab= varname, xlab= xlabel)
lines(mu ~ Density, data = datreg)
# Confidence Interval = x+tn-1, 1-α/2*(s/√n) n <- 5; s <- sd_y; xmean <-
mu;  margin  <-  qt(0.975,df=n-1)*s/sqrt(n)  lowerinterval  <-  xmean  -
margin; upperinterval <- xmean + margin
for (j in 1:n) if(lowerinterval[j] <0) lowerinterval[j] <- 0.01
lowerinterval ; xmean; upperinterval
plot.new()
plot(actualvar ~ Density, data = datreg, pch=16, ylim =c(0,0.4), col= "red",  ylab= varname
, xlab= xlabel) lines(mu ~ Density, data = datreg, col=
"blue", lwd= 2) arrows(x0=datreg$Density, y0=xmean -
margin, x1=datreg$Density, y1=xmean + margin,
      angle=90, code=3, length=0.05, lwd= 1, lty= 1, col= "blue")
# We check if the variance of the Beta regression is a function of density of bugs
Dens_Var <- cbind(datreg$Density, Var_y); Dens_Var pp <-
lm(Var_y  ~  datreg$Density);  summary(pp)  plot(Var_y  ~
datreg$Density, pch=16); abline(pp)
beta_res[i,1]      <-      INT
beta_res[i,2]      <-      REG
beta_res[i,3]      <-      PHI
beta_res[i,4]      <-      LL
beta_res[i,5]      <-      DOF
beta_res[i,6]      <-      R_2
beta_res[i,7] <- p_val_Dens
   } # end combination 4 (adults in 3 days) if(i == 5) {
   ##### Proportion stage 5 nymphs that moved on day 1 #####
varname1 <- paste("day",i-4, sep = "")
varname <- paste("Proportion of bugs that moved in",varname1, sep = "")
datreg <- as.data.frame(feeding_n5_freq3)
actualvar <- datreg$Prop_moved_in_day1
```

```
regname1 <- "beta_reg_prop_moved"
regname2 <- paste("day",i-4, sep = "_")
regname3 <- paste(regname1, regname2, sep = "_") xlabel
<- "Density (number of stage 5 nymphs/hamster)"
regname3 <- betareg(actualvar ~ Density, data = datreg)
summary(regname3)
##################################################################
##
##### Extract from the beta regression results the parameters of interest #####
#################################################################
### thereg <- regname3
# For the Intercept
INT <- thereg[[1]]$mean[1]; names(INT) <-NULL; INT
# For the regressor (Density)
REG <- thereg[[1]]$mean[2]; names(REG) <-NULL; REG
# For the precision (Phi)
PHI <- thereg[[1]]$precision[1]; names(PHI) <- NULL; PHI
# The predicted values (mu)
PRED <- thereg[[3]]; names(PRED) <- NULL; PRED
# Maximum-likelihood
LL <- thereg[[5]]$value; LL
# Degrees of freedom
DOF <- thereg[[14]]; DOF
# Pseudo R-squared
R_2 <- thereg[[20]]; R_2
# p-value of the regressor "Density"
p_val_Dens <- summary(thereg)$coefficients$mean[2,4]; p_val_Dens mu <-
PRED; mu
phi <- PHI; phi
plot(actualvar ~ Density, data = datreg, pch=16, ylim =c(0,0.4), ylab= varname, xlab= xlabel)
lines(mu ~ Density, data = datreg)
# Confidence Interval = x+tn-1, 1-α/2*(s/√n) n <- 5; s <- sd_y; xmean <-
mu; margin <- qt(0.975,df=n-1)*s/sqrt(n)
lowerinterval <- xmean - margin; upperinterval <- xmean + margin for (j in
1:n)
if(lowerinterval[j] <0) lowerinterval[j] <- 0.01
lowerinterval ; xmean; upperinterval
plot.new()
```

```
plot(actualvar ~ Density, data = datreg, pch=16, ylim =c(0,0.4), col= "red",  ylab= varname
, xlab= xlabel) lines(mu ~ Density, data = datreg, col=
"blue", lwd= 2) arrows(x0=datreg$Density, y0=xmean -
margin, x1=datreg$Density, y1=xmean + margin,
      angle=90, code=3, length=0.05, lwd= 1, lty= 1, col= "blue")
# We check if the variance of the Beta regression is a function of density of bugs
Dens_Var <- cbind(datreg$Density, Var_y); Dens_Var pp <-
lm(Var_y ~ datreg$Density); summary(pp) plot(Var_y ~
datreg$Density, pch=16); abline(pp)
beta_res[i,1]      <-      INT
beta_res[i,2]      <-      REG
beta_res[i,3]      <-      PHI
beta_res[i,4]      <-      LL
beta_res[i,5]      <-      DOF
beta_res[i,6]      <-      R_2
beta_res[i,7] <- p_val_Dens
   } # end combination 5 (stage 5 nymphs day 1) if(i == 6) {
   ##### Proportion stage 5 nymphs that moved on day 2 #####
varname1 <- paste("day",i-4, sep = "")
varname <- paste("Proportion of bugs that moved in",varname1, sep = " ") datreg
<- as.data.frame(feeding_n5_freq3) actualvar <- datreg$Prop_moved_in_day2
regname1 <- "beta_reg_prop_moved" regname2 <- paste("day",i-4, sep = "_")
regname3 <- paste(regname1, regname2, sep = "_") xlabel <- "Density (number of
stage 5 nymphs/hamster)"
regname3 <- betareg(actualvar ~ Density, data = datreg)
summary(regname3)


`
################################################################
##
#####   Extract   from   the   beta   regression   results   the   parameters   of   interest   #####
###############################################################
### thereg <- regname3
# For the Intercept
INT <- thereg[[1]]$mean[1]; names(INT) <-NULL; INT
# For the regressor (Density)
REG <- thereg[[1]]$mean[2]; names(REG) <-NULL; REG
```

```
# For the precision  (Phi)
PHI <- thereg[[1]]$precision[1]; names(PHI) <- NULL; PHI
# The predicted values (mu)
PRED <- thereg[[3]]; names(PRED) <- NULL; PRED
# Maximum-likelihood
LL <- thereg[[5]]$value; LL
# Degrees of freedom
DOF <- thereg[[14]]; DOF
# Pseudo R-squared
R_2 <- thereg[[20]]; R_2
# p-value of the regressor "Density"
p_val_Dens  <-  summary(thereg)$coefficients$mean[2,4];  p_val_Dens  mu  <-
PRED; mu
phi <- PHI; phi
plot(actualvar ~ Density, data = datreg, pch=16, ylim =c(0,0.4), ylab= varname, xlab= xlabel)
lines(mu ~ Density, data = datreg)
# Confidence Interval = x+tn-1, 1-α/2*(s/√n) n <- 5; s <- sd_y; xmean <-
mu;  margin  <-  qt(0.975,df=n-1)*s/sqrt(n)  lowerinterval  <-  xmean  -
margin; upperinterval <- xmean + margin for (j in 1:n) if(lowerinterval[j]
<0) lowerinterval[j] <- 0.01 lowerinterval ; xmean; upperinterval plot.new()
plot(actualvar ~ Density, data = datreg, pch=16, ylim =c(0,0.4), col= "red",  ylab= varname
, xlab=  xlabel)  lines(mu  ~  Density,  data  =  datreg,  col=
"blue",  lwd=  2)  arrows(x0=datreg$Density,  y0=xmean  -
margin, x1=datreg$Density, y1=xmean + margin,
      angle=90, code=3, length=0.05, lwd= 1, lty= 1, col= "blue")
# We check if the variance of the Beta regression is a function of density of bugs
Dens_Var <- cbind(datreg$Density, Var_y); Dens_Var pp <-
lm(Var_y  ~  datreg$Density);  summary(pp)  plot(Var_y  ~
datreg$Density, pch=16); abline(pp)
beta_res[i,1]      <-      INT
beta_res[i,2]      <-      REG
beta_res[i,3]      <-      PHI
beta_res[i,4]      <-      LL
beta_res[i,5]      <-      DOF
beta_res[i,6]      <-      R_2
beta_res[i,7] <- p_val_Dens
   } # end combination 6 (stage 5 nymphs day 2) if(i == 7) { ##### Proportion
   stage 5 nymphs that moved on day 3 #####
```

```
varname1 <- paste("day",i-4, sep = "")
varname <- paste("Proportion of bugs that moved in",varname1, sep = " ") datreg
<- as.data.frame(feeding_n5_freq3) actualvar <- datreg$Prop_moved_in_day3
regname1 <- "beta_reg_prop_moved" regname2 <- paste("day",i-4, sep = "_")
regname3 <- paste(regname1, regname2, sep = "_") xlabel <- "Density (number of
stage 5 nymphs/hamster)"
regname3 <- betareg(actualvar ~ Density, data = datreg)
summary(regname3)
##################################################################
##
##### Extract from the beta regression results the parameters of interest #####
#################################################################
### thereg <- regname3
# For the Intercept
INT <- thereg[[1]]$mean[1]; names(INT) <-NULL; INT
# For the regressor (Density)
REG <- thereg[[1]]$mean[2]; names(REG) <-NULL; REG
# For the precision  (Phi)
PHI <- thereg[[1]]$precision[1]; names(PHI) <- NULL; PHI
# The predicted values (mu)
PRED <- thereg[[3]]; names(PRED) <- NULL; PRED
# Maximum-likelihood
LL <- thereg[[5]]$value; LL
# Degrees of freedom
DOF <- thereg[[14]]; DOF
# Pseudo R-squared
R_2 <- thereg[[20]]; R_2
# p-value of the regressor "Density"
p_val_Dens <- summary(thereg)$coefficients$mean[2,4]; p_val_Dens mu <-
PRED; mu
phi <- PHI; phi
plot(actualvar ~ Density, data = datreg, pch=16, ylim =c(0,0.4), ylab= varname, xlab= xlabel)
lines(mu ~ Density, data = datreg)
# Confidence Interval = x+tn-1, 1-α/2*(s/√n) n <- 5; s <- sd_y; xmean <-
mu; margin <- qt(0.975,df=n-1)*s/sqrt(n) lowerinterval <- xmean -
margin; upperinterval <- xmean + margin
for (j in 1:n) if(lowerinterval[j] <0) lowerinterval[j] <- 0.01
lowerinterval ; xmean; upperinterval
```

```
plot.new()
plot(actualvar ~ Density, data = datreg, pch=16, ylim =c(0,0.4), col= "red",  ylab= varname
, xlab= xlabel) lines(mu ~ Density, data = datreg, col=
"blue", lwd= 2) arrows(x0=datreg$Density, y0=xmean -
margin, x1=datreg$Density, y1=xmean + margin,
      angle=90, code=3, length=0.05, lwd= 1, lty= 1, col= "blue")
# We check if the variance of the Beta regression is a function of density of bugs
Dens_Var <- cbind(datreg$Density, Var_y); Dens_Var pp <-
lm(Var_y ~ datreg$Density); summary(pp) plot(Var_y ~
datreg$Density, pch=16); abline(pp)
beta_res[i,1]     <-     INT
beta_res[i,2]     <-     REG
beta_res[i,3]     <-     PHI
beta_res[i,4]     <-     LL
beta_res[i,5]     <-     DOF
beta_res[i,6]     <-     R_2
beta_res[i,7] <- p_val_Dens
   } # end combination 7 (stage 5 nymphs day 3) if(i == 8) { ##### Proportion
   stage 5 nymphs that moved in  3 days #####
varname1 <- paste("day",i-4, sep = "")
varname <- paste("Proportion of bugs that moved in",varname1, sep = " ") datreg
<- as.data.frame(feeding_n5_freq3) actualvar <- datreg$Prop_moved_in_3_Days
regname1 <- "beta_reg_prop_moved" regname2 <- paste("day",i-4, sep = "_")
regname3 <- paste(regname1, regname2, sep = "_") xlabel <- "Density (number of
stage 5 nymphs/hamster)"
regname3 <- betareg(actualvar ~ Density, data = datreg)
summary(regname3)
################################################################
##
#####  Extract  from  the  beta  regression  results  the  parameters  of  interest  #####
###############################################################
### thereg <- regname3
# For the Intercept
INT <- thereg[[1]]$mean[1]; names(INT) <-NULL; INT
# For the regressor (Density)
REG <- thereg[[1]]$mean[2]; names(REG) <-NULL; REG
# For the precision  (Phi)
PHI <- thereg[[1]]$precision[1]; names(PHI) <- NULL; PHI
```

```
# The predicted values (mu)
PRED <- thereg[[3]]; names(PRED) <- NULL; PRED
# Maximum-likelihood
LL <- thereg[[5]]$value; LL
# Degrees of freedom
DOF <- thereg[[14]]; DOF
# Pseudo R-squared
R_2 <- thereg[[20]]; R_2
# p-value of the regressor "Density"
p_val_Dens <- summary(thereg)$coefficients$mean[2,4]; p_val_Dens mu <-
PRED; mu
phi <- PHI; phi
plot(actualvar ~ Density, data = datreg, pch=16, ylim =c(0,0.4), ylab= varname, xlab= xlabel)
lines(mu ~ Density, data = datreg)
# Confidence Interval = x+tn-1, 1-α/2*(s/√n) n <- 5; s <- sd_y; xmean <-
mu; margin <- qt(0.975,df=n-1)*s/sqrt(n) lowerinterval <- xmean -
margin; upperinterval <- xmean + margin
for (j in 1:n) if(lowerinterval[j] <0) lowerinterval[j] <- 0.01
lowerinterval ; xmean; upperinterval
plot.new()
plot(actualvar ~ Density, data = datreg, pch=16, ylim =c(0,0.4), col= "red", ylab= varname
, xlab= xlabel) lines(mu ~ Density, data = datreg, col=
"blue", lwd= 2) arrows(x0=datreg$Density, y0=xmean -
margin, x1=datreg$Density, y1=xmean + margin,
      angle=90, code=3, length=0.05, lwd= 1, lty= 1, col= "blue")
# We check if the variance of the Beta regression is a function of density of bugs Dens_Var <-
cbind(datreg$Density, Var_y); Dens_Var pp <- lm(Var_y ~ datreg$Density); summary(pp) plot(Var_y ~
datreg$Density, pch=16); abline(pp)
beta_res[i,1]    <-    INT
beta_res[i,2]    <-    REG
beta_res[i,3]    <-    PHI
beta_res[i,4]    <-    LL
beta_res[i,5]    <-    DOF
beta_res[i,6]    <-    R_2
beta_res[i,7] <- p_val_Dens
   } # end combination 8 (stage 5 nymphs in 3 days)
} # This is the end of the loop of i= 1:8 beta_res
write.csv(beta_res, file="beta res prop adults and stage 5 nymphs that moved (fed and not fed).csv")
```

```
########## End for Percent mortality in the three-day trials ##########
##############################################
####   Proportion   N5   molting   in   the   three-day   trials   #####
##############################################
# position <- c(1:5,22)
molting_n5 <- molting_longevity[1:18,position] molting_n5
molting_n5_2  <-  molting_n5  %>%  group_by(Density)
        %>%
        summarise(Mean_prop_N5_Molt= mean(Pct_molted/100))
molting_n5_3 <- as.data.frame(molting_n5_2) molting_n5_3
##############################################
#####     Proportion     Stage     5     nymphs     that     fed     on     Day     1     #####
##############################################
beta_reg_prop_molting_n5_3 <- betareg(Mean_prop_N5_Molt ~ Density, data = molting_n5_3)
summary(beta_reg_prop_molting_n5_3)
#################################################################
##
##### Extract from the beta regression results the parameters of interest #####
####### Calculate the predicted values using the expected proportion E(y)=μ that #######
##### can be computed by applying the inverse link function to the linear predictor ######
#################################################################
########
###
mu
<-
PRED
mu
phi  <-
PHI
phi
# Prediction of the variance of the predicted proportion fed
Var_y <- mu * (1 - mu)/(1+phi)
Var_y
sd_y <- sqrt(Var_y)
sd_y
par(mar = c(5.1, 6, 4.1, 2.1)) plot(Mean_prop_N5_Molt ~ Density, data = molting_n5_3, pch=16,
ylim =c(0,0.6), ylab= "Mean proportion of stage 5 nymphs\n molting in a three-week period", xlab= "
Density
```

```
(number of stage 5 nymphs/hamster)") lines(mu ~
Density, data = molting_n5_3)
# Confidence Interval = x+tn-1, 1-α/2*(s/√n)
n <- 5
s <- sd_y
xmean <- mu
margin <- qt(0.975,df=n-1)*s/sqrt(n)
lowerinterval <- xmean - margin upperinterval
<- xmean + margin
for (j in 1:n) if(lowerinterval[j] <0) lowerinterval[j] <- 0.01
lowerinterval xmean
upperinterval
par(mar = c(5.1, 6, 4.1, 2.1)) plot.new() plot(Mean_prop_N5_Molt ~ Density, data = molting_n5_3,
pch=16, ylim =c(0,0.8), col= " red", ylab= "Mean proportion of stage 5 nymphs\n molting in a three-
week period", xlab=
" Density (number of stage 5 nymphs/hamster)") lines(mu ~
Density, data = molting_n5_3, col= "blue", lwd= 3)
arrows(x0=molting_n5_3$Density, y0=xmean - margin,
x1=molting_n5_3$Density, y1=xmean + margin, angle=90, code=3,
length=0.05, lwd= 1, lty= 1, col= "blue")
# We check if the variance of the Beta regression is a function of density of bugs
Dens_Var <- cbind(molting_n5_3$Density, Var_y) Dens_Var
pp <- lm(Var_y ~ molting_n5_3$Density) summary(pp)
plot(Var_y ~ molting_n5_3$Density, pch=16) abline(pp)
########## End for Proportion N5 molting in the three-day trials ##########
##########################################################
########## Prop Surv Adult females in the 3-weeks period ##########
##########################################################
surviving_fema_3w <- molting_longevity[,1:10]
surviving_fema_3w_b <- subset(surviving_fema_3w, surviving_fema_3w$Stage == 6 &
surviving_fema_3w$Sex == 1)
surviving_fema_3w_c <- surviving_fema_3w_b[,-(3:7)]
surviving_fema_3w_c
surviving_fema_3w_d <- surviving_fema_3w_c[-(1:17),]
surviving_fema_3w_d
surviving_fema_3w_e <- surviving_fema_3w_d %>% mutate(prop_surv_week1
  = N_Fem_Alive_week1/Density, prop_surv_week2 =
  N_Fem_Alive_week2/Density, prop_surv_week3 =
```

```
    N_Fem_Alive_week3/Density,
    prop_surv_3weeks = prop_surv_week1*prop_surv_week2*prop_surv_week3)
surviving_fema_3w_e
surviving_fema_3w_f      <-      surviving_fema_3w_e[,-(3:5)]
surviving_fema_3w_f
surviving_fema_3w_g <- surviving_fema_3w_f %>%
    group_by(Density) %>%
    summarise_at(vars(prop_surv_week1,prop_surv_week2,prop_surv_week3,          prop_surv_3weeks),
    funs(mean))
surviving_fema_3w_g <- as.data.frame(surviving_fema_3w_g) surviving_fema_3w_g
##############################################################
#####    Create    a    matrix    to    hold    the    various    beta    regression    results    #####
##############################################################
beta_res <- data.frame(Intcpt = rep(0,4), Density = rep(0,4),Phi = rep(0,4), LL = rep(0,4),
DoF  =  rep(0,4),  Ps_R2  =  rep(0,4),  p_val_Dens  =  rep(0,4))
str(beta_res)
for   (i   in
   1:4)   {
   if(i   ==
   1) {
##################################################
######       Proportion       females       that       survived       on       week       1       #####
##################################################
beta_reg_prop_surv_fem_week1 <- betareg(prop_surv_week1 ~ Density, data = surviving_fema_3w_g)
summary(beta_reg_prop_surv_fem_week1)
###################################################################
##   #####   Extract   from   the   beta   regression   results   the   parameters   of   interest   #####
###################################################################
## thereg <- beta_reg_prop_surv_fem_week1
# For the Intercept
INT       <-       thereg[[1]]$mean[1]
names(INT) <-NULL
INT
#  For  the  regressor  (Density)
REG  <-  thereg[[1]]$mean[2]
names(REG) <-NULL
REG
# For the precision  (Phi)
```

```
PHI <- thereg[[1]]$precision[1]
names(PHI) <- NULL
PHI
#  The  predicted  values  (mu)
PRED        <-        thereg[[3]]
names(PRED) <- NULL
PRED
# Maximum-likelihood
LL <- thereg[[5]]$value
LL
# Degrees of freedom
DOF <- thereg[[14]]
DOF
# Pseudo R-squared
R_2 <- thereg[[20]]
R_2
# p-value of the regressor "Density"
p_val_Dens    <-    summary(thereg)$coefficients$mean[2,4]
p_val_Dens
##################################################################
######## ###
####### Calculate the predicted values using the expected proportion E(y)=μ that #######
##### can be computed by applying the inverse link function to the linear predictor ######
##################################################################
########
###
mu
<-
PRED
mu
phi  <-
PHI
phi
# Prediction of the variance of the predicted proportion fed
Var_y <- mu * (1 - mu)/(1+phi)
Var_y
sd_y <- sqrt(Var_y)
sd_y
```

```
plot(prop_surv_week1 ~ Density, data = surviving_fema_3w_g, pch=16, ylim =c(0.2,0.5), ylab= " Mean
proportion adults that died on week 1", xlab= " Density (number of adults/hamster)")
lines(mu ~ Density, data = surviving_fema_3w_g)
# Confidence Interval = x+tn-1, 1-α/2*(s/√n) n
<- 5
s <- sd_y
xmean <- mu
margin <- qt(0.975,df=n-1)*s/sqrt(n)
lowerinterval <- xmean - margin upperinterval
<- xmean + margin
for (j in 1:n) if(lowerinterval[j] <0) lowerinterval[j] <- 0.01
lowerinterval xmean
upperinterval
par(mar = c(5.1, 6, 4.1, 2.1)) plot.new() plot(prop_surv_week1 ~ Density, data =
surviving_fema_3w_g, pch=16, ylim =c(0.2,0.5), col= " red", ylab= "Mean proportion adults that died
on week 1", xlab= " Density (number of adults/hamster)") lines(mu ~ Density, data =
surviving_fema_3w_g, col= "blue", lwd= 3) arrows(x0=surviving_fema_3w_g$Density, y0=xmean -
margin, x1=surviving_fema_3w_g$Density, y1=xmean + margin, angle=90, code=3, length=0.05,
lwd= 1, lty= 1, col= "blue")
# We check if the variance of the Beta regression is a function of density of bugs
Dens_Var <- cbind(surviving_fema_3w_g$Density, Var_y)
Dens_Var
pp <- lm(Var_y ~ surviving_fema_3w_g$Density)
summary(pp)
plot(Var_y ~ surviving_fema_3w_g$Density, pch=16)
abline(pp)
beta_res[i,1] <- INT
beta_res[i,2] <- REG
beta_res[i,3] <- PHI
beta_res[i,4] <- LL
beta_res[i,5] <- DOF
beta_res[i,6] <- R_2
beta_res[i,7] <- p_val_Dens
} # End of the "if" for i=1 (Fem/Week1)
if (i == 2) {
############################################
###### Proportion females that survived on week 2 #####
############################################
```

```
beta_reg_prop_surv_fem_week2 <- betareg(prop_surv_week2 ~ Density, data = surviving_fema_3w_g)
summary(beta_reg_prop_surv_fem_week2)
################################################################
##
#####   Extract   from   the   beta   regression   results   the   parameters   of   interest   #####
################################################################
### thereg <- beta_reg_prop_surv_fem_week2
# For the Intercept
INT <- thereg[[1]]$mean[1]
names(INT) <-NULL
INT
#  For  the  regressor  (Density)
REG  <-  thereg[[1]]$mean[2]
names(REG) <-NULL
REG
# For the precision  (Phi)
PHI <- thereg[[1]]$precision[1]
names(PHI) <- NULL
PHI
#  The  predicted  values  (mu)
PRED        <-        thereg[[3]]
names(PRED) <- NULL
PRED
# Maximum-likelihood
LL <- thereg[[5]]$value
LL
# Degrees of freedom
DOF <- thereg[[14]]
DOF
# Pseudo R-squared
R_2 <- thereg[[20]]
R_2
# p-value of the regressor "Density"
p_val_Dens     <-     summary(thereg)$coefficients$mean[2,4]
p_val_Dens
################################################################
######## ###
####### Calculate the predicted values using the expected proportion E(y)=μ that #######
```

```
##### can be computed by applying the inverse link function to the linear predictor ######
################################################################
########
###
mu
<-
PRED
mu
phi  <-
PHI
phi
# Prediction of the variance of the predicted proportion fed
Var_y <- mu * (1 - mu)/(1+phi)
Var_y
sd_y <- sqrt(Var_y)
sd_y
plot(prop_surv_week2 ~ Density, data = surviving_fema_3w_g, pch=16, ylim =c(0.2,0.5), ylab= " Mean
proportion adults that died on week 2", xlab= " Density (number of adults/hamster)")
lines(mu ~ Density, data = surviving_fema_3w_g)
# Confidence Interval = x+tn-1, 1-α/2*(s/√n) n
<- 5
s <- sd_y
xmean <- mu
margin  <-  qt(0.975,df=n-1)*s/sqrt(n)
lowerinterval <- xmean - margin upperinterval
<- xmean + margin
for (j in 1:n) if(lowerinterval[j] <0) lowerinterval[j] <- 0.01
lowerinterval xmean
upperinterval
par(mar = c(5.1, 6, 4.1, 2.1)) plot.new() plot(prop_surv_week2 ~ Density, data =
surviving_fema_3w_g, pch=16, ylim =c(0.2,0.5), col= " red", ylab= "Mean proportion adults that died
on week 2", xlab= " Density (number of adults/hamster)") lines(mu ~ Density, data =
surviving_fema_3w_g, col= "blue", lwd= 3) arrows(x0=surviving_fema_3w_g$Density, y0=xmean -
margin, x1=surviving_fema_3w_g$Density, y1=xmean + margin, angle=90, code=3, length=0.05,
lwd= 1, lty= 1, col= "blue")
# We check if the variance of the Beta regression is a function of density of bugs
Dens_Var <- cbind(surviving_fema_3w_g$Density, Var_y)
Dens_Var
```

```
pp <- lm(Var_y ~ surviving_fema_3w_g$Density)
summary(pp)
plot(Var_y ~ surviving_fema_3w_g$Density, pch=16)
abline(pp)
beta_res[i,1] <- INT
beta_res[i,2] <- REG
beta_res[i,3] <- PHI
beta_res[i,4] <- LL
beta_res[i,5] <- DOF
beta_res[i,6] <- R_2
beta_res[i,7] <- p_val_Dens
} # End of the "if" for i=2 (Fem/Week2)
if (i == 3) {
#################################################
###### Proportion females that survived on week 2 #####
#################################################
beta_reg_prop_surv_fem_week3 <- betareg(prop_surv_week3 ~ Density, data = surviving_fema_3w_g)
summary(beta_reg_prop_surv_fem_week3)
#################################################################
##
##### Extract from the beta regression results the parameters of interest #####
################################################################
### thereg <- beta_reg_prop_surv_fem_week3
# For the Intercept
INT <- thereg[[1]]$mean[1]
names(INT) <-NULL
INT
# For the regressor (Density)
REG <- thereg[[1]]$mean[2]
names(REG) <-NULL
REG
# For the precision (Phi)
PHI <- thereg[[1]]$precision[1]
names(PHI) <- NULL
PHI
# The predicted values (mu)
PRED <- thereg[[3]]
names(PRED) <- NULL
```

```
PRED
# Maximum-likelihood
LL <- thereg[[5]]$value
LL
# Degrees of freedom
DOF <- thereg[[14]]
DOF
# Pseudo R-squared
R_2 <- thereg[[20]]
R_2
# p-value of the regressor "Density"
p_val_Dens     <-     summary(thereg)$coefficients$mean[2,4]
p_val_Dens
#################################################################
######## ###
####### Calculate the predicted values using the expected proportion E(y)=μ that #######
##### can be computed by applying the inverse link function to the linear predictor ######
#################################################################
########
###
mu
<-
PRED
mu
phi  <-
PHI
phi
# Prediction of the variance of the predicted proportion fed
Var_y <- mu * (1 - mu)/(1+phi)
Var_y
sd_y <- sqrt(Var_y)
sd_y
plot(prop_surv_week3 ~ Density, data = surviving_fema_3w_g, pch=16, ylim =c(0,0.4), ylab= " Mean
proportion adults that died on week 3", xlab= " Density (number of adults/hamster)")
lines(mu ~ Density, data = surviving_fema_3w_g)
# Confidence Interval = x+tn-1, 1-α/2*(s/√n) n
<- 5
s <- sd_y
```

```
xmean <- mu
margin <- qt(0.975,df=n-1)*s/sqrt(n)
lowerinterval <- xmean - margin upperinterval
<- xmean + margin
for (j in 1:n) if(lowerinterval[j] <0) lowerinterval[j] <- 0.01
lowerinterval xmean
upperinterval
par(mar = c(5.1, 6, 4.1, 2.1)) plot.new() plot(prop_surv_week3 ~ Density, data =
surviving_fema_3w_g, pch=16, ylim =c(0,0.4), col = " red", ylab= "Mean proportion adults that died on
week 3", xlab= " Density (number of adults/hamster)") lines(mu ~ Density, data =
surviving_fema_3w_g, col= "blue", lwd= 3) arrows(x0=surviving_fema_3w_g$Density, y0=xmean -
margin, x1=surviving_fema_3w_g$Density, y1=xmean + margin, angle=90, code=3, length=0.05,
lwd= 1, lty= 1, col= "blue")
# We check if the variance of the Beta regression is a function of density of bugs
Dens_Var <- cbind(surviving_fema_3w_g$Density, Var_y)
Dens_Var
pp <- lm(Var_y ~ surviving_fema_3w_g$Density)
summary(pp) plot(Var_y ~
surviving_fema_3w_g$Density, pch=16) abline(pp)
beta_res[i,1] <- INT
beta_res[i,2] <- REG
beta_res[i,3] <- PHI
beta_res[i,4] <- LL
beta_res[i,5] <- DOF
beta_res[i,6] <- R_2
beta_res[i,7] <- p_val_Dens
} # End of the "if" for i=3 (Fem/Week3)
if (i == 4) {
############################################
###### Proportion females that survived in 3Weeks #####
############################################
beta_reg_prop_surv_fem_3Weeks <- betareg(prop_surv_3weeks ~ Density, data =
surviving_fema_3w_g)
summary(beta_reg_prop_surv_fem_3Weeks)
#########################################################
##
##### Extract from the beta regression results the parameters of interest #####
#########################################################
```

```
### thereg <- beta_reg_prop_surv_fem_3Weeks
# For the Intercept
INT       <-       thereg[[1]]$mean[1]
names(INT) <-NULL
INT
#  For  the  regressor  (Density)
REG  <-  thereg[[1]]$mean[2]
names(REG) <-NULL
REG
# For the precision  (Phi) PHI
<-  thereg[[1]]$precision[1]
names(PHI) <- NULL
PHI
#  The  predicted  values  (mu)
PRED       <-       thereg[[3]]
names(PRED) <- NULL
PRED
# Maximum-likelihood
LL <- thereg[[5]]$value
LL
# Degrees of freedom
DOF <- thereg[[14]]
DOF
# Pseudo R-squared
R_2 <- thereg[[20]]
R_2
# p-value of the regressor "Density"
p_val_Dens    <-    summary(thereg)$coefficients$mean[2,4]
p_val_Dens
################################################################
######## ###
####### Calculate the predicted values using the expected proportion E(y)=μ that #######
##### can be computed by applying the inverse link function to the linear predictor ######
################################################################
########
###
mu
<-
```

```
PRED
mu
phi  <-
PHI
phi
# Prediction of the variance of the predicted proportion fed
Var_y <- mu * (1 - mu)/(1+phi)
Var_y
sd_y <- sqrt(Var_y)
sd_y
plot(prop_surv_3weeks ~ Density, data = surviving_fema_3w_g, pch=16, ylim =c(0,0.1), ylab= " Mean
proportion adults that died in 3 weeks", xlab= " Density (number of adults/hamster)")
lines(mu ~ Density, data = surviving_fema_3w_g)
# Confidence Interval = x+tn-1, 1-α/2*(s/√n)
n <- 5
s <- sd_y
xmean <- mu
margin      <-      qt(0.975,df=n-1)*s/sqrt(n)
lowerinterval  <-  xmean  -  margin  upperinterval
<- xmean + margin
for  (j  in  1:n)  if(lowerinterval[j]  <0)  lowerinterval[j]  <-  0.01
lowerinterval xmean
upperinterval
par(mar   =   c(5.1,   6,   4.1,   2.1))   plot.new()   plot(prop_surv_3weeks   ~   Density,   data   =
surviving_fema_3w_g, pch=16, ylim =c(0,0.1), col= " red", ylab= "Mean proportion adults that died
in 3 weeks", xlab= " Density
(number     of     adults/hamster)")     lines(mu     ~     Density,     data     =
surviving_fema_3w_g,          col=          "blue",          lwd=          3)
arrows(x0=surviving_fema_3w_g$Density,      y0=xmean      -      margin,
x1=surviving_fema_3w_g$Density, y1=xmean + margin, angle=90, code=3,
length=0.05, lwd= 1, lty= 1, col= "blue")
# We check if the variance of the Beta regression is a function of density of bugs
Dens_Var <- cbind(surviving_fema_3w_g$Density, Var_y)
Dens_Var
pp     <-     lm(Var_y     ~     surviving_fema_3w_g$Density)
summary(pp)
plot(Var_y     ~     surviving_fema_3w_g$Density,     pch=16)
abline(pp)
```

```
beta_res[i,1]       <-       INT
beta_res[i,2]       <-       REG
beta_res[i,3]       <-       PHI
beta_res[i,4]       <-       LL
beta_res[i,5]       <-       DOF
beta_res[i,6]       <-       R_2
beta_res[i,7] <- p_val_Dens
} # End of the "if" for i=4 (Fem/3Weeks) } # This is the end of
the  for  loop  of  i=  1:4  beta_res  write.csv(beta_res,
file="beta_res_weekly_surv_fem.csv")
########## End for  Prop Surv Adult females in the 3-weeks period ##########
#######################################
#####        Analysis        of        the        Proportion        Dead        #####
#######################################
position <- c(1:5, 47:53, 74:86, 91:103) feeding
<- dat[,position]
#  We  select  the  appropriate  variables  from  N5  and  Ad
names(feeding) position <- c(1:4, 10:12) feeding_freq <-
feeding[,position]
feeding_freq2  <-  feeding_freq[,-4]  #  Delete  Sex  feeding_n5_freq  <-
subset(feeding_freq2,  feeding_freq2$Stage  ==  5)  feeding_ad_freq  <-
subset(feeding_freq2, feeding_freq2$Stage == 6)
##### Adults #####
feeding_ad_freq2 <- feeding_ad_freq[complete.cases(feeding_ad_freq), ] # Delete rows with NA
# Covert percentages into proportions and group by density for adults
feeding_ad_freq3_0      <-      feeding_ad_freq2      %>%
   group_by(Density) %>%
   mutate(Prop_feeding_once_in_3d  =  Pct_feeding_once_in_3d/100,  Prop_feeding_twice_in_3d  =
   Pct_feeding_twice_in_3d/100,
   Prop_feeding_thrice_in_3d  =  Pct_feeding_thrice_in_3d/100)  feeding_ad_freq3_0  <-
as.data.frame(feeding_ad_freq3_0) feeding_ad_freq3_0
#  Calculate  the  means  among  replicates  feeding_ad_freq3  <-
feeding_ad_freq3_0 %>% group_by(Density) %>%
   summarise(Prop_feeding_once_in_3d = mean(Prop_feeding_once_in_3d),
              Prop_feeding_twice_in_3d            =            mean(Prop_feeding_twice_in_3d),
              Prop_feeding_thrice_in_3d = mean(Prop_feeding_thrice_in_3d))
feeding_ad_freq3
# NOTE: Beta regression can be applied opnly for those bugs that fed once and twice
```

```
# because for bugs that fed thrice in three days there are many zeroes (not permitted)
##### Stage 5 nymphs #####
feeding_n5_freq2 <- feeding_n5_freq[complete.cases(feeding_n5_freq), ] # Delete rows with NA
# Convert percentages into proportions and group by density for n5ults
feeding_n5_freq3_0 <- feeding_n5_freq2 %>% group_by(Density) %>%
  mutate(Prop_feeding_once_in_3d = Pct_feeding_once_in_3d/100,
  Prop_feeding_twice_in_3d = Pct_feeding_twice_in_3d/100,
  Prop_feeding_thrice_in_3d = Pct_feeding_thrice_in_3d/100) feeding_n5_freq3_0 <-
as.data.frame(feeding_n5_freq3_0) feeding_n5_freq3_0
# We calculate the means among replicates
feeding_n5_freq3 <- feeding_n5_freq3_0 %>%
group_by(Density) %>%
  summarise(Prop_feeding_once_in_3d = mean(Prop_feeding_once_in_3d),
            Prop_feeding_twice_in_3d = mean(Prop_feeding_twice_in_3d),
            Prop_feeding_thrice_in_3d = mean(Prop_feeding_thrice_in_3d))
feeding_n5_freq3
# NOTE: Beta regression can be applied opnly for those bugs that fed once and twice
# because for bugs that fed thrice in three days there are many zeroes (not permitted)
#######################################################
##### Create a matrix to hold the various beta regression results #####
#######################################################
# NOTE: Here the 4 cases of the beta_res matrix correspond to the following beta regressions
#    For N5: Prop_feeding_once_in_3d and Prop_feeding_twice_in_3d #    For    Ad:
Prop_feeding_once_in_3d and Prop_feeding_twice_in_3d
beta_res <- data.frame(Intcpt = rep(0,4), Density = rep(0,4),Phi = rep(0,4), LL = rep(0,4),
DoF = rep(0,4), Ps_R2 = rep(0,4), p_val_Dens = rep(0,4))
str(beta_res)
for (i in
  1:4) {
  if(i ==
  1) {
################################################
#####  Proportion  Stage  5  nymphs  that  fed  once  in  3  days  ####
################################################
beta_reg_prop_fed_once <- betareg(Prop_feeding_once_in_3d ~ Density, data = feeding_n5_freq3)
summary(beta_reg_prop_fed_once)
############################################################
##
```

```
#####   Extract  from  the  beta  regression  results  the  parameters  of  interest  #####
##############################################################
### thereg <- beta_reg_prop_fed_once
# For the Intercept
INT <- thereg[[1]]$mean[1]; names(INT) <-NULL
# For the regressor (Density)
REG <- thereg[[1]]$mean[2]; names(REG) <-NULL
# For the precision  (Phi)
PHI <- thereg[[1]]$precision[1]; names(PHI) <- NULL
# The predicted values (mu)
PRED <- thereg[[3]]; names(PRED) <- NULL
# Maximum-likelihood
LL <- thereg[[5]]$value; LL
# Degrees of freedom
DOF <- thereg[[14]]; DOF
# Pseudo R-squared
R_2 <- thereg[[20]]; R_2
# p-value of the regressor "Density"
p_val_Dens <- summary(thereg)$coefficients$mean[2,4]; p_val_Dens
###############################################################
######## ###
####### Calculate the predicted values using the expected proportion E(y)=μ that #######
##### can be computed by applying the inverse link function to the linear predictor ######
##### Similarly we can also compute the variance to graph the confidence intervals ######
###############################################################
########
###
mu
<-
PRED
phi <- PHI
# Prediction of the variance of the predicted proportion fed
Var_y <- mu * (1 - mu)/(1+phi)
sd_y <- sqrt(Var_y)
plot(Prop_feeding_once_in_3d ~ Density, data = feeding_n5_freq3, pch=16, ylim =c(0,1), ylab=
"Mean proportion of stage 5 nymphs\nthat fed only once in a three-day period", xlab= " Density
(number of stage 5 nymphs/hamster)") lines(mu ~ Density, data = feeding_n5_freq3)
# Confidence Interval = x+tn-1, 1-α/2*(s/√n)
```

```
n <- 5; s <- sd_y; xmean <- mu margin <-
qt(0.975,df=n-1)*s/sqrt(n) lowerinterval <-
xmean - margin upperinterval <- xmean +
margin
for (j in 1:n) if(lowerinterval[j] <0) lowerinterval[j] <- 0.01
lowerinterval xmean
upperinterval
par(mar = c(5.1, 6, 4.1, 2.1))
plot.new()
plot(Prop_feeding_once_in_3d ~ Density, data = feeding_n5_freq3, pch=16, ylim =c(0,1), col= " red",
ylab= "Mean proportion of stage 5 nymphs\nthat fed only once in a three-day period", xlab= " Density
(number of stage 5 nymphs/hamster)") lines(mu ~ Density, data = feeding_n5_freq3, col= "blue",
lwd= 3) arrows(x0=feeding_n5_freq3$Density, y0=xmean - margin, x1=feeding_n5_freq3$Density,
y1=xmean + margin, angle=90, code=3, length=0.05, lwd= 1, lty= 1, col= "blue")
# We check if the variance of the Beta regression is a function of density of bugs
Dens_Var <- cbind(feeding_n5_freq3$Density, Var_y)
Dens_Var
pp <- lm(Var_y ~ feeding_n5_freq3$Density) summary(pp)
plot(Var_y ~ feeding_n5_freq3$Density, pch=16) abline(pp)
  } # End of combination 1 (n5 fed once in 3 days)
beta_res[i,1]    <-    INT
beta_res[i,2]    <-    REG
beta_res[i,3]    <-    PHI
beta_res[i,4]    <-    LL
beta_res[i,5]    <-    DOF
beta_res[i,6]    <-    R_2
beta_res[i,7] <- p_val_Dens
  if(i == 2) {
##############################################
####  Proportion  Stage  5  nymphs  that  fed  twice  in  3  days  ####
##############################################
beta_reg_prop_fed_twice <- betareg(Prop_feeding_twice_in_3d ~ Density, data = feeding_n5_freq3)
summary(beta_reg_prop_fed_twice)
##########################################################
##
#####  Extract  from  the  beta  regression  results  the  parameters  of  interest  #####
#########################################################
### thereg <- beta_reg_prop_fed_twice
```

```
# For the Intercept
INT <- thereg[[1]]$mean[1]; names(INT) <-NULL
# For the regressor (Density)
REG <- thereg[[1]]$mean[2]; names(REG) <-NULL
# For the precision  (Phi)
PHI <- thereg[[1]]$precision[1]; names(PHI) <- NULL
# The predicted values (mu)
PRED <- thereg[[3]]; names(PRED) <- NULL
# Maximum-likelihood
LL <- thereg[[5]]$value; LL
# Degrees of freedom
DOF <- thereg[[14]]; DOF
# Pseudo R-squared
R_2 <- thereg[[20]]; R_2
# p-value of the regressor "Density"
p_val_Dens <- summary(thereg)$coefficients$mean[2,4]; p_val_Dens
##################################################################
######## ###
####### Calculate the predicted values using the expected proportion E(y)=μ that #######
##### can be computed by applying the inverse link function to the linear predictor ######
##### Similarly we can also compute the variance to graph the confidence intervals ######
##################################################################
########
###
mu
<-
PRED
phi <- PHI
# Prediction of the variance of the predicted proportion fed
Var_y <- mu * (1 - mu)/(1+phi)
sd_y          <-          sqrt(Var_y)
plot(Prop_feeding_twice_in_3d    ~
Density, data = feeding_n5_freq3,
pch=16, ylim =c(0,0.5
), ylab= "Mean proportion of stage 5 nymphs\nthat fed twice in a three-day period", xlab
= " Density (number of stage 5 nymphs/hamster)") lines(mu ~
Density, data = feeding_n5_freq3)
# Confidence Interval = x+tn-1, 1-α/2*(s/√n)
```

```
n <- 5; s <- sd_y; xmean <- mu margin <-
qt(0.975,df=n-1)*s/sqrt(n) lowerinterval <-
xmean - margin upperinterval <- xmean +
margin
for (j in 1:n) if(lowerinterval[j] <0) lowerinterval[j] <- 0.01
lowerinterval xmean
upperinterval
par(mar = c(5.1, 6, 4.1, 2.1)) plot.new() plot(Prop_feeding_twice_in_3d ~ Density, data =
feeding_n5_freq3, pch=16, ylim =c(0,0.5 ), col= " red", ylab= "Mean proportion of stage 5
nymphs\nthat fed twice in a three-day period", xlab= " Density (number of stage 5 nymphs/hamster)")
lines(mu ~ Density, data = feeding_n5_freq3, col= "blue", lwd= 3)
arrows(x0=feeding_n5_freq3$Density, y0=xmean - margin, x1=feeding_n5_freq3$Density, y1=xmean
+ margin, angle=90, code=3, length=0.05, lwd= 1, lty= 1, col= "blue")
# We check if the variance of the Beta regression is a function of density of bugs
Dens_Var <- cbind(feeding_n5_freq3$Density, Var_y)
Dens_Var
pp <- lm(Var_y ~ feeding_n5_freq3$Density) summary(pp)
plot(Var_y ~ feeding_n5_freq3$Density, pch=16) abline(pp)
  } # End of combination 2 (n5 fed twice in 3 days)
beta_res[i,1] <- INT
beta_res[i,2] <- REG
beta_res[i,3] <- PHI
beta_res[i,4] <- LL
beta_res[i,5] <- DOF
beta_res[i,6] <- R_2
beta_res[i,7] <- p_val_Dens
if(i == 3) {
##########################################
##### Proportion adults that fed once in 3 days ####
##########################################
beta_reg_prop_fed_once <- betareg(Prop_feeding_once_in_3d ~ Density, data = feeding_ad_freq3)
summary(beta_reg_prop_fed_once)
##############################################################
##
##### Extract from the beta regression results the parameters of interest #####
#############################################################
### thereg <- beta_reg_prop_fed_once
# For the Intercept
```

```
INT <- thereg[[1]]$mean[1]; names(INT) <-NULL
# For the regressor (Density)
REG <- thereg[[1]]$mean[2]; names(REG) <-NULL
# For the precision  (Phi)
PHI <- thereg[[1]]$precision[1]; names(PHI) <- NULL
# The predicted values (mu)
PRED <- thereg[[3]]; names(PRED) <- NULL
# Maximum-likelihood
LL <- thereg[[5]]$value; LL
# Degrees of freedom
DOF <- thereg[[14]]; DOF
# Pseudo R-squared
R_2 <- thereg[[20]]; R_2
# p-value of the regressor "Density"
p_val_Dens <- summary(thereg)$coefficients$mean[2,4]; p_val_Dens
#################################################################
######## ###
####### Calculate the predicted values using the expected proportion E(y)=μ that #######
##### can be computed by applying the inverse link function to the linear predictor ######
##### Similarly we can also compute the variance to graph the confidence intervals ######
#################################################################
########
###
mu
<-
PRED
phi <- PHI
# Prediction of the variance of the predicted proportion fed
Var_y <- mu * (1 - mu)/(1+phi)
sd_y <- sqrt(Var_y)
plot(Prop_feeding_once_in_3d ~ Density, data = feeding_ad_freq3, pch=16, ylim =c(0,1), ylab= "Mean
proportion of adults\nthat fed only once in a three-day period", xlab= "
Density (number of stage 5 nymphs/hamster)") lines(mu ~
Density, data = feeding_ad_freq3)
# Confidence Interval = x+tn-1, 1-α/2*(s/√n)
n <- 5; s <- sd_y; xmean <- mu margin <-
qt(0.975,df=n-1)*s/sqrt(n) lowerinterval <-
xmean - margin upperinterval <- xmean +
```

```
margin
for (j in 1:n) if(lowerinterval[j] <0) lowerinterval[j] <- 0.01
lowerinterval xmean
upperinterval
par(mar = c(5.1, 6, 4.1, 2.1)) plot.new() plot(Prop_feeding_once_in_3d ~ Density, data =
feeding_ad_freq3, pch=16, ylim =c(0,1), col= " red", ylab= "Mean proportion of adults\nthat fed only
once in a three-day period", xlab= " Density (number of stage 5 nymphs/hamster)") lines(mu ~
Density, data = feeding_ad_freq3, col= "blue", lwd= 3) arrows(x0=feeding_ad_freq3$Density,
y0=xmean - margin, x1=feeding_ad_freq3$Density, y1=xmean + margin, angle=90, code=3,
length=0.05, lwd= 1, lty= 1, col= "blue")
# We check if the variance of the Beta regression is a function of density of bugs
Dens_Var <- cbind(feeding_ad_freq3$Density, Var_y)
Dens_Var
pp <- lm(Var_y ~ feeding_ad_freq3$Density) summary(pp)
plot(Var_y ~ feeding_ad_freq3$Density, pch=16) abline(pp)
  } # End of combination 3 (adults fed once in 3 days)
beta_res[i,1]     <-     INT
beta_res[i,2]     <-     REG
beta_res[i,3]     <-     PHI
beta_res[i,4]     <-     LL
beta_res[i,5]     <-     DOF
beta_res[i,6]     <-     R_2
beta_res[i,7] <- p_val_Dens
if(i == 4) {
##########################################
####     Proportion     adults     that     fed     twice     in     3     days     ####
##########################################
beta_reg_prop_fed_twice <- betareg(Prop_feeding_twice_in_3d ~ Density, data = feeding_ad_freq3)
summary(beta_reg_prop_fed_twice)
################################################################
##
#####   Extract   from   the   beta   regression   results   the   parameters   of   interest   #####
###############################################################
### thereg <- beta_reg_prop_fed_twice
# For the Intercept
INT <- thereg[[1]]$mean[1]; names(INT) <-NULL
# For the regressor (Density)
REG <- thereg[[1]]$mean[2]; names(REG) <-NULL
```

```
# For the precision  (Phi)
PHI <- thereg[[1]]$precision[1]; names(PHI) <- NULL
# The predicted values (mu)
PRED <- thereg[[3]]; names(PRED) <- NULL
# Maximum-likelihood
LL <- thereg[[5]]$value; LL
# Degrees of freedom
DOF <- thereg[[14]]; DOF
# Pseudo R-squared
R_2 <- thereg[[20]]; R_2
# p-value of the regressor "Density"
p_val_Dens <- summary(thereg)$coefficients$mean[2,4]; p_val_Dens
##############################################################
######## ###
####### Calculate the predicted values using the expected proportion E(y)=μ that #######
##### can be computed by applying the inverse link function to the linear predictor ######
##### Similarly we can also compute the variance to graph the confidence intervals ######
##############################################################
########
###
mu
<-
PRED
phi <- PHI
# Prediction of the variance of the predicted proportion fed
Var_y <- mu * (1 - mu)/(1+phi)
sd_y <- sqrt(Var_y)
plot(Prop_feeding_twice_in_3d ~ Density, data = feeding_ad_freq3, pch=16, ylim =c(0,0.5
), ylab= "Mean proportion of stage 5 nymphs\nthat fed twice in a three-day period", xlab
= " Density (number of stage 5 nymphs/hamster)") lines(mu ~
Density, data = feeding_ad_freq3)
# Confidence Interval = x+tn-1, 1-α/2*(s/√n)
n <- 5; s <- sd_y; xmean <- mu margin <-
qt(0.975,df=n-1)*s/sqrt(n) lowerinterval <-
xmean - margin upperinterval <- xmean +
margin
for (j in 1:n) if(lowerinterval[j] <0) lowerinterval[j] <- 0.01
lowerinterval xmean
```

```
upperinterval
par(mar = c(5.1, 6, 4.1, 2.1)) plot.new() plot(Prop_feeding_twice_in_3d ~ Density, data = feeding_ad_freq3, pch=16, ylim =c(0,0.5 ), col= " red", ylab= "Mean proportion of stage 5 nymphs\nthat fed twice in a three-day period", xlab= " Density (number of stage 5 nymphs/hamster)")
lines(mu ~ Density, data = feeding_ad_freq3, col= "blue", lwd= 3)
arrows(x0=feeding_ad_freq3$Density, y0=xmean - margin, x1=feeding_ad_freq3$Density, y1=xmean + margin, angle=90, code=3, length=0.05, lwd= 1, lty= 1, col= "blue")
# We check if the variance of the Beta regression is a function of density of bugs
Dens_Var <- cbind(feeding_ad_freq3$Density, Var_y)
Dens_Var
pp <- lm(Var_y ~ feeding_ad_freq3$Density) summary(pp)
plot(Var_y ~ feeding_ad_freq3$Density, pch=16) abline(pp)
beta_res[i,1]     <-     INT
beta_res[i,2]     <-     REG
beta_res[i,3]     <-     PHI
beta_res[i,4]     <-     LL
beta_res[i,5]     <-     DOF
beta_res[i,6]     <-     R_2
beta_res[i,7] <- p_val_Dens
  } # End of combination 2 (n5 fed twice in 3 days)
} # End of loop for the four combinations
write.csv(beta_res, file="beta_res_fed_once_twice.csv")
#######%%%%%%%%%#########%%%%%%%%%#########%%%%%%%%%######
##%% %%%%%%%########
#############################################
##### Percent mortality in the three-day trials #####
#############################################
###################################
##### Beta regressions only for N5 ####
#####        With        graphs        with        predicted        #####
###################################
names(mortality)
#  [1] "Density"          "Replicate"          "Stage"             "Sex"
#  [5] "Irritability"       "N_dead_D1"          "N_dead_D2"          "N_dead_D3"
#  [9] "N_dead_3Days"       "Prop_dead_D1"       "Prop_dead_D2"       "Prop_dead_D3"
# [13] "Mean_prop_dead_3Days" "Sd_prop_dead_3Days"
dat_N5_prop_dead <- subset(mortality, mortality$Stage == 5) dat_N5_prop_dead
dat_AD_prop_dead <- subset(mortality, mortality$Stage == 6) dat_AD_prop_dead
```

```
position <- c(1,2,5,10:12)
dat_N5_prop_dead2 <- dat_N5_prop_dead[1:18,position] dat_N5_prop_dead2
dat_N5_prop_dead3 <- dat_N5_prop_dead2 %>% mutate(prop_dead_3Days = 1-((1-
    Prop_dead_D1)*(1-Prop_dead_D2)*(1-
    Prop_dead_D3))) dat_N5_prop_dead3
dat_N5_prop_dead3b <- dat_N5_prop_dead3 %>%
    group_by(Density) %>%
    summarise_at(vars(Prop_dead_D1,Prop_dead_D2,Prop_dead_
    D3,prop_dead_3Days), funs(mean))
dat_N5_prop_dead3b <- as.data.frame(dat_N5_prop_dead3b) dat_N5_prop_dead3b
#$$$$$$$$$$$$$$$$$$$$$$$$$$$$$$$$$$$$$$$$$$$$$$$$$$$$$$$$$$$$$$$$$$$$$$$$$ $$$
############################################################
##### Create a matrix to hold the various beta regression results #####
############################################################
# NOTE: Here the 6 cases of the beta_res matrix correspond to the following beta regressions
#                   N5_Day1, N5_Day2, N5_Day3, AD_Day1, AD_Day2, AD_Day3
beta_res <- data.frame(Intcpt = rep(0,8), Density = rep(0,8),Phi = rep(0,8), LL = rep(0,8),
DoF = rep(0,8), Ps_R2 = rep(0,8), p_val_Dens = rep(0,8))
str(beta_res)
for (i in
    1:8) {
    if(i ==
    1) {
#################################################
##### Proportion Stage 5 nymphs that died on Day 1 ####
#################################################
beta_reg_prop_dead_N5_Day1 <- betareg(Prop_dead_D1 ~ Density, data = dat_N5_prop_dead3b)
summary(beta_reg_prop_dead_N5_Day1)
#&&&&&&&&&&&&&&&&&&&&&&&&&&&&&&&&&&&&&&&&&&&&&&&&&&&& &&
#################################################################
##
##### Extract from the beta regression results the parameters of interest #####
#################################################################
##
thereg <- beta_reg_prop_dead_N5_Day1
# For the Intercept
INT <- thereg[[1]]$mean[1]
names(INT) <-NULL
```

```
INT
#  For  the  regressor  (Density)
REG  <-  thereg[[1]]$mean[2]
names(REG) <-NULL
REG
# For the precision  (Phi)
PHI <- thereg[[1]]$precision[1]
names(PHI) <- NULL
PHI
#  The  predicted  values  (mu)
PRED        <-        thereg[[3]]
names(PRED) <- NULL
PRED
# Maximum-likelihood
LL <- thereg[[5]]$value
LL
# Degrees of freedom
DOF <- thereg[[14]]
DOF
# Pseudo R-squared
R_2 <- thereg[[20]]
R_2
# p-value of the regressor "Density"
p_val_Dens    <-    summary(thereg)$coefficients$mean[2,4]
p_val_Dens
#&&&&&&&&&&&&&&&&&&&&&&&&&&&&&&&&&&&&&&&&&&&&&&&&&&&&&& &&
##################################################################
######## ###
####### Calculate the predicted values using the expected proportion E(y)=μ that
#######
##### can be computed by applying the inverse link function to the linear predictor ######
##### Similarly we can also compute the variance to graph the confidence intervals ######
##################################################################
########
###
mu
<-
PRED
```

```
mu
phi  <-
PHI
phi
# Prediction of the variance of the predicted proportion fed
Var_y <- mu * (1 - mu)/(1+phi)
Var_y
sd_y <- sqrt(Var_y)
sd_y
plot(Prop_dead_D1 ~ Density, data = dat_N5_prop_dead3b, pch=16, ylim =c(0,0.5), ylab= " Mean proportion of stage 5 nymphs\nthat died on day 1", xlab= " Density (number of stage 5 nymphs/hamster)")
lines(mu ~ Density, data = dat_N5_prop_dead3b)
# Confidence Interval = x+tn-1, 1-α/2*(s/√n) n
<- 5
s <- sd_y
xmean <- mu
margin <- qt(0.975,df=n-1)*s/sqrt(n)
lowerinterval <- xmean - margin upperinterval
<- xmean + margin
for (j in 1:n) if(lowerinterval[j] <0) lowerinterval[j] <- 0.01
for (j in 1:n) if(upperinterval[j] >1) upperinterval[j] <- 0.99
lowerinterval xmean
upperinterval
par(mar = c(5.1, 6, 4.1, 2.1))
plot.new()
plot(Prop_dead_D1 ~ Density, data = dat_N5_prop_dead3b, pch=16, ylim =c(0,0.3), col=
" red", ylab= "Mean proportion of stage 5 nymphs\nthat died on day 1", xlab= " Density (number of stage 5 nymphs/hamster)")
# Beta regression lines(mu ~ Density, data = dat_N5_prop_dead3b, col=
"blue", lwd= 3) arrows(x0=dat_N5_prop_dead3b$Density, y0=xmean -
margin, x1=dat_N5_prop_dead3b$Density, y1=xmean + margin, angle=90,
code=3, length=0.05, lwd= 1, lty= 1, col= "blue")
# NOTE: The confidence interval lines are the ones corresponding to the predictions of the beta regression
# We check if the variance of the Beta regression is a function of density of bugs
Dens_Var <- cbind(dat_N5_prop_dead3b$Density, Var_y)
Dens_Var
pp <- lm(Var_y ~ dat_N5_prop_dead3b$Density)
```

```
summary(pp)
plot(Var_y     ~     dat_N5_prop_dead3b$Density,     pch=16)
abline(pp)
#################################################################
########
########
beta_res[i,1]     <-     INT
beta_res[i,2]     <-     REG
beta_res[i,3]     <-     PHI
beta_res[i,4]     <-     LL
beta_res[i,5]     <-     DOF
beta_res[i,6]     <-     R_2
beta_res[i,7] <- p_val_Dens
} # End of the "if" for i=1 (N5/Day1)
if (i == 2) {
#################################################
#####     Proportion     Stage     5     nymphs     that     died     on     day     2     #####
#################################################
beta_reg_prop_dead_N5_Day2 <- betareg(Prop_dead_D2 ~ Density, data = dat_N5_prop_dead3b)
summary(beta_reg_prop_dead_N5_Day2)
#&&&&&&&&&&&&&&&&&&&&&&&&&&&&&&&&&&&&&&&&&&&&&&&&&&&& &&
#################################################################
##
#####   Extract   from   the   beta   regression   results   the   parameters   of   interest   #####
################################################################
### thereg <- beta_reg_prop_dead_N5_Day2
# For the Intercept
INT     <-     thereg[[1]]$mean[1]
names(INT) <-NULL
INT
#  For  the  regressor  (Density)
REG  <-  thereg[[1]]$mean[2]
names(REG) <-NULL
REG
# For the precision  (Phi)
PHI <- thereg[[1]]$precision[1]
names(PHI) <- NULL
PHI
```

```
#  The  predicted  values  (mu)
PRED        <-        thereg[[3]]
names(PRED) <- NULL
PRED
# Maximum-likelihood
LL <- thereg[[5]]$value
LL
# Degrees of freedom
DOF <- thereg[[14]]
DOF
# Pseudo R-squared
R_2 <- thereg[[20]]
R_2
# p-value of the regressor "Density"
p_val_Dens <- summary(thereg)$coefficients$mean[2,4]
p_val_Dens
#&&&&&&&&&&&&&&&&&&&&&&&&&&&&&&&&&&&&&&&&&&&&&&&&&&&&& &&
##################################################################
######## ###
####### Calculate the predicted values using the expected proportion E(y)=μ that #######
##### can be computed by applying the inverse link function to the linear predictor ######
##### Similarly we can also compute the variance to graph the confidence intervals ######
##################################################################
########
###
mu      <-
PRED mu
phi  <-
PHI
phi
# Prediction of the variance of the predicted proportion fed
Var_y <- mu * (1 - mu)/(1+phi)
Var_y
sd_y <- sqrt(Var_y)
# Confidence Interval = x+tn-1, 1-α/2*(s/√n)
n <- 5
s <- sd_y
xmean <- mu
```

```
margin <- qt(0.975,df=n-1)*s/sqrt(n)
lowerinterval <- xmean - margin upperinterval
<- xmean + margin
for (j in 1:n) if(lowerinterval[j] <0) lowerinterval[j] <- 0.01
for (j in 1:n) if(upperinterval[j] >1) upperinterval[j] <- 0.99
lowerinterval xmean
upperinterval
par(mar = c(5.1, 6, 4.1, 2.1)) plot.new() plot(Prop_dead_D2 ~ Density, data =
dat_N5_prop_dead3b, pch=16, ylim =c(0,0.5), col= " red", ylab= "Mean proportion of stage 5
nymphs\nthat died on day 2", xlab= " Density (number of stage 5 nymphs/hamster)")
# Beta regression lines(mu ~ Density, data = dat_N5_prop_dead3b, col=
"blue", lwd= 2) arrows(x0=dat_N5_prop_dead3b$Density, y0=xmean -
margin, x1=dat_N5_prop_dead3b$Density, y1=xmean + margin, angle=90,
code=3, length=0.05, lwd= 1, lty= 1, col= "blue")
# NOTE: The confidence interval lines are the ones corresponding to the predictions of the beta regression
# We check if the variance of the Beta regression is a function of density of bugs
Dens_Var <- cbind(dat_N5_prop_dead3b$Density, Var_y)
Dens_Var
pp <- lm(Var_y ~ dat_N5_prop_dead3b$Density)
summary(pp)
plot(Var_y ~ dat_N5_prop_dead3b$Density, pch=16)
abline(pp)
#################################################################
######## ########
##### NOTE: The bug density has a significant effect on the variance in the results of Day 2 #####
#################################################################
########
########
beta_res[i,1] <- INT
beta_res[i,2] <- REG
beta_res[i,3] <- PHI
beta_res[i,4] <- LL
beta_res[i,5] <- DOF
beta_res[i,6] <- R_2
beta_res[i,7] <- p_val_Dens
} # End of the "if" for i=2
(N5/Day2)
#$$$$$$$$$$$$$$$$$$$$$$$$$$$$$$$$$$$$$$$$$$$$$$$$$$$$$$$$$$$$$$$$
```

```
$$$
if (i
==
3)
{
###############################################
##### Proportion Stage 5 nymphs that died on day 3 #####
##########################################
##### for (j in 1:5) {
if(dat_N5_prop_dead3b$Prop_dead_D3[j] <=0) dat_N5_prop_dead3b$Prop_dead_D3[j] = 0.001
  }
beta_reg_prop_dead_N5_Day3 <- betareg(Prop_dead_D3 ~ Density, data = dat_N5_prop_dead3b)
summary(beta_reg_prop_dead_N5_Day3)
#&&&&&&&&&&&&&&&&&&&&&&&&&&&&&&&&&&&&&&&&&&&&&&&& &&
#############################################################
##
#####  Extract  from  the  beta  regression  results  the  parameters  of  interest  #####
############################################################
### thereg <- beta_reg_prop_dead_N5_Day3
# For the Intercept
INT      <-      thereg[[1]]$mean[1]
names(INT) <-NULL
INT
#  For  the  regressor  (Density)
REG  <-  thereg[[1]]$mean[2]
names(REG) <-NULL
REG
# For the precision  (Phi)
PHI      <-      thereg[[1]]$precision[1]
names(PHI) <- NULL
PHI
#  The  predicted  values  (mu)
PRED      <-      thereg[[3]]
names(PRED) <- NULL
PRED
# Maximum-likelihood
LL <- thereg[[5]]$value
LL
```

```
# Degrees of freedom
DOF <- thereg[[14]]
DOF
# Pseudo R-squared
R_2 <- thereg[[20]]
R_2
# p-value of the regressor "Density"
p_val_Dens     <-     summary(thereg)$coefficients$mean[2,4]
p_val_Dens
#&&&&&&&&&&&&&&&&&&&&&&&&&&&&&&&&&&&&&&&&&&&&&&&&&&&&&&&&& &&
###########################################################
######## ###
####### Calculate the predicted values using the expected proportion E(y)=μ that #######
##### can be computed by applying the inverse link function to the linear predictor ######
##### Similarly we can also compute the variance to graph the confidence intervals ######
###########################################################
########
###
mu      <-
PRED mu
phi  <-
PHI
phi
plot(Pr
op_de
ad_D3
~
Densit
y, data
=
dat_N
5_prop
_dead
3b,
pch=1
6, ylim
=c(0,0
.25),
```

```
ylab =
" Mean
propor
tion of
stage
5
nymph
s\nthat
died
on day
3",
xlab=
"
Densit
y
(numb
er of
stage
5
nymph
s/ham
ster)")
lines(mu ~ Density, data = dat_N5_prop_dead3b)
# Confidence Interval = x+tn-1, 1-α/2*(s/√n) n
<- 5
s <- sd_y
xmean <- mu
margin <- qt(0.975,df=n-1)*s/sqrt(n)
lowerinterval <- xmean - margin upperinterval
<- xmean + margin
for (j in 1:n) if(lowerinterval[j] <0) lowerinterval[j] <- 0.01
for (j in 1:n) if(upperinterval[j] >1) upperinterval[j] <- 0.99
lowerinterval xmean
upperinterval
par(mar = c(5.1, 6, 4.1, 2.1)) plot.new() plot(Prop_dead_D3 ~ Density, data =
dat_N5_prop_dead3b, pch=16, ylim =c(0,0.5), col= "red", ylab= "Mean proportion of stage 5
nymphs\nthat died on day 3", xlab= " Density (number of stage 5 nymphs/hamster)")
# Beta regression lines(mu ~ Density, data = dat_N5_prop_dead3b, col=
```

```
"blue",  lwd=  2)  arrows(x0=dat_N5_prop_dead3b$Density,  y0=xmean  -
margin, x1=dat_N5_prop_dead3b$Density, y1=xmean + margin, angle=90,
code=3, length=0.05, lwd= 1, lty= 1, col= "blue")
# NOTE: The confidence interval lines are the ones corresponding to the predictions of the beta regression
# We check if the variance of the Beta regression is a function of density of bugs
Dens_Var  <-  cbind(dat_N5_prop_dead3b$Density,  Var_y)
Dens_Var
pp    <-    lm(Var_y    ~    dat_N5_prop_dead3b$Density)
summary(pp)
plot(Var_y    ~    dat_N5_prop_dead3b$Density,    pch=16)
abline(pp)
beta_res[i,1]    <-    INT
beta_res[i,2]    <-    REG
beta_res[i,3]    <-    PHI
beta_res[i,4]    <-    LL
beta_res[i,5]    <-    DOF
beta_res[i,6]    <-    R_2
beta_res[i,7] <- p_val_Dens
} # End of the "if" for i=3 (N5/Day3)
#$$$$$$$$$$$$$$$$$$$$$$$$$$$$$$$$$$$$$$$$$$$$$$$$$$$$$$$$$$$$$$$$$$$$
$$$
if (i
==
4)
{
##################################################
#####    Proportion    Stage    5    nymphs    that    died    on    3Days    #####
##################################################
beta_reg_prop_dead_N5_3Days <- betareg(prop_dead_3Days ~ Density, data = dat_N5_prop_dead3b)
summary(beta_reg_prop_dead_N5_3Days)
#&&&&&&&&&&&&&&&&&&&&&&&&&&&&&&&&&&&&&&&&&&&&&&&&&&&& &&
#################################################################
##
#####  Extract  from  the  beta  regression  results  the  parameters  of  interest  #####
################################################################
### thereg <- beta_reg_prop_dead_N5_3Days
# For the Intercept
INT    <-    thereg[[1]]$mean[1]
```

```
names(INT) <-NULL
INT
# For the regressor (Density)
REG        <-        thereg[[1]]$mean[2]
names(REG) <-NULL
REG
# For the precision  (Phi)
PHI <- thereg[[1]]$precision[1]
names(PHI) <- NULL
PHI
#   The   predicted   values   (mu)
PRED         <-         thereg[[3]]
names(PRED) <- NULL
PRED
# Maximum-likelihood
LL <- thereg[[5]]$value
LL
# Degrees of freedom
DOF <- thereg[[14]]
DOF
# Pseudo R-squared
R_2 <- thereg[[20]]
R_2
# p-value of the regressor "Density"
p_val_Dens      <-      summary(thereg)$coefficients$mean[2,4]
p_val_Dens
#&&&&&&&&&&&&&&&&&&&&&&&&&&&&&&&&&&&&&&&&&&&&&&&&&&&&&&&&&&& &&
################################################################
######## ###
####### Calculate the predicted values using the expected proportion E(y)=μ that #######
##### can be computed by applying the inverse link function to the linear predictor ######
##### Similarly we can also compute the variance to graph the confidence intervals ######
################################################################
######## ###
mu       <-
PRED mu
phi   <-
PHI
```

```
phi
plot(prop_dead_3Days ~ Density, data = dat_N5_prop_dead3b, pch=16, ylim =c(0,0.25), ylab= " Mean proportion of stage 5 nymphs\nthat died on day 3", xlab= " Density
(number of stage 5 nymphs/hamster)") lines(mu ~
Density, data = dat_N5_prop_dead3b)
# Confidence Interval = x+tn-1, 1-α/2*(s/√n) n
<- 5
s <- sd_y
xmean <- mu
margin <- qt(0.975,df=n-1)*s/sqrt(n)
lowerinterval <- xmean - margin upperinterval
<- xmean + margin
for (j in 1:n) if(lowerinterval[j] <0) lowerinterval[j] <- 0.01
for (j in 1:n) if(upperinterval[j] >1) upperinterval[j] <- 0.99
lowerinterval xmean
upperinterval
par(mar = c(5.1, 6, 4.1, 2.1)) plot.new() plot(prop_dead_3Days ~ Density, data = dat_N5_prop_dead3b, pch=16, ylim =c(0,0.7), col = "red", ylab= "Mean proportion of stage 5 nymphs\nthat died in 3 days", xlab= " Density (number of stage 5 nymphs/hamster)")
# Beta regression lines(mu ~ Density, data = dat_N5_prop_dead3b, col=
"blue", lwd= 2) arrows(x0=dat_N5_prop_dead3b$Density, y0=xmean -
margin, x1=dat_N5_prop_dead3b$Density, y1=xmean + margin, angle=90,
code=3, length=0.05, lwd= 1, lty= 1, col= "blue")
# NOTE: The confidence interval lines are the ones corresponding to the predictions of the beta regression
# We check if the variance of the Beta regression is a function of density of bugs
Dens_Var <- cbind(dat_N5_prop_dead3b$Density, Var_y)
Dens_Var
pp <- lm(Var_y ~ dat_N5_prop_dead3b$Density)
summary(pp)
plot(Var_y ~ dat_N5_prop_dead3b$Density, pch=16)
abline(pp)
beta_res[i,1] <- INT
beta_res[i,2] <- REG
beta_res[i,3] <- PHI
beta_res[i,4] <- LL
beta_res[i,5] <- DOF
beta_res[i,6] <- R_2
beta_res[i,7] <- p_val_Dens
```

```
} # End of the "if" for i=4 (N5/3Days)
#$$$$$$$$$$$$$$$$$$$$$$$$$$$$$$$$$$$$$$$$$$$$$$$$$$$$$$$$$$$$$$$$$$ $$$
#####################################
#####     Start     with     the     analysis     of     Adults     ######
#####################################
position <- c(1,2,5,10:12)
dat_AD_prop_dead2 <- dat_AD_prop_dead[1:17,position] dat_AD_prop_dead2
dat_AD_prop_dead3 <- dat_AD_prop_dead2 %>% mutate(prop_dead_3Days = 1-((1-
    Prop_dead_D1)*(1-Prop_dead_D2)*(1-
    Prop_dead_D3)))          dat_AD_prop_dead3
dat_AD_prop_dead3b  <-  dat_AD_prop_dead3  %>%
group_by(Density) %>%
    summarise_at(vars(Prop_dead_D1,Prop_dead_D2,Prop_dead_D3,prop_dead_3Days), funs(mean))
dat_AD_prop_dead3b     <-     as.data.frame(dat_AD_prop_dead3b)
dat_AD_prop_dead3b if (i == 5) {
##########################################
######## Beta regressions only for Adults ########
#####     Proportion     of     adults     that     died     on     day     1     #####
##########################################
beta_reg_prop_feed_Ad  <-  betareg(Prop_dead_D1  ~  Density,  data  =
dat_AD_prop_dead3b) summary(beta_reg_prop_feed_Ad)
################################################################
##
#####  Extract  from  the  beta  regression  results  the  parameters  of  interest  #####
###############################################################
### thereg <- beta_reg_prop_feed_Ad
# For the Intercept
INT      <-      thereg[[1]]$mean[1]
names(INT) <-NULL
INT
#  For  the  regressor  (Density)
REG  <-  thereg[[1]]$mean[2]
names(REG) <-NULL
REG
# For the precision  (Phi)
PHI <- thereg[[1]]$precision[1]
names(PHI) <- NULL
PHI
```

```
#  The  predicted  values  (mu)
PRED       <-       thereg[[3]]
names(PRED) <- NULL
PRED
# Maximum-likelihood
LL <- thereg[[5]]$value
LL
# Degrees of freedom
DOF <- thereg[[14]]
DOF
# Pseudo R-squared
R_2 <- thereg[[20]]
R_2
# p-value of the regressor "Density"
p_val_Dens    <-    summary(thereg)$coefficients$mean[2,4]
p_val_Dens
#&&&&&&&&&&&&&&&&&&&&&&&&&&&&&&&&&&&&&&&&&&&&&&&&&&&
&&
mu      <-
PRED mu
phi  <-
PHI
phi
# Prediction of the variance of the predicted proportion fed
Var_y <- mu * (1 - mu)/(1+phi)
Var_y
sd_y <- sqrt(Var_y)
sd_y
plot(Prop_dead_D1 ~ Density, data = dat_AD_prop_dead3b, pch=16, ylim =c(0,1), ylab= "
Mean proportion of adults\nthat died on day 1", xlab= " Density (number of adults/hamster)")
lines(mu ~ Density, data = dat_N5_prop_dead3b)
# Confidence Interval = x+tn-1, 1-α/2*(s/√n) n
<- 5
s <- sd_y
xmean <- mu
margin  <-  qt(0.975,df=n-1)*s/sqrt(n)  lowerinterval
<- xmean - margin upperinterval <- xmean + margin
for  (j  in  1:n)  if(lowerinterval[j]  <0)  lowerinterval[j]  <-  0.01
```

```
lowerinterval xmean
upperinterval
par(mar = c(5.1, 6, 4.1, 2.1)) plot.new() plot(Prop_dead_D1 ~ Density, data =
dat_AD_prop_dead3b, pch=16, ylim =c(0,0.4), col= "red", ylab= "Mean proportion of adults\nthat
died on day 1", xlab= " Density (number of adults/hamster)") lines(mu ~ Density, data =
dat_AD_prop_dead3b, col= "blue", lwd= 2) arrows(x0=dat_AD_prop_dead3b$Density, y0=xmean -
margin, x1=dat_AD_prop_dead3b$Density, y1=xmean + margin, angle=90, code=3, length=0.05,
lwd= 1, lty= 1, col= "blue")
# We check if the variance of the Beta regression is a function of density of bugs
Dens_Var <- cbind(dat_AD_prop_dead3b$Density, Var_y)
Dens_Var
pp <- lm(Var_y ~ dat_AD_prop_dead3b$Density)
summary(pp)
plot(Var_y ~ dat_AD_prop_dead3b$Density, pch=16)
abline(pp)
beta_res[i,1] <- INT
beta_res[i,2] <- REG
beta_res[i,3] <- PHI
beta_res[i,4] <- LL
beta_res[i,5] <- DOF
beta_res[i,6] <- R_2
beta_res[i,7] <- p_val_Dens
} # End of the "if" for i=5 (AD/Day1)
#$$$$$$$$$$$$$$$$$$$$$$$$$$$$$$$$$$$$$$$$$$$$$$$$$$$$$$$$$$$$$$$
$$$
if (i == 6) {
########################################
##### Proportion Adults that died on Day 2 ####
########################################
beta_reg_prop_dead_AD_Day2 <- betareg(Prop_dead_D2 ~ Density, data = dat_AD_prop_dead3b)
summary(beta_reg_prop_dead_AD_Day2)
#&&&&&&&&&&&&&&&&&&&&&&&&&&&&&&&&&&&&&&&&&&&&&&&&& &&
################################################################
##
##### Extract from the beta regression results the parameters of interest #####
################################################################
##
thereg <- beta_reg_prop_dead_AD_Day2
```

```
# For the Intercept
INT        <-        thereg[[1]]$mean[1]
names(INT) <-NULL
INT
#  For  the  regressor  (Density)
REG  <-  thereg[[1]]$mean[2]
names(REG) <-NULL
REG
# For the precision  (Phi)
PHI <- thereg[[1]]$precision[1]
names(PHI) <- NULL
PHI
#  The  predicted  values  (mu)
PRED        <-        thereg[[3]]
names(PRED) <- NULL
PRED
# Maximum-likelihood
LL <- thereg[[5]]$value
LL
# Degrees of freedom
DOF <- thereg[[14]]
DOF
# Pseudo R-squared
R_2 <- thereg[[20]]
R_2
# p-value of the regressor "Density"
p_val_Dens     <-     summary(thereg)$coefficients$mean[2,4]
p_val_Dens
#&&&&&&&&&&&&&&&&&&&&&&&&&&&&&&&&&&&&&&&&&&&&&&&&&&& &&
#################################################################
######## ###
####### Calculate the predicted values using the expected proportion E(y)=μ that #######
##### can be computed by applying the inverse link function to the linear predictor ######
##### Similarly we can also compute the variance to graph the confidence intervals ######
#################################################################
########
###
mu
```

```
<-
PRED
mu
phi  <-
PHI
phi
# Prediction of the variance of the predicted proportion fed
Var_y <- mu * (1 - mu)/(1+phi)
Var_y
sd_y <- sqrt(Var_y)
sd_y
plot(Prop_dead_D2 ~ Density, data = dat_AD_prop_dead3b, pch=16, ylim =c(0,0.3), ylab= " Mean
proportion of Adults\nthat died on day 2", xlab= " Density (number of
Adults/hamster)")
lines(mu ~ Density, data = dat_AD_prop_dead3b)
# Confidence Interval = x+tn-1, 1-α/2*(s/√n) n
<- 5
s <- sd_y
xmean <- mu
margin <- qt(0.975,df=n-1)*s/sqrt(n)
lowerinterval <- xmean - margin upperinterval
<- xmean + margin
for (j in 1:n) if(lowerinterval[j] <0) lowerinterval[j] <- 0.01
lowerinterval xmean
upperinterval
par(mar = c(5.1, 6, 4.1, 2.1)) plot.new() plot(Prop_dead_D2 ~ Density, data =
dat_AD_prop_dead3b, pch=16, ylim =c(0,0.3), col= " red", ylab= "Mean proportion of Adults\nthat
died on day 2", xlab= " Density (number of Adults/hamster)") # Beta regression lines(mu ~ Density,
data = dat_AD_prop_dead3b, col= "blue", lwd= 3) arrows(x0=dat_AD_prop_dead3b$Density,
y0=xmean - margin, x1=dat_AD_prop_dead3b$Density, y1=xmean + margin, angle=90, code=3,
length=0.05, lwd= 1, lty= 1, col= "blue")
# NOTE: The confidence interval lines are the ones corresponding to the predictions of the beta regression
# We check if the variance of the Beta regression is a function of density of bugs
Dens_Var <- cbind(dat_AD_prop_dead3b$Density, Var_y)
Dens_Var
pp <- lm(Var_y ~ dat_AD_prop_dead3b$Density)
summary(pp)
plot(Var_y ~ dat_AD_prop_dead3b$Density, pch=16)
```

```
abline(pp)
#################################################################
######## ########
##### NOTE: The bug density has a significant effect on the variance in the results of Day 1 #####
#################################################################
########
########
beta_res[i,1]      <-      INT
beta_res[i,2]      <-      REG
beta_res[i,3]      <-      PHI
beta_res[i,4]      <-      LL
beta_res[i,5]      <-      DOF
beta_res[i,6]      <-      R_2
beta_res[i,7] <- p_val_Dens
} # End of the "if" for i=6 (AD/Day2)
if (i == 7) {
##########################################
#####       Proportion       Adults       that       died       on       day       3       #####
##########################################
beta_reg_prop_dead_AD_Day3 <- betareg(Prop_dead_D3 ~ Density, data = dat_AD_prop_dead3b)
summary(beta_reg_prop_dead_AD_Day3)
#&&&&&&&&&&&&&&&&&&&&&&&&&&&&&&&&&&&&&&&&&&&&&&&&&& &&
#################################################################
##
#####   Extract   from   the   beta   regression   results   the   parameters   of   interest   #####
################################################################
### thereg <- beta_reg_prop_dead_AD_Day3
# For the Intercept
INT      <-      thereg[[1]]$mean[1]
names(INT) <-NULL
INT
#  For  the  regressor  (Density)
REG  <-  thereg[[1]]$mean[2]
names(REG) <-NULL
REG
# For the precision  (Phi)
PHI <- thereg[[1]]$precision[1]
names(PHI) <- NULL
```

```
PHI
#  The  predicted  values  (mu)
PRED        <-        thereg[[3]]
names(PRED) <- NULL
PRED
# Maximum-likelihood
LL <- thereg[[5]]$value
LL
# Degrees of freedom
DOF <- thereg[[14]]
DOF
# Pseudo R-squared
R_2 <- thereg[[20]]
R_2
# p-value of the regressor "Density"
p_val_Dens     <-     summary(thereg)$coefficients$mean[2,4]
p_val_Dens
#&&&&&&&&&&&&&&&&&&&&&&&&&&&&&&&&&&&&&&&&&&&&&&&&&&&&& &&
##################################################################
######## ###
####### Calculate the predicted values using the expected proportion E(y)=μ that #######
##### can be computed by applying the inverse link function to the linear predictor ######
##### Similarly we can also compute the variance to graph the confidence intervals ######
##################################################################
########
###
mu
<-
PRED
mu
phi  <-
PHI
phi
# Prediction of the variance of the predicted proportion fed
Var_y <- mu * (1 - mu)/(1+phi)
Var_y
sd_y <- sqrt(Var_y)
# Confidence Interval = x+tn-1, 1-α/2*(s/√n)
```

```
n <- 5
s <- sd_y
xmean <- mu
margin <- qt(0.975,df=n-1)*s/sqrt(n)
lowerinterval <- xmean - margin upperinterval
<- xmean + margin
for (j in 1:n) if(lowerinterval[j] <0) lowerinterval[j] <- 0.01
lowerinterval xmean
upperinterval
par(mar = c(5.1, 6, 4.1, 2.1)) plot.new() plot(Prop_dead_D3 ~ Density, data =
dat_AD_prop_dead3b, pch=16, ylim =c(0,0.1), col= " red", ylab= "Mean proportion of Adults\nthat
died on day 3", xlab= " Density (number of Adults/hamster)") # Beta regression lines(mu ~ Density,
data = dat_AD_prop_dead3b, col= "blue", lwd= 2) arrows(x0=dat_AD_prop_dead3b$Density,
y0=xmean - margin, x1=dat_AD_prop_dead3b$Density, y1=xmean + margin, angle=90, code=3,
length=0.05, lwd= 1, lty= 1, col= "blue")
# NOTE: The confidence interval lines are the ones corresponding to the predictions of the beta regression
# We check if the variance of the Beta regression is a function of density of bugs
Dens_Var <- cbind(dat_AD_prop_dead3b$Density, Var_y)
Dens_Var
pp <- lm(Var_y ~ dat_AD_prop_dead3b$Density)
summary(pp)
plot(Var_y ~ dat_AD_prop_dead3b$Density, pch=16)
abline(pp)
beta_res[i,1] <- INT
beta_res[i,2] <- REG
beta_res[i,3] <- PHI
beta_res[i,4] <- LL
beta_res[i,5] <- DOF
beta_res[i,6] <- R_2
beta_res[i,7] <- p_val_Dens
} # End of the "if" for i=7 (AD/Day3)
#$$$$$$$$$$$$$$$$$$$$$$$$$$$$$$$$$$$$$$$$$$$$$$$$$$$$$$$$$$$$$$$$$
$$$
if (i
==
8)
{
#################################################
```

```
##### Proportion Adults that died on day 3 #####
########################################
beta_reg_prop_dead_AD_3days <- betareg(prop_dead_3Days ~ Density, data = dat_AD_prop_dead3b)
summary(beta_reg_prop_dead_AD_3days)
#&&&&&&&&&&&&&&&&&&&&&&&&&&&&&&&&&&&&&&&&&&&&&&&&& &&
#####################################################
##
#####   Extract   from   the   beta   regression   results   the   parameters   of   interest   #####
####################################################
### thereg <- beta_reg_prop_dead_AD_3days
# For the Intercept
INT <- thereg[[1]]$mean[1]
names(INT) <-NULL
INT
#  For  the  regressor  (Density)
REG  <-  thereg[[1]]$mean[2]
names(REG) <-NULL
REG
# For the precision  (Phi)
PHI <- thereg[[1]]$precision[1]
names(PHI) <- NULL
PHI
#  The  predicted  values  (mu)
PRED       <-       thereg[[3]]
names(PRED) <- NULL
PRED
# Maximum-likelihood
LL <- thereg[[5]]$value
LL
# Degrees of freedom
DOF <- thereg[[14]]
DOF
# Pseudo R-squared
R_2 <- thereg[[20]]
R_2
# p-value of the regressor "Density"
p_val_Dens     <-     summary(thereg)$coefficients$mean[2,4]
p_val_Dens
```

```
#&&&&&&&&&&&&&&&&&&&&&&&&&&&&&&&&&&&&&&&&&&&&&&&&&&&&&& &&
################################################################
######## ###
####### Calculate the predicted values using the expected proportion E(y)=μ that #######
##### can be computed by applying the inverse link function to the linear predictor ######
##### Similarly we can also compute the variance to graph the confidence intervals ######
################################################################
########
###
mu      <-
PRED mu
phi  <-
PHI
phi
plot(prop_dead_3Days ~ Density, data = dat_AD_prop_dead3b, pch=16, ylim =c(0,0.25), ylab= " Mean
proportion of Adults\nthat died in 3 days", xlab= " Density (number of Adults/hamster)")
lines(mu ~ Density, data = dat_AD_prop_dead3b)
# Confidence Interval = x+tn-1, 1-α/2*(s/√n)
n <- 5
s <- sd_y
xmean <- mu
margin      <-      qt(0.975,df=n-1)*s/sqrt(n)
lowerinterval <- xmean - margin upperinterval
<- xmean + margin
for (j in 1:n) if(lowerinterval[j] <0) lowerinterval[j] <- 0.01
lowerinterval xmean
upperinterval
par(mar  =  c(5.1,  6,  4.1,  2.1))  plot.new()  plot(prop_dead_3Days  ~  Density,  data  =
dat_AD_prop_dead3b, pch=16, ylim =c(0,0.5), col= "red",  ylab= "Mean proportion of Adults\nthat
died in 3 days", xlab= " Density
(number   of   Adults/hamster)")   lines(mu   ~   Density,   data   =
dat_AD_prop_dead3b,         col=         "blue",         lwd=         2)
arrows(x0=dat_AD_prop_dead3b$Density,     y0=xmean     -     margin,
x1=dat_AD_prop_dead3b$Density, y1=xmean + margin, angle=90, code=3,
length=0.05, lwd= 1, lty= 1, col= "blue")
#  We  check  if  the  variance  of  the  Beta  regression  is  a  function  of  density  of  bugs  Dens_Var  <-
cbind(dat_AD_prop_dead3b$Density, Var_y) Dens_Var
pp     <-     lm(Var_y     ~     dat_AD_prop_dead3b$Density)
```

```
summary(pp)
plot(Var_y    ~    dat_AD_prop_dead3b$Density,    pch=16)
abline(pp)
beta_res[i,1]     <-     INT
beta_res[i,2]     <-     REG
beta_res[i,3]     <-     PHI
beta_res[i,4]     <-     LL
beta_res[i,5]     <-     DOF
beta_res[i,6]     <-     R_2
beta_res[i,7] <- p_val_Dens
} # End of the "if" for i=3 (N5/Day3)
#$$$$$$$$$$$$$$$$$$$$$$$$$$$$$$$$$$$$$$$$$$$$$$$$$$$$$$$$$$$$$$$$$
$$$
if (i
==
4)
{
##################################################
##### Proportion Adults that died on 3Days #####
##################################################
beta_reg_prop_dead_AD_3Days <- betareg(prop_dead_3Days ~ Density, data = dat_AD_prop_dead3b)
summary(beta_reg_prop_dead_AD_3Days)
#&&&&&&&&&&&&&&&&&&&&&&&&&&&&&&&&&&&&&&&&&&&&&&&&&&&&&&& &&
#################################################################
##
#####  Extract  from  the  beta  regression  results  the  parameters  of  interest  #####
################################################################
### thereg <- beta_reg_prop_dead_AD_3Days
# For the Intercept
INT     <-     thereg[[1]]$mean[1]
names(INT) <-NULL
INT
#  For  the  regressor  (Density)
REG  <-  thereg[[1]]$mean[2]
names(REG) <-NULL
REG
# For the precision  (Phi)
PHI <- thereg[[1]]$precision[1]
```

```
names(PHI) <- NULL
PHI
#  The  predicted  values  (mu)
PRED        <-        thereg[[3]]
names(PRED) <- NULL
PRED
# Maximum-likelihood
LL <- thereg[[5]]$value
LL
# Degrees of freedom
DOF <- thereg[[14]]
DOF
# Pseudo R-squared
R_2 <- thereg[[20]]
R_2
# p-value of the regressor "Density"
p_val_Dens     <-     summary(thereg)$coefficients$mean[2,4]
p_val_Dens
#&&&&&&&&&&&&&&&&&&&&&&&&&&&&&&&&&&&&&&&&&&&&&&&&&&&& &&
##############################################################
######## ###
####### Calculate the predicted values using the expected proportion E(y)=μ that #######
##### can be computed by applying the inverse link function to the linear predictor ######
##### Similarly we can also compute the variance to graph the confidence intervals ######
##############################################################
########
###
mu      <-
PRED mu
phi  <-
PHI
phi
plot(prop_dead_3Days ~ Density, data = dat_AD_prop_dead3b, pch=16, ylim =c(0,0.25), ylab= " Mean proportion of Adults\nthat died in 3 days", xlab= " Density (number of Adults/hamster)")
lines(mu ~ Density, data = dat_AD_prop_dead3b)
# Confidence Interval = x+tn-1, 1-α/2*(s/√n)
n <- 5
s <- sd_y
```

```
xmean <- mu
margin <- qt(0.975,df=n-1)*s/sqrt(n)
lowerinterval <- xmean - margin upperinterval
<- xmean + margin
for (j in 1:n) if(lowerinterval[j] <0) lowerinterval[j] <- 0.01
lowerinterval xmean
upperinterval
par(mar = c(5.1, 6, 4.1, 2.1)) plot.new() plot(prop_dead_3Days ~ Density, data =
dat_AD_prop_dead3b, pch=16, ylim =c(0,0.3), col= "red", ylab= "Mean proportion of Adults\nthat
died in 3 days", xlab= " Density
(number of Adults/hamster)") lines(mu ~ Density, data =
dat_AD_prop_dead3b, col= "blue", lwd= 2)
arrows(x0=dat_AD_prop_dead3b$Density, y0=xmean - margin,
x1=dat_AD_prop_dead3b$Density, y1=xmean + margin, angle=90, code=3,
length=0.05, lwd= 1, lty= 1, col= "blue")
# We check if the variance of the Beta regression is a function of density of bugs
Dens_Var <- cbind(dat_AD_prop_dead3b$Density, Var_y)
Dens_Var
pp <- lm(Var_y ~ dat_AD_prop_dead3b$Density)
summary(pp)
plot(Var_y ~ dat_AD_prop_dead3b$Density, pch=16)
abline(pp)
beta_res[i,1] <- INT
beta_res[i,2] <- REG
beta_res[i,3] <- PHI
beta_res[i,4] <- LL
beta_res[i,5] <- DOF
beta_res[i,6] <- R_2
beta_res[i,7] <- p_val_Dens
} # End of the "if" for i=4 (N5/3Days) } # This is the
end of the loop of i= 1:6 beta_res write.csv(beta_res,
file="beta_res_mortality.csv")
########## End for analysis of the proportion of bugs dead ##########
##############################################
##### Analysis of the proportion of bugs that Fed #####
##############################################
#########################################
##### Percent feeding in the three-day trials #####
```

```
###########################################
##################################
#####          Beta          regressions          only          for          N5          ####
##################################
dat_N5_Pct_feeding <- subset(feeding, feeding$Stage == 5) dat_N5_Pct_feeding
dat_N5_Pct_feeding2       <-       dat_N5_Pct_feeding[55:72,]
dat_N5_Pct_feeding2
pos3<- c(1,2,35:38)
dat_N5_Pct_feeding_3      <-      dat_N5_Pct_feeding2[,pos3]
dat_N5_Pct_feeding_3
prop_feeding_N5       <-       dat_N5_Pct_feeding_3       %>%
       group_by(Density) %>%
       summarise(Mean_prop_feeding_N5_Day1= mean(Pct_Fed_D1/100),
       Mean_prop_feeding_N5_Day2= mean(Pct_Fed_D2/100),
       Mean_prop_feeding_N5_Day3= mean(Pct_Fed_D3/100)) prop_feeding_N5
#############################################################
##### Create a matrix to hold the various beta regression results #####
#############################################################
# NOTE: Here the 6 cases of the beta_res matrix correspond to the following beta regressions
#                       N5_Day1, N5_Day2, N5_Day3, AD_Day1, AD_Day2, AD_Day3
beta_res <- data.frame(Intcpt = rep(0,6), Density = rep(0,6),Phi = rep(0,6), LL = rep(0,6),
DoF  =  rep(0,6),  Ps_R2  =  rep(0,6),  p_val_Dens  =  rep(0,6))
str(beta_res)
for   (i   in
   1:6)   {
   if(i   ==
   1) {
##################################################
##### Proportion Stage 5 nymphs that fed on Day 1 #####
##################################################
beta_reg_prop_feed_N5_Day1 <- betareg(Mean_prop_feeding_N5_Day1 ~ Density, data
= prop_feeding_N5)
summary(beta_reg_prop_feed_N5_Day1)
#&&&&&&&&&&&&&&&&&&&&&&&&&&&&&&&&&&&&&&&&&&&&&&&&&
&&
##################################################################
##
#####   Extract   from   the   beta   regression   results   the   parameters   of   interest   #####
```

```
#####################################################################
##
thereg <- beta_reg_prop_feed_N5_Day1
# For the Intercept
INT        <-        thereg[[1]]$mean[1]
names(INT) <-NULL
INT
#  For  the  regressor  (Density)
REG  <-  thereg[[1]]$mean[2]
names(REG) <-NULL
REG
# For the precision  (Phi)
PHI <- thereg[[1]]$precision[1]
names(PHI) <- NULL
PHI
#  The  predicted  values  (mu)
PRED        <-        thereg[[3]]
names(PRED) <- NULL
PRED
# Maximum-likelihood
LL <- thereg[[5]]$value
LL
# Degrees of freedom
DOF <- thereg[[14]]
DOF
# Pseudo R-squared
R_2 <- thereg[[20]]
R_2
# p-value of the regressor "Density"
p_val_Dens     <-     summary(thereg)$coefficients$mean[2,4]
p_val_Dens
#&&&&&&&&&&&&&&&&&&&&&&&&&&&&&&&&&&&&&&&&&&&&&&&&&&&&&&&&&&& &&
#####################################################################
######## ###
####### Calculate the predicted values using the expected proportion E(y)=μ that #######
##### can be computed by applying the inverse link function to the linear predictor ######
##### Similarly we can also compute the variance to graph the confidence intervals ######
#####################################################################
```

```
########
###
mu      <-
PRED mu
phi  <-
PHI
phi
# Prediction of the variance of the predicted proportion fed
Var_y <- mu * (1 - mu)/(1+phi)
Var_y
sd_y <- sqrt(Var_y)
sd_y
plot(Mean_prop_feeding_N5_Day1 ~ Density, data = prop_feeding_N5, pch=16, ylim =c(0
,0.8), ylab= " Mean proportion of stage 5 nymphs that fed on day 1", xlab= " Density
(number of stage 5 nymphs/hamster)") lines(mu ~
Density, data = prop_feeding_N5)
# Confidence Interval = x+tn-1, 1-α/2*(s/√n) n
<- 5
s <- sd_y
xmean <- mu
margin <- qt(0.975,df=n-1)*s/sqrt(n)
lowerinterval  <-  xmean  -  margin  upperinterval
<- xmean + margin
for  (j  in  1:n)  if(lowerinterval[j]  <0)  lowerinterval[j]  <-  0.01
for  (j  in  1:n)  if(upperinterval[j]  >1)  upperinterval[j]  <-  0.99
lowerinterval xmean
upperinterval
par(mar  =  c(5.1,  6,  4.1,  2.1))  plot.new()  plot(Mean_prop_feeding_N5_Day1  ~  Density,  data  =
prop_feeding_N5, pch=16, ylim =c(
0.4,0.8), col= " red", ylab= "Mean proportion of stage 5 nymphs\nthat fed on day 1", xlab = " Density
(number of stage 5 nymphs/hamster)")
#  Beta  regression  lines(mu  ~  Density,  data  =  prop_feeding_N5,  col=
"blue",   lwd=   3)   arrows(x0=prop_feeding_N5$Density,   y0=xmean   -
margin, x1=prop_feeding_N5$Density, y1=xmean + margin, angle=90,
code=3, length=0.05, lwd= 1, lty= 1, col= "blue")
# NOTE: The confidence interval lines are the ones corresponding to the predictions of the beta regression
# We check if the variance of the Beta regression is a function of density of bugs
Dens_Var <- cbind(prop_feeding_N5$Density, Var_y) Dens_Var
```

```
pp <- lm(Var_y ~ prop_feeding_N5$Density) summary(pp)
plot(Var_y ~ prop_feeding_N5$Density, pch=16) abline(pp)
#################################################################
######## ########
##### NOTE: The bug density has a significant effect on the variance in the results of Day 1 #####
#################################################################
########
########
beta_res[i,1]    <-
INT    beta_res[i,2]
<-                REG
beta_res[i,3]    <-
PHI    beta_res[i,4]
<- LL beta_res[i,5]
<-                DOF
beta_res[i,6]    <-
R_2    beta_res[i,7]
<- p_val_Dens
} # End of the "if" for i=1 (N5/Day1)
if (i == 2) {
################################################
#####    Proportion    Stage    5    nymphs    that    fed    on    Day    2    #####
################################################
beta_reg_prop_feed_N5_Day2 <- betareg(Mean_prop_feeding_N5_Day2 ~ Density, data
= prop_feeding_N5)
summary(beta_reg_prop_feed_N5_Day2)
#&&&&&&&&&&&&&&&&&&&&&&&&&&&&&&&&&&&&&&&&&&&&&&& &&
#################################################################
##
#####   Extract   from   the   beta   regression   results   the   parameters   of   interest   #####
#################################################################
### thereg <- beta_reg_prop_feed_N5_Day2
# For the Intercept
INT       <-       thereg[[1]]$mean[1]
names(INT) <-NULL
INT
#  For  the  regressor  (Density)
REG  <-  thereg[[1]]$mean[2]
```

```
names(REG) <-NULL
REG
# For the precision  (Phi)
PHI <- thereg[[1]]$precision[1]
names(PHI) <- NULL
PHI
#  The  predicted  values  (mu)
PRED        <-        thereg[[3]]
names(PRED) <- NULL
PRED
# Maximum-likelihood
LL <- thereg[[5]]$value
LL
# Degrees of freedom
DOF <- thereg[[14]]
DOF
# Pseudo R-squared
R_2 <- thereg[[20]]
R_2
# p-value of the regressor "Density"
p_val_Dens     <-     summary(thereg)$coefficients$mean[2,4]
p_val_Dens
#&&&&&&&&&&&&&&&&&&&&&&&&&&&&&&&&&&&&&&&&&&&&&&&&&&&&&&& &&
################################################################
######## ###
####### Calculate the predicted values using the expected proportion E(y)=μ that #######
##### can be computed by applying the inverse link function to the linear predictor ######
##### Similarly we can also compute the variance to graph the confidence intervals ######
################################################################
########
###
mu      <-
PRED mu
phi  <-
PHI
phi
# Prediction of the variance of the predicted proportion fed
Var_y <- mu * (1 - mu)/(1+phi)
```

```
Var_y
sd_y <- sqrt(Var_y)
# Confidence Interval = x+tn-1, 1-α/2*(s/√n) n
<- 5
s <- sd_y
xmean <- mu
margin       <-       qt(0.975,df=n-1)*s/sqrt(n)
lowerinterval <- xmean - margin upperinterval
<- xmean + margin
for (j in 1:n) if(lowerinterval[j] <0) lowerinterval[j] <- 0.01
for (j in 1:n) if(upperinterval[j] >1) upperinterval[j] <- 0.99
lowerinterval xmean
upperinterval
par(mar = c(5.1, 6, 4.1, 2.1)) plot.new() plot(Mean_prop_feeding_N5_Day2 ~ Density, data =
prop_feeding_N5, pch=16, ylim =c(0
,0.5), col= " red", ylab= "Mean proportion of stage 5 nymphs\nthat fed on day 2", xlab= "
Density (number of stage 5 nymphs/hamster)")
# Beta regression lines(mu ~ Density, data = prop_feeding_N5, col=
"blue", lwd= 2) arrows(x0=prop_feeding_N5$Density, y0=xmean -
margin, x1=prop_feeding_N5$Density, y1=xmean + margin, angle=90,
code=3, length=0.05, lwd= 1, lty= 1, col= "blue")
# NOTE: The confidence interval lines are the ones corresponding to the predictions of the beta regression
# We check if the variance of the Beta regression is a function of density of bugs
Dens_Var <- cbind(prop_feeding_N5$Density, Var_y) Dens_Var
pp  <-  lm(Var_y  ~  prop_feeding_N5$Density)
summary(pp)              plot(Var_y              ~
prop_feeding_N5$Density, pch=16) abline(pp)
##################################################################
######## ########
##### NOTE: The bug density has a significant effect on the variance in the results of Day 2 #####
##################################################################
########
########
beta_res[i,1]    <-    INT
beta_res[i,2]    <-    REG
beta_res[i,3]    <-    PHI
beta_res[i,4]    <-    LL
beta_res[i,5]    <-    DOF
```

```
beta_res[i,6]      <-      R_2
beta_res[i,7] <- p_val_Dens
} # End of the "if" for i=2 (N5/Day2)
#$$$$$$$$$$$$$$$$$$$$$$$$$$$$$$$$$$$$$$$$$$$$$$$$$$$$$$$$$$$$$$$$$$$$$$$$
$$$
if (i
==
3)
{
##################################################
#####     Proportion     Stage     5     nymphs     that     fed     on     Day     3     #####
##################################################
beta_reg_prop_feed_N5_Day3 <- betareg(Mean_prop_feeding_N5_Day3 ~ Density, data
= prop_feeding_N5)
summary(beta_reg_prop_feed_N5_Day3)
#&&&&&&&&&&&&&&&&&&&&&&&&&&&&&&&&&&&&&&&&&&&&&&&&&&&&&&&&&& &&
##################################################################
##
#####  Extract  from  the  beta  regression  results  the  parameters  of  interest  #####
#################################################################
### thereg <- beta_reg_prop_feed_N5_Day3
# For the Intercept
INT       <-       thereg[[1]]$mean[1]
names(INT) <-NULL
INT
#  For  the  regressor  (Density)
REG  <-  thereg[[1]]$mean[2]
names(REG) <-NULL
REG
# For the precision  (Phi)
PHI <- thereg[[1]]$precision[1]
names(PHI) <- NULL
PHI
#  The  predicted  values  (mu)
PRED       <-       thereg[[3]]
names(PRED) <- NULL
PRED
# Maximum-likelihood
```

```
LL <- thereg[[5]]$value
LL
# Degrees of freedom
DOF <- thereg[[14]]
DOF
# Pseudo R-squared
R_2 <- thereg[[20]]
R_2
# p-value of the regressor "Density"
p_val_Dens     <-     summary(thereg)$coefficients$mean[2,4]
p_val_Dens
#&&&&&&&&&&&&&&&&&&&&&&&&&&&&&&&&&&&&&&&&&&&&&&&&&&&&&&& &&
#################################################################
######## ###
####### Calculate the predicted values using the expected proportion E(y)=μ that #######
##### can be computed by applying the inverse link function to the linear predictor ######
##### Similarly we can also compute the variance to graph the confidence intervals ######
#################################################################
########
###
mu      <-
PRED mu
phi  <-
PHI
phi
plot(Mean_prop_feeding_N5_Day3 ~ Density, data = prop_feeding_N5, pch=16, ylim =c(0
,0.25), ylab= " Mean proportion of stage 5 nymphs\nthat fed on day 3", xlab= " Density
(number of stage 5 nymphs/hamster)") lines(mu ~
Density, data = prop_feeding_N5)
# Confidence Interval = x+tn-1, 1-α/2*(s/√n) n
<- 5
s <- sd_y
xmean <- mu
margin      <-      qt(0.975,df=n-1)*s/sqrt(n)
lowerinterval  <-  xmean  -  margin  upperinterval
<- xmean + margin
for  (j  in  1:n)  if(lowerinterval[j]  <0)  lowerinterval[j]  <-  0.01
for  (j  in  1:n)  if(upperinterval[j]  >1)  upperinterval[j]  <-  0.99
```

```
lowerinterval xmean
upperinterval
par(mar = c(5.1, 6, 4.1, 2.1)) plot.new() plot(Mean_prop_feeding_N5_Day3 ~ Density, data =
prop_feeding_N5, pch=16, ylim =c(0
,0.3), col= "red", ylab= "Mean proportion of stage 5 nymphs that fed on day 3", xlab= "
Density (number of stage 5 nymphs/hamster)")
# Beta regression lines(mu ~ Density, data = prop_feeding_N5, col=
"blue", lwd= 2) arrows(x0=prop_feeding_N5$Density, y0=xmean -
margin, x1=prop_feeding_N5$Density, y1=xmean + margin, angle=90,
code=3, length=0.05, lwd= 1, lty= 1, col= "blue")
# NOTE: The confidence interval lines are the ones corresponding to the predictions of the beta regression
# We check if the variance of the Beta regression is a function of density of bugs
Dens_Var <- cbind(prop_feeding_N5$Density, Var_y) Dens_Var
pp <- lm(Var_y ~ prop_feeding_N5$Density) summary(pp)
plot(Var_y ~ prop_feeding_N5$Density, pch=16) abline(pp)
beta_res[i,1]       <-       INT
beta_res[i,2]       <-       REG
beta_res[i,3]       <-       PHI
beta_res[i,4]       <-       LL
beta_res[i,5]       <-       DOF
beta_res[i,6]       <-       R_2
beta_res[i,7] <- p_val_Dens
} # End of the "if" for i=3 (N5/Day3)
#$$$$$$$$$$$$$$$$$$$$$$$$$$$$$$$$$$$$$$$$$$$$$$$$$$$$$$$$$$$$$$$$$$$$$$$$$$
$$$
if (i
==
4)
{
####################################
#####        Beta        regressions        only        for        Adults        #####
####################################
dat_ad_Pct_feeding <- subset(feeding, feeding$Stage == 6) dat_ad_Pct_feeding
dat_ad_Pct_feeding2      <-      dat_ad_Pct_feeding[69:85,1:9]
dat_ad_Pct_feeding2
dat_ad_Pct_feeding3      <-      dat_ad_Pct_feeding2[,-(3:5)]
dat_ad_Pct_feeding3
prop_feeding <- dat_ad_Pct_feeding3 %>% group_by(Density)
```

```
    %>%
    summarise(Mean_prop_feeding_Day1= mean(N_feeding_Day1/Density),
    Mean_prop_feeding_Day2= mean(N_feeding_Day2/Density),
    Mean_prop_feeding_Day3= mean(N_feeding_Day3/Density)) prop_feeding
########################################
#####   Proportion   of   adults   that   fed   on   Day   1   #####
########################################
beta_reg_prop_feed_Ad <- betareg(Mean_prop_feeding_Day1 ~ Density, data = prop_feeding)
summary(beta_reg_prop_feed_Ad)
#&&&&&&&&&&&&&&&&&&&&&&&&&&&&&&&&&&&&&&&&&&&&&& &&
############################################################
##
#####  Extract  from  the  beta  regression  results  the  parameters  of  interest  #####
############################################################
### thereg <- beta_reg_prop_feed_Ad
# For the Intercept
INT      <-      thereg[[1]]$mean[1]
names(INT) <-NULL
INT
#  For  the  regressor  (Density)
REG  <-  thereg[[1]]$mean[2]
names(REG) <-NULL
REG
# For the precision  (Phi)
PHI <- thereg[[1]]$precision[1]
names(PHI) <- NULL
PHI
# The predicted values (mu)
PRED      <-      thereg[[3]]
names(PRED) <- NULL
PRED
# Maximum-likelihood
LL <- thereg[[5]]$value
LL
# Degrees of freedom
DOF <- thereg[[14]]
DOF
# Pseudo R-squared
```

```
R_2 <- thereg[[20]]
R_2
# p-value of the regressor "Density"
p_val_Dens     <-     summary(thereg)$coefficients$mean[2,4]
p_val_Dens
#&&&&&&&&&&&&&&&&&&&&&&&&&&&&&&&&&&&&&&&&&&&&&&&&&&&&&&&&&&
&&
mu      <-
PRED mu
phi  <-
PHI
phi
# Prediction of the variance of the predicted proportion fed
Var_y <- mu * (1 - mu)/(1+phi)
Var_y
sd_y <- sqrt(Var_y)
sd_y
plot(Mean_prop_feeding_Day1 ~ Density, data = prop_feeding, pch=16, ylim =c(0,1), ylab
= " Mean proportion of adults\nthat fed on day 1", xlab= " Density (number of adults/hamster)")
lines(mu ~ Density, data = prop_feeding_N5)
# Confidence Interval = x+tn-1, 1-α/2*(s/√n) n
<- 5
s <- sd_y
xmean <- mu
margin      <-      qt(0.975,df=n-1)*s/sqrt(n)
lowerinterval <- xmean - margin upperinterval
<- xmean + margin
for (j in 1:n) if(lowerinterval[j] <0) lowerinterval[j] <- 0.01
lowerinterval xmean
upperinterval
par(mar = c(5.1, 6, 4.1, 2.1)) plot.new() plot(Mean_prop_feeding_N5_Day1 ~ Density, data =
prop_feeding_N5, pch=16, ylim =c(0 ,1), col= "red",  ylab= "Mean proportion of adults\nthat fed on day
1", xlab= " Density (number of adults/hamster)")
# Beta regression lines(mu ~ Density, data = prop_feeding_N5, col=
"blue", lwd= 2) arrows(x0=prop_feeding_N5$Density, y0=xmean -
margin, x1=prop_feeding_N5$Density, y1=xmean + margin, angle=90,
code=3, length=0.05, lwd= 1, lty= 1, col= "blue")
# NOTE: The confidence interval lines are the ones corresponding to the predictions of the beta regression
```

```
# We check if the variance of the Beta regression is a function of density of bugs
Dens_Var <- cbind(prop_feeding$Density, Var_y)
Dens_Var
pp <- lm(Var_y ~ prop_feeding$Density) summary(pp)
plot(Var_y ~ prop_feeding_N5$Density, pch=16) abline(pp)
beta_res[i,1]      <-      INT
beta_res[i,2]      <-      REG
beta_res[i,3]      <-      PHI
beta_res[i,4]      <-      LL
beta_res[i,5]      <-      DOF
beta_res[i,6]      <-      R_2
beta_res[i,7] <- p_val_Dens
} # End of the "if" for i=4 (AD/Day1)
#$$$$$$$$$$$$$$$$$$$$$$$$$$$$$$$$$$$$$$$$$$$$$$$$$$$$$$$$$$$$$$$$$$$$$$$$$$$$$$
$$$
if (i
==
5)
{
#######################################
#####      Proportion      of      adults      that      fed      on      Day      2      #####
#######################################
beta_reg_prop_feed_Ad <- betareg(Mean_prop_feeding_Day2 ~ Density, data = prop_feeding)
summary(beta_reg_prop_feed_Ad)
thereg <- beta_reg_prop_feed_Ad
# For the Intercept
INT      <-      thereg[[1]]$mean[1]
names(INT) <-NULL
INT
#  For  the  regressor  (Density)
REG  <-  thereg[[1]]$mean[2]
names(REG) <-NULL
REG
# For the precision  (Phi)
PHI <- thereg[[1]]$precision[1]
names(PHI) <- NULL
PHI
#  The  predicted  values  (mu)
```

```
PRED <- thereg[[3]]
names(PRED) <- NULL
PRED
# Maximum-likelihood
LL <- thereg[[5]]$value
LL
# Degrees of freedom
DOF <- thereg[[14]]
DOF
# Pseudo R-squared
R_2 <- thereg[[20]]
R_2
# p-value of the regressor "Density"
p_val_Dens <- summary(thereg)$coefficients$mean[2,4]
p_val_Dens
#&&&&&&&&&&&&&&&&&&&&&&&&&&&&&&&&&&&&&&&&&&&&&&&&&&&&
&&
mu
<-
PRED
mu
phi <-
PHI
phi
# Prediction of the variance of the predicted proportion fed
Var_y <- mu * (1 - mu)/(1+phi)
Var_y
sd_y <- sqrt(Var_y)
sd_y
plot(Mean_prop_feeding_Day2 ~ Density, data = prop_feeding, pch=16, ylim =c(0,0.3), ylab= " Mean
proportion of adults\nthat fed on day 2", xlab= " Density (number of adults/hamster)")
lines(mu ~ Density, data = prop_feeding)
# Confidence Interval = x+tn-1, 1-α/2*(s/√n) n
<- 5
s <- sd_y
xmean <- mu
margin <- qt(0.975,df=n-1)*s/sqrt(n)
lowerinterval <- xmean - margin upperinterval
```

```
<- xmean + margin
for (j in 1:n) if(lowerinterval[j] <0) lowerinterval[j] <- 0.01
lowerinterval
xmean
upperinterval
par(mar = c(5.1, 6, 4.1, 2.1)) plot.new() plot(Mean_prop_feeding_N5_Day2 ~ Density, data =
prop_feeding_N5, pch=16, ylim =c(0 ,0.3), col= "red", ylab= "Mean proportion of adults\nthat fed on
day 2", xlab= " Density (number of adults/hamster)")
# Beta regression lines(mu ~ Density, data = prop_feeding, col=
"blue", lwd= 2) arrows(x0=prop_feeding_N5$Density, y0=xmean -
margin, x1=prop_feeding_N5$Density, y1=xmean + margin,
angle=90, code=3, length=0.05, lwd= 1, lty= 1, col= "blue")
# NOTE: The confidence interval lines are the ones corresponding to the predictions of the beta regression
# We check if the variance of the Beta regression is a function of density of bugs
Dens_Var <- cbind(prop_feeding$Density, Var_y)
Dens_Var
pp <- lm(Var_y ~ prop_feeding$Density) summary(pp)
plot(Var_y ~ prop_feeding_N5$Density, pch=16) abline(pp)
beta_res[i,1] <- INT
beta_res[i,2] <- REG
beta_res[i,3] <- PHI
beta_res[i,4] <- LL
beta_res[i,5] <- DOF
beta_res[i,6] <- R_2
beta_res[i,7] <- p_val_Dens
} # End of the "if" for i=5 (AD/Day2)
#$$$$$$$$$$$$$$$$$$$$$$$$$$$$$$$$$$$$$$$$$$$$$$$$$$$$$$$$$$$$$$$$$$$$$ $$$
if (i == 6) {
########################################
##### Proportion of adults that fed on Day 3 #####
########################################
beta_reg_prop_feed_Ad <- betareg(Mean_prop_feeding_Day3 ~ Density, data = prop_feeding)
summary(beta_reg_prop_feed_Ad)
thereg <- beta_reg_prop_feed_Ad
# For the Intercept
INT <- thereg[[1]]$mean[1]
names(INT) <-NULL
INT
```

```
#  For  the  regressor  (Density)
REG  <-  thereg[[1]]$mean[2]
names(REG) <-NULL
REG
# For the precision  (Phi) PHI
<-  thereg[[1]]$precision[1]
names(PHI) <- NULL
PHI
#  The  predicted  values  (mu)
PRED        <-        thereg[[3]]
names(PRED) <- NULL
PRED
# Maximum-likelihood
LL <- thereg[[5]]$value
LL
# Degrees of freedom
DOF <- thereg[[14]]
DOF
# Pseudo R-squared
R_2 <- thereg[[20]]
R_2
# p-value of the regressor "Density"
p_val_Dens <- summary(thereg)$coefficients$mean[2,4]
p_val_Dens
#&&&&&&&&&&&&&&&&&&&&&&&&&&&&&&&&&&&&&&&&&&&&&&&&&&&&
&&
mu
<-
PRED
mu
phi  <-
PHI
phi
# Prediction of the variance of the predicted proportion fed
Var_y <- mu * (1 - mu)/(1+phi)
Var_y
sd_y <- sqrt(Var_y)
sd_y
```

```
plot(Mean_prop_feeding_Day3 ~ Density, data = prop_feeding, pch=16, ylim =c(0,0.3), ylab= " Mean
proportion of adults\nthat fed on day 3", xlab= " Density (number of adults/hamster)")
lines(mu ~ Density, data = prop_feeding)
# Confidence Interval = x+tn-1, 1-α/2*(s/√n)
n <- 5
s <- sd_y
xmean <- mu
margin      <-      qt(0.975,df=n-1)*s/sqrt(n)
lowerinterval <- xmean - margin upperinterval
<- xmean + margin
for (j in 1:n) if(lowerinterval[j] <0) lowerinterval[j] <- 0.01
lowerinterval xmean
upperinterval
par(mar = c(5.1, 6, 4.1, 2.1)) plot.new() plot(Mean_prop_feeding_Day3 ~ Density, data =
prop_feeding, pch=16, ylim =c(0,0.2), col= "red", ylab= "Mean proportion of adults\nthat fed on
day 3", xlab= " Density (number of adults/hamster)")
# Beta regression lines(mu ~ Density, data = prop_feeding, col=
"blue", lwd= 2) arrows(x0=prop_feeding$Density, y0=xmean -
margin, x1=prop_feeding$Density, y1=xmean + margin,
      angle=90, code=3, length=0.05, lwd= 1, lty= 1, col= "blue")
# NOTE: The confidence interval lines are the ones corresponding to the predictions of the beta regression
# We check if the variance of the Beta regression is a function of density of bugs
Dens_Var <- cbind(prop_feeding$Density, Var_y)
Dens_Var
pp <- lm(Var_y ~ prop_feeding$Density) summary(pp)
plot(Var_y ~ prop_feeding_N5$Density, pch=16) abline(pp)
beta_res[i,1]      <-      INT
beta_res[i,2]      <-      REG
beta_res[i,3]      <-      PHI
beta_res[i,4]      <-      LL
beta_res[i,5]      <-      DOF
beta_res[i,6]      <-      R_2
beta_res[i,7] <- p_val_Dens
} # End of the "if" for i=6 (AD/Day3)
} # This is the end of the loop of i=
1:6 beta_res
########## End for the analysis of the proportion of bugs that Fed ##########
#################################################################
```

```
##### Analysis of the proportion of bugs that fed once, twice or thrice #####
###############################################################
## position <- c(1:5, 47:53, 74:86, 91:103)
feeding <- dat[,position]
# We select the appropriate variables from N5 and Ad
names(feeding) position <- c(1:4, 10:12) feeding_freq <-
feeding[,position]
feeding_freq2 <- feeding_freq[,-4] # Delete Sex feeding_n5_freq <-
subset(feeding_freq2, feeding_freq2$Stage == 5) feeding_ad_freq <-
subset(feeding_freq2, feeding_freq2$Stage == 6)
##### Adults #####
feeding_ad_freq2 <- feeding_ad_freq[complete.cases(feeding_ad_freq), ] # Delete rows with NA
# Convert percentages into proportions and group by density for adults
feeding_ad_freq3_0 <- feeding_ad_freq2 %>% group_by(Density) %>%
  mutate(Prop_feeding_once_in_3d = Pct_feeding_once_in_3d/100,
  Prop_feeding_twice_in_3d = Pct_feeding_twice_in_3d/100,
  Prop_feeding_thrice_in_3d = Pct_feeding_thrice_in_3d/100) feeding_ad_freq3_0 <-
as.data.frame(feeding_ad_freq3_0) feeding_ad_freq3_0
# We calculate the means among replicates feeding_ad_freq3
<- feeding_ad_freq3_0 %>% group_by(Density) %>%
  summarise(Prop_feeding_once_in_3d = mean(Prop_feeding_once_in_3d),
            Prop_feeding_twice_in_3d = mean(Prop_feeding_twice_in_3d),
            Prop_feeding_thrice_in_3d = mean(Prop_feeding_thrice_in_3d))
feeding_ad_freq3
# NOTE: Beta regression can be applied opnly for those bugs that fed once and twice
# because for bugs that fed thrice in three days there are many zeroes (not permitted)
##### Stage 5 nymphs #####
feeding_n5_freq2 <- feeding_n5_freq[complete.cases(feeding_n5_freq), ] # Delete rows with NA
# Convert percentages into proportions and group by density for n5ults feeding_n5_freq3_0 <-
feeding_n5_freq2 %>%
  group_by(Density) %>% mutate(Prop_feeding_once_in_3d =
  Pct_feeding_once_in_3d/100,
  Prop_feeding_twice_in_3d = Pct_feeding_twice_in_3d/100,
  Prop_feeding_thrice_in_3d = Pct_feeding_thrice_in_3d/100) feeding_n5_freq3_0 <-
as.data.frame(feeding_n5_freq3_0) feeding_n5_freq3_0
# We calculate the means among replicates feeding_n5_freq3 <-
feeding_n5_freq3_0 %>%
  group_by(Density) %>%
```

```
    summarise(Prop_feeding_once_in_3d = mean(Prop_feeding_once_in_3d),
              Prop_feeding_twice_in_3d = mean(Prop_feeding_twice_in_3d),
              Prop_feeding_thrice_in_3d = mean(Prop_feeding_thrice_in_3d))
feeding_n5_freq3
# NOTE: Beta regression can be applied opnly for those bugs that fed once and twice
# because for bugs that fed thrice in three days there are many zeroes (not permitted)
##########################################################
##### Create a matrix to hold the various beta regression results #####
##########################################################
# NOTE: Here the 4 cases of the beta_res matrix correspond to the following beta regressions
#       For N5: Prop_feeding_once_in_3d and Prop_feeding_twice_in_3d #     For     Ad:
Prop_feeding_once_in_3d and Prop_feeding_twice_in_3d
beta_res <- data.frame(Intcpt = rep(0,4), Density = rep(0,4),Phi = rep(0,4), LL = rep(0,4),
DoF = rep(0,4), Ps_R2 = rep(0,4), p_val_Dens = rep(0,4))
str(beta_res)
for (i in
   1:4) {
   if(i ==
   1) {
###################################################
#####    Proportion    Stage    5    nymphs    that    fed    once    in    3    days    ####
###################################################
beta_reg_prop_fed_once <- betareg(Prop_feeding_once_in_3d ~ Density, data = feeding_n5_freq3)
summary(beta_reg_prop_fed_once)
###########################################################
##
#####   Extract   from   the   beta   regression   results   the   parameters   of   interest   #####
###########################################################
### thereg <- beta_reg_prop_fed_once
# For the Intercept
INT <- thereg[[1]]$mean[1]; names(INT) <-NULL
# For the regressor (Density)
REG <- thereg[[1]]$mean[2]; names(REG) <-NULL
# For the precision  (Phi)
PHI <- thereg[[1]]$precision[1]; names(PHI) <- NULL
# The predicted values (mu)
PRED <- thereg[[3]]; names(PRED) <- NULL
# Maximum-likelihood
```

```
LL <- thereg[[5]]$value; LL
# Degrees of freedom
DOF <- thereg[[14]]; DOF
# Pseudo R-squared
R_2 <- thereg[[20]]; R_2
# p-value of the regressor "Density"
p_val_Dens <- summary(thereg)$coefficients$mean[2,4]; p_val_Dens
##################################################################
######## ###
####### Calculate the predicted values using the expected proportion E(y)=μ that #######
##### can be computed by applying the inverse link function to the linear predictor ######
##### Similarly we can also compute the variance to graph the confidence intervals ######
##################################################################
########
###
mu
<-
PRED
phi <- PHI
# Prediction of the variance of the predicted proportion fed
Var_y <- mu * (1 - mu)/(1+phi)
sd_y <- sqrt(Var_y)
plot(Prop_feeding_once_in_3d ~ Density, data = feeding_n5_freq3, pch=16, ylim =c(0,1), ylab=
"Mean proportion of stage 5 nymphs\nthat fed only once in a three-day period", xlab= " Density
(number of stage 5 nymphs/hamster)") lines(mu ~ Density, data = feeding_n5_freq3)
# Confidence Interval = x+tn-1, 1-α/2*(s/√n)
n <- 5; s <- sd_y; xmean <- mu margin <-
qt(0.975,df=n-1)*s/sqrt(n) lowerinterval <-
xmean - margin upperinterval <- xmean +
margin
for (j in 1:n) if(lowerinterval[j] <0) lowerinterval[j] <- 0.01
lowerinterval xmean
upperinterval
par(mar = c(5.1, 6, 4.1, 2.1))
plot.new()
plot(Prop_feeding_once_in_3d ~ Density, data = feeding_n5_freq3, pch=16, ylim =c(0,1), col= " red",
ylab= "Mean proportion of stage 5 nymphs\nthat fed only once in a three-day period", xlab= " Density
(number of stage 5 nymphs/hamster)") lines(mu ~ Density, data = feeding_n5_freq3, col= "blue",
```

```
lwd= 3) arrows(x0=feeding_n5_freq3$Density, y0=xmean - margin, x1=feeding_n5_freq3$Density,
y1=xmean + margin, angle=90, code=3, length=0.05, lwd= 1, lty= 1, col= "blue")
# We check if the variance of the Beta regression is a function of density of bugs
Dens_Var <- cbind(feeding_n5_freq3$Density, Var_y)
Dens_Var
pp <- lm(Var_y ~ feeding_n5_freq3$Density) summary(pp)
plot(Var_y ~ feeding_n5_freq3$Density, pch=16) abline(pp)
   } # End of combination 1 (n5 fed once in 3 days)
beta_res[i,1]      <-      INT
beta_res[i,2]      <-      REG
beta_res[i,3]      <-      PHI
beta_res[i,4]      <-      LL
beta_res[i,5]      <-      DOF
beta_res[i,6]      <-      R_2
beta_res[i,7] <- p_val_Dens
   if(i == 2) {
##################################################
####    Proportion    Stage    5    nymphs    that    fed    twice    in    3    days    ####
##################################################
beta_reg_prop_fed_twice <- betareg(Prop_feeding_twice_in_3d ~ Density, data = feeding_n5_freq3)
summary(beta_reg_prop_fed_twice)
##############################################################
##
#####   Extract   from   the   beta   regression   results   the   parameters   of   interest   #####
##############################################################
### thereg <- beta_reg_prop_fed_twice
# For the Intercept
INT <- thereg[[1]]$mean[1]; names(INT) <-NULL
# For the regressor (Density)
REG <- thereg[[1]]$mean[2]; names(REG) <-NULL
# For the precision  (Phi)
PHI <- thereg[[1]]$precision[1]; names(PHI) <- NULL
# The predicted values (mu)
PRED <- thereg[[3]]; names(PRED) <- NULL
# Maximum-likelihood
LL <- thereg[[5]]$value; LL
# Degrees of freedom
DOF <- thereg[[14]]; DOF
```

```
# Pseudo R-squared
R_2 <- thereg[[20]]; R_2
# p-value of the regressor "Density"
p_val_Dens <- summary(thereg)$coefficients$mean[2,4]; p_val_Dens
################################################################
######## ###
####### Calculate the predicted values using the expected proportion E(y)=μ that #######
##### can be computed by applying the inverse link function to the linear predictor ######
##### Similarly we can also compute the variance to graph the confidence intervals ######
################################################################
########
###
mu
<-
PRED
phi <- PHI
# Prediction of the variance of the predicted proportion fed
Var_y <- mu * (1 - mu)/(1+phi)
sd_y <- sqrt(Var_y)
plot(Prop_feeding_twice_in_3d ~ Density, data = feeding_n5_freq3, pch=16, ylim =c(0,0.5
), ylab= "Mean proportion of stage 5 nymphs\nthat fed twice in a three-day period", xlab
= " Density (number of stage 5 nymphs/hamster)") lines(mu ~
Density, data = feeding_n5_freq3)
# Confidence Interval = x+tn-1, 1-ɑ/2*(s/√n)
n <- 5; s <- sd_y; xmean <- mu margin <-
qt(0.975,df=n-1)*s/sqrt(n) lowerinterval <-
xmean - margin upperinterval <- xmean +
margin
for (j in 1:n) if(lowerinterval[j] <0) lowerinterval[j] <- 0.01
lowerinterval xmean
upperinterval
par(mar = c(5.1, 6, 4.1, 2.1)) plot.new() plot(Prop_feeding_twice_in_3d ~ Density, data =
feeding_n5_freq3, pch=16, ylim =c(0,0.5 ), col= " red", ylab= "Mean proportion of stage 5
nymphs\nthat fed twice in a three-day period", xlab= " Density (number of stage 5 nymphs/hamster)")
lines(mu ~ Density, data = feeding_n5_freq3, col= "blue", lwd= 3)
arrows(x0=feeding_n5_freq3$Density, y0=xmean - margin, x1=feeding_n5_freq3$Density, y1=xmean
+ margin, angle=90, code=3, length=0.05, lwd= 1, lty= 1, col= "blue")
# We check if the variance of the Beta regression is a function of density of bugs
```

```
Dens_Var <- cbind(feeding_n5_freq3$Density, Var_y)
Dens_Var
pp <- lm(Var_y ~ feeding_n5_freq3$Density) summary(pp)
plot(Var_y ~ feeding_n5_freq3$Density, pch=16) abline(pp)
  } # End of combination 2 (n5 fed twice in 3 days)
beta_res[i,1]    <-    INT
beta_res[i,2]    <-    REG
beta_res[i,3]    <-    PHI
beta_res[i,4]    <-    LL
beta_res[i,5]    <-    DOF
beta_res[i,6]    <-    R_2
beta_res[i,7] <- p_val_Dens
if(i == 3) {
############################################
#####    Proportion    adults    that    fed    once    in    3    days    ####
############################################
beta_reg_prop_fed_once <- betareg(Prop_feeding_once_in_3d ~ Density, data = feeding_ad_freq3)
summary(beta_reg_prop_fed_once)
#################################################################
##
#####  Extract  from  the  beta  regression  results  the  parameters  of  interest  #####
################################################################
### thereg <- beta_reg_prop_fed_once
# For the Intercept
INT <- thereg[[1]]$mean[1]; names(INT) <-NULL
# For the regressor (Density)
REG <- thereg[[1]]$mean[2]; names(REG) <-NULL
# For the precision  (Phi)
PHI <- thereg[[1]]$precision[1]; names(PHI) <- NULL
# The predicted values (mu)
PRED <- thereg[[3]]; names(PRED) <- NULL
# Maximum-likelihood
LL <- thereg[[5]]$value; LL
# Degrees of freedom
DOF <- thereg[[14]]; DOF
# Pseudo R-squared
R_2 <- thereg[[20]]; R_2
# p-value of the regressor "Density"
```

```
p_val_Dens <- summary(thereg)$coefficients$mean[2,4]; p_val_Dens
#################################################################
######## ###
####### Calculate the predicted values using the expected proportion E(y)=μ that #######
##### can be computed by applying the inverse link function to the linear predictor ######
##### Similarly we can also compute the variance to graph the confidence intervals ######
#################################################################
########
###
mu
<-
PRED
phi <- PHI
# Prediction of the variance of the predicted proportion fed
Var_y <- mu * (1 - mu)/(1+phi)
sd_y <- sqrt(Var_y)
plot(Prop_feeding_once_in_3d ~ Density, data = feeding_ad_freq3, pch=16, ylim =c(0,1), ylab= "Mean
proportion of adults\nthat fed only once in a three-day period", xlab= "
Density (number of stage 5 nymphs/hamster)") lines(mu ~
Density, data = feeding_ad_freq3)
# Confidence Interval = x+tn-1, 1-α/2*(s/√n)
n <- 5; s <- sd_y; xmean <- mu margin <-
qt(0.975,df=n-1)*s/sqrt(n) lowerinterval <-
xmean - margin upperinterval <- xmean +
margin
for (j in 1:n) if(lowerinterval[j] <0) lowerinterval[j] <- 0.01
lowerinterval xmean
upperinterval
par(mar = c(5.1, 6, 4.1, 2.1)) plot.new() plot(Prop_feeding_once_in_3d ~ Density, data =
feeding_ad_freq3, pch=16, ylim =c(0,1), col= " red", ylab= "Mean proportion of adults\nthat fed only
once in a three-day period", xlab= " Density (number of stage 5 nymphs/hamster)") lines(mu ~
Density, data = feeding_ad_freq3, col= "blue", lwd= 3) arrows(x0=feeding_ad_freq3$Density,
y0=xmean - margin, x1=feeding_ad_freq3$Density, y1=xmean + margin, angle=90, code=3,
length=0.05, lwd= 1, lty= 1, col= "blue")
# We check if the variance of the Beta regression is a function of density of bugs
Dens_Var <- cbind(feeding_ad_freq3$Density, Var_y)
Dens_Var
pp <- lm(Var_y ~ feeding_ad_freq3$Density) summary(pp)
```

```
plot(Var_y ~ feeding_ad_freq3$Density, pch=16) abline(pp)
   } # End of combination 3 (adults fed once in 3 days)
beta_res[i,1]      <-      INT
beta_res[i,2]      <-      REG
beta_res[i,3]      <-      PHI
beta_res[i,4]      <-      LL
beta_res[i,5]      <-      DOF
beta_res[i,6]      <-      R_2
beta_res[i,7]  <-  p_val_Dens
if(i == 4) {
##########################################
####     Proportion     adults     that     fed     twice     in     3     days     ####
##########################################
beta_reg_prop_fed_twice <- betareg(Prop_feeding_twice_in_3d ~ Density, data = feeding_ad_freq3)
summary(beta_reg_prop_fed_twice)
################################################################
##
#####   Extract   from   the   beta   regression   results   the   parameters   of   interest   #####
###############################################################
### thereg <- beta_reg_prop_fed_twice
# For the Intercept
INT <- thereg[[1]]$mean[1]; names(INT) <-NULL
# For the regressor (Density)
REG <- thereg[[1]]$mean[2]; names(REG) <-NULL
# For the precision  (Phi)
PHI <- thereg[[1]]$precision[1]; names(PHI) <- NULL
# The predicted values (mu)
PRED <- thereg[[3]]; names(PRED) <- NULL
# Maximum-likelihood
LL <- thereg[[5]]$value; LL
# Degrees of freedom
DOF <- thereg[[14]]; DOF
# Pseudo R-squared
R_2 <- thereg[[20]]; R_2
# p-value of the regressor "Density"
p_val_Dens <- summary(thereg)$coefficients$mean[2,4]; p_val_Dens
################################################################
######## ###
```

```
####### Calculate the predicted values using the expected proportion E(y)=μ that #######
##### can be computed by applying the inverse link function to the linear predictor ######
##### Similarly we can also compute the variance to graph the confidence intervals ######
######################################################################
########
###
mu
<-
PRED
phi <- PHI
# Prediction of the variance of the predicted proportion fed
Var_y <- mu * (1 - mu)/(1+phi)
sd_y <- sqrt(Var_y)
plot(Prop_feeding_twice_in_3d ~ Density, data = feeding_ad_freq3, pch=16, ylim =c(0,0.5
), ylab= "Mean proportion of stage 5 nymphs\nthat fed twice in a three-day period", xlab
= " Density (number of stage 5 nymphs/hamster)") lines(mu ~
Density, data = feeding_ad_freq3)
# Confidence Interval = x+tn-1, 1-α/2*(s/√n)
n <- 5; s <- sd_y; xmean <- mu margin <-
qt(0.975,df=n-1)*s/sqrt(n) lowerinterval <-
xmean - margin upperinterval <- xmean +
margin
for (j in 1:n) if(lowerinterval[j] <0) lowerinterval[j] <- 0.01
lowerinterval xmean
upperinterval
par(mar = c(5.1, 6, 4.1, 2.1)) plot.new() plot(Prop_feeding_twice_in_3d ~ Density, data =
feeding_ad_freq3, pch=16, ylim =c(0,0.5 ), col= " red", ylab= "Mean proportion of adults\nthat fed
twice in a three-day period", xlab= " Density (number of stage 5 nymphs/hamster)") lines(mu ~
Density, data = feeding_ad_freq3, col= "blue", lwd= 3) arrows(x0=feeding_ad_freq3$Density,
y0=xmean - margin, x1=feeding_ad_freq3$Density, y1=xmean + margin, angle=90, code=3,
length=0.05, lwd= 1, lty= 1, col= "blue")
# We check if the variance of the Beta regression is a function of density of bugs
Dens_Var <- cbind(feeding_ad_freq3$Density, Var_y)
Dens_Var
pp <- lm(Var_y ~ feeding_ad_freq3$Density) summary(pp)
plot(Var_y ~ feeding_ad_freq3$Density, pch=16) abline(pp)
beta_res[i,1] <- INT
beta_res[i,2] <- REG
```

```
beta_res[i,3]      <-       PHI
beta_res[i,4]       <-       LL
beta_res[i,5]      <-       DOF
beta_res[i,6]      <-       R_2
beta_res[i,7] <- p_val_Dens
    } # End of combination 2 (n5 fed twice in 3 days)
} # End of loop for the four combinations beta_res
########## End for the analysis of the proportion of bugs that fed  once, twice or thrice
##########
```
